# Total Flavones of *Abelmoschus manihot* Ameliorates Podocyte Pyroptosis and Injury in High Glucose Conditions by Targeting METTL3-Dependent m^6^A Modification-Mediated NLRP3-Inflammasome Activation and PTEN/PI3K/Akt Signaling

**DOI:** 10.3389/fphar.2021.667644

**Published:** 2021-07-15

**Authors:** Bu-Hui Liu, Yue Tu, Guang-Xia Ni, Jin Yan, Liang Yue, Zi-Lin Li, Jing-Jing Wu, Yu-Ting Cao, Zi-Yue Wan, Wei Sun, Yi-Gang Wan

**Affiliations:** ^1^ Nephrology Division, Affiliated Hospital of Nanjing University of Chinese Medicine, Nanjing, China; ^2^ Department of Traditional Chinese Medicine, Nanjing Drum Tower Hospital Clinical College of Nanjing University of Chinese Medicine, Nanjing, China; ^3^ Department of Traditional Chinese Medicine Health Preservation, Acupuncture, Moxibustion and Massage College, Health Preservation and Rehabilitation College, Nanjing University of Chinese Medicine, Nanjing, China; ^4^ Graduate School of Social Sciences, Faculty of Social Sciences, Hitotsubashi University, Tokyo, Japan

**Keywords:** diabetic kidney disease, total flavones of *Abelmoschus manihot*, podocyte pyroptosis, NLRP3-inflammasome activation, PTEN/PI3K/Akt signaling, m^6^A modification

## Abstract

**Background:** The total flavones of *Abelmoschus manihot* (TFA), a compound that is extracted from *Abelmoschus manihot*, has been widely used in China to reduce podocyte injury in diabetic kidney disease (DKD). However, the mechanisms underlying the therapeutic action of this compound have yet to be elucidated. Podocyte pyroptosis is characterized by activation of the NLRP3 inflammasome and plays an important role in inflammation-mediated diabetic kidneys. Regulation of the PTEN/PI3K/Akt pathway is an effective strategy for improving podocyte damage in DKD. Previous research has also shown that N6-methyladenosine (m^6^A) modification is involved in DKD and that m^6^A-modified PTEN regulates the PI3K/Akt pathway. In this study, we investigated whether TFA alleviates podocyte pyroptosis and injury by targeting m^6^A modification-mediated NLRP3-inflammasome activation and PTEN/PI3K/Akt signaling.

**Methods:** We used MPC-5 cells under high glucose (HG) conditions to investigate the key molecules that are involved in podocyte pyroptosis and injury, including activation of the NLRP3 inflammasome and the PTEN/PI3K/Akt pathway. We detected alterations in the levels of three methyltransferases that are involved in m^6^A modification. We also investigated changes in the levels of these key molecules in podocytes with the overexpression or knockdown of methyltransferase-like (METTL)3.

**Results:** Analysis showed that TFA and MCC950 protected podocytes against HG-induced pyroptosis and injury by reducing the protein expression levels of gasdermin D, interleukin-1β, and interleukin-18, and by increasing the protein expression levels of nephrin, ZO-1, WT1 and podocalyxin. TFA and 740Y-P inhibited activation of the NLRP3 inflammasome *via* the PI3K/Akt pathway by inhibiting the protein levels of NIMA-related kinase7, NLRP3, ASC, and caspase-1, and by increasing the protein expression levels of p-PI3K and p-Akt. TFA improved pyroptosis and injury in HG-stimulated podocytes by regulating METTL3-dependent m^6^A modification.

**Conclusion:** Collectively, our data indicated that TFA could ameliorate pyroptosis and injury in podocytes under HG conditions by adjusting METTL3-dependent m^6^A modification and regulating NLRP3-inflammasome activation and PTEN/PI3K/Akt signaling. This study provides a better understanding of how TFA can protect podocytes in DKD.

## Introduction

Diabetic kidney disease (DKD) is the most common microvascular complication of diabetes and is one of the primary causes of end-stage renal disease (ESRD) (Kato and Natarajan, 2019). It is therefore necessary to investigate the pathogenesis of DKD and identify effective therapies to prevent the progression of DKD to ESRD. DKD is characterized by glomerulosclerosis, tubulointerstitial fibrosis, and renal vascular disease (Shi and Hu, 2014). Podocytes are one of the key cell types in the glomerulus and play a vital role in protecting the glomerular filtration barrier. Podocytes are thought to play a fundamental role in the pathophysiology of DKD. Therapies that are able to protect podocytes are considered to be of vital importance for the treatment of DKD. Podocyte injury is characterized by the downregulation of podocyte-specific proteins, including nephrin, wilms tumor type 1 (WT1), and podocalyxin, as well as cytoskeletal protein zonula occludens 1 (ZO-1) (Wang et al., 2011; Ying and Wu, 2017). Increased levels of inflammation are also regarded as an initial hallmark of DKD and play a key role in podocyte injury (Reidy et al., 2014).

The NOD-like receptor pyrin domain-containing protein 3 (NLRP3) inflammasome is the most well-studied inflammasome and has been reported to recruit the adaptor protein apoptosis-associated speck-like protein containing a C-terminal caspase recruitment domain (ASC) to activate cysteinyl aspartate-specific proteinase (caspase)-1, thus leading to the secretion of mature interleukin-1β (IL-1β) and interleukin-18 (IL-18) (Mulay, 2019). A previous study showed that the highly conserved serine or threonine kinase NIMA-related kinase 7 (NEK7) is an important requirement in NLRP3 activation *via* direct interaction with NLRP3-NEK7 (Sharif et al., 2019). Recent research has demonstrated that the NLRP3 inflammasome plays a vital role in podocyte injury under high glucose (HG) conditions (Hou et al., 2020). Pyroptosis is a newly discovered lytic form of programmed cell death that has been shown to play a critical role in DKD (Lin et al., 2020). Pyroptosis involves cell swelling, rupture, the secretion of cell contents, and dramatic proinflammatory effects. The NLRP3 inflammasome is known to be the most important initiator of pyroptosis. Pyroptosis is initiated by the canonical inflammasome pathway in which NLRP3 mediates the cleavage of gasdermin D (GSDMD) and the activation of IL-1β and IL-18, thus leading to the induction of caspase-1 (Yu et al., 2020). As a downstream effector of inflammasomes, the hydrolyzed form of GSDMD released N-terminus of GSDMD (GSDMD-N). The polymerization of GSDMD-N forms pores; the subsequent exchange of extracellular and intracellular substances ultimately results in pyroptosis (De Vasconcelos and Lamkanfi, 2020). Cheng et al. previously reported that GSDMD-mediated pyroptosis is activated and plays a role in the loss of podocytes in a mouse model of diabetic nephropathy (Cheng et al., 2020). Thus, controlling the activation of the NLRP3 inflammasome and podocyte pyroptosis play important roles in the progression of DKD.

The phosphatidylinositol 3-kinases (PI3K) have been linked to an extraordinarily diverse group of cellular functions *via* the activation of protein kinase B, also known as Akt. These cellular functions include cell growth, proliferation, differentiation, motility, and survival (Jafari et al., 2019). Regulation of the PI3K/Akt signaling pathway has been increasingly implicated in protecting podocytes in DKD (Song et al., 2014; Wang et al., 2014; Yang et al., 2020). In addition, phosphate and tension homology (PTEN) is a form of 3,4,5-triphosphate inositol lipase that negatively regulates the PI3K signaling pathway (Maidarti et al., 2020). Xing et al. previously proved that the down-regulation of PTEN activity led to sustained activation of the PI3K/Akt signaling pathway and ultimately induced the phenotypic transition of podocytes under HG conditions (Xing et al., 2015). Therefore, regulation of the PTEN/PI3K/Akt pathway in podocyte injury is considered as an effective strategy for the treatment of DKD.

The methylation of adenosine at the N6 position leads to the formation of N6-methyladenosine (m^6^A); this is the most abundant and reversible methylation modification. Furthermore, m^6^A modification can be carried out by methyltransferases, including methyltransferase-like (METTL)3, METTL14, and Wilms tumor 1-associated protein (WTAP) (Tong et al., 2018). PTEN is one of the host RNAs for m^6^A modification. A recent study reported that METTL3 rescued cell viability by targeting the PTEN/Akt signaling cascade in HG-treated retinal pigment epithelium (RPE) cells (Zha et al., 2020). However, METTL14, but not METTL3, has been shown to regulate the PI3K/Akt signaling pathway via PTEN in the HG-induced epithelial-mesenchymal transition (EMT) of renal tubular cells (Xu et al., 2021). Consequently, m^6^A-modified PTEN may regulate the PI3K/Akt signaling pathway in DKD; however, the mechanisms underlying this process are likely to be complex and have yet to be elucidated.

The total flavones of *Abelmoschus manihot* (TFA) are the main components of Huangkui capsule (HKC; the local name in China); this is a preparation of a modern Chinese patented medicine extracted from *Abelmoschus manihot* (AM). This preparation has been approved by the China State Food and Drug Administration (Z19990040) for the treatment of kidney disease and has been used over the past 2 decades (Zhang et al., 2014; Li et al., 2021). Our previous studies proved that following drug-intervention, HKC can safely and efficiently alleviate the early pathological changes in the glomeruli in the kidneys by inhibiting the PI3K/Akt signaling activity, both in a rat model of DKD induced by unilateral nephrectomy combined with the intraperitoneal injection of streptozotocin (STZ), and in murine mesangial cells under HG conditions (Wu et al., 2018). In addition, HKC has been shown to alleviate renal tubular injury by inhibiting NLRP3 inflammasome activation in the rat model of DKD (Han et al., 2019). Despite these findings, there are still some important but unresolved issues with regards to the precise role of podocytes in DKD when treated by TFA. For example, it has not yet been ascertained whether TFA can improve podocyte pyroptosis and injury by inhibiting the activation of the NLRP3 inflammasome and by regulating the PTEN/PI3K/Akt signaling pathway and m^6^A modification. Furthermore, the therapeutic mechanisms associated with such processes have yet to be determined.

In the present study, we designed a series of cell experiments to verify our hypothesis that TFA may alleviate podocyte pyroptosis and injury by targeting m^6^A modification-mediated NLRP3-inflammasome activation and PTEN/PI3K/Akt signaling.

## Materials and Methods

### Reagents and Drugs

Dulbecco’s modified eagle medium (DMEM) was obtained from HyClone, Co., Ltd. (UT, United States). Fetal bovine serum (FBS) was obtained from Gibco, Co., Ltd. (Grand Island, NY). Recombinant γ-interferon (IFN-γ) was obtained from Peprotech Co., Ltd. (London, United Kingdom). TFA was obtained from Suzhong Pharmaceutical Group Co., Ltd. (Taizhou, China). TFA was solubilized in distilled water to a final concentration of 1 g/L and stored at 4°C prior to use. MCC950 and 740Y-P were obtained from MedChemexpress Co., Ltd. (Shanghai, China) and solubilized in 10% dimethyl sulfoxide (DMSO) at a final concentration of 1 mM.

### Cell Culture and Treatment

An immortalized mouse podocyte cell-5 line (MPC-5 cells) was provided by Professor Jian Yao (Division of Molecular Signaling, Department of Advanced Biomedical Research, Interdisciplinary Graduate School of Medicine and Engineering, University of Yamanashi, Yamanashi, Japan). The MPC-5 cells were cultured at a permissive temperature (33°C) in 5% CO_2_ in DMEM containing 10% FBS and recombinant IFN-γ (10 U/mL). Following passage, MPC-5 cells were cultured at 37°C in 5% CO_2_ for 14 days in DMEM medium without IFN-γ to induce differentiation. Fully differentiated MPC-5 cells were then cultured in 5.6 and 30 mM glucose concentrations to induce cellular injury, and with or without MCC950 (10 µM) or 740Y-P (30 μM) for 1 h, prior to investigating the effect of TFA on podocyte injury.

### Cell Viability Assessment

Cell viability was determined by CCK-8 assays (Biosharp, Shanghai, China). The cells were seeded into 96-well plates at a density of 0.5 × 10^4^ cells per well in DMEM containing 0.1% FBS; three replicates were carried out for each experimental group. When the confluency was 70–80%, we added a high glucose (HG) concentration with or without TFA or MCC950 treatment for 24 h. The CCK-8 solution (10 μl) was added to each well and incubated for 2 h. The optical density (OD) was determined at an absorbance of 450 nm and cell viability was calculated.

### The Overexpression and Knockdown of METTL3 in MPC-5 Cells

cDNA fragment of METTL3 were amplified and ligated into the pcDNA3.1 vector to construct an overexpression (OE) vector for METTL3 (METTL3 OE); the vector was synthesized by Beijing Syngentech Co., Ltd. (Beijing, China). The small interfering RNA (siRNA) for METTL3 knockdown (METTL3 KD) was also purchased from Beijing Syngentech Co., Ltd. (Beijing, China). A Lipofectamine 3000 transfection kit, purchased from Thermo Fisher Scientific Co., Ltd. (Waltham, United States) was then used to transfect MPC-5 cells with the vector or siRNA; this kit was used in accordance with the manufacturer’s instructions.

### Western Blot Analysis

Protein expression levels were detected by western blotting (WB). MPC-5 cells were lysed with a total protein extraction kit (KeyGEN, China) and protein concentration was determined by a bicinchoninic acid kit in accordance with the manufacturer’s instruction. Protein loading buffer was added into each sample at a ratio of 4:1 and mixed. Samples were then denatured by boiling at 100°C for 8 min. Each group of proteins was subjected to sodium dodecyl sulfate-polyacrylamide gel electrophoresis (SDS-PAGE). We used a range of primary antibodies, including GSDMD-N (Abcam, Cambridge, MA; 1:1,000), IL-1β (Abcam, Cambridge, MA; 1:1,000), IL-18 (Abcam, Cambridge, MA; 1:1,000), nephrin (Abcam, Cambridge, MA; 1:500), ZO-1 (Absin, Shanghai, China; 1:500), WT1 (Absin, Shanghai, China; 1:500), podocalyxin (Absin, Shanghai, China; 1:500), caspase-1 (Abcam, Cambridge, MA; 1:500), ASC (Absin, Shanghai, China; 1:1,000), NLRP3 (Abcam, Cambridge, MA; 1:1,000), phosphorylated-PI3K (p-PI3K) (Cell Signaling, Beverly, MA; 1:1,000), PI3K (Cell Signaling, Beverly, MA; 1:1,000), phosphorylated-Akt (p-Akt) (Cell Signaling, Beverly, MA; 1:1,000), Akt (Cell Signaling, Beverly, MA; 1:1,000), PTEN (Cell Signaling, Beverly, MA; 1:1,000), NEK7 (Abcam, Cambridge, MA; 1:1,000), METTL3 (Cell Signaling, Beverly, MA; 1:1,000), METTL14 (Cell Signaling, Beverly, MA; 1:1,000), WTAP (Cell Signaling, Beverly, MA; 1:1,000), and GAPDH (Cell Signaling, Beverly, MA; 1:1,000). Membranes were incubated at 4°C overnight and then incubated with goat anti-rabbit IgG horseradish peroxidase-conjugated secondary antibody for 1 h at room temperature. The blots were finally visualized using an enhanced chemiluminescence detection system (Tanon-5200Muilti, China). Quantitative analysis was performed using ImageJ Software (NIH, United States; http://rsbweb.nih.gov/ij/index.html).

### Calcein-AM/PI Double Staining

A calcein-AM/PI double staining kit was purchased from Dojindo Laboratories (Tokyo, Japan). First, a calcein-AM/PI working solution was prepared using 2 μM calcein-AM, and 4.5 μM PI. After intervention, the cells of each group were washed with PBS once, then stained with 100 μl calcein-AM/PI working solution and incubated at 37°C for 15 min. After incubation, the cells were washed twice. The cells were assessed under a fluorescence microscope (200×) or detected by flow cytometry to determine the percentage of dead/live cells.

### Quantitative Real-Time PCR

Total RNA was extracted using TRIzol (Invitrogen, Thermo Fisher Scientific, Waltham, MA, United States). Complementary DNA (cDNA) was synthesized and processed by quantitative real-time polymerase chain reaction (qRT-PCR) analysis using a QuantiNova SYBR Green PCR Kit (QIAGEN, Hilden, Germany). All experiments were performed according to the manufacturer’s instructions. qRT-PCR analysis was performed using an ABI Step One Plus Real-Time PCR instrument (Applied Biosystems, Inc., Thermo Fisher Scientific, Foster City, CA, United States). The primer pair sequences were as follows: PTEN Forward: 5′-TGG​CGG​AAC​TTG​CAA​TCC​TCA​GT-3′, Reverse: 5′-TCC​CGT​CGT​GTG​GGT​CCT​GA-3′; GAPDH, forward: 5′-AAC​AGC​CTC​AAG​ATC​ATC​AGC​A-3′, reverse: 5′-ATG​AGT​CCT​TCC​ACG​ATA​CCA-3′. The relative expression levels of PTEN mRNA were determined by the Ct (2^−ΔΔCt^) method (Chen et al., 2016). GAPDH was used for normalization.

### The Measurement of m^6^A Content

The total m^6^A content of MPC-5 cells was determined using an EpiQuik™ m^6^A RNA methylation quantification kit (Epigentek Group Inc. United States); the kit was used in accordance with the manufacturer’s instructions. The total content of m^6^A was calculated using the following formula: m^6^A % = {[(Sample OD − NC OD)/S] ÷ [(PC OD − NC OD)/P]} × 100%, and OD, NC, S, PC, and P represent the optical density, negative controls, total amount of input RNA, positive controls, and total amount of positive control RNA, respectively.

### Methylated RNA Immunoprecipitation With qPCR (MeRIP-qPCR)

Next, we performed m^6^A immunoprecipitation to measure the m^6^A modification of PTEN. In brief, a m^6^A antibody and normal rabbit IgG were conjugated to protein A/G magnetic beads (Bio-linkedin, China) at 4°C overnight. RNA was then fragmented and incubated with magnetic beads in immunoprecipitation buffer supplemented with an RNase inhibitor at 4°C for 3 h. Following two washes with IP buffer, we used elution buffer to elute the mRNA from the beads. Following extraction and precipitation, the input RNA and eluted RNA were reverse transcribed. Their abundance was then determined by RT-PCR.

### Statistical Analysis

All the data were collected and presented as means ± standard deviation (SD). Data were analyzed by SPSS 24.0 (IBM, Armonk, NY, United States). Differences between multiple groups were compared by one-way analysis of variance (ANOVA) on normally distributed data with the least significant difference (LSD) post hoc test, or non-parametric Kruskal-Wallis if not. A *p* value < 0.05 or < 0.01 indicated a statistically significant difference.

## Results

### Total flavones of *Abelmoschus manihot* Ameliorated Pyroptosis and Injury in Podocytes Induced by High Glucose

**FIGURE 1:**
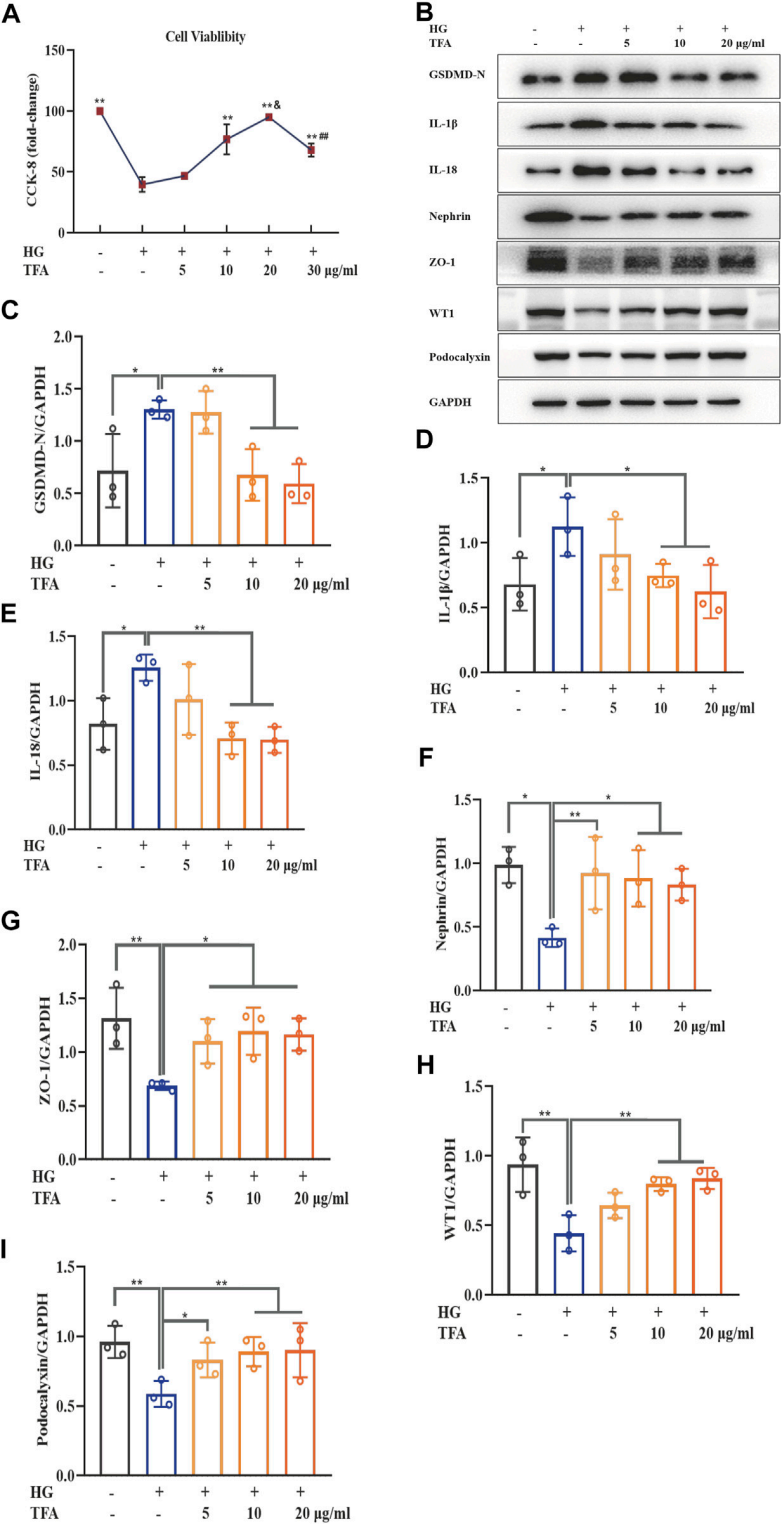
TFA ameliorated podocyte pyroptosis and injury induced by HG in MPC-5 cells in a dose-dependent manner. **(A)** The cell viability in cultured MPC-5 cells exposed to HG at 30 mM for 48 h with or without TFA at 5, 10, 20, and 30 μg/ml for 24 h; **(B)** WB analysis of GSDMD-N, IL-1β, IL-18, nephrin, ZO-1, WT1 and podocalyxin in cultured MPC-5 cells exposed to HG at 30 mM for 48 h with or without TFA at 5, 10, and 20 μg/ml for 24 h; **(C)** GSDMD-N was quantified by densitometry; **(D)** IL-1β was quantified by densitometry; **(E)** IL-18 was quantified by densitometry; **(F)** Nephrin was quantified by densitometry; **(G)** ZO-1 was quantified by densitometry; **(H)** WT1 was quantified by densitometry; **(I)** Podocalyxin was quantified by densitometry. Data are expressed as mean ± S.D., (*n* = 3). *p < 0.05, **p < 0.01 *vs.* the HG group; ^&^ *p* < 0.05 *vs*. the TFA (10 μg/ml) group; ^##^ *p* < 0.01 *vs*. the TFA (20 μg/ml) group. Abbreviations: TFA, total flavones of *Abelmoschus manihot*; HG, high glucose; MPC-5, mouse podocyte cell-5; WB, western blotting; GSDMD-N, N-terminus of GSDMD; IL-1β, interleukin-1β; IL-18, interleukin-18; ZO-1, Zonula occludens 1; WT1, Wilms tumor type 1.

To identify the protective effects of TFA in podocytes, we investigated the effects of TFA on pyroptosis and injury in podocytes induced by HG. First, we used the CCK-8 assay to investigate cellular viability. As shown in Figure 1A, compared to control cells, the viability of podocytes exposed to HG for 48 h was remarkably reduced to 45%. However, this reduction in viability was significantly reversed in podocytes that were co-treated with TFA for 24 h. This effect occurred in a dose-dependent manner and the protective effects towards podocytes were most significant at a TFA concentration of 20 μg/ml. Next, we used WB analysis to detect the expression levels of several proteins related to pyroptosis (GSDMD-N, IL-1β, and IL-18) as well as the levels of proteins known to protect podocytes (nephrin, ZO-1, WT1 and podocalyxin). Our analysis showed that HG conditions increased the protein levels of GSDMD-N, IL-1β, and IL-18, but reduced the protein levels of nephrin, ZO-1, WT1 and podocalyxin, when compared to those of the control cells. Conversely, the protein expression levels of GSDMD-N, IL-1β, IL-18, nephrin, ZO-1, WT1 and podocalyxin were rescued following co-treatment with TFA for 24 h; these effects occurred in a dose-dependent manner (Figures 1B–I).

**FIGURE 2:**
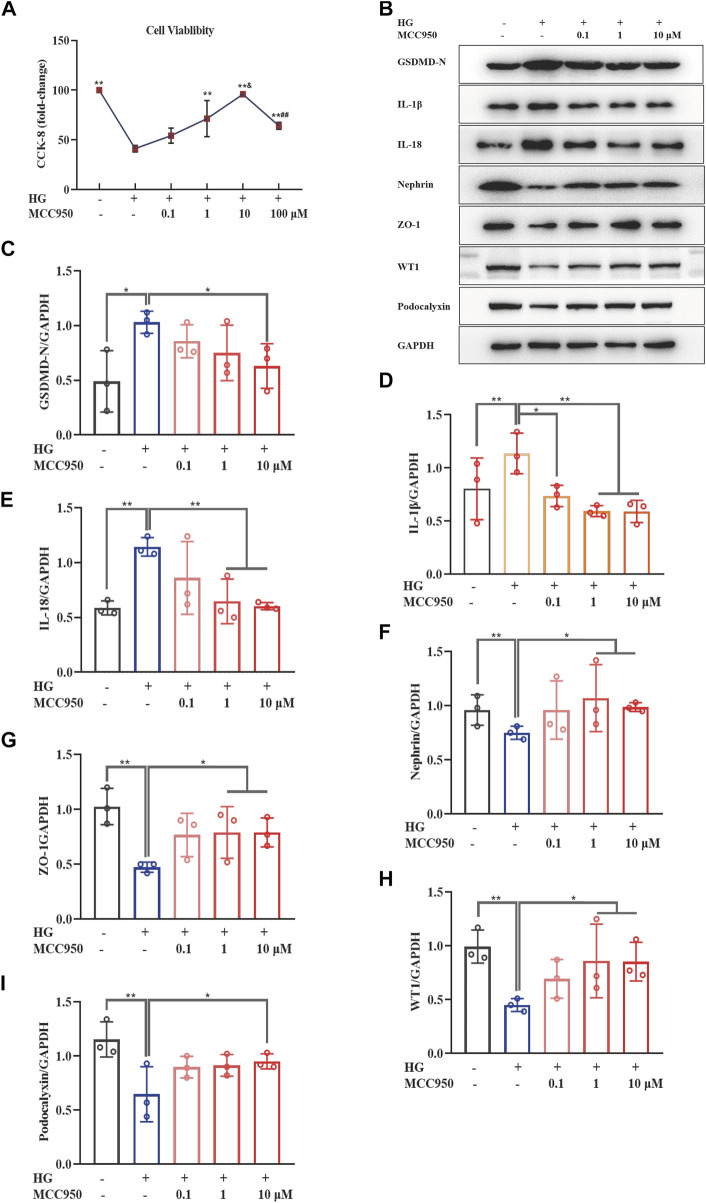
MCC950 ameliorated podocyte pyroptosis and injury induced by HG in a dose-dependent manner in MPC-5 cells. **(A)** The cell viability in cultured MPC-5 cells exposed to HG at 30 mM for 48 h with or without MCC950 at 0.1, 1, 10, and 100 μM for 24 h; **(B)** WB analysis of GSDMD-N, IL-1β, IL-18, nephrin, ZO-1, WT1 and podocalyxin in cultured MPC-5 cells exposed to HG at 30 mM for 48 h with or without MCC950 at 0.1, 1, and 10 μM for 24 h; **(C)** GSDMD-N was quantified by densitometry; **(D)** IL-1β was quantified by densitometry; **(E)** IL-18 was quantified by densitometry; **(F)** Nephrin was quantified by densitometry; **(G)** ZO-1 was quantified by densitometry; **(H)** WT1 was quantified by densitometry; **(I)** Podocalyxin was quantified by densitometry. Data are expressed as mean ± S.D., (*n* = 3). *p < 0.05, **p < 0.01 *vs.* the HG group; ^&^ *p* < 0.05 *vs.* the TFA (10 μg/ml) group; ^##^ *p* < 0.01 *vs.* the TFA (20 μg/ml) group. Abbreviations: HG, high glucose; MPC-5, mouse podocyte cell-5; WB, western blotting; GSDMD-N, N-terminus of GSDMD; IL-1β, interleukin-1β; IL-18, interleukin-18; ZO-1, Zonula occludens 1; WT1, Wilms tumor type 1.

To further confirm the specific role of TFA in the alleviation of pyroptosis and injury in podocytes, we carried out a series of experiments using MCC950 (an inhibitor of NLRP3). As shown in Figure 2A, the CCK-8 assay showed that MCC950 led to the restoration of cellular viability at a concentration of 10 μM under HG conditions. Next, we investigated the effects of MCC950 on HG-stimulated pyroptosis and injury in podocytes. Our results revealed that MCC950 did not only down-regulate the protein expression levels of GSDMD-N, IL-1β, and IL-18; it also upregulated the protein expression levels of nephrin, ZO-1, WT1 and podocalyxin in podocytes under HG conditions; these effects occurred in a dose-dependent manner (Figures 2B–I).

These results demonstrated that TFA and MCC950 could protect podocytes against HG-induced pyroptosis and injury and that this effect may be related to the inhibition of NLRP3 inflammasome activation.

### Total flavones of *Abelmoschus manihot* Protected Podocytes by Inhibiting Activation of the NLRP3 Inflammasome via the PI3K/Akt Signaling Pathway

**FIGURE 3:**
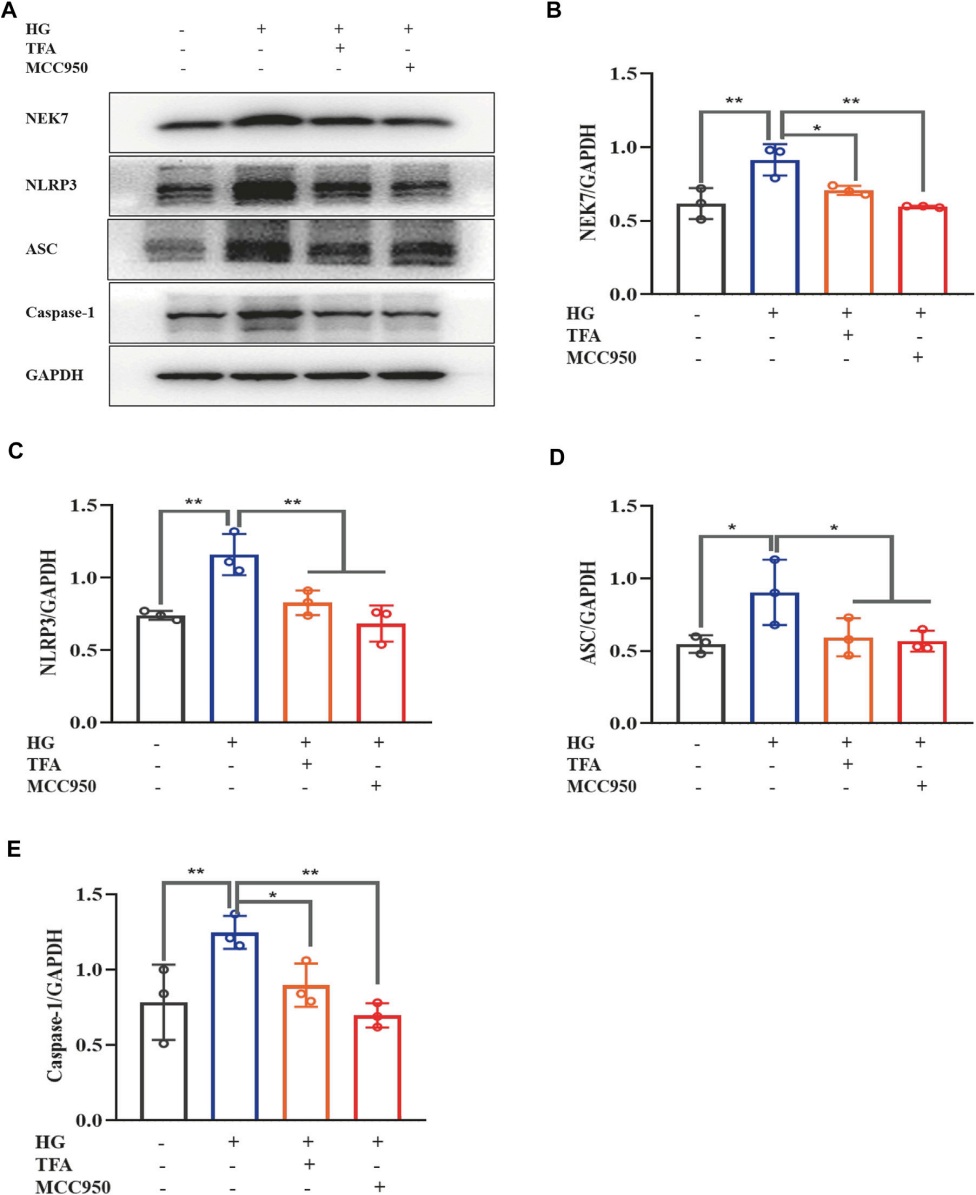
TFA and MCC950 attenuated HG-induced inflammasome activation in MPC-5 cells. **(A)** WB analysis of NEK7, NLRP3, ASC, and caspase-1, in cultured MPC-5 cells exposed to HG at 30 mM for 48 h with or without TFA (20 μg/ml) and MCC950 (10 μM) treatment for 24 h; **(B)** NEK7 was quantified by densitometry; **(C)** NLRP3 was quantified by densitometry; **(D)** ASC was quantified by densitometry; **(E)** Caspase-1 was quantified by densitometry. Data are expressed as mean ± S.D., (*n* = 3). *p < 0.05, **p < 0.01. Abbreviations: TFA, total flavones of *Abelmoschus manihot*; HG, high glucose; MPC-5, mouse podocyte cell-5; WB, western blotting; NEK7, NIMA-related kinase 7; NLRP3, NOD-like receptor pyrin domain-containing protein 3; ASC, a C-terminal caspase recruitment domain; Caspases-1, cysteinyl aspartate-specific proteinase-1.

Recent studies have shown that the activation of the NLRP3 inflammasome is a trigger of pyroptosis, and that NEK7 acts as a kinase regulator to activate the NLRP3 inflammasome. To further explore the regulatory effect of TFA on the activation of inflammasomes, we used WB to detect the protein expression levels of NEK7, NLRP3, ASC, and caspase-1 under HG conditions (Figure 3A). We found that the expression levels of all proteins were significantly elevated in the HG group when compared to levels in the control cells. When treated with TFA (20 μg/ml) or MCC950 (10 μM) for 24 h, the protein expression levels of NEK7, NLRP3, ASC, and caspase-1, all improved in the cultured podocytes exposed to HG conditions (Figures 3B–E).

**FIGURE 4:**
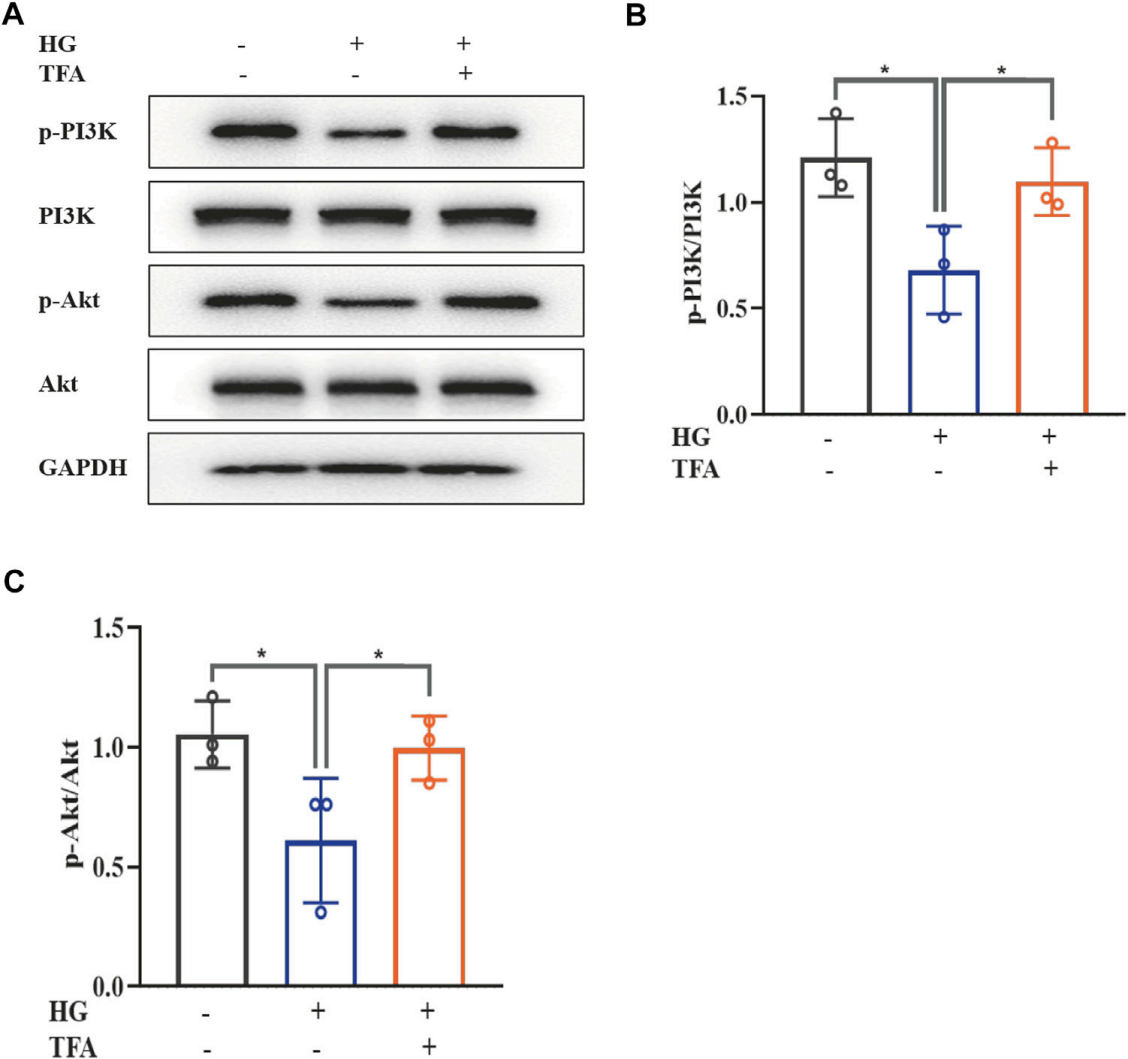
TFA activated the PI3K/Akt signaling pathway in HG-afflicted MPC-5 cells. **(A)** WB analysis of p-PI3K, PI3K, p-Akt, and Akt, in cultured MPC-5 cells exposed to HG at 30 mM for 48 h with or without TFA (20 μg/ml) treatment for 24 h; **(B)** p-PI3K was quantified by densitometry; **(C)** p-Akt was quantified by densitometry. Data are expressed as mean ± S.D., (*n* = 3). *p < 0.05, **p < 0.01. Abbreviations: TFA, total flavones of *Abelmoschus manihot*; PI3K, phosphatidylinositol 3-kinases; Akt, protein kinase B; HG, high glucose; MPC-5, mouse podocyte cell-5; WB, western blotting; p-PI3K, phosphorylated-phosphatidylinositol 3-kinase; p-Akt, phosphorylated-protein kinase B.

The PI3K/Akt signaling pathway has been found to be closely involved with the progression of pyroptosis in podocytes and represents the downstream target for inflammasome activation in HG conditions. The main signaling molecules of the PI3K/Akt pathway include p-PI3K and p-Akt. Therefore, we determined the effect of TFA on the protein expression levels of p-PI3K and p-Akt in HG-stimulated podocytes by WB analysis. As shown in Figure 4, HG inhibited the phosphorylation levels of PI3K and Akt when compared to those of the control cells. Furthermore, co-treatment with TFA (20 μg/ml) for 24 h significantly improved the protein expression levels of p-PI3K and p-Akt (Figures 4A–C).

**FIGURE 5:**
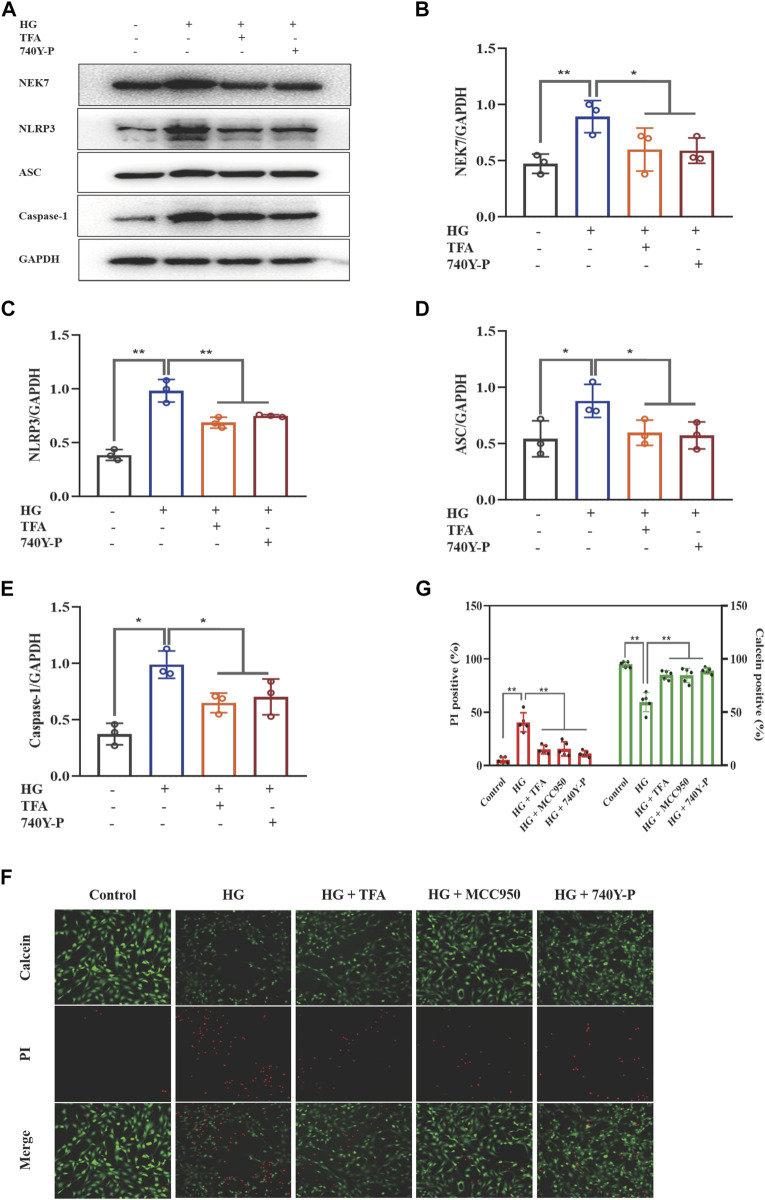
TFA and 740Y-P inhibited HG-induced inflammasome activation in MPC-5 cells. **(A)** WB analysis of NEK7, NLRP3, ASC, and caspase-1, in cultured MPC-5 cells exposed to HG at 30 mM for 48 h with or without TFA (20 μg/ml) and 740Y-P (30 μM) treatment for 24 h; **(B)** NEK7 was quantified by densitometry; **(C)** NLRP3 was quantified by densitometry; **(D)** ASC was quantified by densitometry; **(E)** Caspase-1 was quantified by densitometry; **(F)** Representative pictures of calcein-AM/PI double stains. The live cells are stained by calcein and the death cells are stained by PI. Four fields (200×) were randomly selected to calculate the percentage of alive/dead cells. In this part of the experiment, MPC-5 cells exposed to HG at 30 mM for 48 h with or without TFA (20 μg/ml), MCC950 (10 μM) and 740Y-P (30 μM) treatment for 24 h **(G)**The ratio of positive cells was calculated according to the calcein-AM/PI double stains. Data are expressed as mean ± S.D., (*n* = 3 or *n* = 4). *p < 0.05, **p < 0.01. Abbreviations: TFA, total flavones of *Abelmoschus manihot*; HG, high glucose; MPC-5, mouse podocyte cell-5; WB, western blotting; NEK7, NIMA-related kinase 7; NLRP3, NOD-like receptor pyrin domain-containing protein 3; ASC, a C-terminal caspase recruitment domain; Caspases-1, cysteinyl aspartate-specific proteinase-1.

**FIGURE 6:**
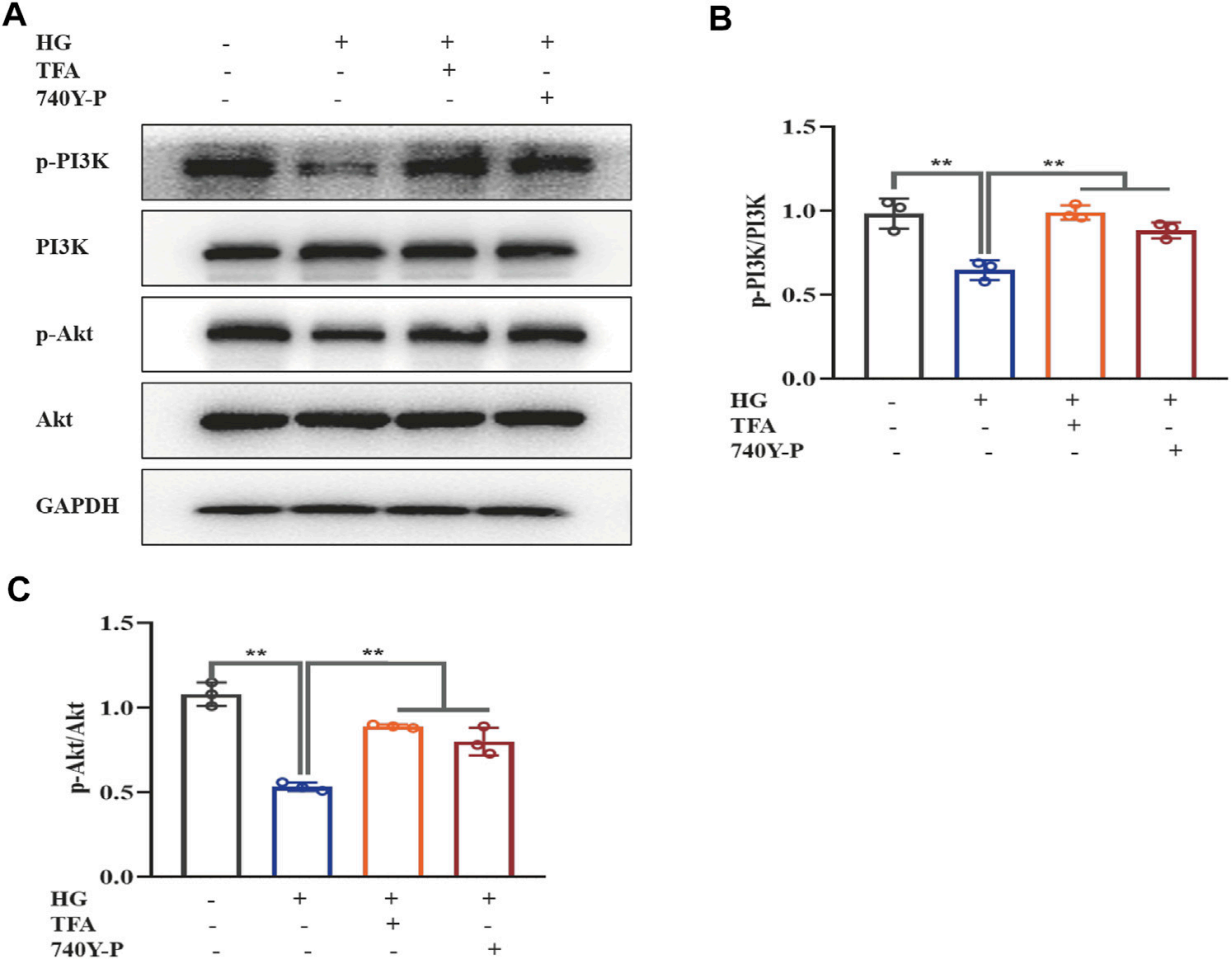
TFA and 740Y-P activated the PI3K/Akt signaling pathway in HG-afflicted MPC-5 cells. **(A)** WB analysis of p-PI3K, PI3K, p-Akt, and Akt, in cultured MPC-5 cells exposed to HG at 30 mM for 48 h with or without TFA (20 μg/ml) and 740Y-P (30 μM) treatment for 24 h; **(B)** p-PI3K was quantified by densitometry; **(C)** p-Akt was quantified by densitometry. Data are expressed as mean ± S.D., (*n* = 3). *p < 0.05, **p < 0.01. Abbreviations: TFA, total flavones of *Abelmoschus manihot*; PI3K, phosphatidylinositol 3-kinases; Akt, protein kinase B; HG, high glucose; MPC-5, mouse podocyte cell-5; WB, western blotting; p-PI3K, phosphorylated-phosphatidylinositol 3-kinase; p-Akt, phosphorylated-protein kinase B.

To further determine the regulatory effect of TFA on the activation of the PI3K/Akt pathway and the downstream NLRP3 inflammasomes, we used an exogenous agonist of PI3K (740Y-P) as a control. The levels of pyroptosis-related proteins were then detected by WB. Similar to the previous results, TFA and 740Y-P were found to suppress the protein expression levels of NEK7, NLRP3, ASC, and caspase-1 (Figures 5A–E) and increase the protein expression levels of p-PI3K and p-Akt in cultured podocytes exposed to HG conditions (Figures 6A–C). Calcein-AM/PI double staining was performed to identify live/dead cells. With HG stimulation for 48 h, the percentage of calcein-positive cells significantly decreased, whereas that of PI-positive cells significantly increased. In HG condition, with the co-treatment of TFA, MCC950 and 740Y-P the percentage of calcein-positive cells were increased and the PI-positive cells were decreased (Figures 5F,G). In addition, the mimic changes could be found in the levels of GSDMD, NLRP3, IL-1β, IL-18, nephrin, ZO-1 and WT-1 in HG-stimulated podocytes with or without TFA (20 μg/ml), MCC950 (10 μM) and 740Y-P (30 μM) detecting by ELISA or qRT-PCR (Supplementary Figures S1, S2).

Collectively, these data suggested that TFA and 740Y-P protected podocytes from HG-induced pyroptosis and injury by inhibiting the activation of the NLRP3 inflammasome via the PI3K/Akt signaling pathway.

### Total flavones of *Abelmoschus manihot* Inhibited the PTEN/PI3K/Akt Signaling by Attenuating m^6^A Modification

**FIGURE 7:**
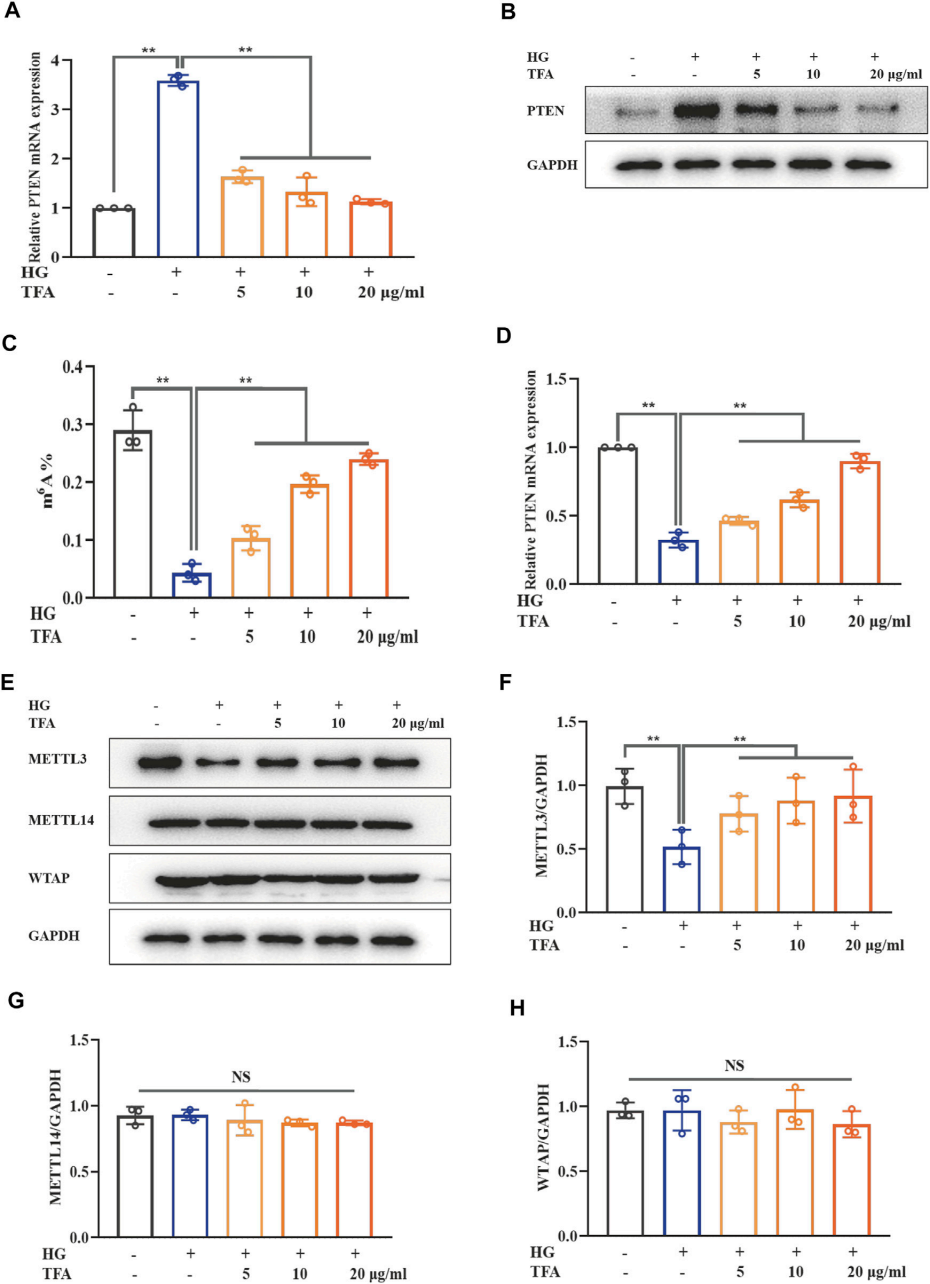
TFA inhibited PTEN/PI3K/Akt signaling by increasing the m^6^A modification of PTEN mRNA in HG-afflicted MPC-5 cells. **(A)** The mRNA levels of PTEN in cultured MPC-5 cells exposed to HG at 30 mM for 48 h with or without TFA at 5, 10, and 20 μg/ml for 24 h by qRT-PCR; **(B)** WB analysis of PTEN in cultured MPC-5 cells exposed to HG at 30 mM for 48 h with or without TFA at 5, 10, and 20 μg/ml for 24 h; **(C)** Quantification of m^6^A levels in cultured MPC-5 cells exposed to HG at 30 mM for 48 h with or without TFA at 5, 10, and 20 μg/ml for 24 h by the m^6^A RNA methylation quantification kit; **(D)** The levels of methylated PTEN mRNA in the cultured MPC-5 cells exposed to HG at 30 mM for 48 h with or without TFA at 5, 10, and 20 μg/ml for 24 h by a Me-RIP assay; **(E)** WB analysis of METTL3, METTL14, and WTAP, in cultured MPC-5 cells exposed to HG at 30 mM for 48 h with or without TFA at 5, 10, and 20 μg/ml for 24 h; **(F)** METTL3 was quantified by densitometry; **(G)** METTL14 was quantified by densitometry; **(H)** WTAP was quantified by densitometry. Data are expressed as mean ± S.D., (*n* = 3). *p < 0.05, **p < 0.01. Abbreviations: TFA, total flavones of *Abelmoschus manihot*; PTEN, phosphate and tension homology; PI3K, phosphatidylinositol 3-kinases; Akt, protein kinase B; m^6^A, N6-methyladenosine; HG, high glucose; MPC-5, mouse podocyte cell-5; qRT-PCR, quantitative real-time polymerase chain reaction; WB, western blotting; MeRIP, methylated RNA immunoprecipitation; METTL, methyltransferase-like; WTAP, Wilms tumor 1-associated protein; NS, not significant.

The PTEN signaling pathway is the upstream target of the PI3K/Akt pathway. Our results showed that both mRNA and protein expression levels of PTEN were elevated in HG-treated podocytes and that TFA could reduce the levels of PTEN (Figures 7A,B). m^6^A modification includes a range of mRNA transcriptional modifications and has been found to be a dynamic regulator of various biological processes. Next, we investigated the levels of intracellular m^6^A modification in podocytes and found that the levels of global m^6^A modification were remarkably reduced under HG conditions; these were effectively rescued by co-treatment of TFA (Figure 7C). In order to confirm the relationship between PTEN transcription and m^6^A modification, we investigated the methylation levels of PTEN mRNA by Me-RIP assay. Figure 7D shows that the levels of methylated PTEN mRNA were reduced in HG conditions when compared to those of the control cells. However, this level of suppression was ameliorated by the co-treatment of podocytes with TFA.

It is known that the methylation modification of m^6^A needs to be completed under the catalysis of a m^6^A methyltransferase complex containing METTL3, METTL14, and WTAP. Next, we observed the protein expression levels of these key m^6^A methyltransferase members in HG-stimulated podocytes (Figure 7E). The expression levels of METTL3 protein were significantly down-regulated in HG-stimulated podocytes when compared to those of the control cells. In comparison with the stimulation of HG, TFA could up-regulate the activation of METTL3 methyltransferase in a dose-dependent manner (Figure 7F). However, the expression levels of METTL14 and WTAP in HG-treated podocytes did not change remarkably (Figures 7G,H).

Collectively, these data showed that TFA restored activation of the PTEN/PI3K/Akt signaling pathway induced by HG conditions by regulating the m^6^A methylated modification catalyzed by METTL3.

### Total Flavones of *Abelmoschus manihot* Ameliorated Pyroptosis and Injury in Podocytes in a METTL3-Dependent Manner by Regulating the Activation of the NLRP3 Inflammasome and PTEN/PI3K/Akt Signaling Pathway

**FIGURE 8:**
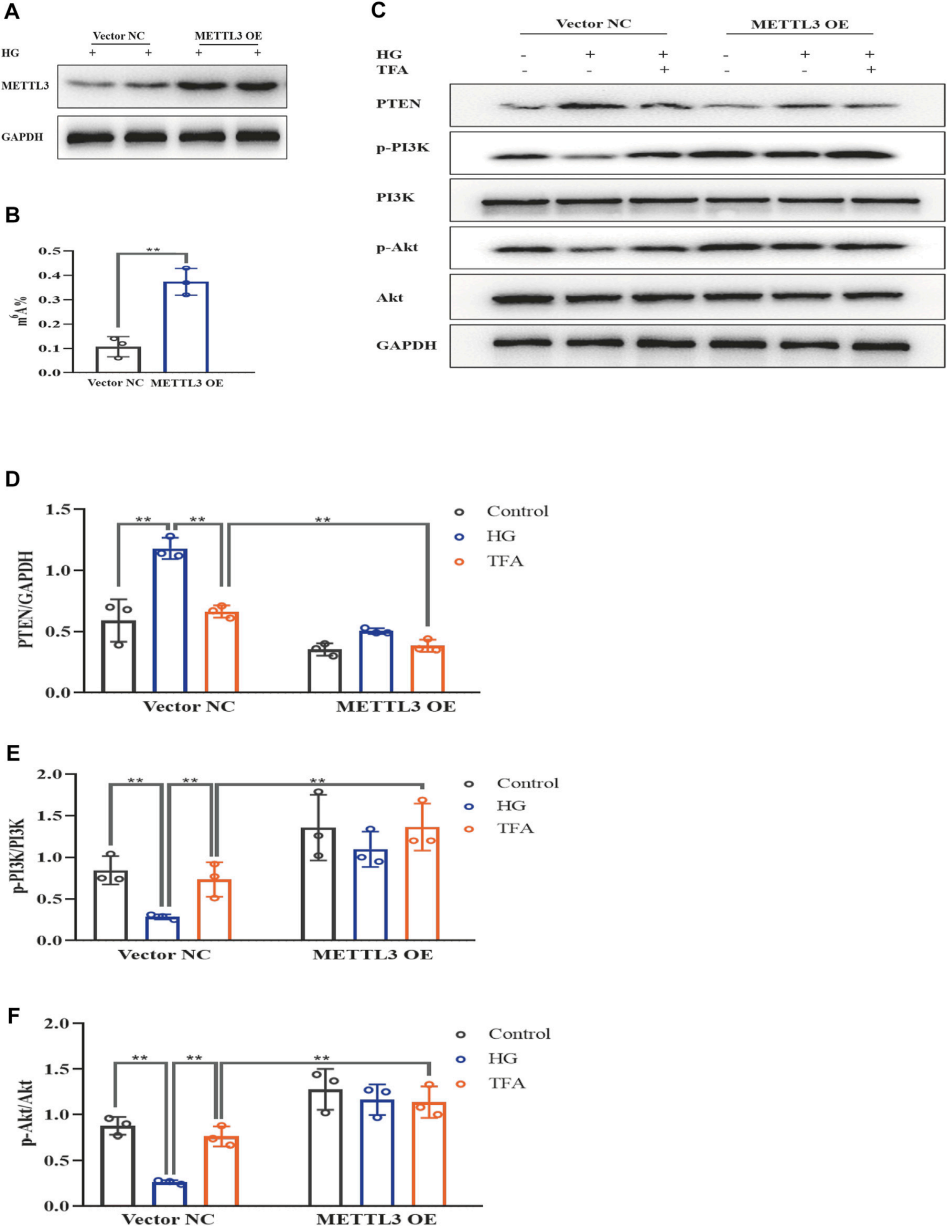
TFA regulated the PTEN/PI3K/Akt signaling pathway in a METTL3-dependent manner *via* m^6^A modification in HG-afflicted MPC-5 cells with overexpressed METTL3. **(A)** WB analysis of METTL3 in cultured MPC-5 cells transfected in duplicates with a NC vector or a METTL3 OE vector for 24 h and exposed to HG (30 mM) for 48 h; **(B)** Quantification of m^6^A levels in cultured MPC-5 cells transfected in duplicates with a NC vector or METTL3 OE vector for 24 h and exposed to HG (30 mM) for 48 h, as determined by a m^6^A RNA methylation quantification kit; **(C)** WB analysis of PTEN, p-PI3K, PI3K, p-Akt, and Akt, in cultured MPC-5 cells transfected with NC or METTL3 OE vectors for 24 h, following by exposure to HG at 30 mM for 48 h with or without TFA (20 μg/ml) for 24 h; **(D)** PTEN was quantified by densitometry; **(E)** p-PI3K was quantified by densitometry; **(F)** p-Akt was quantified by densitometry. Data are expressed as mean ± S.D., (*n* = 3). *p < 0.05, **p < 0.01. Abbreviations: TFA, total flavones of *Abelmoschus manihot*; PTEN, phosphate and tension homology; PI3K, phosphatidylinositol 3-kinases; Akt, protein kinase B; METTL, methyltransferase-like; m^6^A, N6-methyladenosine; HG, high glucose; MPC-5, mouse podocyte cell-5; WB, western blotting; NC, negative control; OE, overexpression; p-PI3K, phosphorylated-phosphatidylinositol 3-kinase; p-Akt, phosphorylated-protein kinase B.

**FIGURE 9:**
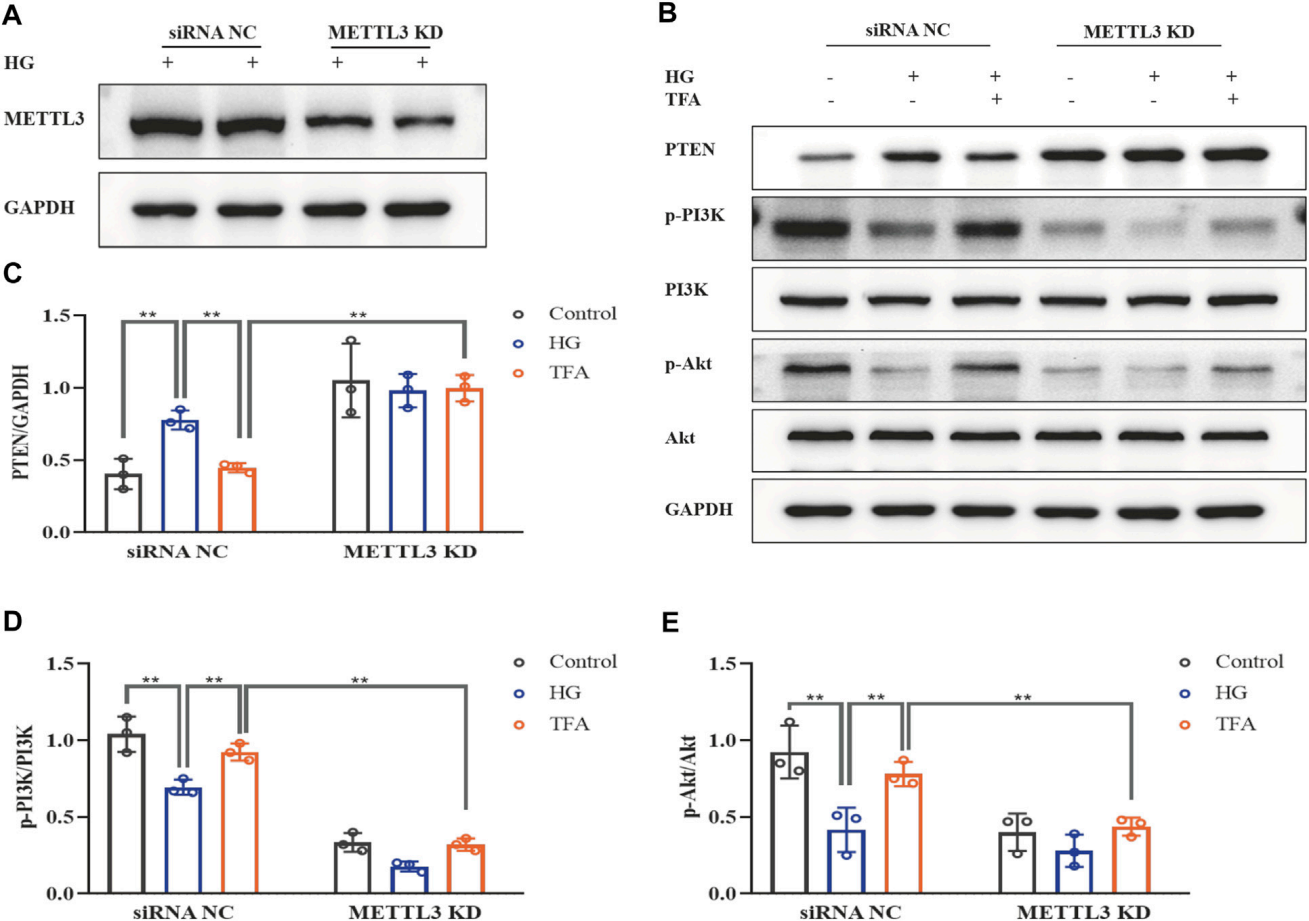
TFA regulated the PTEN/PI3K/Akt signaling pathway in a METTL3-dependent manner *via* m^6^A modification in HG-afflicted MPC-5 cells with knockdown of METTL3. **(A)** WB analysis of METTL3 in cultured MPC-5 cells transfected in duplicates with a NC siRNA or a METTL3 KD siRNA for 24 h and exposed to HG (30 mM) for 48 h; **(B)** WB analysis of PTEN, p-PI3K, PI3K, p-Akt, and Akt, in cultured MPC-5 cells transfected with NC siRNA or METTL3 KD siRNA for 24 h, following by exposure to HG at 30 mM for 48 h with or without TFA (20 μg/ml) for 24 h; **(C)** PTEN was quantified by densitometry; **(D)** p-PI3K was quantified by densitometry; **(E)** p-Akt was quantified by densitometry. Data are expressed as mean ± S.D., (*n* = 3). *p < 0.05, **p < 0.01. Abbreviations: TFA, total flavones of *Abelmoschus manihot*; PTEN, phosphate and tension homology; PI3K, phosphatidylinositol 3-kinases; Akt, protein kinase B; METTL, methyltransferase-like; m^6^A, N6-methyladenosine; HG, high glucose; MPC-5, mouse podocyte cell-5; WB, western blotting; NC, negative control; siRNA, small interfering RNA; KD, knockdown; p-PI3K, phosphorylated-phosphatidylinositol 3-kinase; p-Akt, phosphorylated-protein kinase B.

To further evaluate the effects of METTL3 on the m^6^A modification of PTEN, we detected the impact on the PTEN/PI3K/Akt pathway in HG-stimulated podocytes transfected with the negative control (NC) and METTL3 OE vectors, as well as NC and METTL3 KD siRNAs. Here, we used cDNA fragment for METTL3 to construct a pcDNA3.1 vector that could effectively elevate the expression levels of METTL3 protein when transfected into podocytes (Figure 8A). The global m^6^A content showed similar results (Figure 8B). We used METTL3 KD siRNA that could effectively decrease the expression levels of METTL3 protein when transfected into podocytes (Figure 9A). Then, we detected the protein expression levels of the key molecules within the PTEN/PI3K/Akt pathway, including PTEN, p-PI3K, and p-Akt (Figures 8C, 9B). HG increased the protein expression levels of PTEN and decreased the protein expression levels of p-PI3K and p-Akt significantly in podocytes transfected with NC vector. With the co-treatment of TFA, the protein expression levels were reversed. We found that the protein expression levels of PTEN were reduced, and p-PI3K and p-Akt were elevated in podocytes transfected with METTL3 OE vector under HG conditions when compared to that in podocytes transfected with NC vector (Figures 8D–F). While the protein expression levels of PTEN were elevated, and p-PI3K and p-Akt were decreased in podocytes transfected with METTL3 KD siRNA under HG conditions when compared to those of the cells transfected with NC siRNA (Figures 9C–E). Furthermore, TFA significantly reversed the HG-induced upregulation of PTEN, as well as the downregulation of p-PI3K and p-Akt, in podocytes overexpressing or knocking-down METTL3 when compared to those of the cells transfected with NC vector or NC siRNA (Figures 8D–F, 9C–E).

**FIGURE 10:**
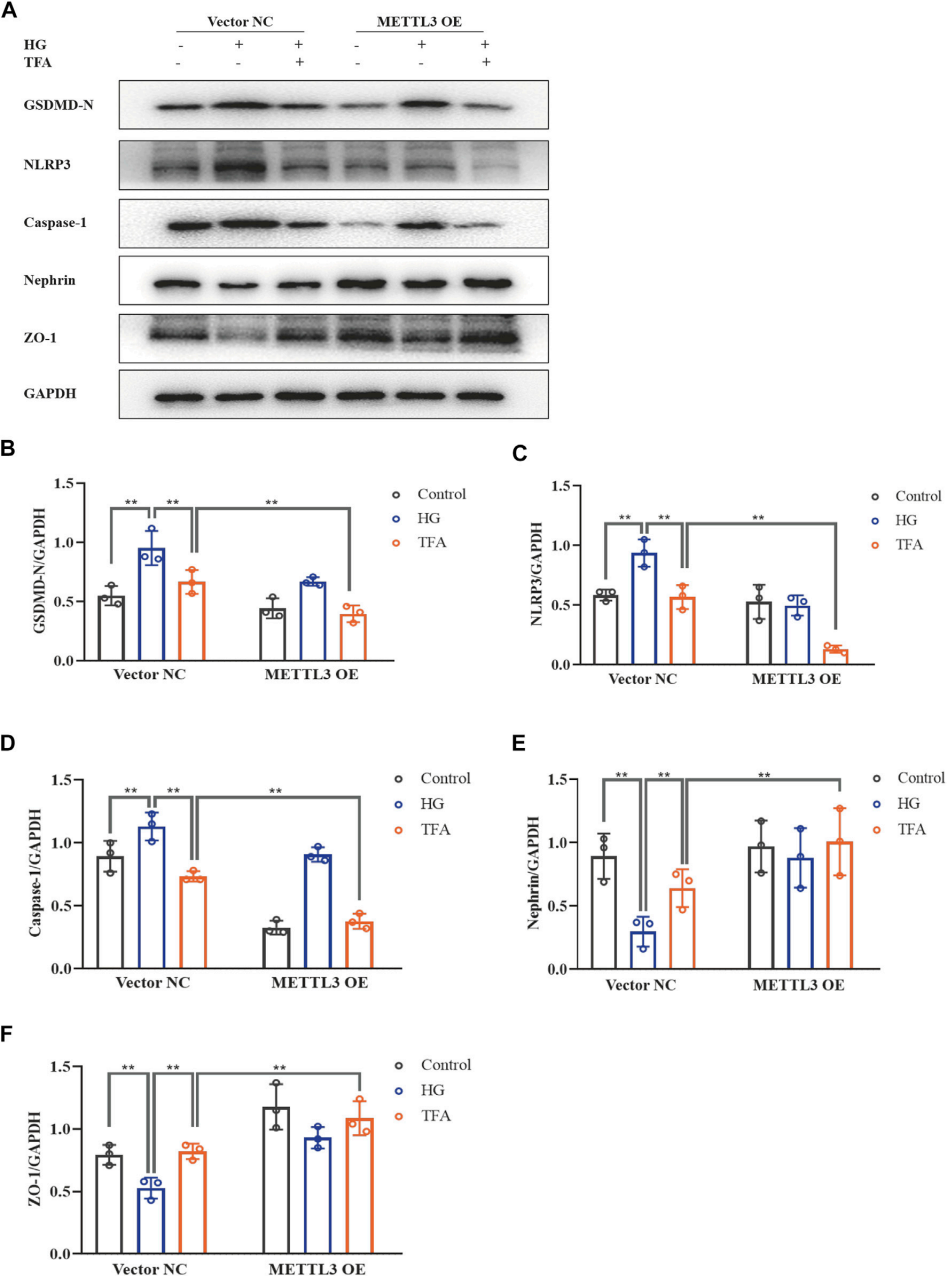
TFA attenuated podocyte pyroptosis and injury in a METTL3-dependent manner by inhibiting the activation of the NLRP3 inflammasome in HG-afflicted MPC-5 cells with overexpressed METTL3. **(A)** WB analysis of GSDMD-N, NLRP3, caspase-1, nephrin, and ZO-1, in cultured MPC-5 cells transfected with a NC vector or a METTL3 OE vector for 24 h, following by exposure to HG at 30 mM for 48 h with or without TFA (20 μg/ml) for 24 h; **(B)** GSDMD-N was quantified by densitometry; **(C)** NLRP3 was quantified by densitometry; **(D)** Caspase-1 was quantified by densitometry; **(E)** Nephrin was quantified by densitometry; **(F)** ZO-1 was quantified by densitometry. Data are expressed as mean ± S.D., (n = 3). *p < 0.05, **p < 0.01. Abbreviations: TFA, total flavones of *Abelmoschus manihot*; METTL, methyltransferase-like; NLRP3, NOD-like receptor pyrin domain-containing protein 3; HG, high glucose; MPC-5, mouse podocyte cell-5; WB, western blotting; GSDMD-N, N-terminus of GSDMD; Caspases-1, cysteinyl aspartate-specific proteinase-1; ZO-1, Zonula occludens 1; NC, negative control; OE, overexpression.

**FIGURE 11:**
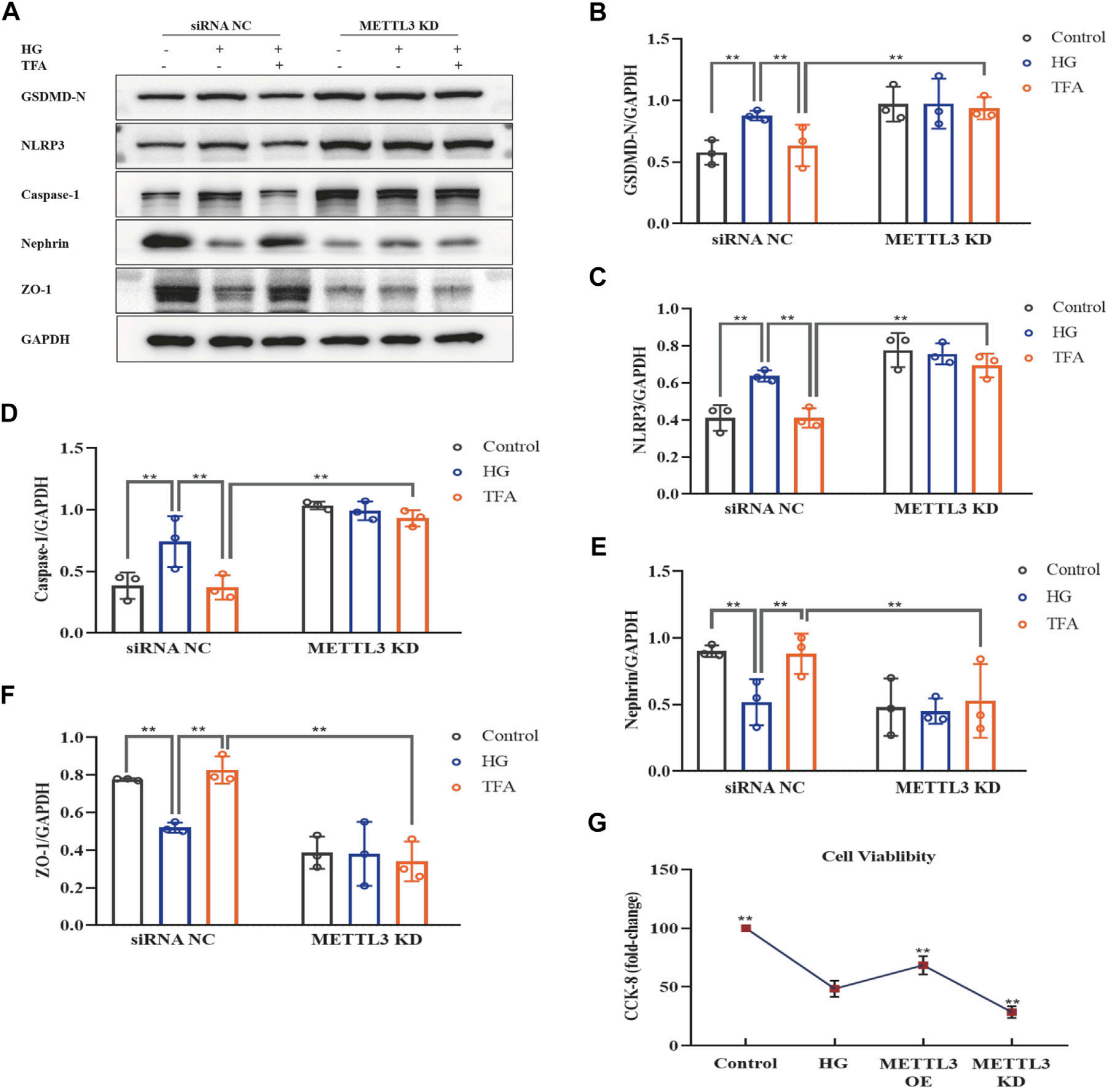
TFA attenuated podocyte pyroptosis and injury in a METTL3-dependent manner by inhibiting the activation of the NLRP3 inflammasome in HG-afflicted MPC-5 cells with knockdown of METTL3. **(A)** WB analysis of GSDMD-N, NLRP3, caspase-1, nephrin, and ZO-1, in cultured MPC-5 cells transfected with a NC siRNA or a METTL3 KD siRNA for 24 h, following by exposure to HG at 30 mM for 48 h with or without TFA (20 μg/ml) for 24 h; **(B)** GSDMD-N was quantified by densitometry; **(C)** NLRP3 was quantified by densitometry; **(D)** Caspase-1 was quantified by densitometry; **(E)** Nephrin was quantified by densitometry; **(F)** ZO-1 was quantified by densitometry; **(G)** The cell viability in cultured MPC-5 cells transfected with METTL3 OE vector or METTL3 KD siRNA for 24 h exposed to HG at 30 mM for 48 h. Data are expressed as mean ± S.D., (n = 3). *p < 0.05, **p < 0.01. Abbreviations: TFA, total flavones of *Abelmoschus manihot*; METTL, methyltransferase-like; NLRP3, NOD-like receptor pyrin domain-containing protein 3; HG, high glucose; MPC-5, mouse podocyte cell-5; WB, western blotting; GSDMD-N, N-terminus of GSDMD; Caspases-1, cysteinyl aspartate-specific proteinase-1; ZO-1, Zonula occludens 1; NC, negative control; siRNA, small interfering RNA; KD, knockdown; OE, overexpression.

Next, we investigated the activation of the NLRP3 inflammasome and podocyte injury under conditions of overexpressing or knocking-down METTL3. The protein expression levels of GSDMD-N, NLRP3, caspase-1, nephrin, and ZO-1, were detected by WB analysis (Figures 10A, 11A). HG increased the protein expression levels of GSDMD-N, NLRP3, and caspase-1, and decreased the protein expression levels of nephrin and ZO-1 significantly in podocytes transfected with NC vector. With the co-treatment of TFA, the protein expression levels were reversed. We found that the protein expression levels of GSDMD-N, NLRP3, and caspase-1 were reduced, and that the protein expression levels of nephrin and ZO-1 were increased in podocytes transfected with METTL3 OE vector in HG conditions when compared to the levels of podocytes transfected with NC vector (Figures 10B–F). While the protein expression levels of GSDMD-N, NLRP3, and caspase-1 were increased, and that the protein expression levels of nephrin and ZO-1 were decreased in podocytes transfected with METTL3 KD siRNA in HG conditions when compared to those of the cells transfected with NC siRNA (Figures 11B–F). TFA significantly reversed these protein changes expression levels in podocytes overexpressing or knocking-down METTL3 in HG conditions when compared to those of cells transfected with NC vector or NC siRNA (Figures 10B–F, 11B–F). In Figure 11G, compared to HG-treated podocytes, the viability of podocytes transfected with METTL3 OE vector was significantly increased, while it was reduced notably in podocytes transfected with METTL3 KD siRNA.

Collectively, these data strongly indicated that TFA ameliorated pyroptosis and injury in podocytes induced by HG in a METTL3-dependent manner and did so by regulating the activation of the NLRP3 inflammasome and the PTEN/PI3K/Akt signaling pathway.

## Discussion

Many traditional Chinese herbal medicines, and their active components, have been widely used to treat kidney disease, including DKD; these treatments have shown promising outcomes (Zhong et al., 2015; Guo et al., 2019). Of these, HKC, and its principal component TFA, have become very well known (Zhang et al., 2014; Chen et al., 2016). Our previous studies found that HKC could alleviate renal injury by attenuating oxidative stress and by inhibiting inflammation by regulating different signaling activities in an experimental rat model of DKD (Mao et al., 2015; Wu et al., 2018; Han et al., 2019). Moreover, TFA has been shown to exert renoprotective effects by therapeutically remodeling dysbiosis in the gut microbiota and by inhibiting intestinal metabolite-derived microinflammation (Tu et al., 2020). Zhou et al. further proved that TFA could reduce urinary albumin excretion and glomerular podocyte apoptosis in a rat model of DKD (Zhou et al., 2012). The protection of podocytes is considered to be of great significance when attempting to delay pathological injury in DKD (Podgórski et al., 2019). In contrast to podocyte apoptosis, pyroptosis is a novel form of cell death that involves the rupture of the plasma membrane and the release of proinflammatory intracellular contents. According to the different types of caspases and stimuli involved, pyroptosis can be divided into a canonical pathway (caspase-1) and a non-canonical pathway (caspase-11/4) (Platnich and Muruve, 2019); the NLRP3 inflammasome is considered to be the most important initiator of pyroptosis (Lin et al., 2020). Hou et al. proved that the restoration of podocyte autophagy by the inactivation of NLRP3 in HG conditions could reduce the extent of podocyte injury (Hou et al., 2020). It has also been reported that activation of the NLRP3 inflammasome requires NEK7, a protein that binds to the leucine-rich repeat domain of NLRP3 in a kinase-independent manner (Shi et al., 2016). Numerous studies have shown that the NLRP3 inflammasome is formed by the recruitment of the adaptor ASC and caspase-1, thus resulting in the release of an abundance of inflammatory factors, including IL-1β and IL-18, thus inducing pyroptosis and inflammatory injury in tissues (Mulay, 2019). In our present study, we found that the expression levels of pyroptosis-related proteins in the canonical pathway, and inflammatory factors that are involved in the activation of the NLRP3 inflammasome, were all upregulated in podocytes under HG conditions, including GSDMD-N, IL-1β, IL-18, NEK7, NLRP3, ASC, and caspase-1. In contrast, the expression levels of nephrin, ZO-1, WT1 and podocalyxin, proteins that are known to exert protective effects upon the podocytes, were down-regulated. However, in a previous study, Cheng et al. identified that the non-canonical inflammasome pathway was implicated in podocyte injury in a mouse model of diabetic nephropathy and involved caspase-11/4 and a GSDMD-dependent pathway (Cheng et al., 2020). Several different pathways of pyroptosis have been discovered in experimental models of DKD, thus demonstrating the complexity of this system. With TFA therapy, we found that the protein expression levels of pyroptosis in podocytes were reduced and that the expression levels of proteins associated with podocyte injury were increased in HG conditions. Moreover, MCC950, a specific small-molecule inhibitor of the NLRP3 inflammasome, yielded a similar protective effect with regards to HG-induced pyroptosis and injury in podocytes. These results suggested that TFA and MCC950 could protect HG-induced pyroptosis and injury in podocytes by inhibiting activation of the NLRP3 inflammasome.

The PI3K/Akt signaling pathway is a vital upstream element that is involved in the activation of the NLRP3 inflammasome (Ives et al., 2015). The PI3K/Akt signaling pathway is a critical mediator of survival, and plays a key regulatory role in the development of DKD (Dragos et al., 2020). Nevertheless, there has been controversy regarding whether the PI3K/Akt pathway is activated or inhibited in models of DKD or in HG-treated podocytes. Yang et al. found that the PI3K/Akt pathway was activated in podocytes that were cultured in HG at 30 mM for 24 h (Yang et al., 2020). In contrast, in the present study, we observed that the phosphorylation levels of PI3K and Akt were down-regulated in HG-stimulated podocytes, thus indicating that the PI3K/Akt pathway was suppressed in HG conditions; when co-treated with TFA, the protein expression levels of p-PI3K and p-Akt were up-regulated. Similarity, Huang et al. created a rat model of DKD by intraperitoneal injections of STZ at a dosage of 65 mg/kg and found that the protein expression levels of p-PI3K and p-Akt were notably reduced in the kidneys (Huang et al., 2016). It is possible that different sources of podocytes, or different culture environments, may induce different outcomes in relevant signaling pathways. For example, we used 740Y-P to activate the PI3K/Akt pathway. In a previous study, Wang et al. reported that 740Y-P could reduce apoptosis in foam cells by increasing the levels of phosphorylation of proteins in the PI3K/Akt signaling pathway in Raw 264.7 cells treated with Nε-carboxymethyl-lysine (Wang et al., 2019). Our data demonstrated that, similar to TFA, the use of 740Y-P as a PI3K agonist suppressed the protein expression levels of NEK7, NLRP3, ASC, and caspase-1 in cultured podocytes exposed to HG, but increased the protein expression levels of p-PI3K and p-Akt. These results suggested that TFA and 740Y-P could protect podocytes from HG-induced pyroptosis and injury by inhibiting the activation of the NLRP3 inflammasome via the PI3K/Akt signaling pathway. PTEN is a tumor-suppressing dual phosphatase that antagonizes the function of PI3K and negatively regulates Akt activity (Papa and Pandolfi, 2019). A recent study confirmed that PTEN is closely related with DKD (Khokhar et al., 2020). Our own research showed that exposure to HG conditions can increase the mRNA and protein expression levels of PTEN in podocytes and that this effect might be associated with the inhibition of PI3K/Akt signaling. Furthermore, TFA therapy was able to down-regulate the expression levels of PTEN mRNA and protein. Therefore, these findings may suggest that TFA protects podocytes against pyroptosis and injury under HG conditions via the PTEN/PI3K/Akt signaling pathway.

It has been reported that m^6^A is the most prevalent and abundant type of internal post-transcriptional RNA modification in eukaryotic cells. A range of different RNA types are involved in m^6^A methylation, including mRNAs, rRNAs, tRNAs, long non-coding RNAs, and microRNAs (Qin et al., 2020). In a previous study, Diao et al. found that the total levels of m^6^A methylated RNA and the levels of methylated PTEN mRNA were remarkably elevated in neurons exposed to hypoxia/reoxygenation (Diao et al., 2020). However, our findings showed that the global m^6^A modification level in podocytes was remarkably reduced under HG conditions, and that the levels of methylated PTEN mRNA were also reduced, as detected by Me-RIP assay. Depending on a variety of conditions, the regulation of PTEN signaling by m^6^A modifications can represent a bi-directional approach for protecting cells against disease. In our study, we found that TFA therapy reversed changes in the global level of m^6^A modification level and the levels of methylated PTEN mRNA in HG-stimulated podocytes. These results indicated that m^6^A-modified PTEN is involved in the regulation of pyroptosis and injury in podocytes under HG conditions.

The biological function of m^6^A modification is dynamically and reversibly mediated by methyltransferases (writers), demethylases (erasers), and m^6^A binding proteins (readers). The methyltransferase complex is responsible for the catalyzation of m^6^A modification and is typically composed of METTL3, METTL14, and WTAP (Tong et al., 2018). A previous study reported that the upregulation of METTL3 alleviated cytotoxic effects and pyroptosis in HG-treated RPE cells and that the knock-down of METTL3 induced the opposite effects (Zha et al., 2020). Similar changes were also observed in the present study in HG-stimulated podocytes. Our present findings revealed that the expression levels of METTL3 protein were significantly down-regulated, while the levels of METTL14 and WTAP protein did not change remarkably. The overexpression of METTL3 reduced the protein expression levels of PTEN and elevated the expression levels of p-PI3K and p-Akt proteins. TFA enhanced the activation of METTL3 methyltransferase in a dose-dependent manner in podocytes in HG conditions. Specifically, the effects of HG on the key molecules involved in the regulation of the PTEN/PI3K/Akt signaling pathway in podocytes, as well as pyroptosis and injury, were all reversed by TFA in cells transfected with METTL3 OE vector or METTL3 KD siRNA. Moreover, the interested protein expression levels under the baseline were different between NC vector and METTL3 OE vector, or between NC siRNA and METTL3 KD in podocytes. The reason for the various of the protein expression levels in podocytes under the baseline is probably due to METTL3 is an upstream key molecular in HG-treated podocytes by regulation of NLRP3-inflammasome activation and PTEN/PI3K/Akt signaling, additionally, the gene level changes of METTL3 OE or KD may affect the downstream molecules’ protein expressions.

These results indicated that TFA ameliorated pyroptosis and injury in podocytes induced by HG conditions via the PTEN/PI3K/Akt signaling pathway in a METTL3-dependent manner. Nevertheless, Xu et al. previously revealed that METTL14-mediated m^6^A RNA modification affected the PI3K/Akt signaling pathway *via* PTEN in HG-induced EMT in renal tubular cells (Xu et al., 2021), thus indicating that changes in the catalyzation of m^6^A modification under HG conditions are complex and vary across different organisms. In our study, under HG conditions, the viability of METTL3 OE vector transfected cells were similar to the viability of podocytes co-treated with the high dose of TFA (30 μg/ml) group. The reason was probably due to the effect of transfection on cell activity. However, the viability of METTL3 OE vector transfected cells was increased significantly than the HG-treated normal podocytes. The reason was probably due to the increased METTL3 gene expression may further enhance the activity of podocytes biologically. Zha et al. found the similar phenomenon that METTL3 may rescue cell viability in high-glucose treated RPE cells (Zha et al., 2020). There is an interesting phenomenon that the GSDMD-N level is not further increased in HG conditions from the baseline in the METTL3 KD group, but the cell viability was reduced notably in podocytes transfected with the METTL3 KD siRNA co-treated with HG, when compared to HG-treated normal podocytes. They showed that METTL3 may stabilize cells in a different way aside from the GSDMD pathway. Besides pyroptosis, TFA may contribute to the survival of podocytes in HG conditions by regulation of the other pathways, such as apoptosis, autophagy or necroptosis.

The present study has three limitations that need to be considered. First, we investigated the effects and mechanisms associated with the action of TFA on podocytes under HG conditions *in vitro*; however, the effects and mechanisms of TFA on experimental models of DKD *in vivo* were not assessed. Future research should include the classical DKD model. Second, both apoptosis and pyroptosis are caspase-dependent programmed cell death pathways. Caspase-3 is a common key protein in the apoptosis. However, it is reported that gasdermin E (GSDME) cleavage by caspase-3 liberates the GSDME-N domain and mediates pyroptosis by forming pores in the plasma membrane. Caspase-3-mediated inflammasome pathway is also a pathway of pyroptosis generation in DKD (Liu et al., 2021). The effect of TFA in regulating apoptosis and caspase-3-mediated inflammasome pathway will be focused in our further study. Third, a novel and vital finding of the current study was that METTL3-dependent m^6^A modification and methylated PTEN expression levels were reduced in podocytes undergoing HG-induced pyroptosis and injury; however, m^6^A modification is complex. Further studies are now needed to explore whether demethylases and m^6^A binding proteins participate in the modification of PTEN in podocytes under HG conditions.

**FIGURE 12:**
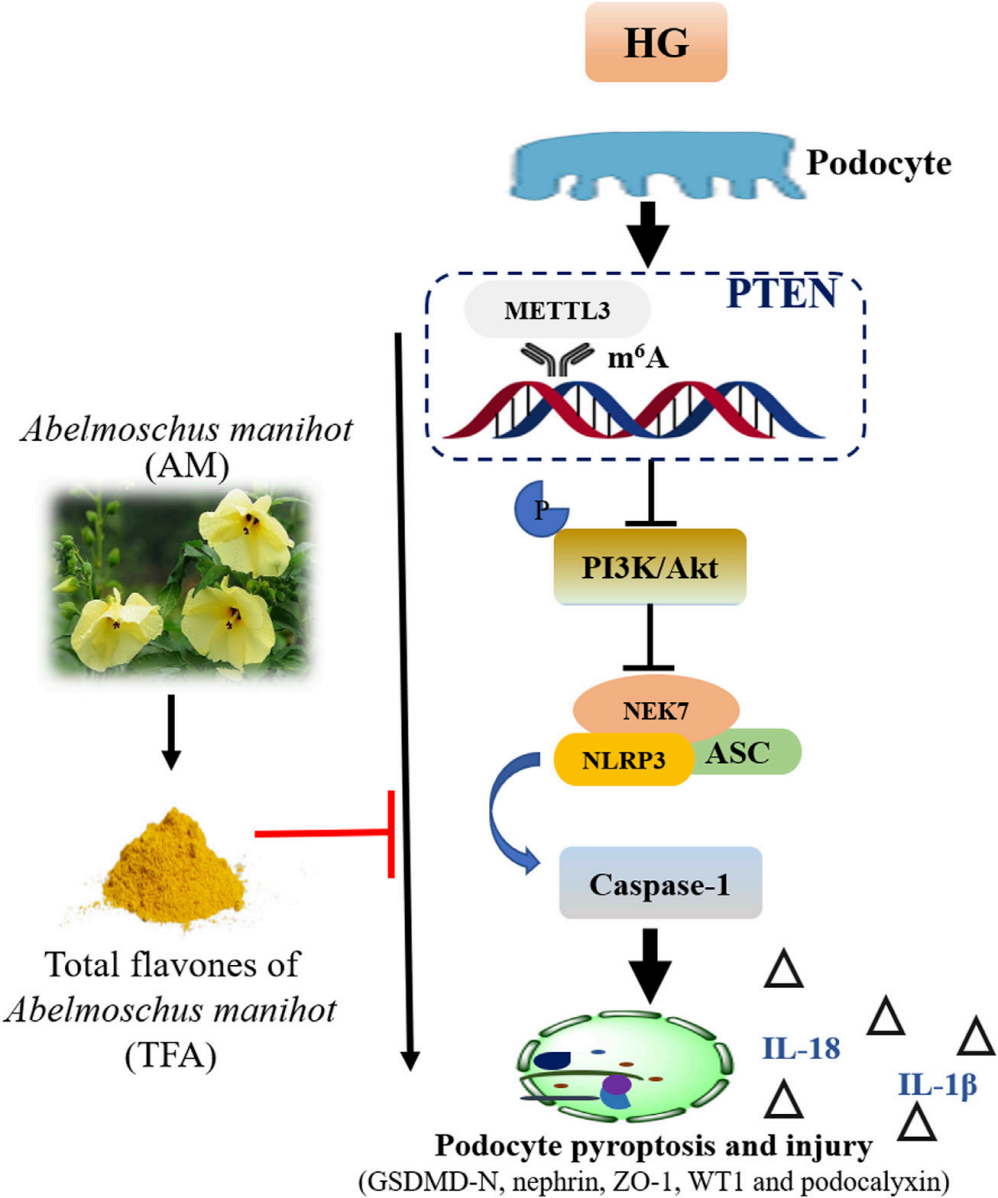
Schematic diagram showing how TFA ameliorates pyroptosis and injury in podocytes exposed to HG conditions. Abbreviations: TFA, total flavones of *Abelmoschus manihot*; HG, high glucose.

Collectively, we demonstrated that TFA could ameliorate HG-induced pyroptosis and injury in podocytes by targeting METTL3-dependent m^6^A modification *via* the regulation of NLRP3-inflammasome activation and PTEN/PI3K/Akt signaling (Figure 12). This study uncovered the underlying mechanisms of pyroptosis and injury in podocytes in DKD, at least in part, and provides a better understanding of the role of TFA in protecting podocytes during the progression of DKD.

## Data Availability

The original contributions presented in the study are included in the article/Supplementary Material, further inquiries can be directed to the corresponding author.

## Glossary

Akt: protein kinase B
AM: *Abelmoschus manihot*
ANOVA: analysis of variance
ASC: a C-terminal caspase recruitment domain
Caspases-1: cysteinyl aspartate-specific proteinase-1
cDNA: complementary DNA
DKD: diabetic kidney disease
DMEM: Dulbecco’s modified eagle medium
DMSO: dimethyl sulfoxide
EMT: epithelial-mesenchymal transition
ESRD: end-stage renal disease
FBS: fetal bovine serum
GSDMD: gasdermin D
GSDME: gasdermin E
GSDMD-N: N-terminus of GSDMD
HG: high glucose
HKC: Huangkui capsule
IFN-γ: recombinant γ-interferon
IL-1β: interleukin-1β
IL-18: interleukin-18
KD: knockdown
LSD: least significant difference
MeRIP: methylated RNA immunoprecipitation
METTL: methyltransferase-like
MPC-5: mouse podocyte cell-5
m^6^A: N6-methyladenosine
NC: negative control
NEK7: NIMA-related kinase 7
NLRP3: NOD-like receptor pyrin domain-containing protein 3
OE: overexpression
OD: optical density
p-PI3K: phosphorylated-phosphatidylinositol 3-kinase
p-Akt: phosphorylated-protein kinase B
PI3K: phosphatidylinositol 3-kinases
PTEN: phosphate and tension homology
qRT-PCR: quantitative real-time polymerase chain reaction
RPE: retinal pigment epithelium
SD: standard deviation
siRNA: small interfering RNA
STZ: streptozotocin
TFA: total flavones of *Abelmoschus manihot*
WB: western blotting
WT1: Wilms tumor type 1
WTAP: Wilms tumor 1-associated protein
ZO-1: Zonula occludens 1

## References

[B1] Chen Y. Cai G. Sun X. Chen X. (2016). Treatment of Chronic Kidney Disease Using a Traditional Chinese medicine, Flos Abelmoschus manihot (Linnaeus) Medicus (Malvaceae). Clin. Exp. Pharmacol. Physiol. 43, 145–148. 10.1111/1440-1681.12528 26667396

[B2] Cheng Q. Pan J. Zhou Z. L. Yin F. Xie H. Y. Chen P. P. (2020). Caspase-11/4 and Gasdermin D-Mediated Pyroptosis Contributes to Podocyte Injury in Mouse Diabetic Nephropathy. Acta Pharmacol. Sin. 42, 954–963. 10.1038/s41401-020-00525-z 32968210 PMC8149386

[B3] De Vasconcelos N. M. Lamkanfi M. (2020). Recent Insights on Inflammasomes, Gasdermin Pores, and Pyroptosis. Cold Spring Harb. Perspect. Biol. 12, a036392. 10.1101/cshperspect.a036392 31570336 PMC7197430

[B4] Diao M.-Y. Zhu Y. Yang J. Xi S.-S. Wen X. Gu Q. (2020). Hypothermia Protects Neurons against Ischemia/reperfusion-Induced Pyroptosis via m6A-Mediated Activation of PTEN and the PI3K/Akt/GSK-3β Signaling Pathway. Brain Res. Bull. 159, 25–31. 10.1016/j.brainresbull.2020.03.011 32200003

[B5] Dragos D. Manea M. M. Timofte D. Ionescu D. (2020). Mechanisms of Herbal Nephroprotection in Diabetes Mellitus. J. Diabetes Res. 2020, 5710513. 10.1155/2020/5710513 32695828 PMC7362309

[B6] Guo J. Gao Y. Wang Y. Liu W. J. Zhou J. Wang Z. (2019). Application of Herbal Medicines with Heat-Clearing Property to Anti-microinflammation in the Treatment of Diabetic Kidney Disease. Evid. Based Complement. Alternat. Med. 2019, 6174350. 10.1155/2019/6174350 31281401 PMC6590606

[B7] Han W. Ma Q. Liu Y. Wu W. Tu Y. Huang L. (2019). Huangkui Capsule Alleviates Renal Tubular Epithelial-Mesenchymal Transition in Diabetic Nephropathy via Inhibiting NLRP3 Inflammasome Activation and TLR4/NF-Κb Signaling. Phytomedicine 57, 203–214. 10.1016/j.phymed.2018.12.021 30785016

[B8] Hou Y. Lin S. Qiu J. Sun W. Dong M. Xiang Y. (2020). NLRP3 Inflammasome Negatively Regulates Podocyte Autophagy in Diabetic Nephropathy. Biochem. Biophysical Res. Commun. 521, 791–798. 10.1016/j.bbrc.2019.10.194 31703838

[B9] Huang G. Lv J. Li T. Huai G. Li X. Xiang S. (2016). Notoginsenoside R1 Ameliorates Podocyte Injury in Rats with Diabetic Nephropathy by Activating the PI3K/Akt Signaling Pathway. Int. J. Mol. Med. 38, 1179–1189. 10.3892/ijmm.2016.2713 27571993 PMC5029967

[B10] Ives A. Nomura J. Martinon F. Roger T. Leroy D. Miner J. N. (2015). Xanthine Oxidoreductase Regulates Macrophage IL1β Secretion upon NLRP3 Inflammasome Activation. Nat. Commun. 6, 6555. 10.1038/ncomms7555 25800347 PMC4382995

[B11] Jafari M. Ghadami E. Dadkhah T. Akhavan‐Niaki H. (2019). PI3k/AKT Signaling Pathway: Erythropoiesis and beyond. J. Cel. Physiol. 234, 2373–2385. 10.1002/jcp.27262 30192008

[B12] Kato M. Natarajan R. (2019). Epigenetics and Epigenomics in Diabetic Kidney Disease and Metabolic Memory. Nat. Rev. Nephrol. 15, 327–345. 10.1038/s41581-019-0135-6 30894700 PMC6889804

[B13] Khokhar M. Roy D. Modi A. Agarwal R. Yadav D. Purohit P. (2020). Perspectives on the Role of PTEN in Diabetic Nephropathy: an Update. Crit. Rev. Clin. Lab. Sci. 57, 470–483. 10.1080/10408363.2020.1746735 32306805

[B14] Li N. Tang H. Wu L. Ge H. Wang Y. Yu H. (2021). Chemical Constituents, Clinical Efficacy and Molecular Mechanisms of the Ethanol Extract of Abelmoschus Manihot Flowers in Treatment of Kidney Diseases. Phytotherapy Res. 35, 198–206. 10.1002/ptr.6818 PMC7891592 32716080

[B15] Lin J. Cheng A. Cheng K. Deng Q. Zhang S. Lan Z. (2020). New Insights into the Mechanisms of Pyroptosis and Implications for Diabetic Kidney Disease. Ijms 21, 7057. 10.3390/ijms21197057 PMC7583981 32992874

[B16] Liu P. Zhang Z. Li Y. (2021). Relevance of the Pyroptosis-Related Inflammasome Pathway in the Pathogenesis of Diabetic Kidney Disease. Front. Immunol. 12, 603416. 10.3389/fimmu.2021.603416 33692782 PMC7937695

[B17] Maidarti M. Anderson R. A. Telfer E. E. (2020). Crosstalk between PTEN/PI3K/Akt Signalling and DNA Damage in the Oocyte: Implications for Primordial Follicle Activation, Oocyte Quality and Ageing. Cells 9, 200. 10.3390/cells9010200 9 PMC7016612 31947601

[B18] Mao Z.-M. Shen S.-M. Wan Y.-G. Sun W. Chen H.-L. Huang M.-M. (2015). Huangkui Capsule Attenuates Renal Fibrosis in Diabetic Nephropathy Rats through Regulating Oxidative Stress and p38MAPK/Akt Pathways, Compared to α-lipoic Acid. J. Ethnopharmacology 173, 256–265. 10.1016/j.jep.2015.07.036 26226437

[B19] Mulay S. R. (2019). Multifactorial Functions of the Inflammasome Component NLRP3 in Pathogenesis of Chronic Kidney Diseases. Kidney Int. 96, 58–66. 10.1016/j.kint.2019.01.014 30922667

[B20] Papa A. Pandolfi P. P. (2019). The PTEN-Pi3k Axis in Cancer. Biomolecules 9, 153. 10.3390/biom9040153 PMC6523724 30999672

[B21] Platnich J. M. Muruve D. A. (2019). NOD-like Receptors and Inflammasomes: A Review of Their Canonical and Non-canonical Signaling Pathways. Arch. Biochem. Biophys. 670, 4–14. 10.1016/j.abb.2019.02.008 30772258

[B22] Podgórski P. Konieczny A. Lis Ł. Witkiewicz W. Hruby Z. (2019). Glomerular Podocytes in Diabetic Renal Disease. Adv. Clin. Exp. Med. 28, 1711–1715. 10.17219/acem/104534 31851794

[B23] Qin Y. Li L. Luo E. Hou J. Yan G. Wang D. (2020). Role of m6A RNA Methylation in Cardiovascular Disease (Review). Int. J. Mol. Med. 46, 1958–1972. 10.3892/ijmm.2020.4746 33125109 PMC7595665

[B24] Reidy K. Kang H. M. Hostetter T. Susztak K. (2014). Molecular Mechanisms of Diabetic Kidney Disease. J. Clin. Invest. 124, 2333–2340. 10.1172/jci72271 24892707 PMC4089448

[B25] Sharif H. Wang L. Wang W. L. Magupalli V. G. Andreeva L. Qiao Q. (2019). Structural Mechanism for NEK7-Licensed Activation of NLRP3 Inflammasome. Nature 570, 338–343. 10.1038/s41586-019-1295-z 31189953 PMC6774351

[B26] Shi H. Wang Y. Li X. Zhan X. Tang M. Fina M. (2016). NLRP3 Activation and Mitosis Are Mutually Exclusive Events Coordinated by NEK7, a New Inflammasome Component. Nat. Immunol. 17, 250–258. 10.1038/ni.3333 26642356 PMC4862588

[B27] Shi Y. Hu F. B. (2014). The Global Implications of Diabetes and Cancer. The Lancet 383, 1947–1948. 10.1016/s0140-6736(14)60886-2 24910221

[B28] Song Z. Guo Y. Zhou M. Zhang X. (2014). The PI3K/p-Akt Signaling Pathway Participates in Calcitriol Ameliorating Podocyte Injury in DN Rats. Metabolism 63, 1324–1333. 10.1016/j.metabol.2014.06.013 25044177

[B29] Tong J. Flavell R. A. Li H.-B. (2018). RNA m6A Modification and its Function in Diseases. Front. Med. 12, 481–489. 10.1007/s11684-018-0654-8 30097961

[B30] Tu Y. Fang Q. J. Sun W. Liu B. H. Liu Y. L. Wu W. (2020). Total Flavones of Abelmoschus Manihot Remodels Gut Microbiota and Inhibits Microinflammation in Chronic Renal Failure Progression by Targeting Autophagy-Mediated Macrophage Polarization. Front. Pharmacol. 11, 566611. 10.3389/fphar.2020.566611 33101025 PMC7554637

[B31] Wang D. Li Y. Wu C. Liu Y. (2011). PINCH1 Is Transcriptional Regulator in Podocytes that Interacts with WT1 and Represses Podocalyxin Expression. Plos. One. 6, e17048. 10.1371/journal.pone.0017048 21390327 PMC3044754

[B32] Wang X.-M. Yao M. Liu S.-X. Hao J. Liu Q.-J. Gao F. (2014). Interplay between the Notch and PI3K/Akt Pathways in High Glucose-Induced Podocyte Apoptosis. Am. J. Physiology-Renal Physiol. 306, F205–F213. 10.1152/ajprenal.90005.2013 24226527

[B33] Wang Z. Bao Z. Ding Y. Xu S. Du R. Yan J. (2019). Nε-carboxymethyl-lysine-induced PI3K/Akt Signaling Inhibition Promotes Foam Cell Apoptosis and Atherosclerosis Progression. Biomed. Pharmacother. 115, 108880. 10.1016/j.biopha.2019.108880 31035012

[B34] Wu W. Hu W. Han W. B. Liu Y. L. Tu Y. Yang H. M. (2018). Inhibition of Akt/mTOR/p70S6K Signaling Activity with Huangkui Capsule Alleviates the Early Glomerular Pathological Changes in Diabetic Nephropathy. Front. Pharmacol. 9, 443. 10.3389/fphar.2018.00443 29881349 PMC5976825

[B35] Xing L. Liu Q. Fu S. Li S. Yang L. Liu S. (2015). PTEN Inhibits High Glucose-Induced Phenotypic Transition in Podocytes. J. Cel. Biochem. 116, 1776–1784. 10.1002/jcb.25136 25736988

[B36] Xu Z. Jia K. Wang H. Gao F. Zhao S. Li F. (2021). METTL14-regulated PI3K/Akt Signaling Pathway via PTEN Affects HDAC5-Mediated Epithelial-Mesenchymal Transition of Renal Tubular Cells in Diabetic Kidney Disease. Cell Death Dis. 12, 32. 10.1038/s41419-020-03312-0 33414476 PMC7791055

[B37] Yang F. Qu Q. Zhao C. Liu X. Yang P. Li Z. (2020). Paecilomyces Cicadae-Fermented Radix Astragali Activates Podocyte Autophagy by Attenuating PI3K/AKT/mTOR Pathways to Protect against Diabetic Nephropathy in Mice. Biomed. Pharmacother. 129, 110479. 10.1016/j.biopha.2020.110479 32768963

[B38] Ying Q. Wu G. (2017). Molecular Mechanisms Involved in Podocyte EMT and Concomitant Diabetic Kidney Diseases: an Update. Ren. Fail. 39, 474–483. 10.1080/0886022x.2017.1313164 28413908 PMC6014344

[B39] Yu Z.-W. Zhang J. Li X. Wang Y. Fu Y.-H. Gao X.-Y. (2020). A New Research Hot Spot: The Role of NLRP3 Inflammasome Activation, a Key Step in Pyroptosis, in Diabetes and Diabetic Complications. Life Sci. 240, 117138. 10.1016/j.lfs.2019.117138 31809715

[B40] Zha X. Xi X. Fan X. Ma M. Zhang Y. Yang Y. (2020). Overexpression of METTL3 Attenuates High-Glucose Induced RPE Cell Pyroptosis by Regulating miR-25-3p/PTEN/Akt Signaling cascade through DGCR8. Aging 12, 8137–8150. 10.18632/aging.103130 32365051 PMC7244028

[B41] Zhang L. Li P. Xing C.-y. Zhao J.-y. He Y.-n. Wang J.-q. (2014). Efficacy and Safety of Abelmoschus Manihot for Primary Glomerular Disease: a Prospective, Multicenter Randomized Controlled Clinical Trial. Am. J. Kidney Dis. 64, 57–65. 10.1053/j.ajkd.2014.01.431 24631042

[B42] Zhong Y. Menon M. C. Deng Y. Chen Y. He J. C. (2015). Recent Advances in Traditional Chinese Medicine for Kidney Disease. Am. J. Kidney Dis. 66, 513–522. 10.1053/j.ajkd.2015.04.013 26015275

[B43] Zhou L. An X.-F. Teng S.-C. Liu J.-S. Shang W.-B. Zhang A.-H. (2012). Pretreatment with the Total Flavone Glycosides of Flos Abelmoschus Manihot and Hyperoside Prevents Glomerular Podocyte Apoptosis in Streptozotocin-Induced Diabetic Nephropathy. J. Med. Food 15, 461–468. 10.1089/jmf.2011.1921 22439874 PMC3338104

