# Supplementary figures and images for: Total Flavones of Abelmoschus manihot Ameliorates Podocyte Pyroptosis and Injury in High Glucose Conditions by Targeting METTL3-Dependent m6A Modification-Mediated NLRP3-Inflammasome Activation and PTEN/PI3K/Akt Signaling (part 1 of 6)
Source: Front Pharmacol. 2021 Jul 15;12:667644. doi: 10.3389/fphar.2021.667644 (PMC8319635; doi:10.3389/fphar.2021.667644)

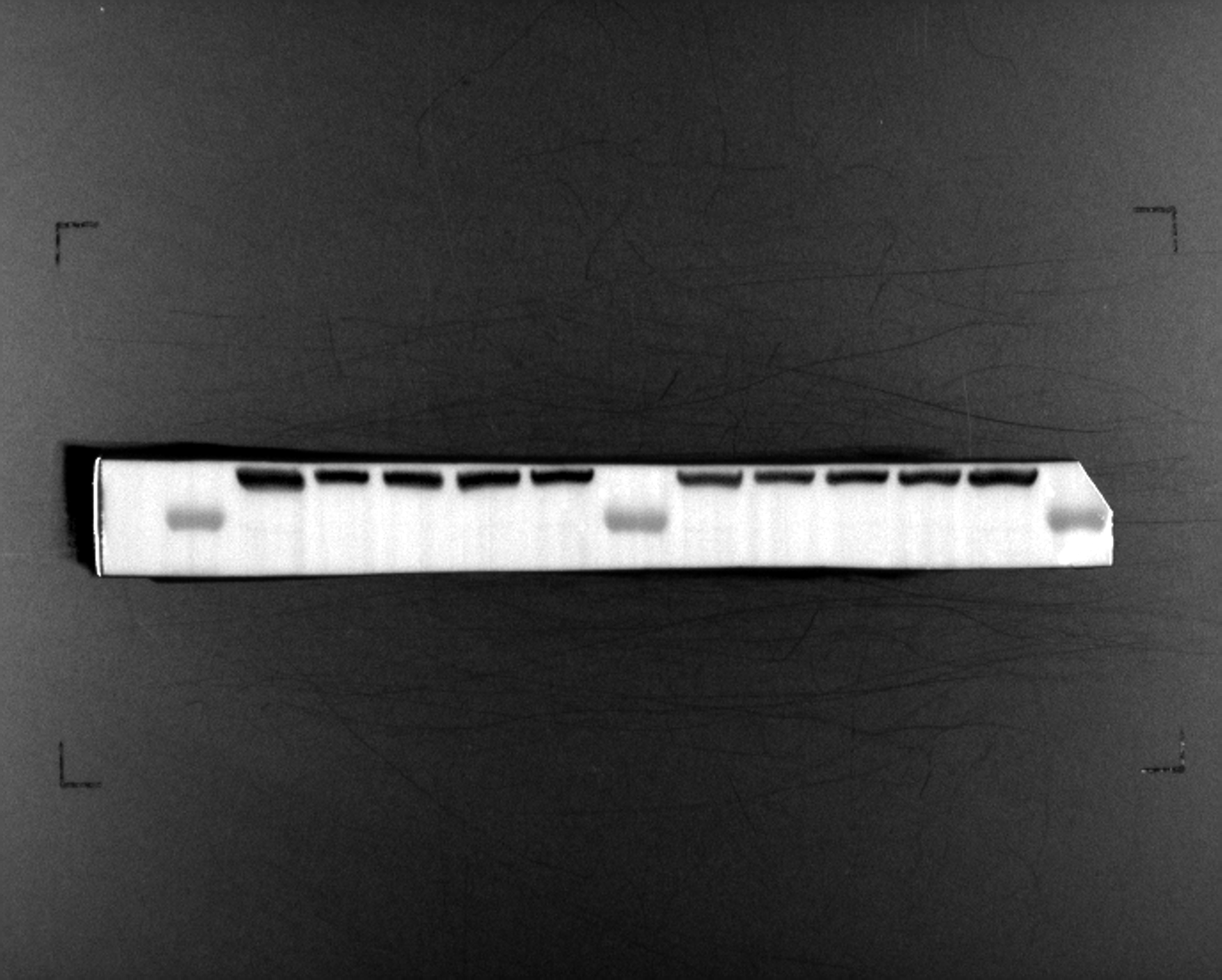

Supplement: Supplementary file 1 [file DataSheet3.zip › Fig.2/WBμ¥íσ╕a/Podocalyxin/1-Podocalyxin-10s YT.Tif]

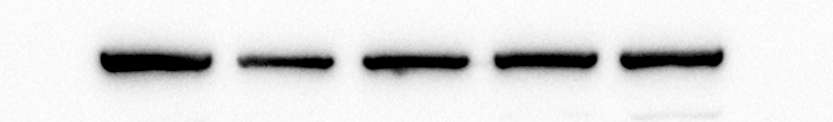

Supplement: Supplementary file 1 [file DataSheet3.zip › Fig.2/WBμ¥íσ╕a/Podocalyxin/τö¿-PS 2-Podocalyxin-10s.tif]

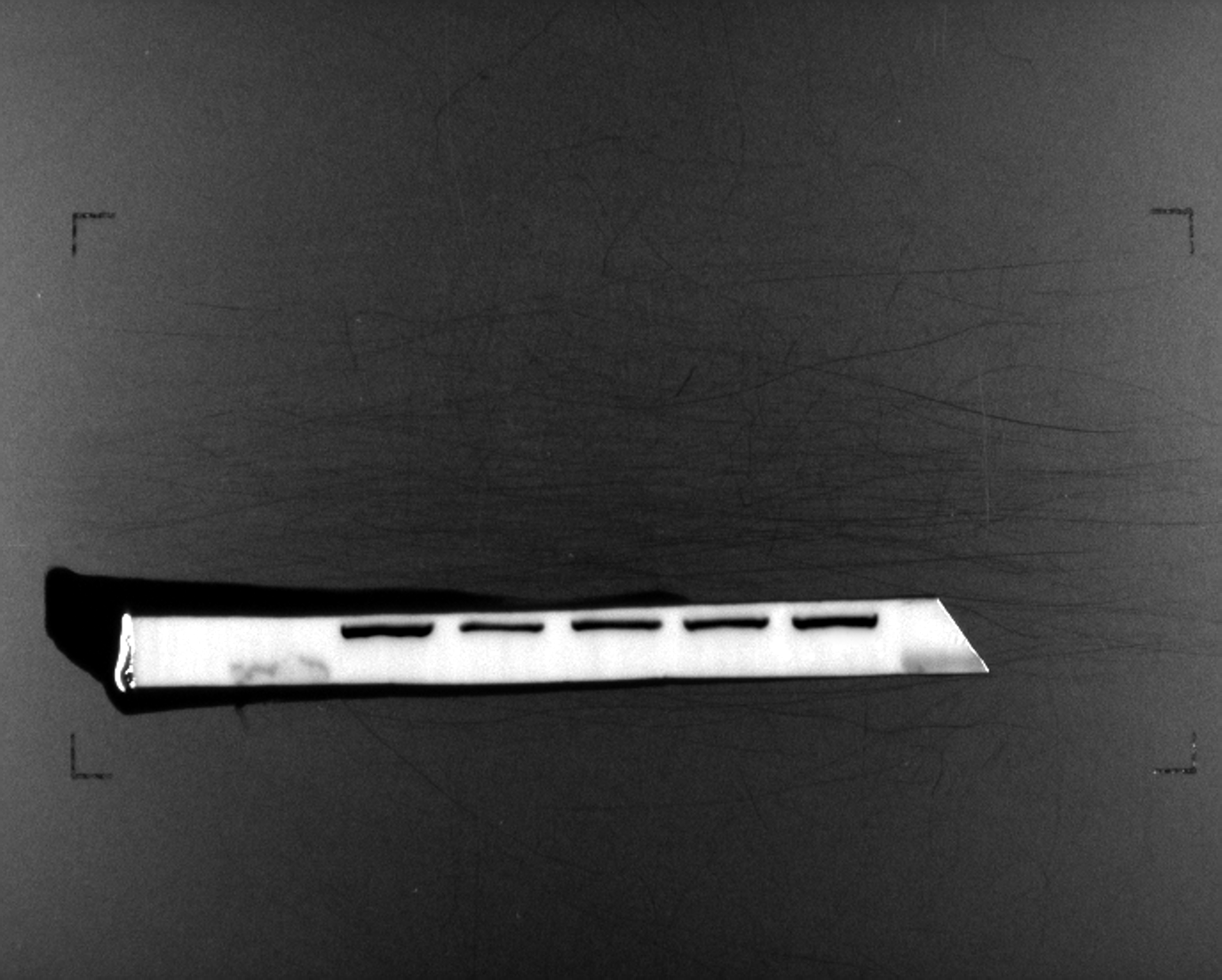

Supplement: Supplementary file 1 [file DataSheet3.zip › Fig.2/WBμ¥íσ╕a/Podocalyxin/2-Podocalyxin-10s YT.Tif]

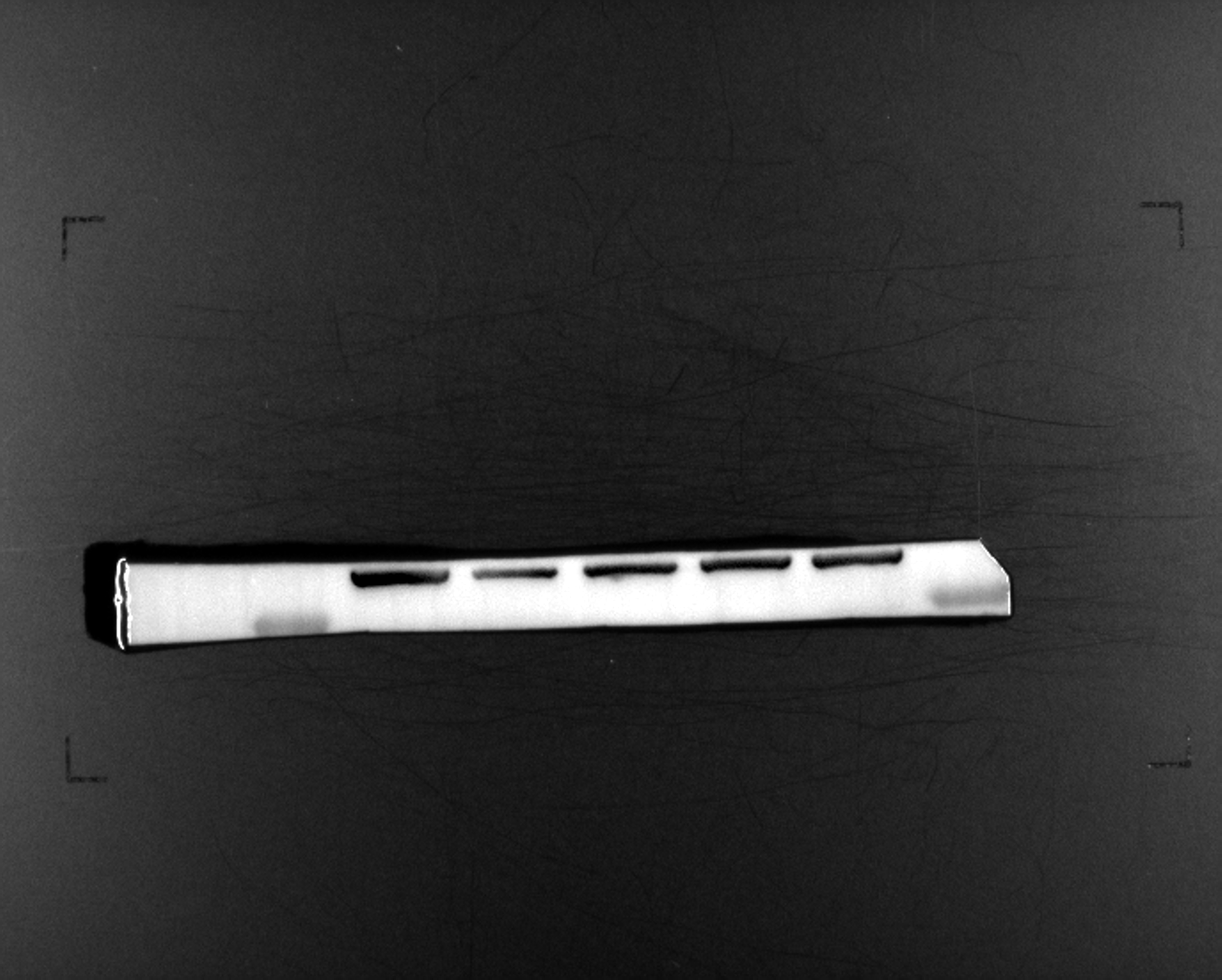

Supplement: Supplementary file 1 [file DataSheet3.zip › Fig.2/WBμ¥íσ╕a/Podocalyxin/3-Podocalyxin-10s YT.Tif]

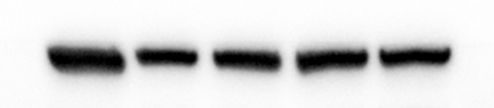

Supplement: Supplementary file 1 [file DataSheet3.zip › Fig.2/WBμ¥íσ╕a/Podocalyxin/PS-σ╖a-1-Podocalyxin-10s.tif]

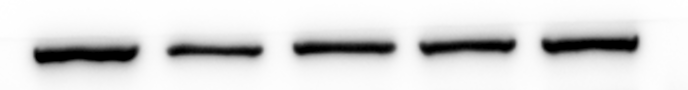

Supplement: Supplementary file 1 [file DataSheet3.zip › Fig.2/WBμ¥íσ╕a/Podocalyxin/PS 3-Podocalyxin-10s.tif]

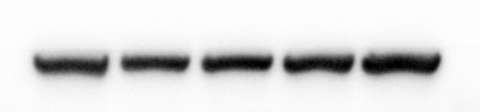

Supplement: Supplementary file 1 [file DataSheet3.zip › Fig.2/WBμ¥íσ╕a/Podocalyxin/PS-σÅ│-1-Podocalyxin-10s.tif]

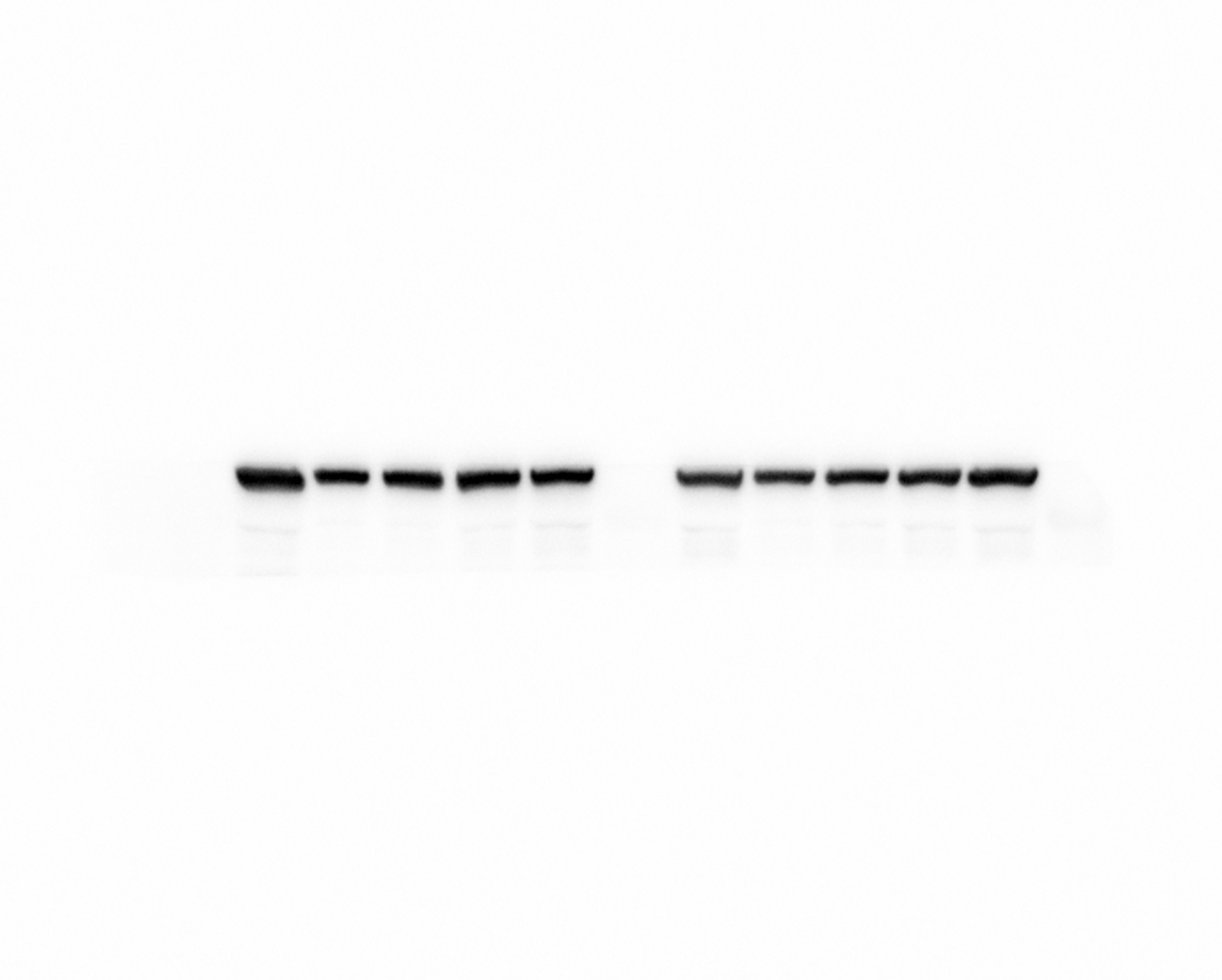

Supplement: Supplementary file 1 [file DataSheet3.zip › Fig.2/WBμ¥íσ╕a/Podocalyxin/1-Podocalyxin-10s.Tif]

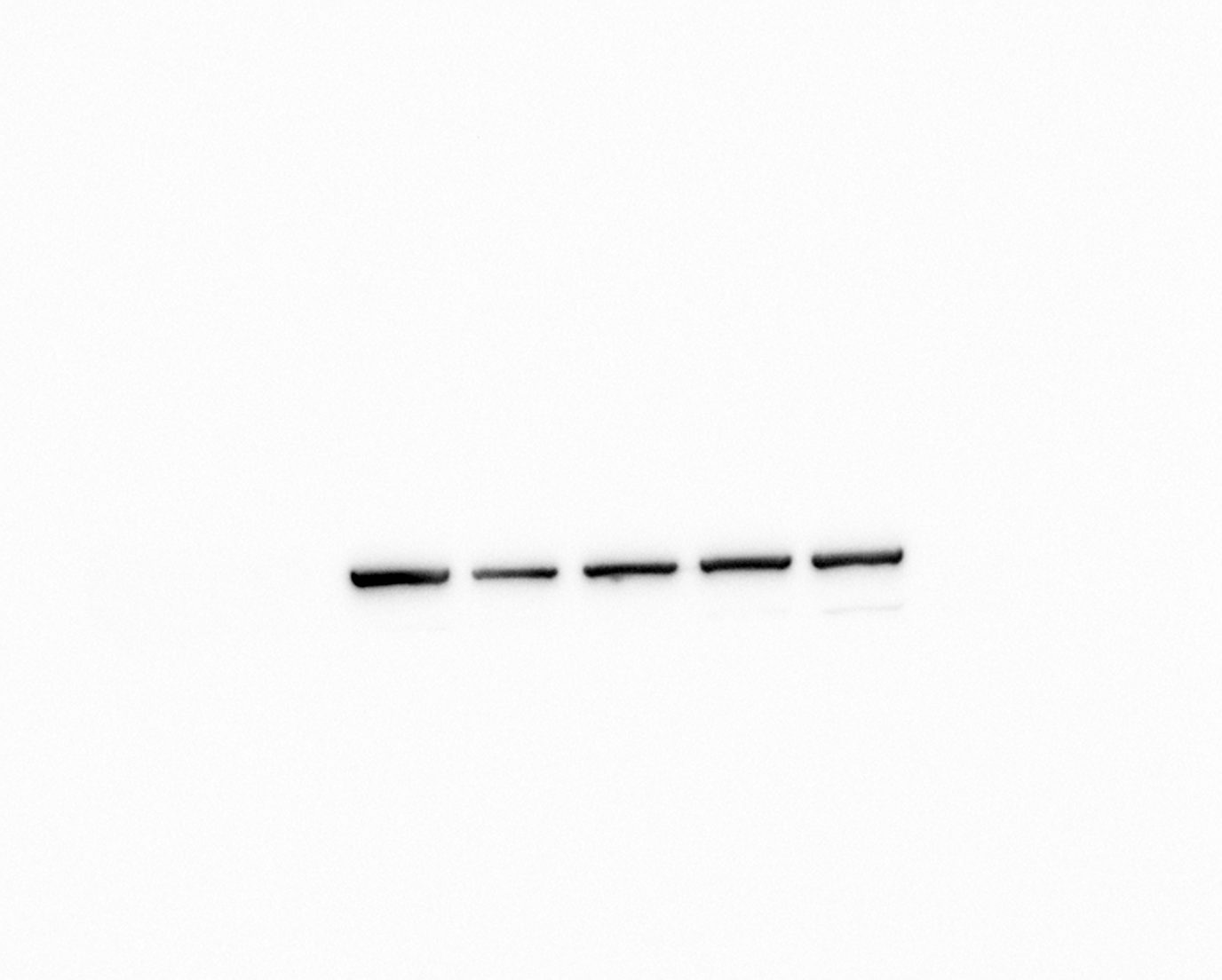

Supplement: Supplementary file 1 [file DataSheet3.zip › Fig.2/WBμ¥íσ╕a/Podocalyxin/2-Podocalyxin-10s.Tif]

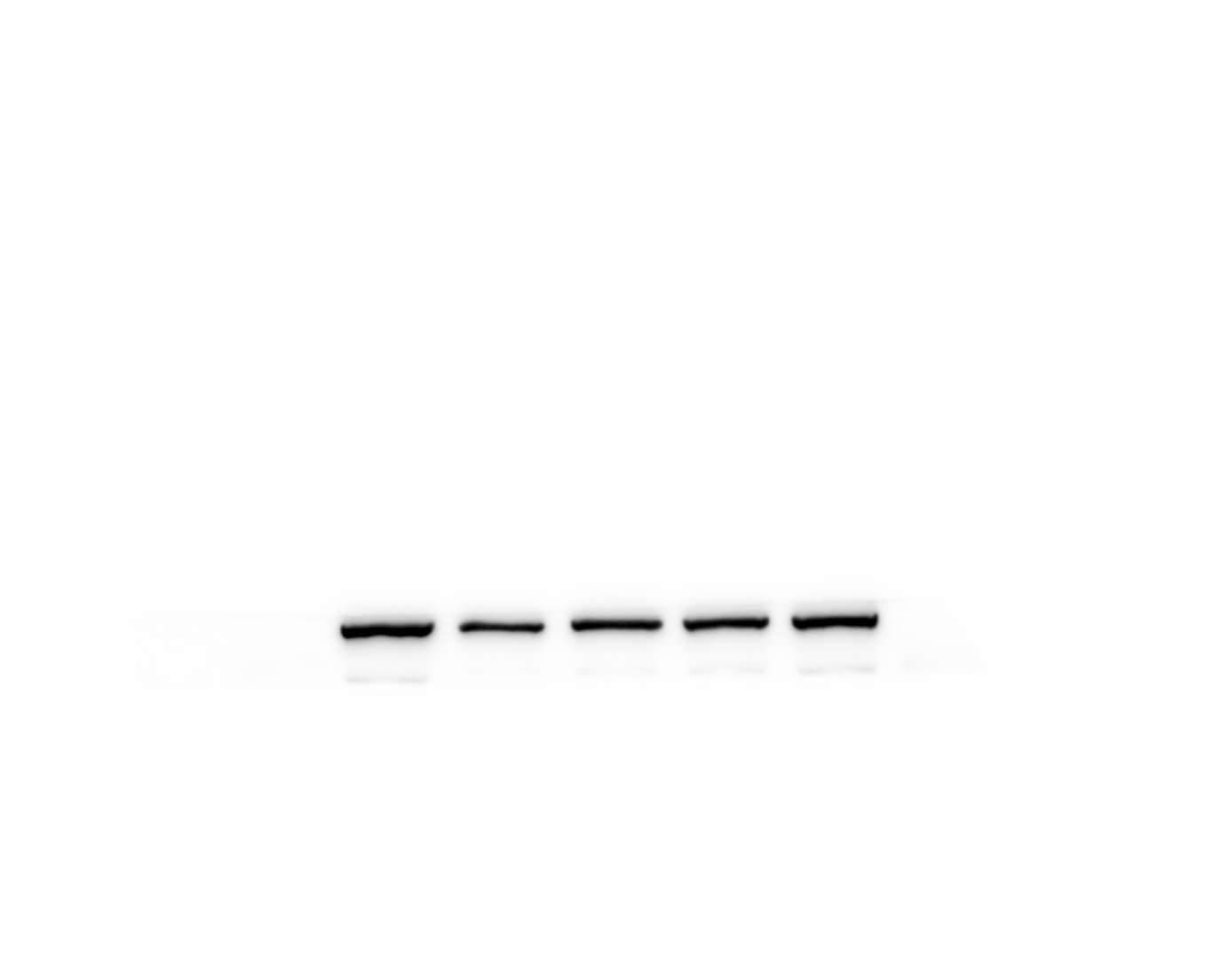

Supplement: Supplementary file 1 [file DataSheet3.zip › Fig.2/WBμ¥íσ╕a/Podocalyxin/3-Podocalyxin-10s.Tif]

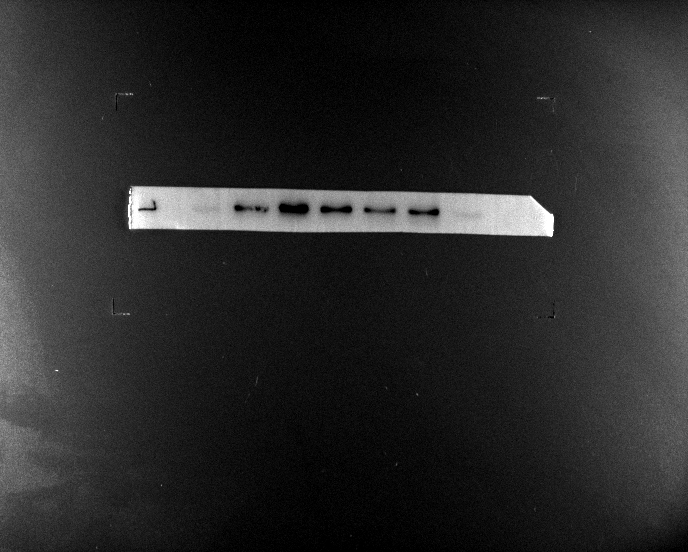

Supplement: Supplementary file 1 [file DataSheet3.zip › Fig.2/WBμ¥íσ╕a/IL-18/3-IL18 YT.tif]

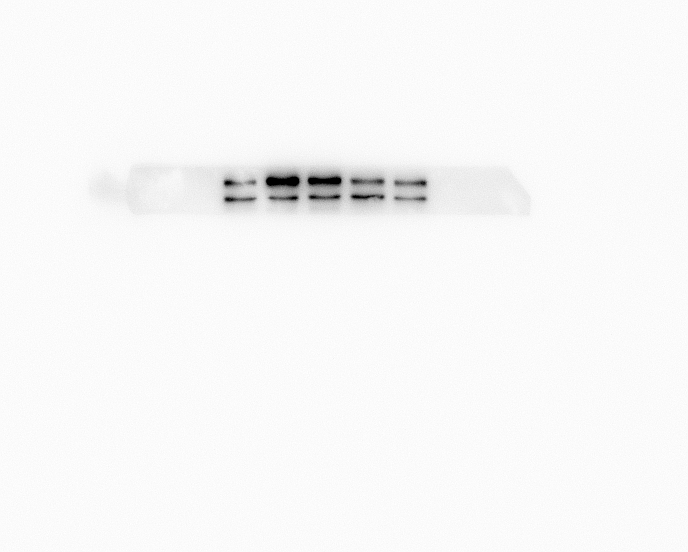

Supplement: Supplementary file 1 [file DataSheet3.zip › Fig.2/WBμ¥íσ╕a/IL-18/2-IL18.tif]

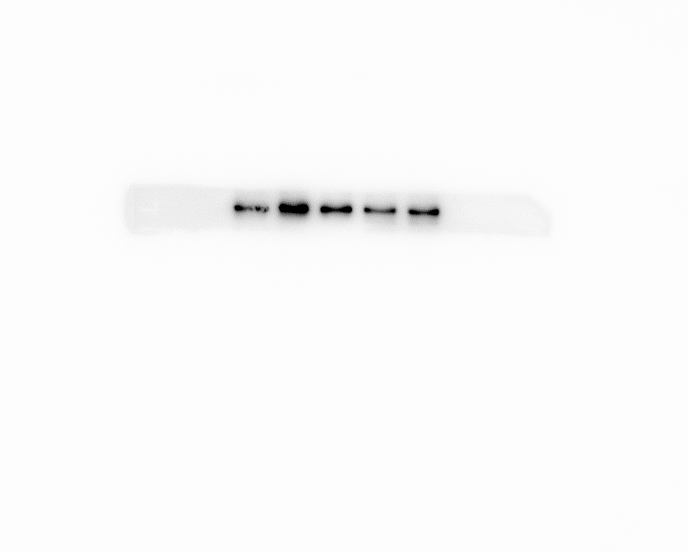

Supplement: Supplementary file 1 [file DataSheet3.zip › Fig.2/WBμ¥íσ╕a/IL-18/3-IL18.tif]

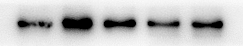

Supplement: Supplementary file 1 [file DataSheet3.zip › Fig.2/WBμ¥íσ╕a/IL-18/3.tif]

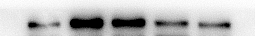

Supplement: Supplementary file 1 [file DataSheet3.zip › Fig.2/WBμ¥íσ╕a/IL-18/2.tif]

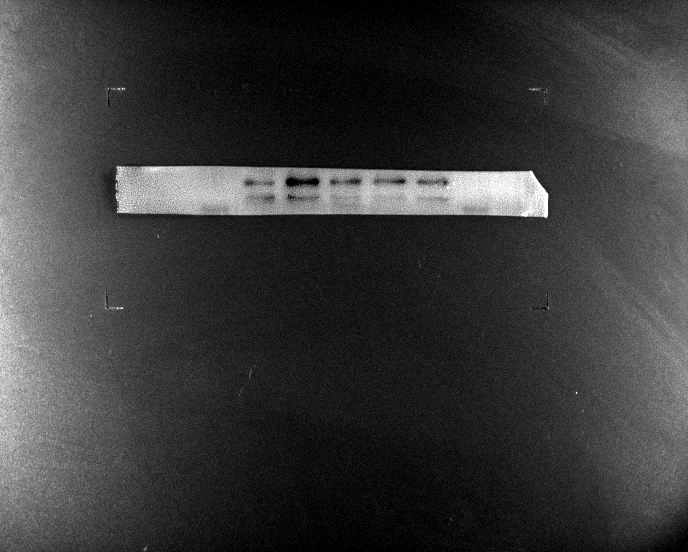

Supplement: Supplementary file 1 [file DataSheet3.zip › Fig.2/WBμ¥íσ╕a/IL-18/1-IL18 YT.tif]

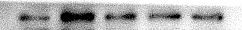

Supplement: Supplementary file 1 [file DataSheet3.zip › Fig.2/WBμ¥íσ╕a/IL-18/1.tif]

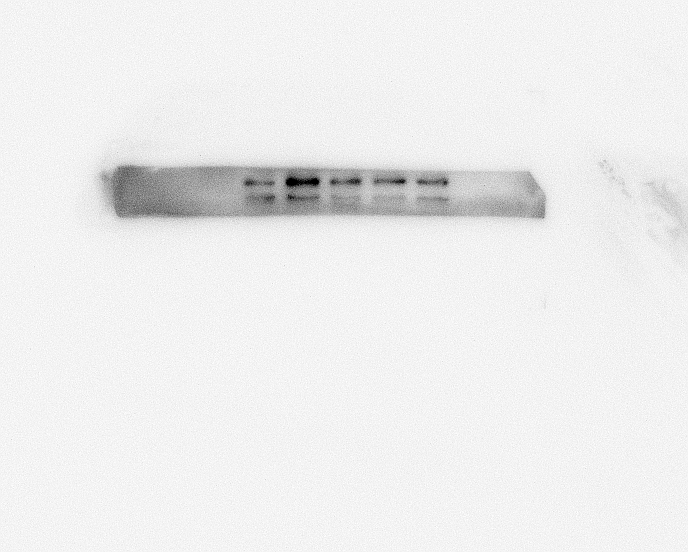

Supplement: Supplementary file 1 [file DataSheet3.zip › Fig.2/WBμ¥íσ╕a/IL-18/1-IL18.tif]

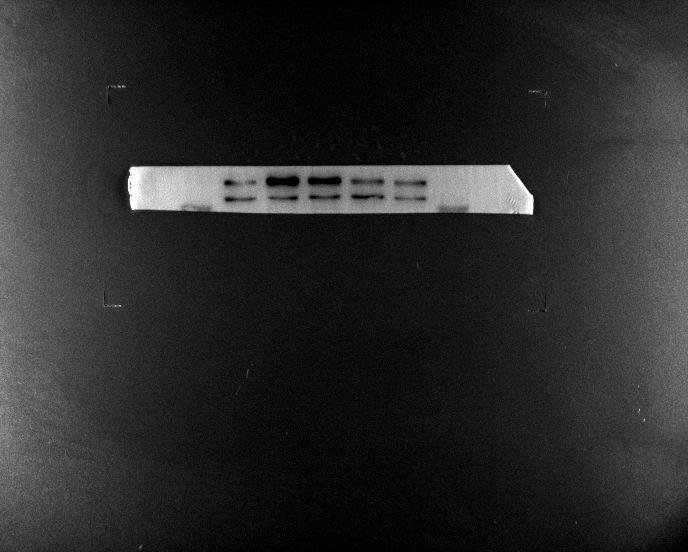

Supplement: Supplementary file 1 [file DataSheet3.zip › Fig.2/WBμ¥íσ╕a/IL-18/2-IL18 YT.tif]

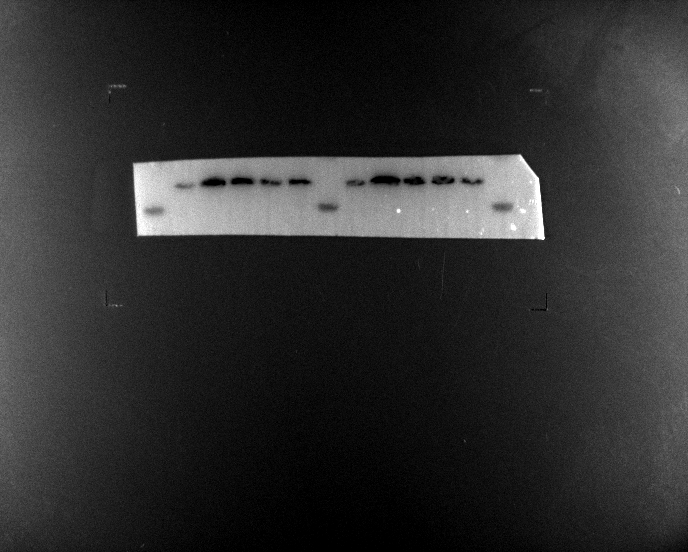

Supplement: Supplementary file 1 [file DataSheet3.zip › Fig.2/WBμ¥íσ╕a/GSDMD/1-2-GSDMD-N YT.tif]

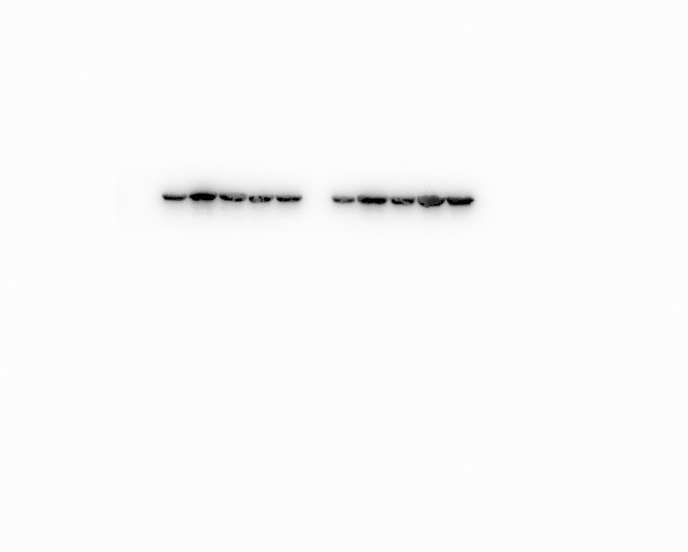

Supplement: Supplementary file 1 [file DataSheet3.zip › Fig.2/WBμ¥íσ╕a/GSDMD/3-GSDMD-N.tif]

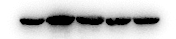

Supplement: Supplementary file 1 [file DataSheet3.zip › Fig.2/WBμ¥íσ╕a/GSDMD/3.tif]

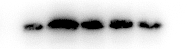

Supplement: Supplementary file 1 [file DataSheet3.zip › Fig.2/WBμ¥íσ╕a/GSDMD/2.tif]

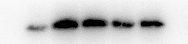

Supplement: Supplementary file 1 [file DataSheet3.zip › Fig.2/WBμ¥íσ╕a/GSDMD/1.tif]

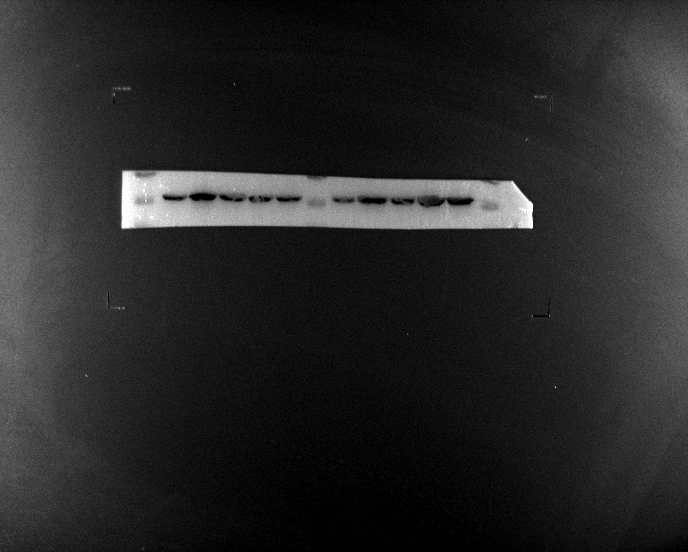

Supplement: Supplementary file 1 [file DataSheet3.zip › Fig.2/WBμ¥íσ╕a/GSDMD/3-GSDMD-N YT.tif]

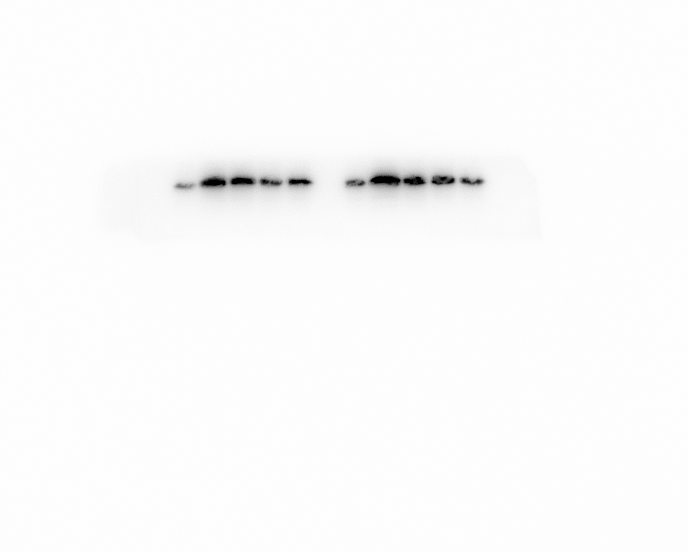

Supplement: Supplementary file 1 [file DataSheet3.zip › Fig.2/WBμ¥íσ╕a/GSDMD/1-2-GSDMD-N.tif]

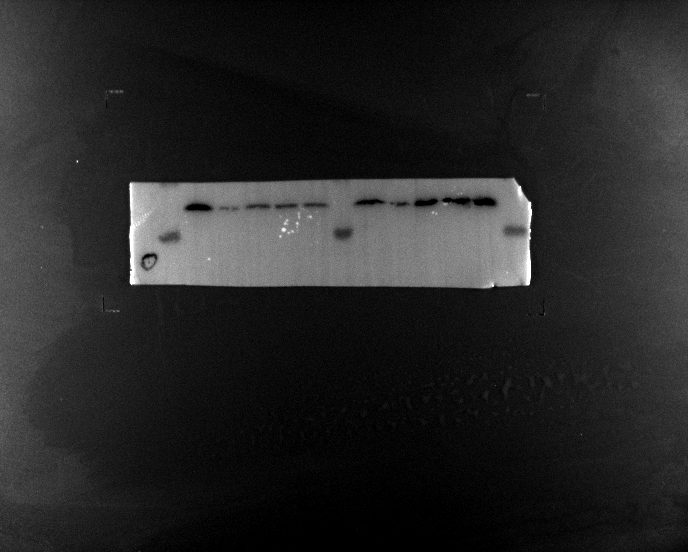

Supplement: Supplementary file 1 [file DataSheet3.zip › Fig.2/WBμ¥íσ╕a/ZO-1/3-4-ZO1 YT.tif]

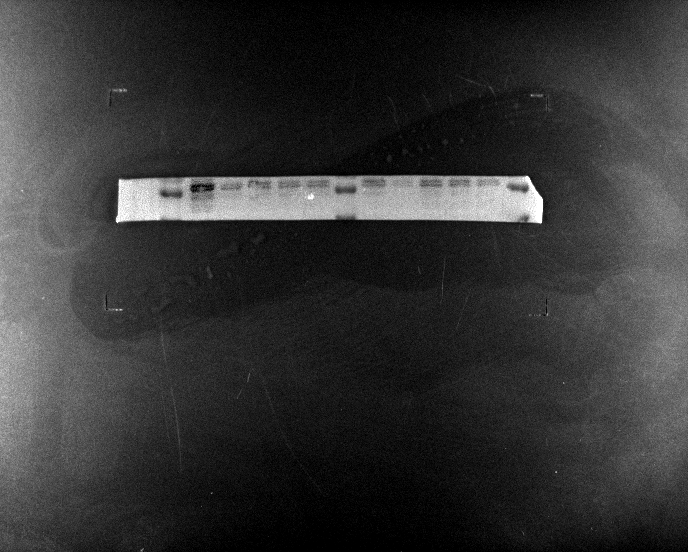

Supplement: Supplementary file 1 [file DataSheet3.zip › Fig.2/WBμ¥íσ╕a/ZO-1/2-ZO1 YT.tif]

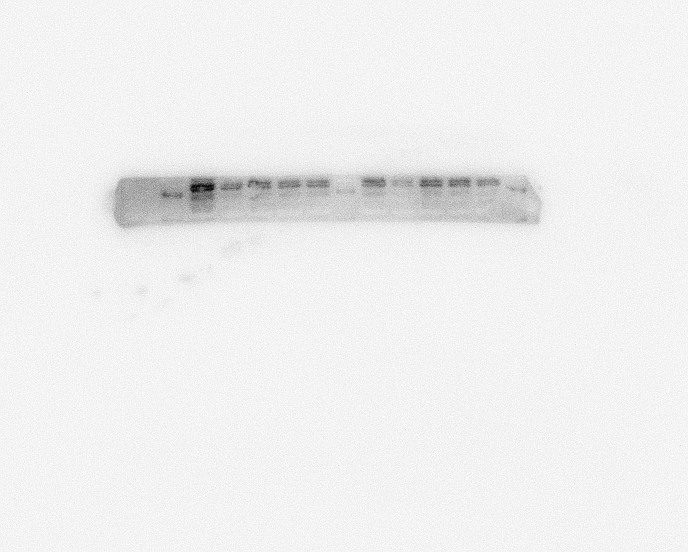

Supplement: Supplementary file 1 [file DataSheet3.zip › Fig.2/WBμ¥íσ╕a/ZO-1/2-ZO1.tif]

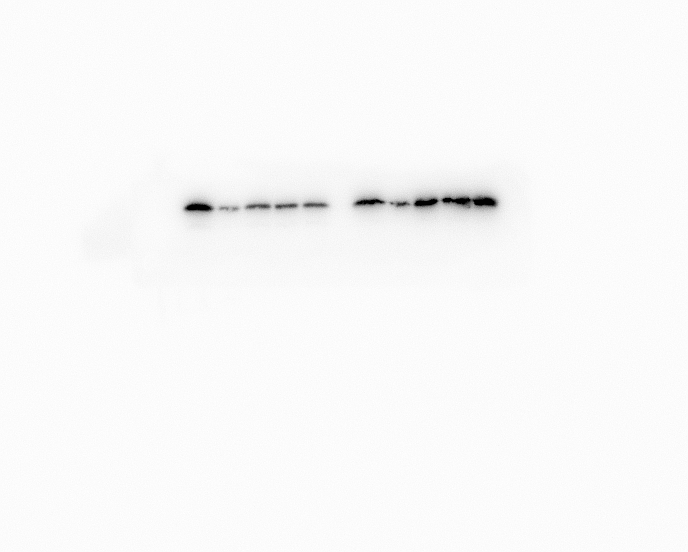

Supplement: Supplementary file 1 [file DataSheet3.zip › Fig.2/WBμ¥íσ╕a/ZO-1/3-4-ZO1.tif]

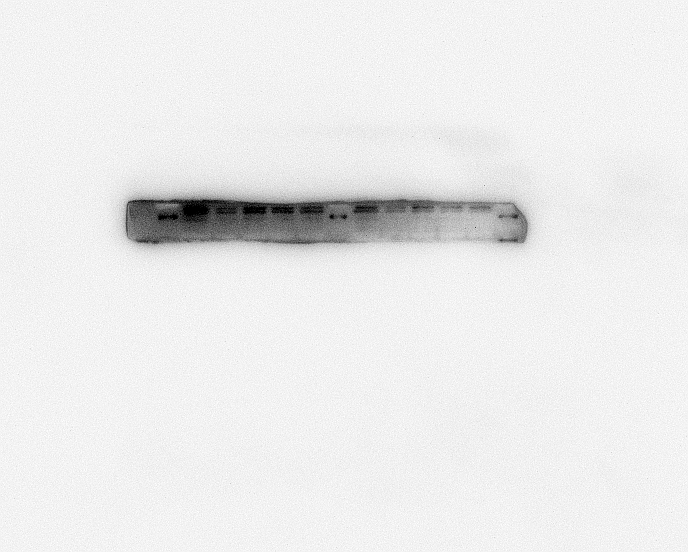

Supplement: Supplementary file 1 [file DataSheet3.zip › Fig.2/WBμ¥íσ╕a/ZO-1/1-ZO1.tif]

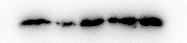

Supplement: Supplementary file 1 [file DataSheet3.zip › Fig.2/WBμ¥íσ╕a/ZO-1/3.tif]

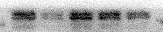

Supplement: Supplementary file 1 [file DataSheet3.zip › Fig.2/WBμ¥íσ╕a/ZO-1/2.tif]

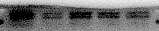

Supplement: Supplementary file 1 [file DataSheet3.zip › Fig.2/WBμ¥íσ╕a/ZO-1/1.tif]

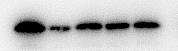

Supplement: Supplementary file 1 [file DataSheet3.zip › Fig.2/WBμ¥íσ╕a/ZO-1/4.tif]

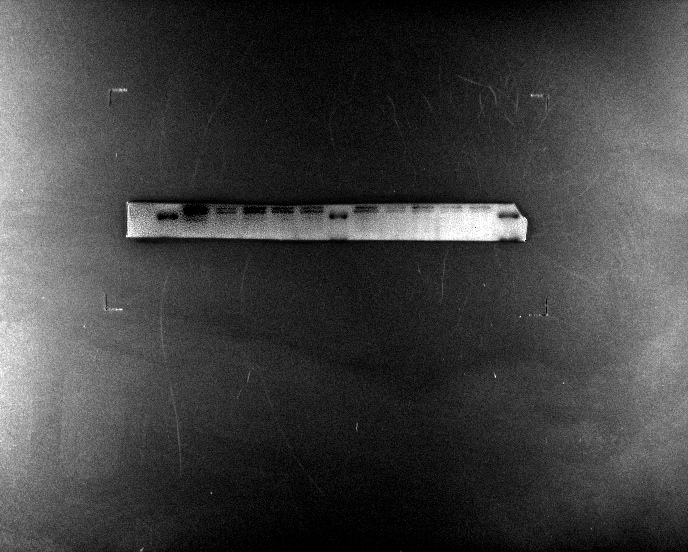

Supplement: Supplementary file 1 [file DataSheet3.zip › Fig.2/WBμ¥íσ╕a/ZO-1/1-ZO1 YT.tif]

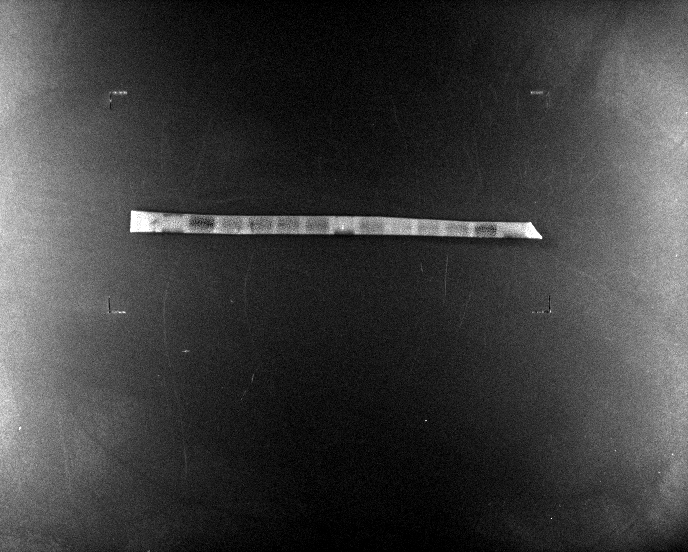

Supplement: Supplementary file 1 [file DataSheet3.zip › Fig.2/WBμ¥íσ╕a/Nephrin/2-Nephrin YT.tif]

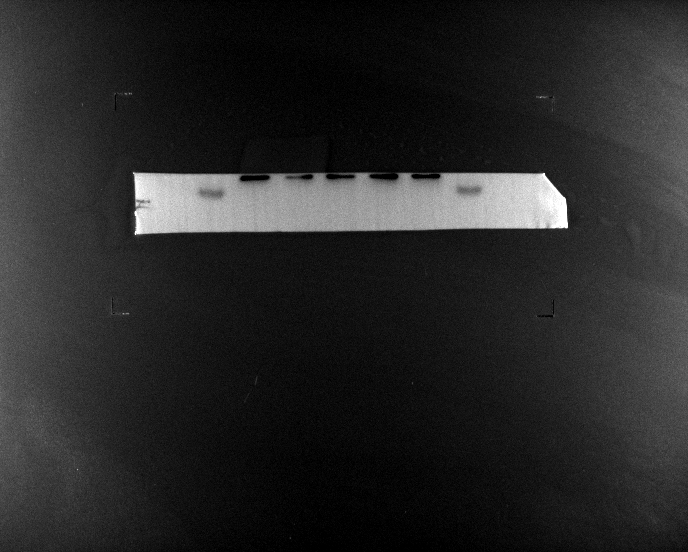

Supplement: Supplementary file 1 [file DataSheet3.zip › Fig.2/WBμ¥íσ╕a/Nephrin/3-Nephrin YT.tif]

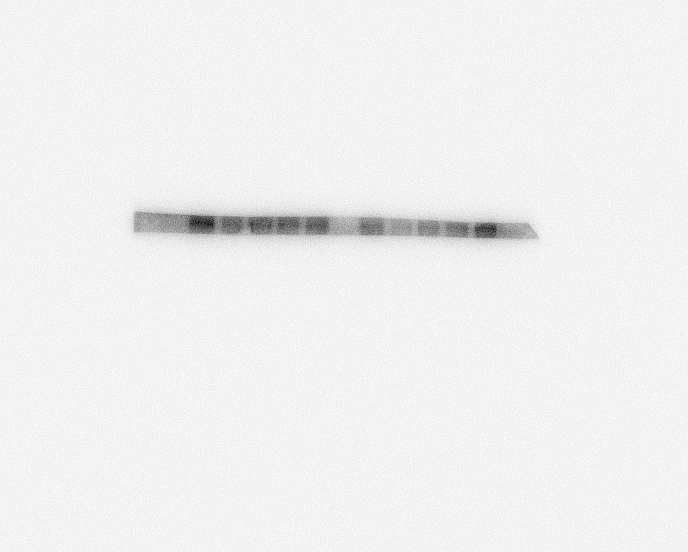

Supplement: Supplementary file 1 [file DataSheet3.zip › Fig.2/WBμ¥íσ╕a/Nephrin/2-Nephrin.tif]

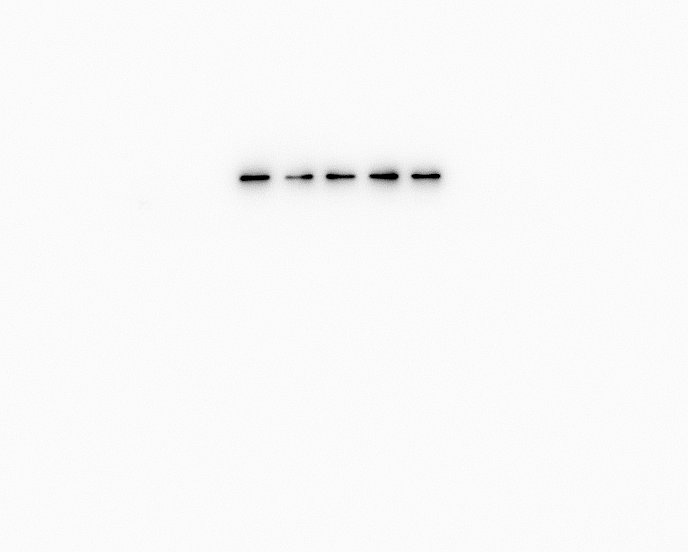

Supplement: Supplementary file 1 [file DataSheet3.zip › Fig.2/WBμ¥íσ╕a/Nephrin/3- Nephrin.tif]

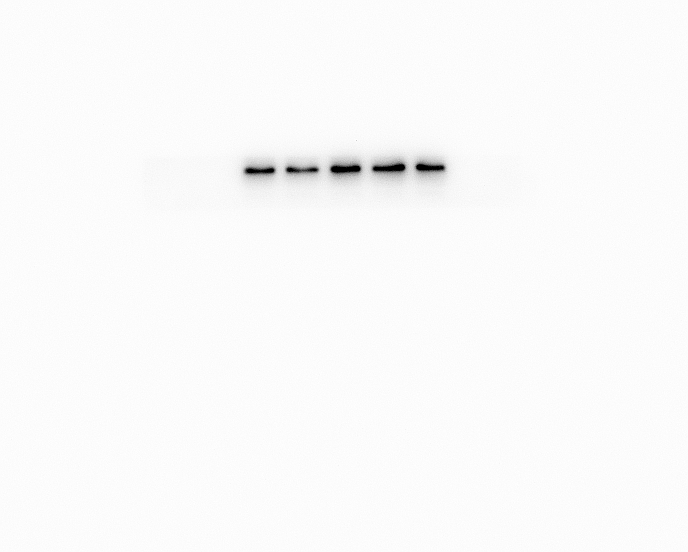

Supplement: Supplementary file 1 [file DataSheet3.zip › Fig.2/WBμ¥íσ╕a/Nephrin/1-Nephrin.tif]

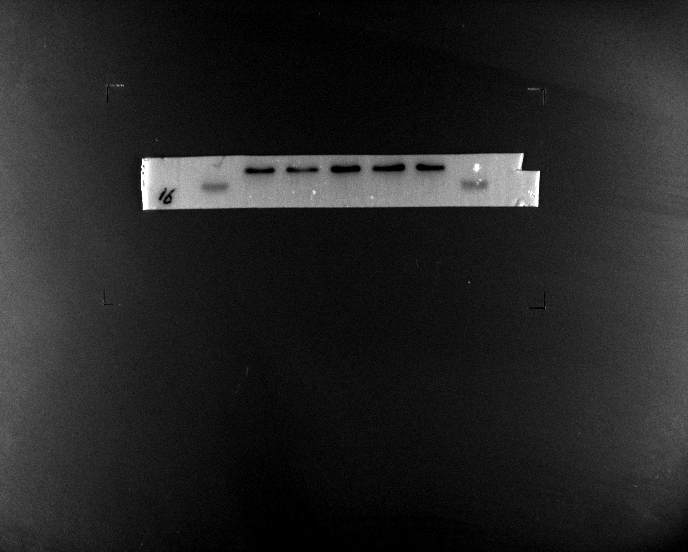

Supplement: Supplementary file 1 [file DataSheet3.zip › Fig.2/WBμ¥íσ╕a/Nephrin/1-Nephrin YT.tif]

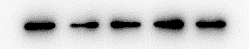

Supplement: Supplementary file 1 [file DataSheet3.zip › Fig.2/WBμ¥íσ╕a/Nephrin/3.tif]

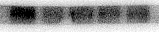

Supplement: Supplementary file 1 [file DataSheet3.zip › Fig.2/WBμ¥íσ╕a/Nephrin/2.tif]

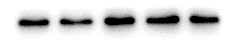

Supplement: Supplementary file 1 [file DataSheet3.zip › Fig.2/WBμ¥íσ╕a/Nephrin/1.tif]

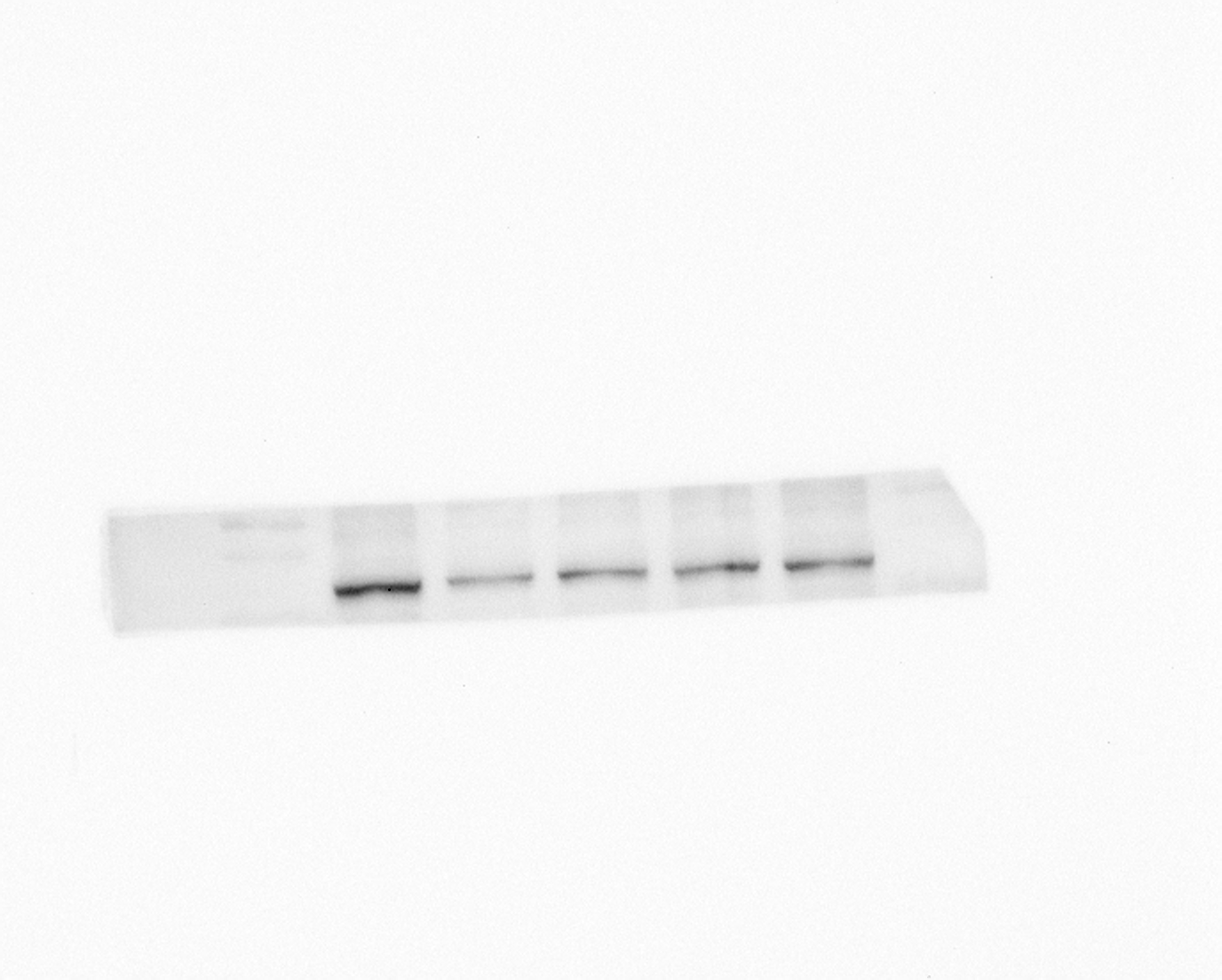

Supplement: Supplementary file 1 [file DataSheet3.zip › Fig.2/WBμ¥íσ╕a/WT1/3-WT1-30s.Tif]

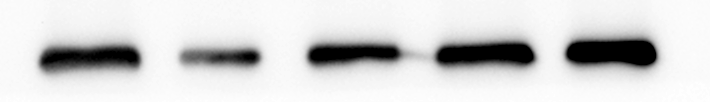

Supplement: Supplementary file 1 [file DataSheet3.zip › Fig.2/WBμ¥íσ╕a/WT1/PS-2-WT1-30s.tif]

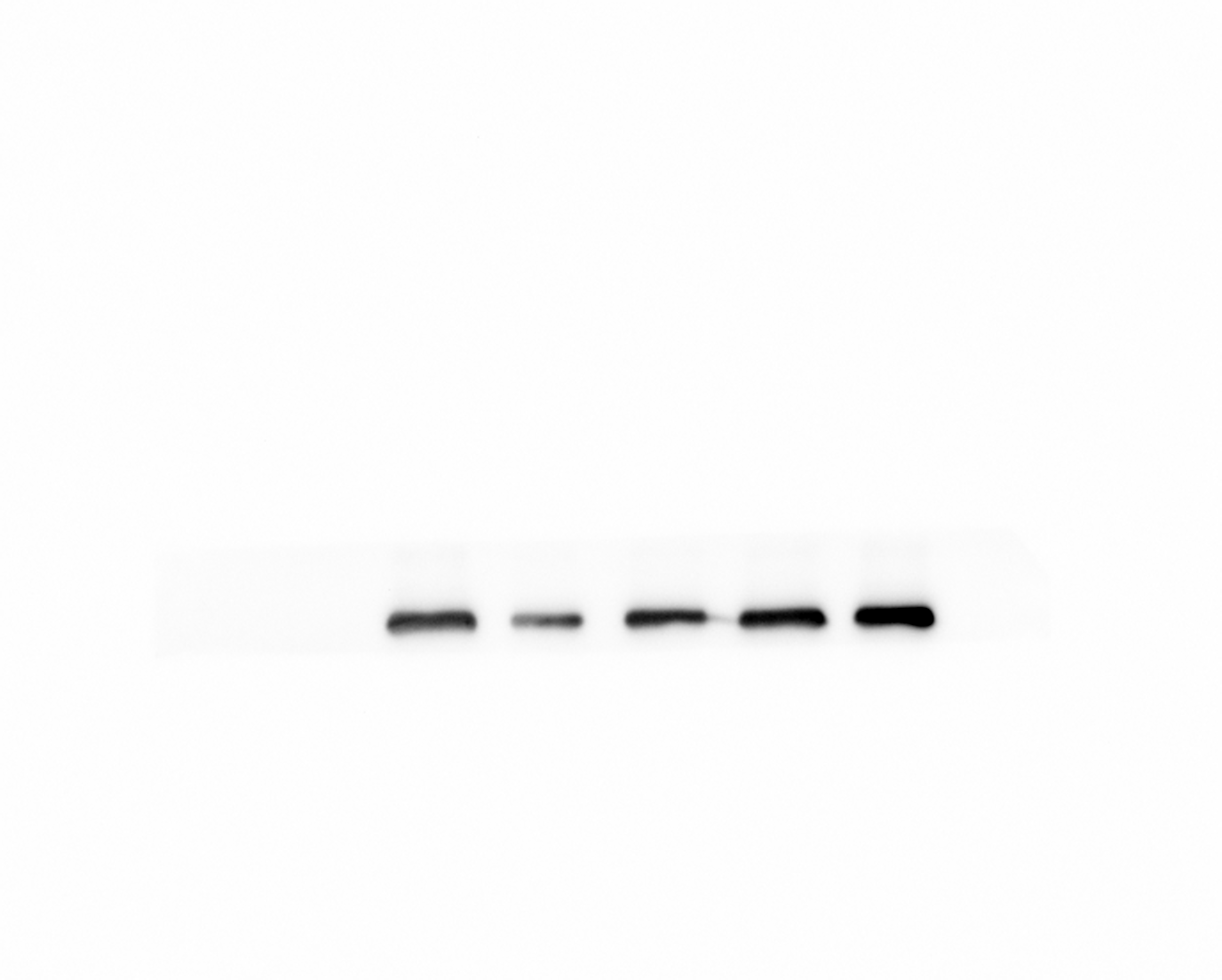

Supplement: Supplementary file 1 [file DataSheet3.zip › Fig.2/WBμ¥íσ╕a/WT1/2-WT1-30s.Tif]

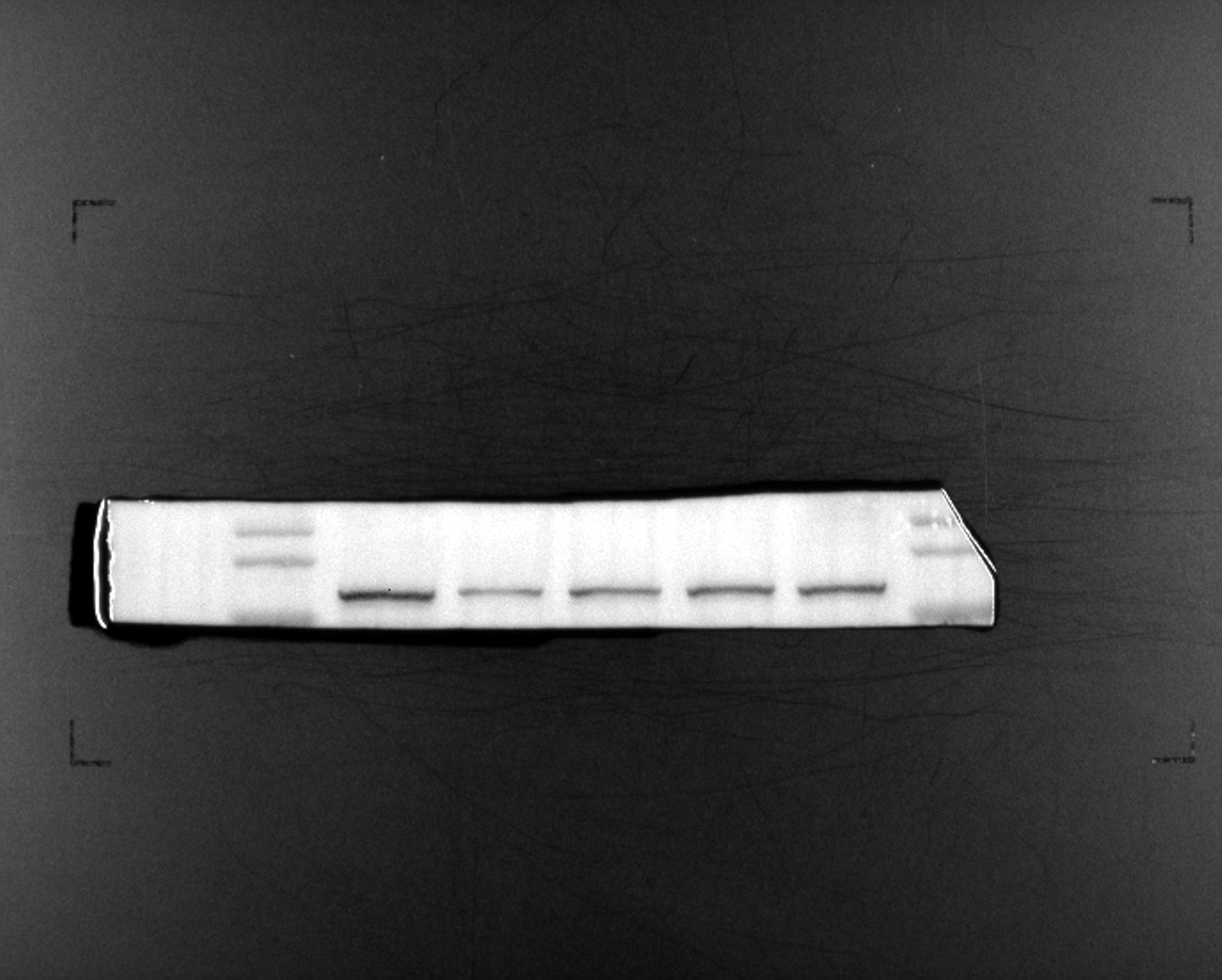

Supplement: Supplementary file 1 [file DataSheet3.zip › Fig.2/WBμ¥íσ╕a/WT1/1-WT1-30s YT.Tif]

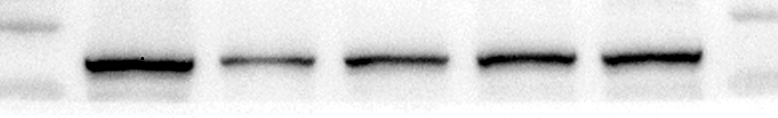

Supplement: Supplementary file 1 [file DataSheet3.zip › Fig.2/WBμ¥íσ╕a/WT1/τö¿-PS 1-WT1-30s.tif]

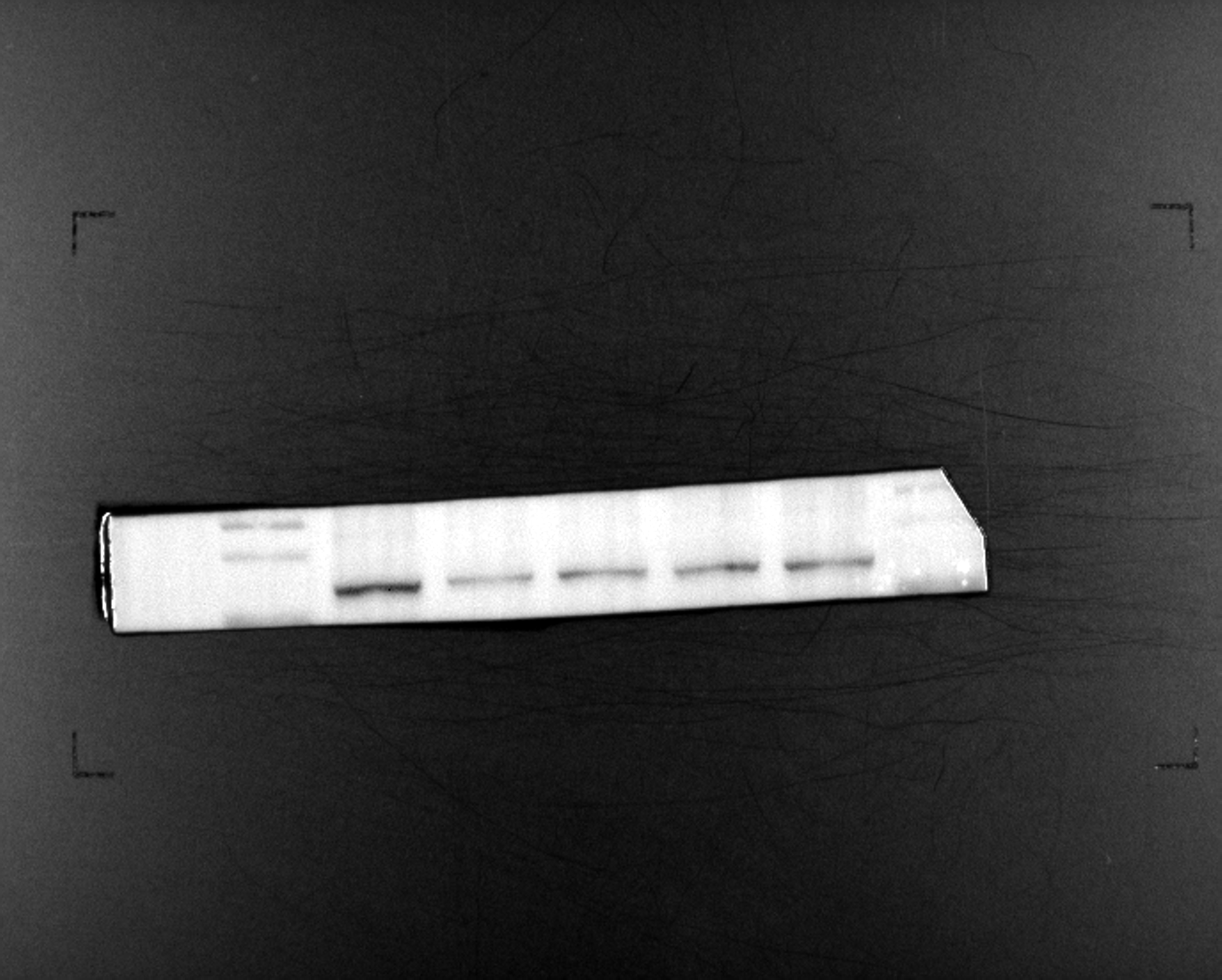

Supplement: Supplementary file 1 [file DataSheet3.zip › Fig.2/WBμ¥íσ╕a/WT1/3-WT1-30s YT.Tif]

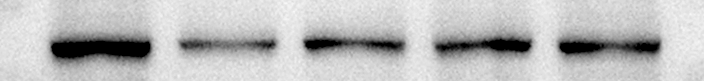

Supplement: Supplementary file 1 [file DataSheet3.zip › Fig.2/WBμ¥íσ╕a/WT1/PS-3-WT1-30s.tif]

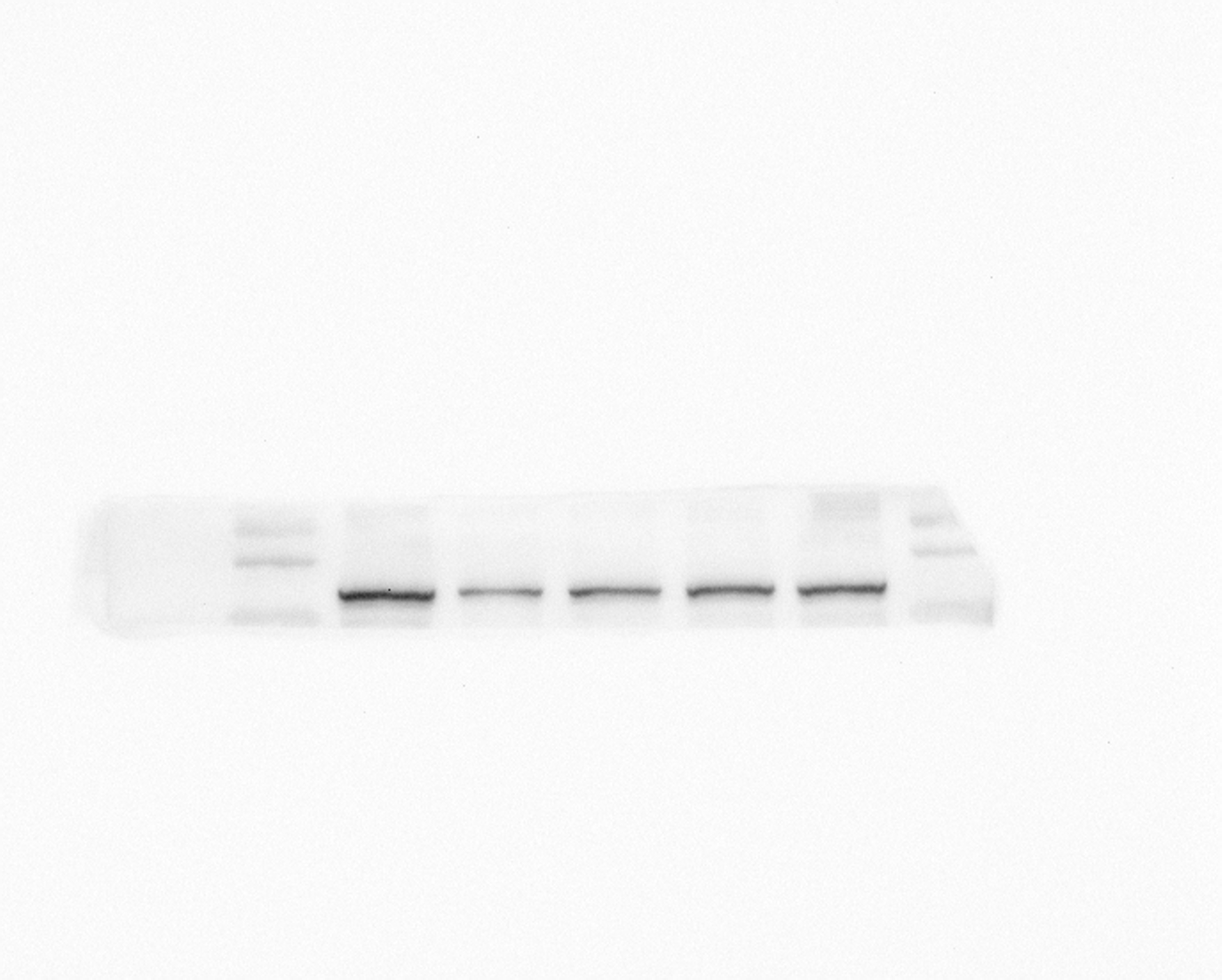

Supplement: Supplementary file 1 [file DataSheet3.zip › Fig.2/WBμ¥íσ╕a/WT1/1-WT1-30s.Tif]

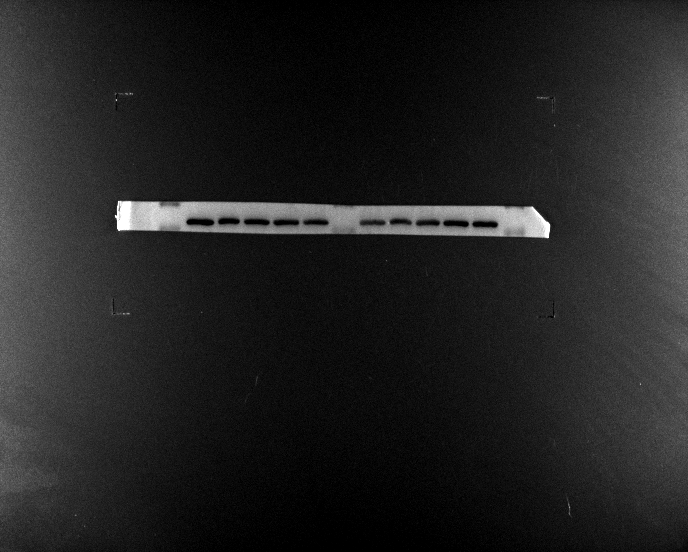

Supplement: Supplementary file 1 [file DataSheet3.zip › Fig.2/WBμ¥íσ╕a/GAPDH/2-3-GAPDH YT.tif]

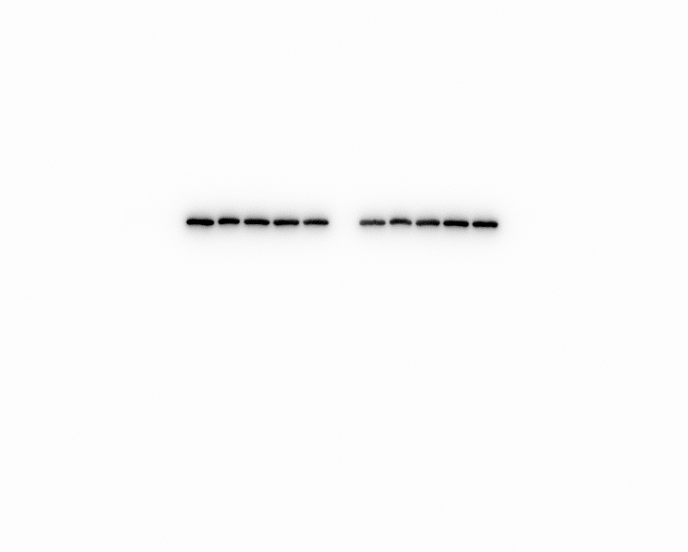

Supplement: Supplementary file 1 [file DataSheet3.zip › Fig.2/WBμ¥íσ╕a/GAPDH/2-3-GAPDH.tif]

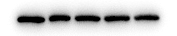

Supplement: Supplementary file 1 [file DataSheet3.zip › Fig.2/WBμ¥íσ╕a/GAPDH/3.tif]

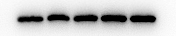

Supplement: Supplementary file 1 [file DataSheet3.zip › Fig.2/WBμ¥íσ╕a/GAPDH/2.tif]

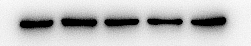

Supplement: Supplementary file 1 [file DataSheet3.zip › Fig.2/WBμ¥íσ╕a/GAPDH/1.tif]

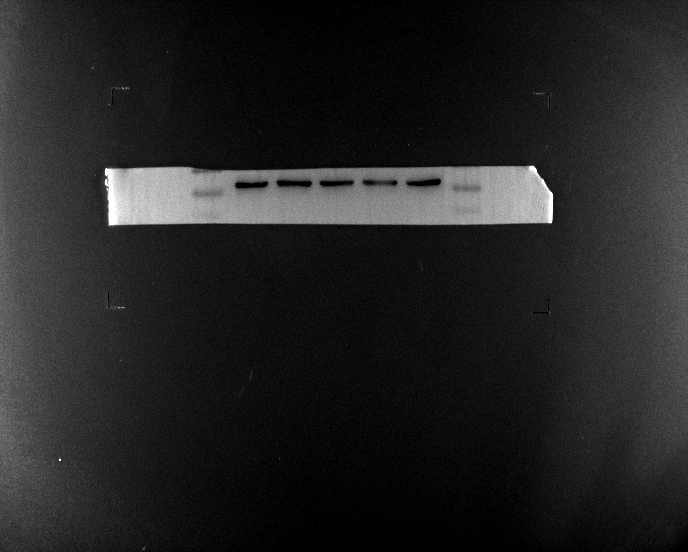

Supplement: Supplementary file 1 [file DataSheet3.zip › Fig.2/WBμ¥íσ╕a/GAPDH/1-GAPDH YT.tif]

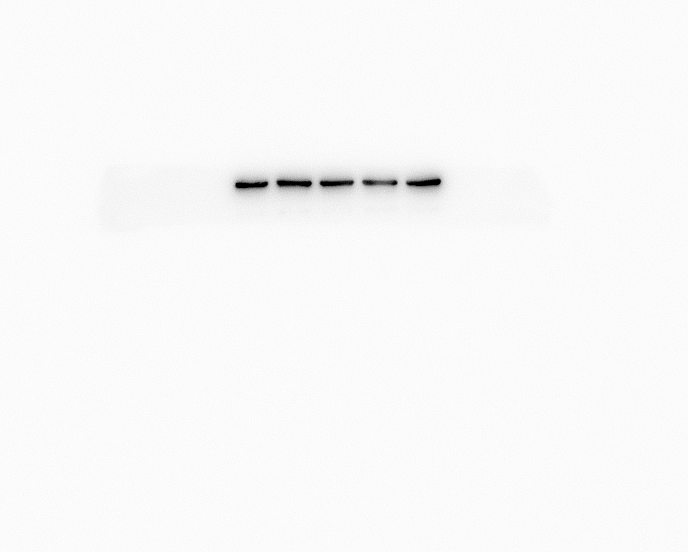

Supplement: Supplementary file 1 [file DataSheet3.zip › Fig.2/WBμ¥íσ╕a/GAPDH/1-GAPDH.tif]

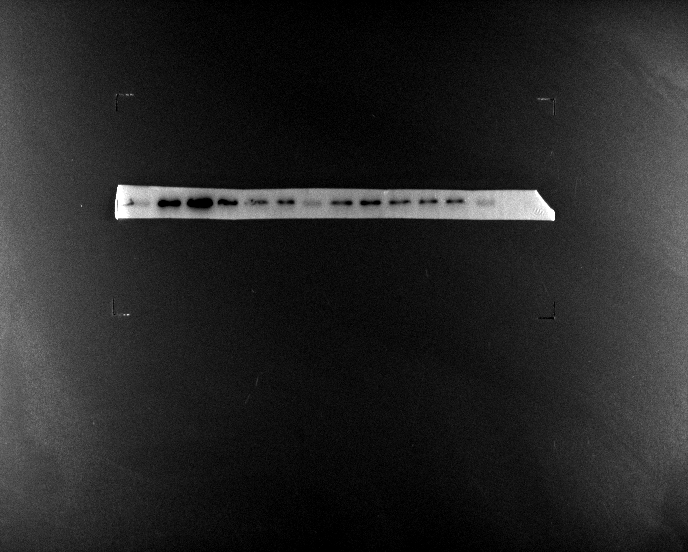

Supplement: Supplementary file 1 [file DataSheet3.zip › Fig.2/WBμ¥íσ╕a/IL-1/2-3-IL1 YT.tif]

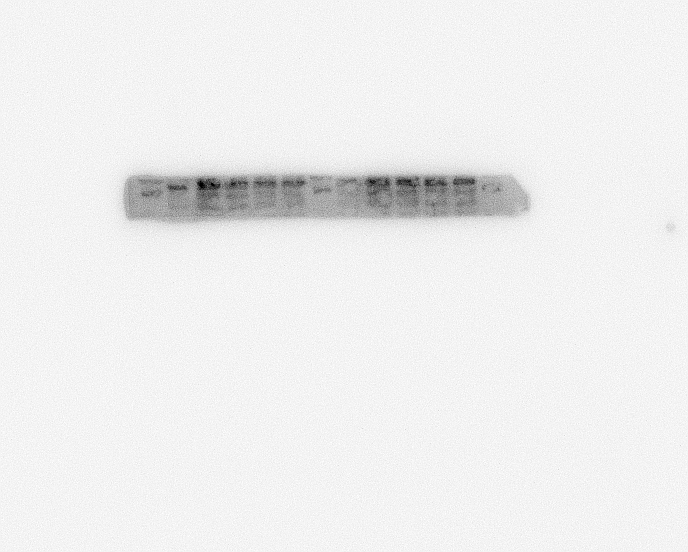

Supplement: Supplementary file 1 [file DataSheet3.zip › Fig.2/WBμ¥íσ╕a/IL-1/1-IL1.tif]

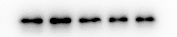

Supplement: Supplementary file 1 [file DataSheet3.zip › Fig.2/WBμ¥íσ╕a/IL-1/3.tif]

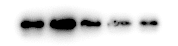

Supplement: Supplementary file 1 [file DataSheet3.zip › Fig.2/WBμ¥íσ╕a/IL-1/2.tif]

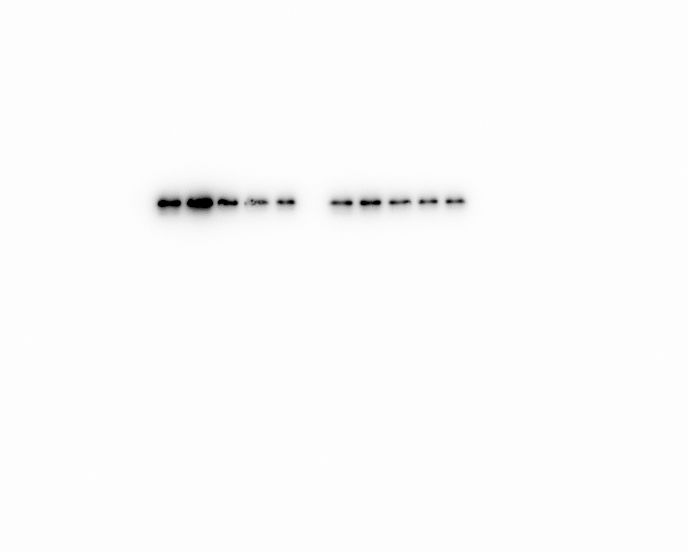

Supplement: Supplementary file 1 [file DataSheet3.zip › Fig.2/WBμ¥íσ╕a/IL-1/2-3-IL1.tif]

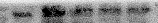

Supplement: Supplementary file 1 [file DataSheet3.zip › Fig.2/WBμ¥íσ╕a/IL-1/1.tif]

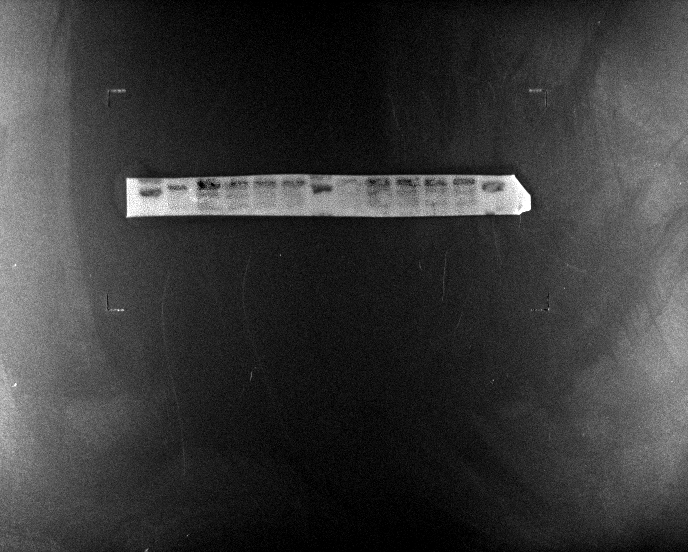

Supplement: Supplementary file 1 [file DataSheet3.zip › Fig.2/WBμ¥íσ╕a/IL-1/1-IL1 YT.tif]

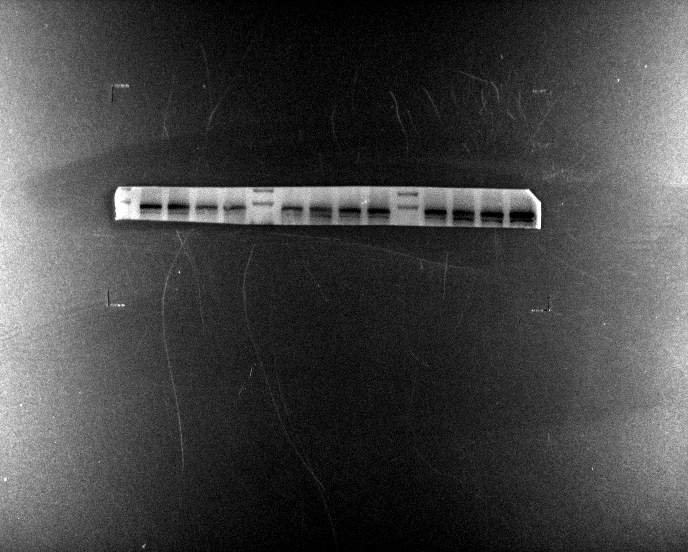

Supplement: Supplementary file 1 [file DataSheet3.zip › Fig.3/ASC/3-ASC YT.tif]

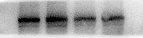

Supplement: Supplementary file 1 [file DataSheet3.zip › Fig.3/ASC/3.tif]

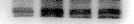

Supplement: Supplementary file 1 [file DataSheet3.zip › Fig.3/ASC/2.tif]

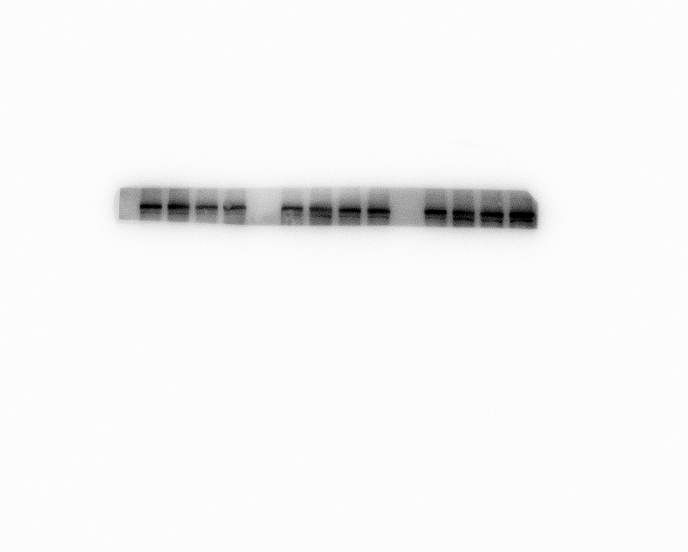

Supplement: Supplementary file 1 [file DataSheet3.zip › Fig.3/ASC/3-ASC.tif]

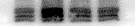

Supplement: Supplementary file 1 [file DataSheet3.zip › Fig.3/ASC/1.tif]

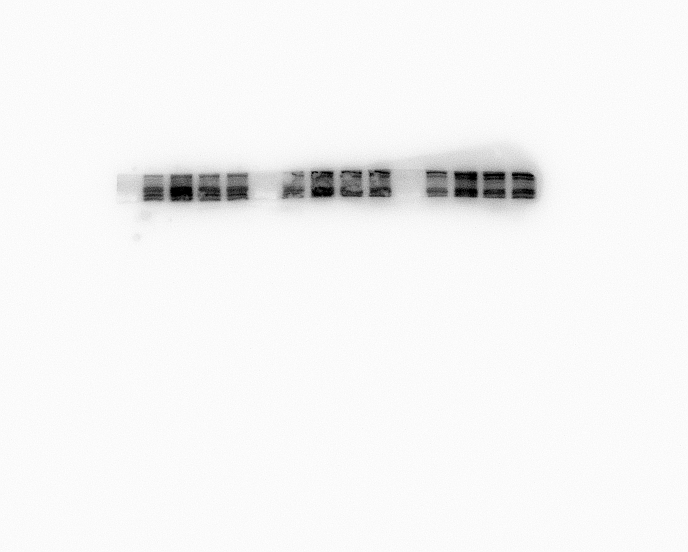

Supplement: Supplementary file 1 [file DataSheet3.zip › Fig.3/ASC/1-2-ASC.tif]

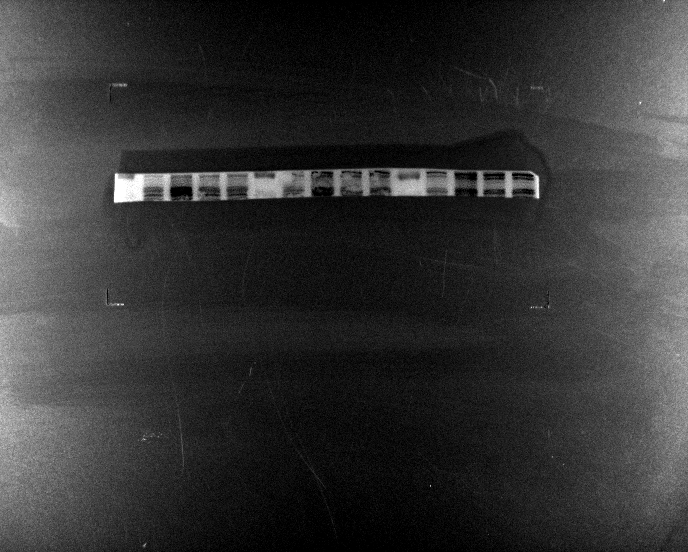

Supplement: Supplementary file 1 [file DataSheet3.zip › Fig.3/ASC/1-2-ASC YT.tif]

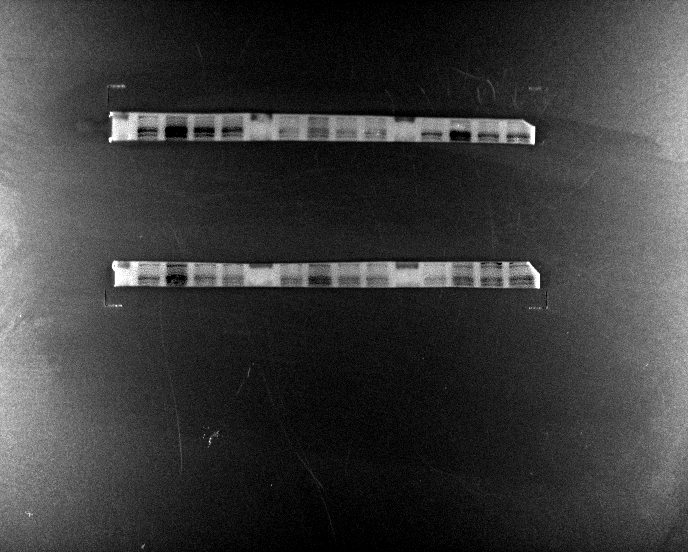

Supplement: Supplementary file 1 [file DataSheet3.zip › Fig.3/NLRP3/NLRP3 YT.tif]

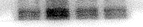

Supplement: Supplementary file 1 [file DataSheet3.zip › Fig.3/NLRP3/3.tif]

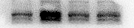

Supplement: Supplementary file 1 [file DataSheet3.zip › Fig.3/NLRP3/2.tif]

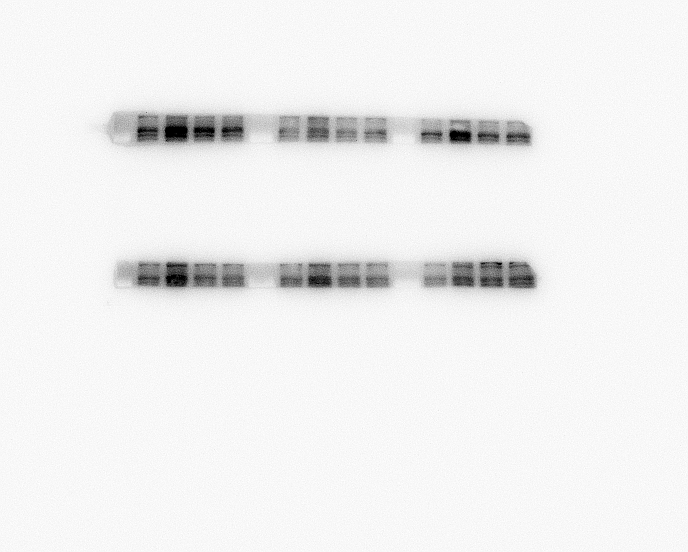

Supplement: Supplementary file 1 [file DataSheet3.zip › Fig.3/NLRP3/NLRP3.tif]

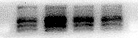

Supplement: Supplementary file 1 [file DataSheet3.zip › Fig.3/NLRP3/1.tif]

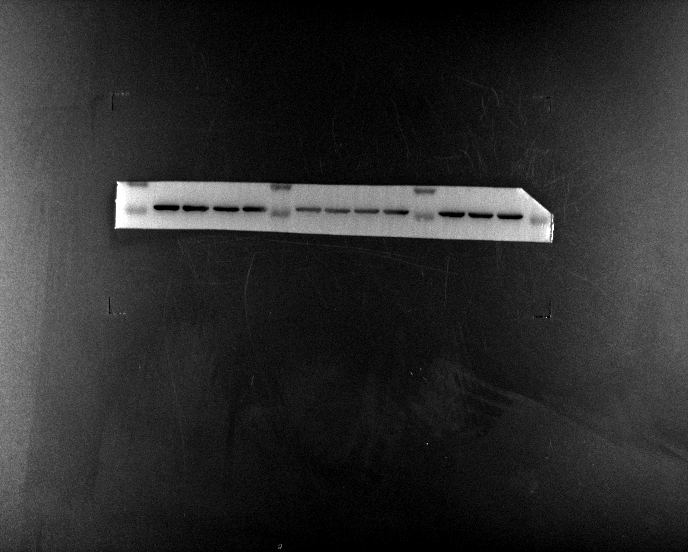

Supplement: Supplementary file 1 [file DataSheet3.zip › Fig.3/A-GAPDH/1-2-GAPDH YT.tif]

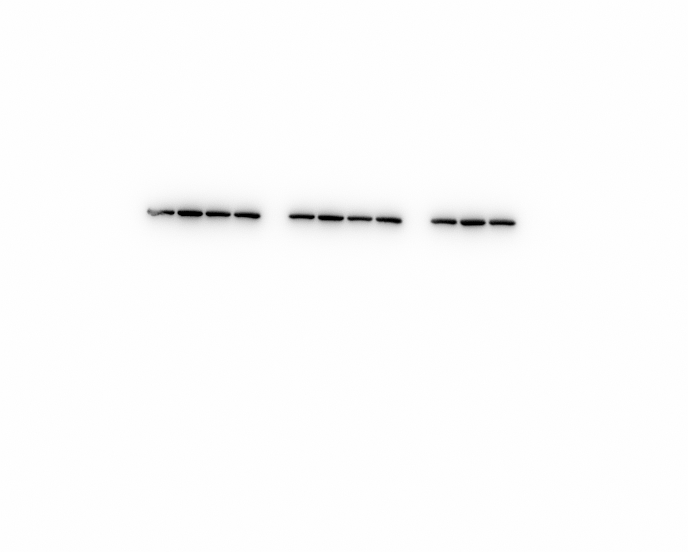

Supplement: Supplementary file 1 [file DataSheet3.zip › Fig.3/A-GAPDH/3-GAPDH.tif]

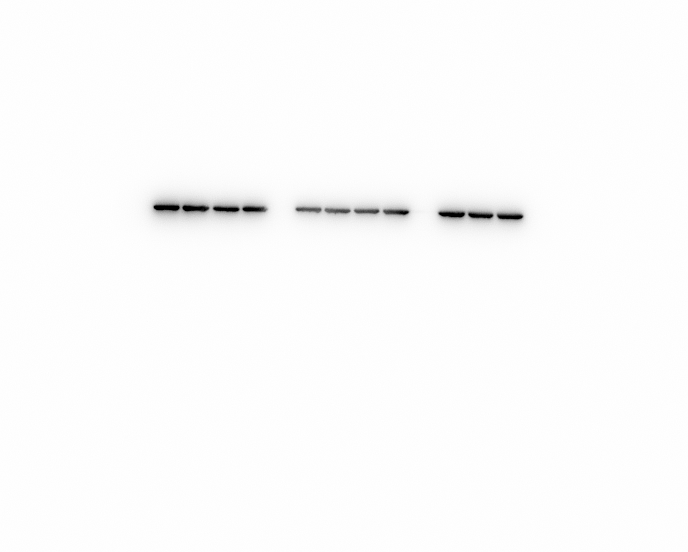

Supplement: Supplementary file 1 [file DataSheet3.zip › Fig.3/A-GAPDH/1-2-GAPDH.tif]

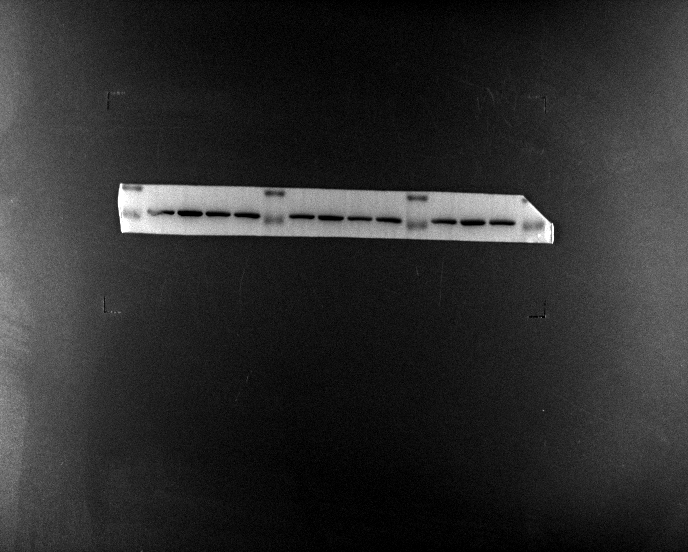

Supplement: Supplementary file 1 [file DataSheet3.zip › Fig.3/A-GAPDH/3-GAPDH YT.tif]

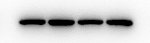

Supplement: Supplementary file 1 [file DataSheet3.zip › Fig.3/A-GAPDH/3.tif]

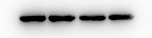

Supplement: Supplementary file 1 [file DataSheet3.zip › Fig.3/A-GAPDH/2.tif]

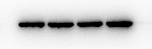

Supplement: Supplementary file 1 [file DataSheet3.zip › Fig.3/A-GAPDH/1.tif]

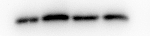

Supplement: Supplementary file 1 [file DataSheet3.zip › Fig.3/NEK7/3.tif]

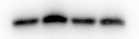

Supplement: Supplementary file 1 [file DataSheet3.zip › Fig.3/NEK7/2.tif]

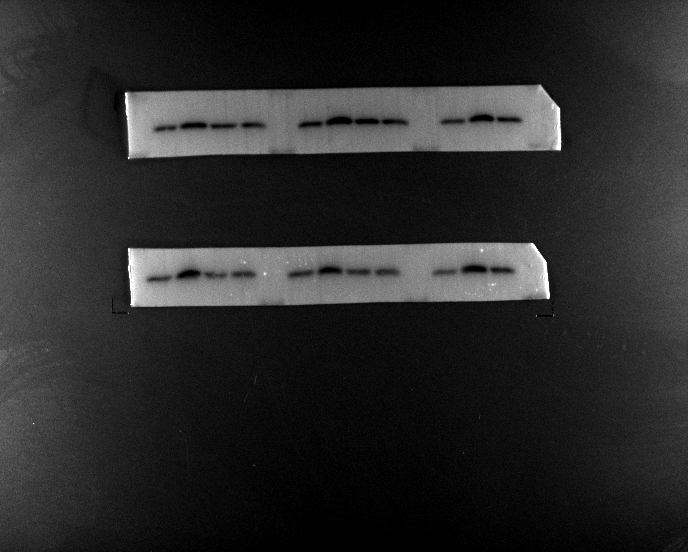

Supplement: Supplementary file 1 [file DataSheet3.zip › Fig.3/NEK7/NEK7 YT.tif]

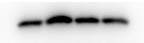

Supplement: Supplementary file 1 [file DataSheet3.zip › Fig.3/NEK7/1.tif]

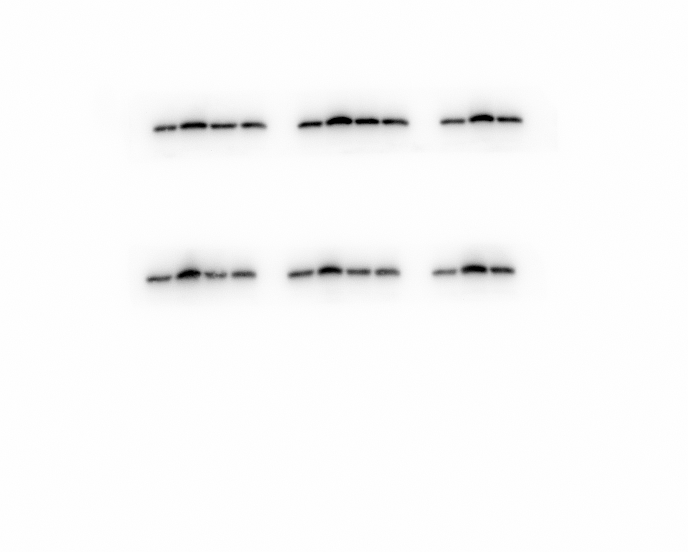

Supplement: Supplementary file 1 [file DataSheet3.zip › Fig.3/NEK7/NEK7.tif]

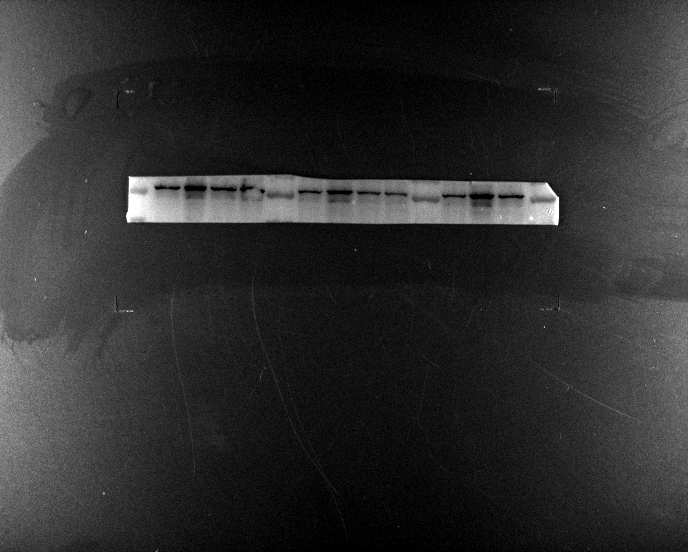

Supplement: Supplementary file 1 [file DataSheet3.zip › Fig.3/Caspase-1/1-2-Caspase1 YT.tif]

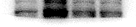

Supplement: Supplementary file 1 [file DataSheet3.zip › Fig.3/Caspase-1/3.tif]

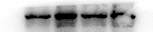

Supplement: Supplementary file 1 [file DataSheet3.zip › Fig.3/Caspase-1/2.tif]

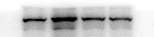

Supplement: Supplementary file 1 [file DataSheet3.zip › Fig.3/Caspase-1/1.tif]

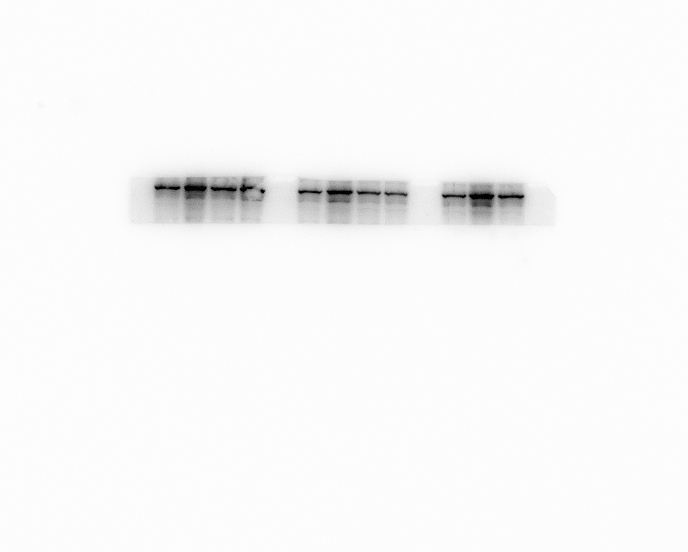

Supplement: Supplementary file 1 [file DataSheet3.zip › Fig.3/Caspase-1/1-2-Caspase1.tif]

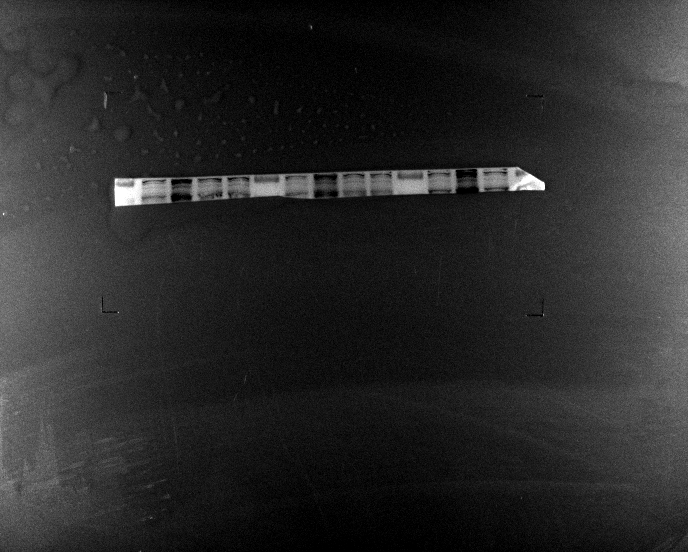

Supplement: Supplementary file 1 [file DataSheet3.zip › Fig.3/Caspase-1/3-Caspase1 YT.tif]

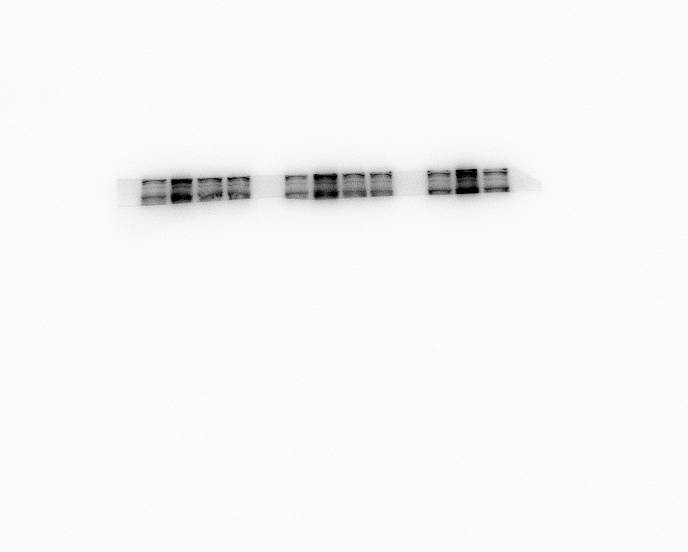

Supplement: Supplementary file 1 [file DataSheet3.zip › Fig.3/Caspase-1/3-Caspase1.tif]

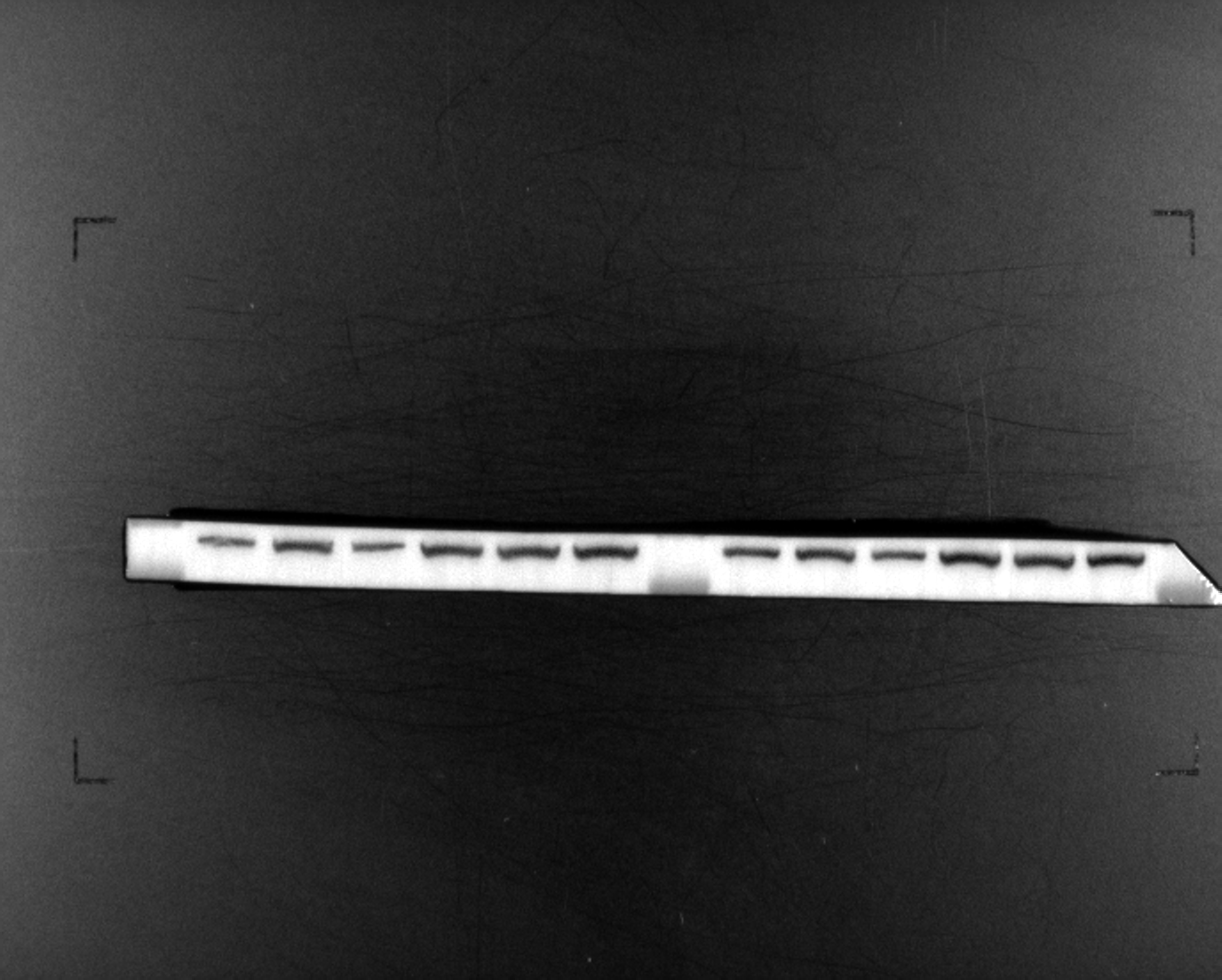

Supplement: Supplementary file 2 [file DataSheet8.zip › Fig.11/1-GSDMD/1-GSDMD-10S YT.Tif]

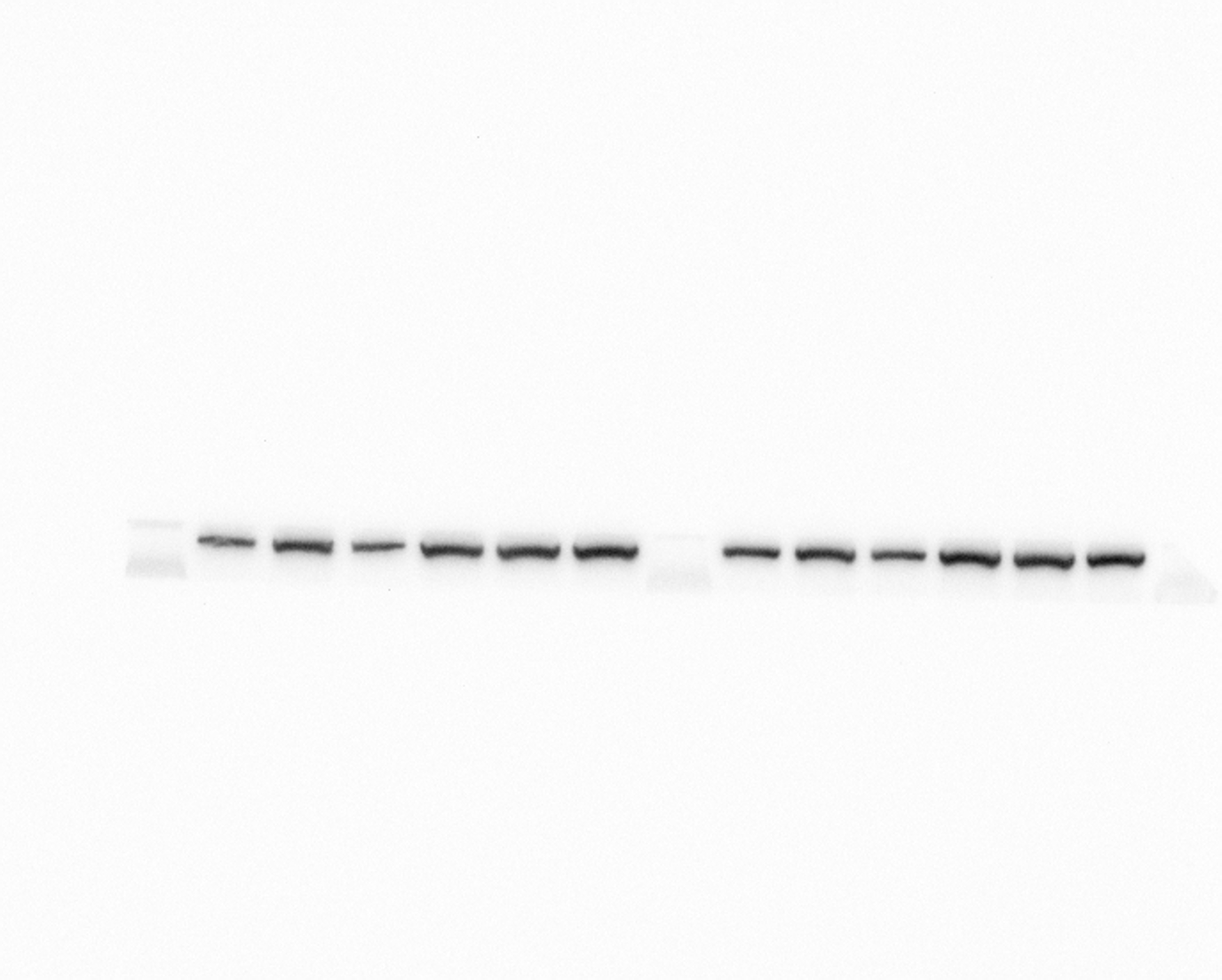

Supplement: Supplementary file 2 [file DataSheet8.zip › Fig.11/1-GSDMD/1-GSDMD-10S.Tif]
