# Supplementary figures and images for: Total Flavones of Abelmoschus manihot Ameliorates Podocyte Pyroptosis and Injury in High Glucose Conditions by Targeting METTL3-Dependent m6A Modification-Mediated NLRP3-Inflammasome Activation and PTEN/PI3K/Akt Signaling (part 2 of 6)
Source: Front Pharmacol. 2021 Jul 15;12:667644. doi: 10.3389/fphar.2021.667644 (PMC8319635; doi:10.3389/fphar.2021.667644)

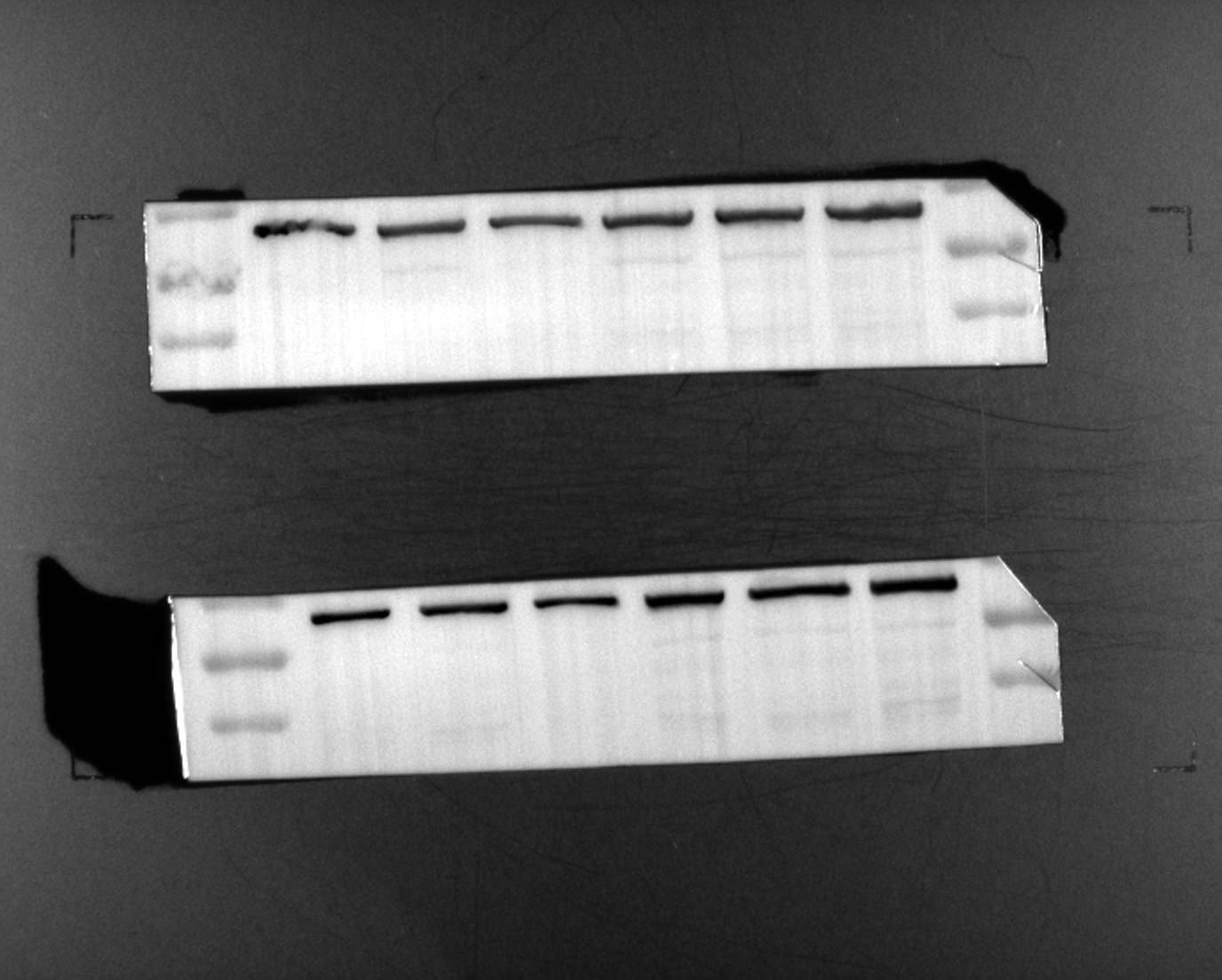

Supplement: Supplementary file 2 [file DataSheet8.zip › Fig.11/1-GSDMD/2-GSDMD-10S YT.Tif]

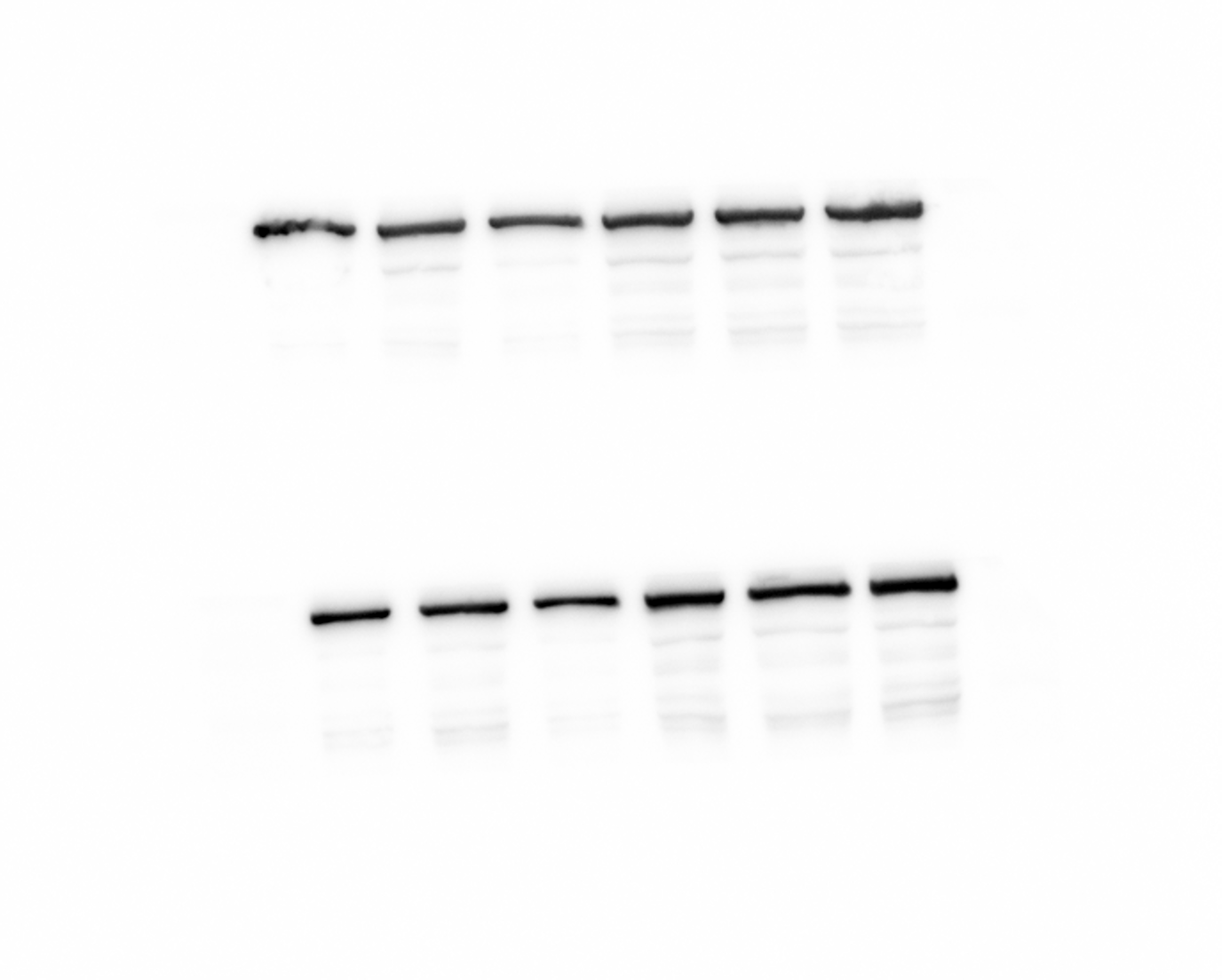

Supplement: Supplementary file 2 [file DataSheet8.zip › Fig.11/1-GSDMD/2-GSDMD-10S.Tif]

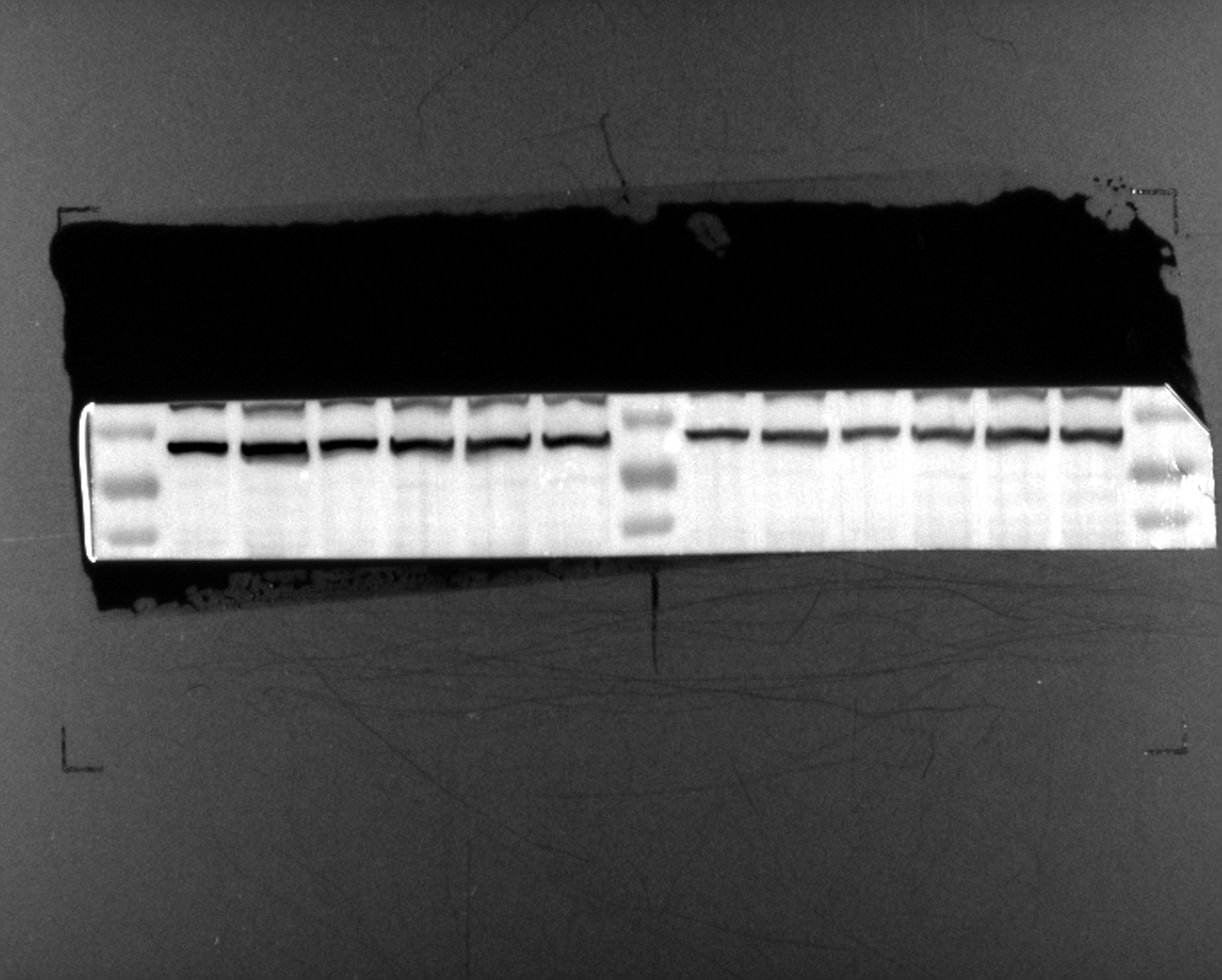

Supplement: Supplementary file 2 [file DataSheet8.zip › Fig.11/1-GSDMD/3-GSDMD-10S YT.Tif]

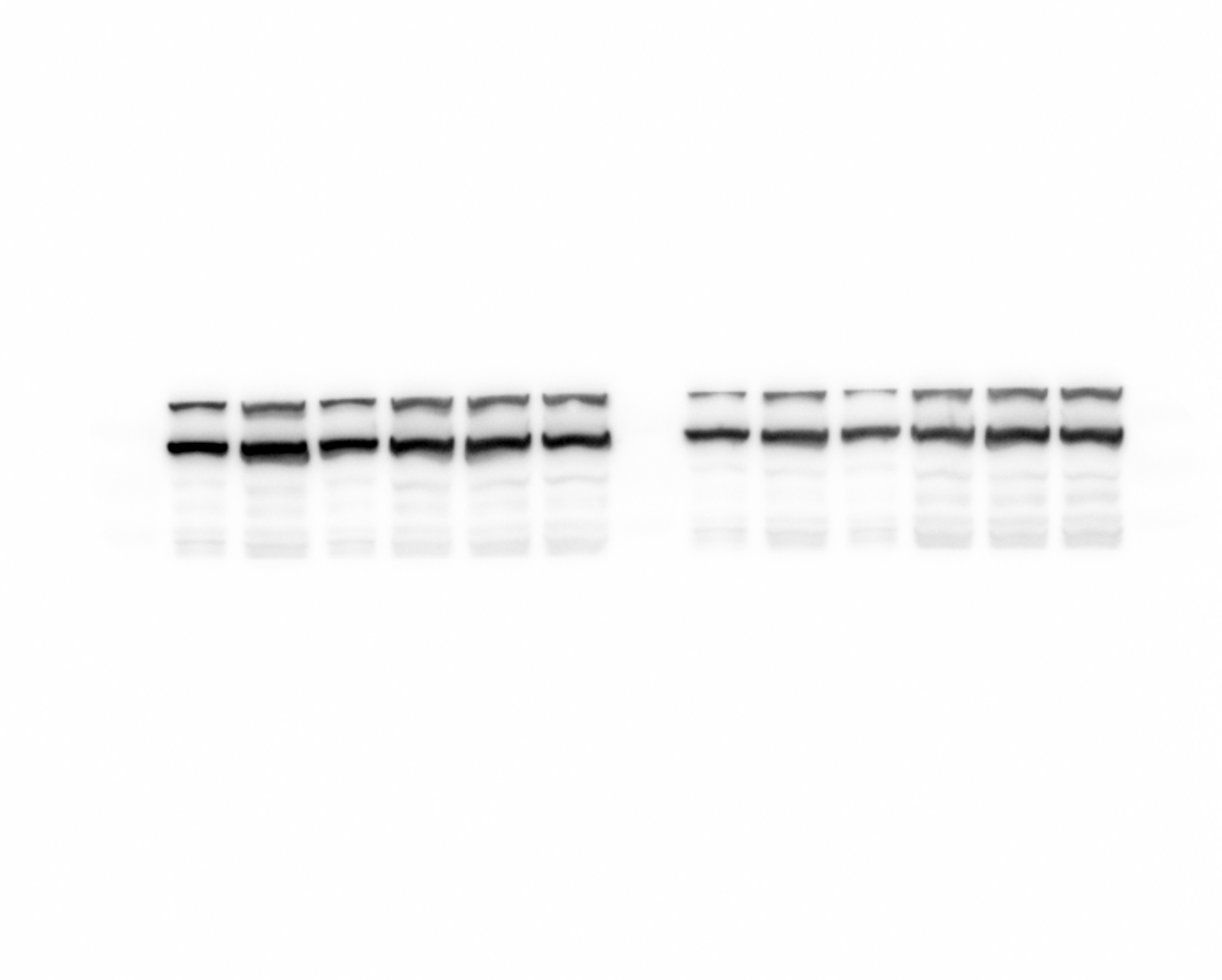

Supplement: Supplementary file 2 [file DataSheet8.zip › Fig.11/1-GSDMD/3-GSDMD-10S.Tif]

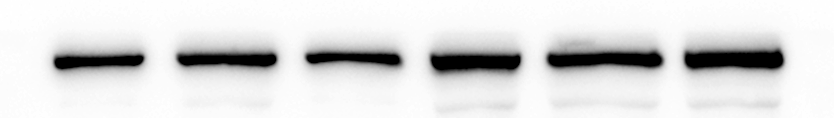

Supplement: Supplementary file 2 [file DataSheet8.zip › Fig.11/1-GSDMD/PS 下-2-GSDMD-10S.tif]

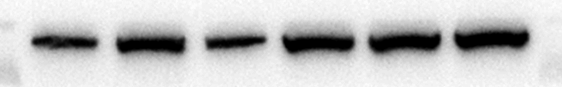

Supplement: Supplementary file 2 [file DataSheet8.zip › Fig.11/1-GSDMD/PS 左-1-GSDMD-10S.tif]

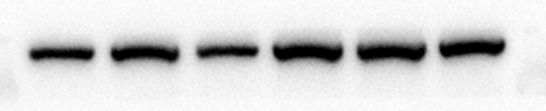

Supplement: Supplementary file 2 [file DataSheet8.zip › Fig.11/1-GSDMD/用 PS 右-1-GSDMD-10S.tif]

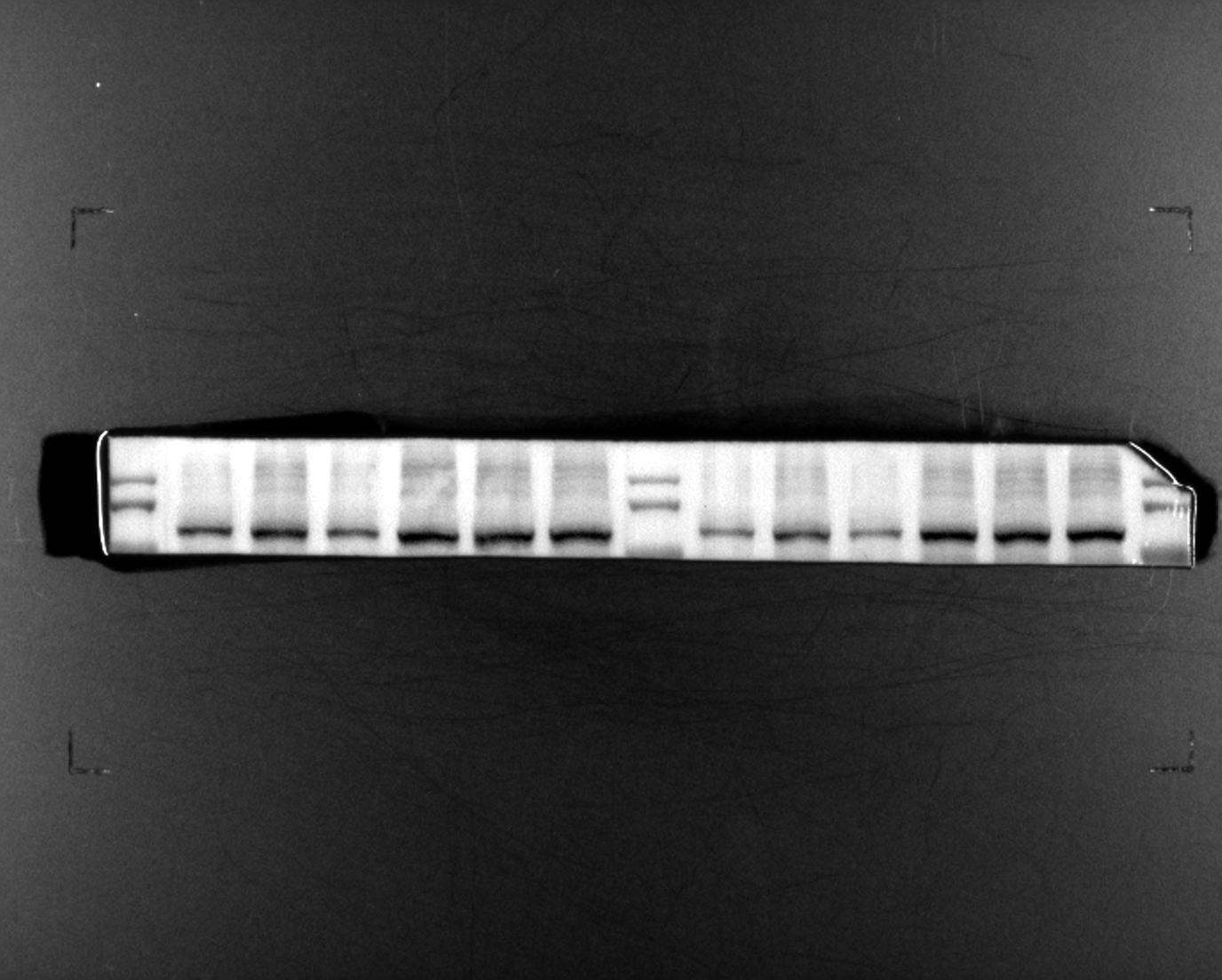

Supplement: Supplementary file 2 [file DataSheet8.zip › Fig.11/2-NLRP3/1-NLRP3-30S YT.Tif]

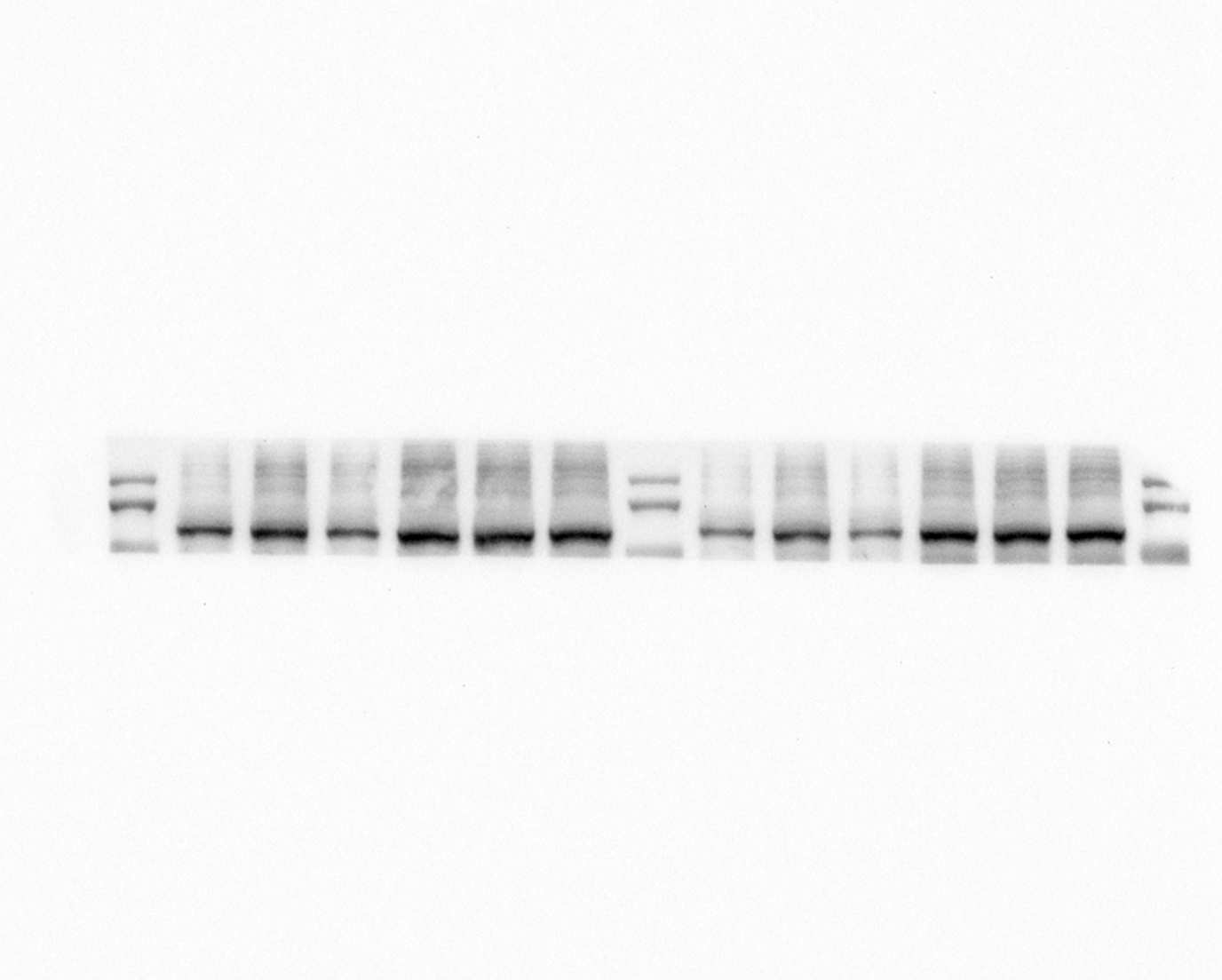

Supplement: Supplementary file 2 [file DataSheet8.zip › Fig.11/2-NLRP3/1-NLRP3-30S.Tif]

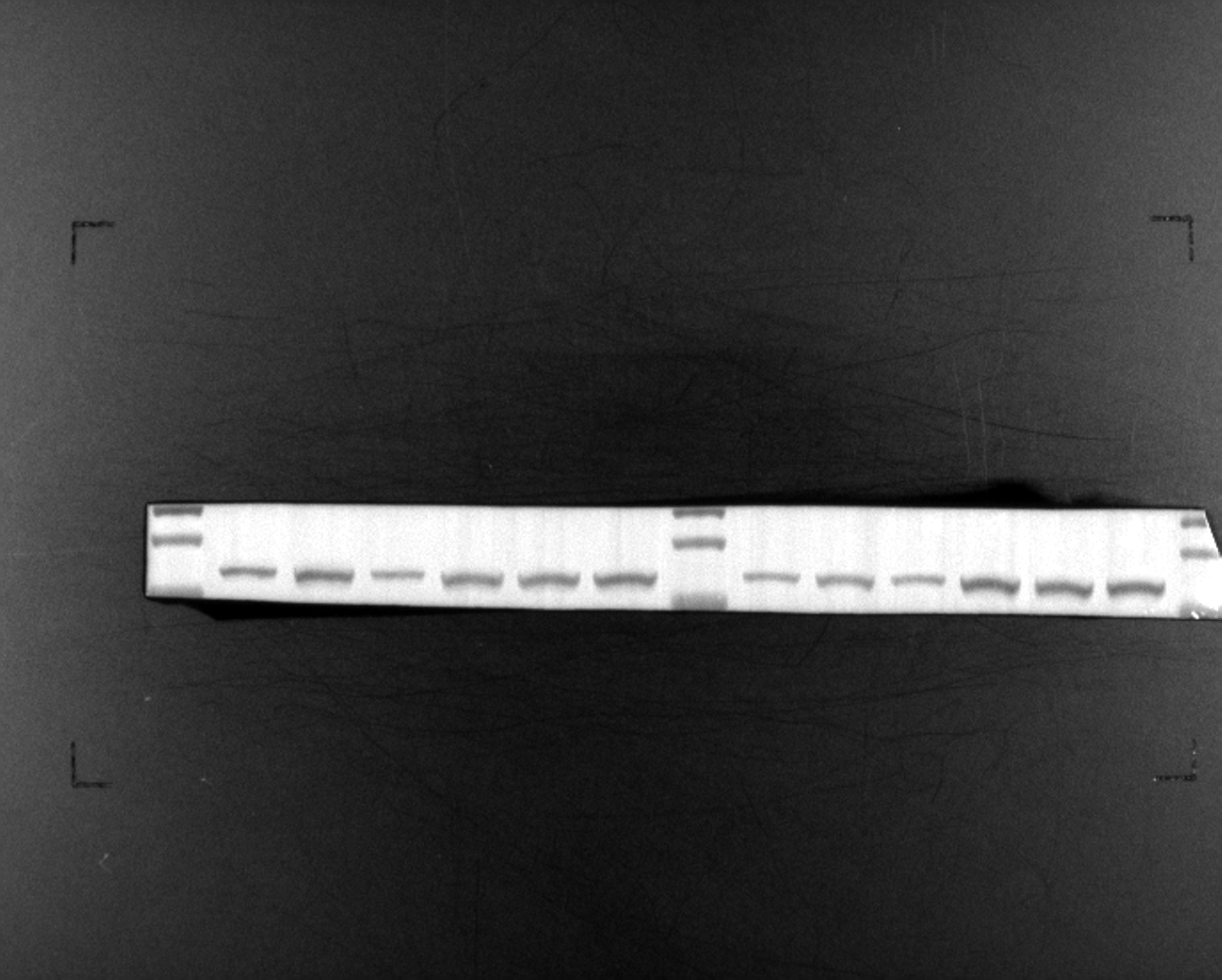

Supplement: Supplementary file 2 [file DataSheet8.zip › Fig.11/2-NLRP3/2-NLRP3-30S YT.Tif]

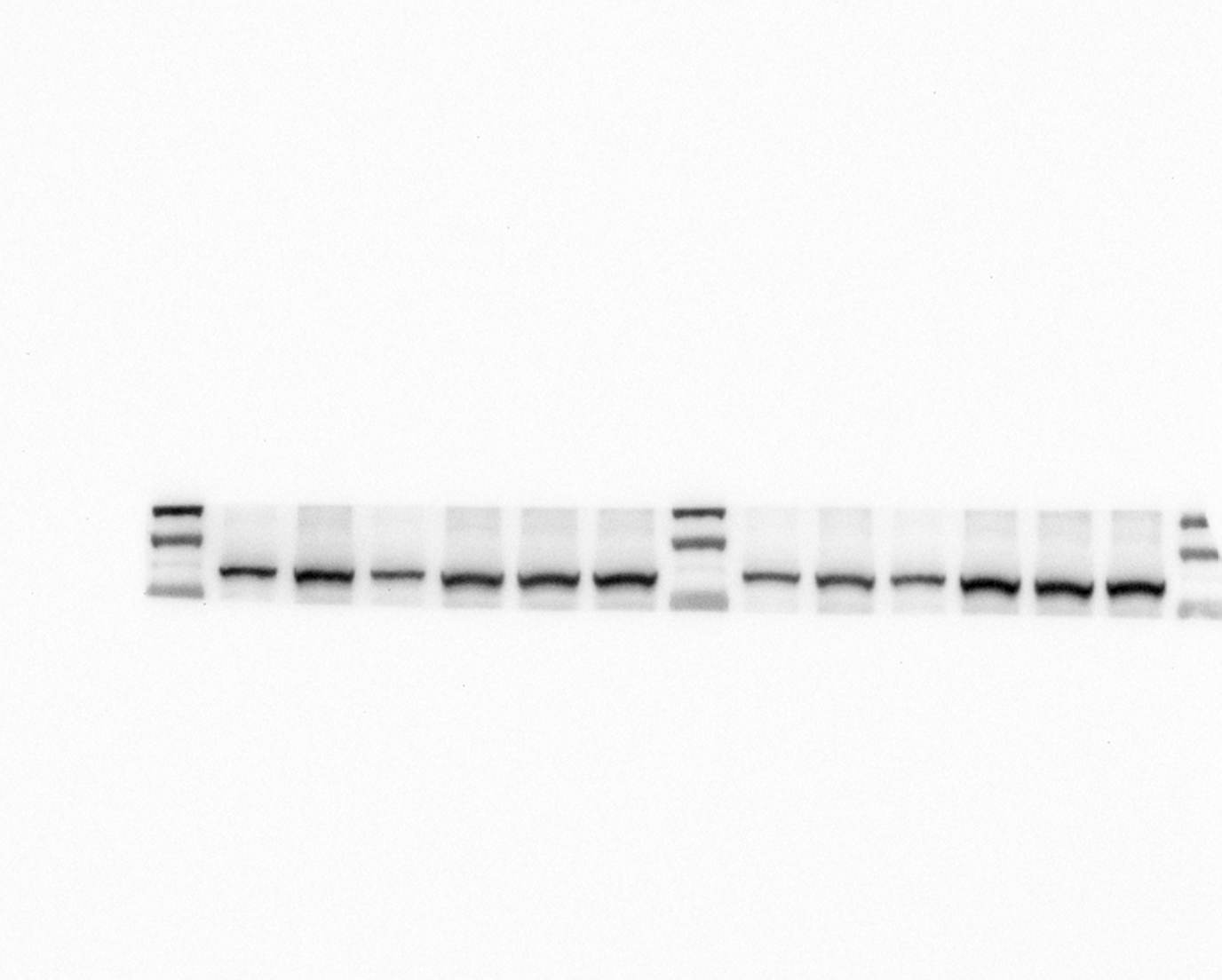

Supplement: Supplementary file 2 [file DataSheet8.zip › Fig.11/2-NLRP3/2-NLRP3-30S.Tif]

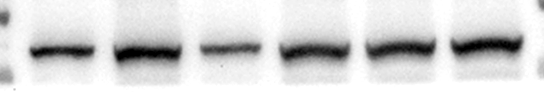

Supplement: Supplementary file 2 [file DataSheet8.zip › Fig.11/2-NLRP3/PS-左-2-NLRP3-30S.tif]

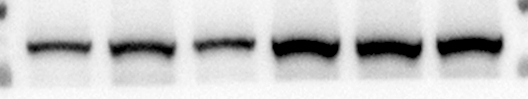

Supplement: Supplementary file 2 [file DataSheet8.zip › Fig.11/2-NLRP3/用 PS-右-2-NLRP3-30S.tif]

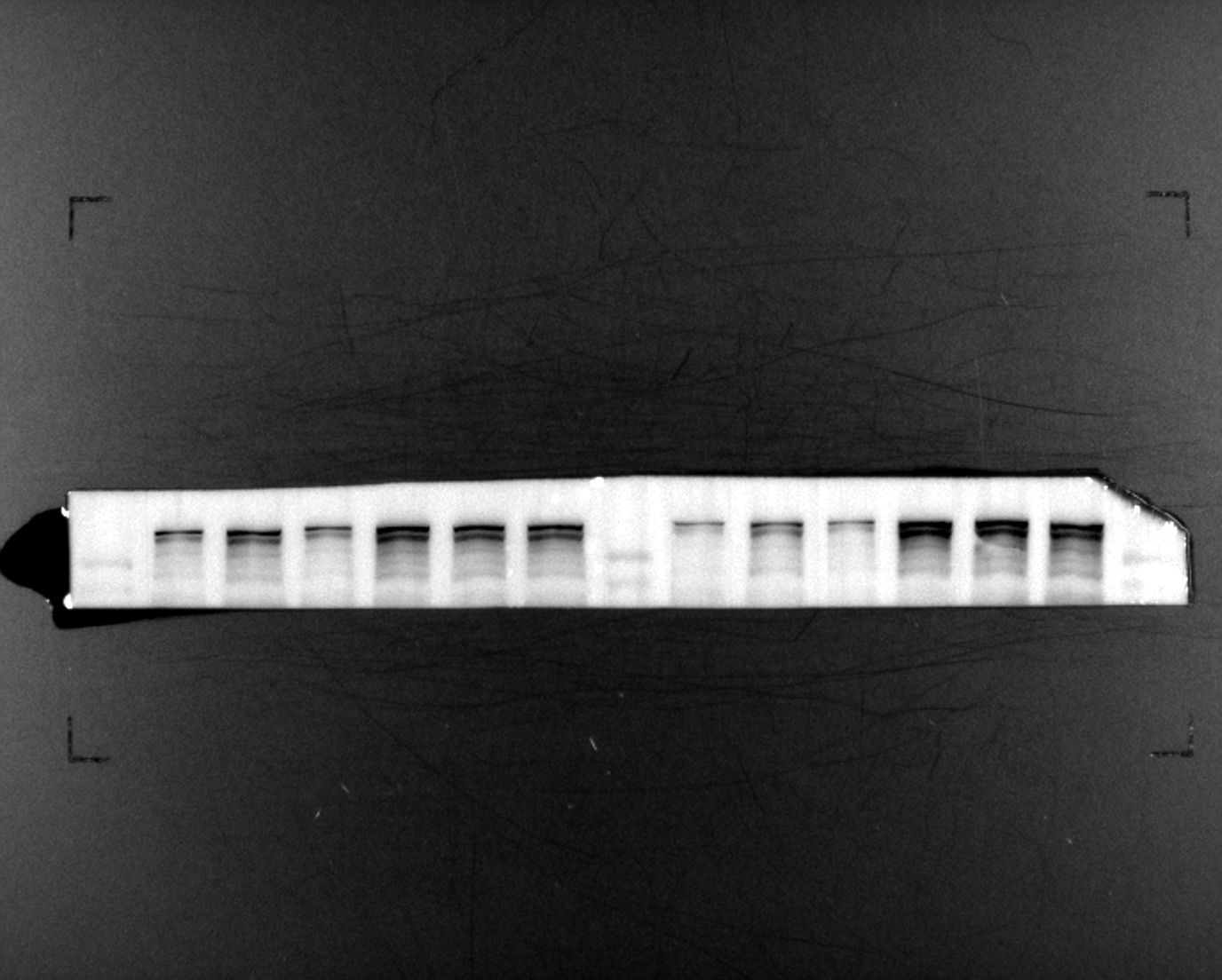

Supplement: Supplementary file 2 [file DataSheet8.zip › Fig.11/3-CASPASE-1/1-Caspase-1-30s YT.Tif]

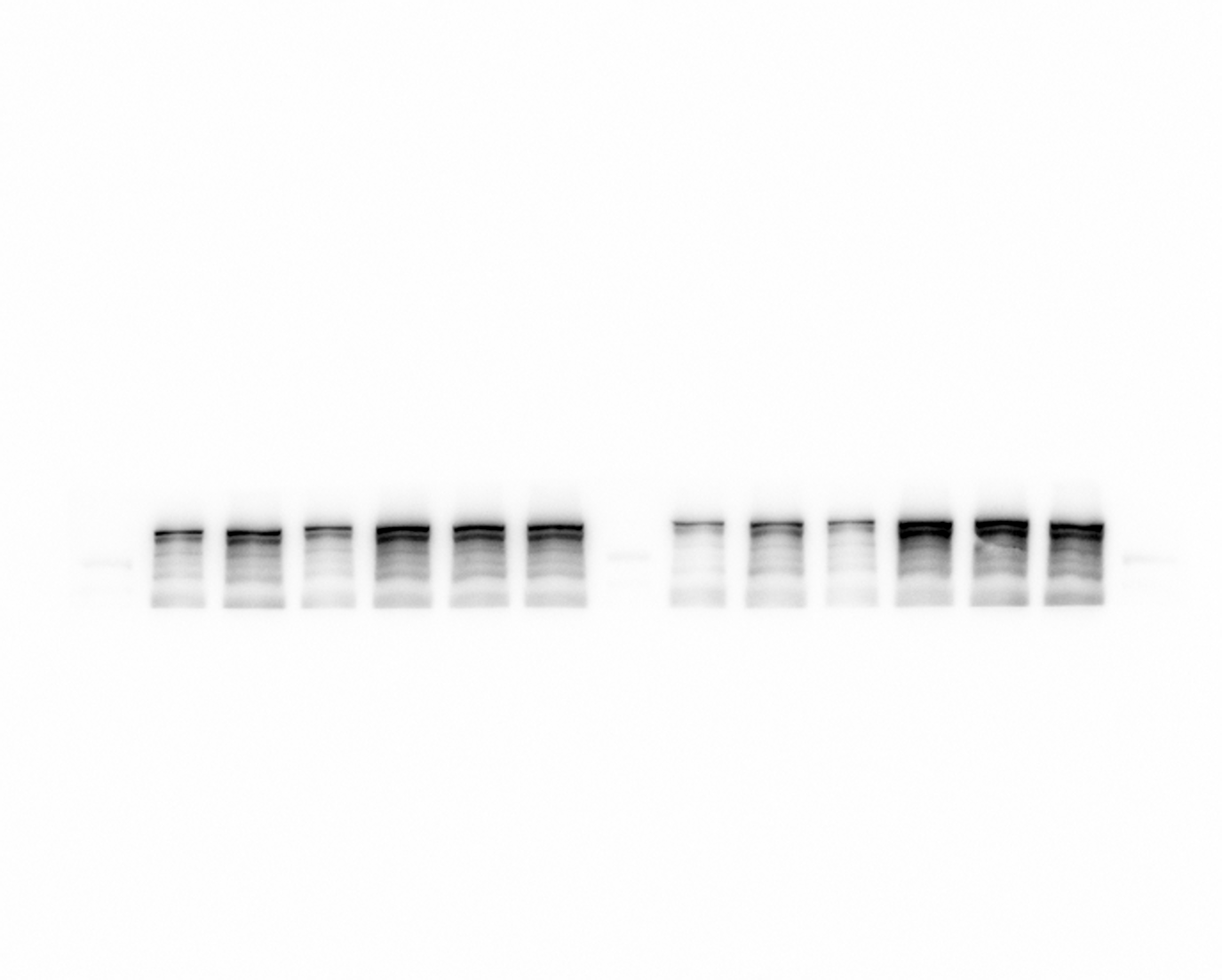

Supplement: Supplementary file 2 [file DataSheet8.zip › Fig.11/3-CASPASE-1/1-Caspase-1-30s.Tif]

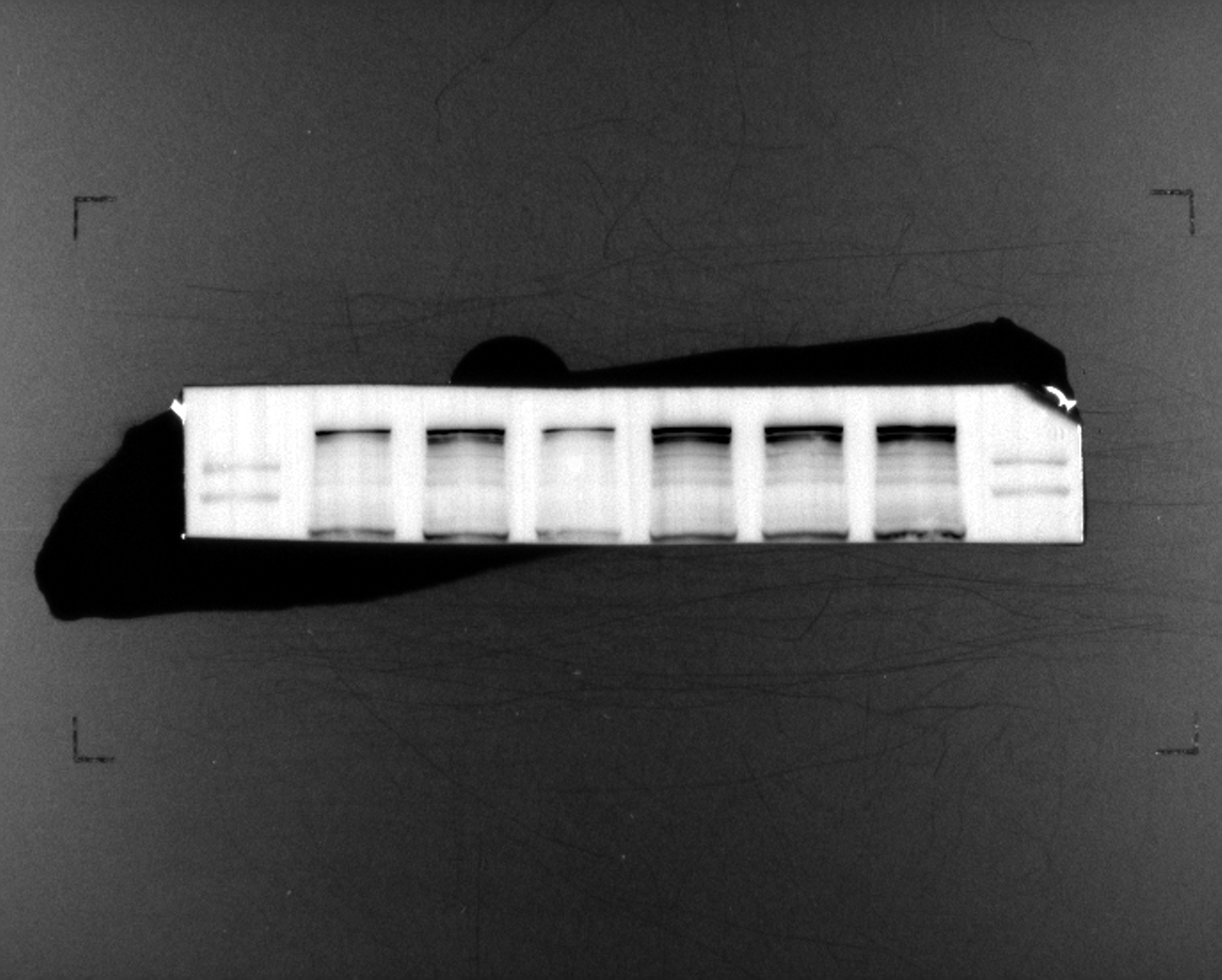

Supplement: Supplementary file 2 [file DataSheet8.zip › Fig.11/3-CASPASE-1/2-Caspase-1-30s YT.Tif]

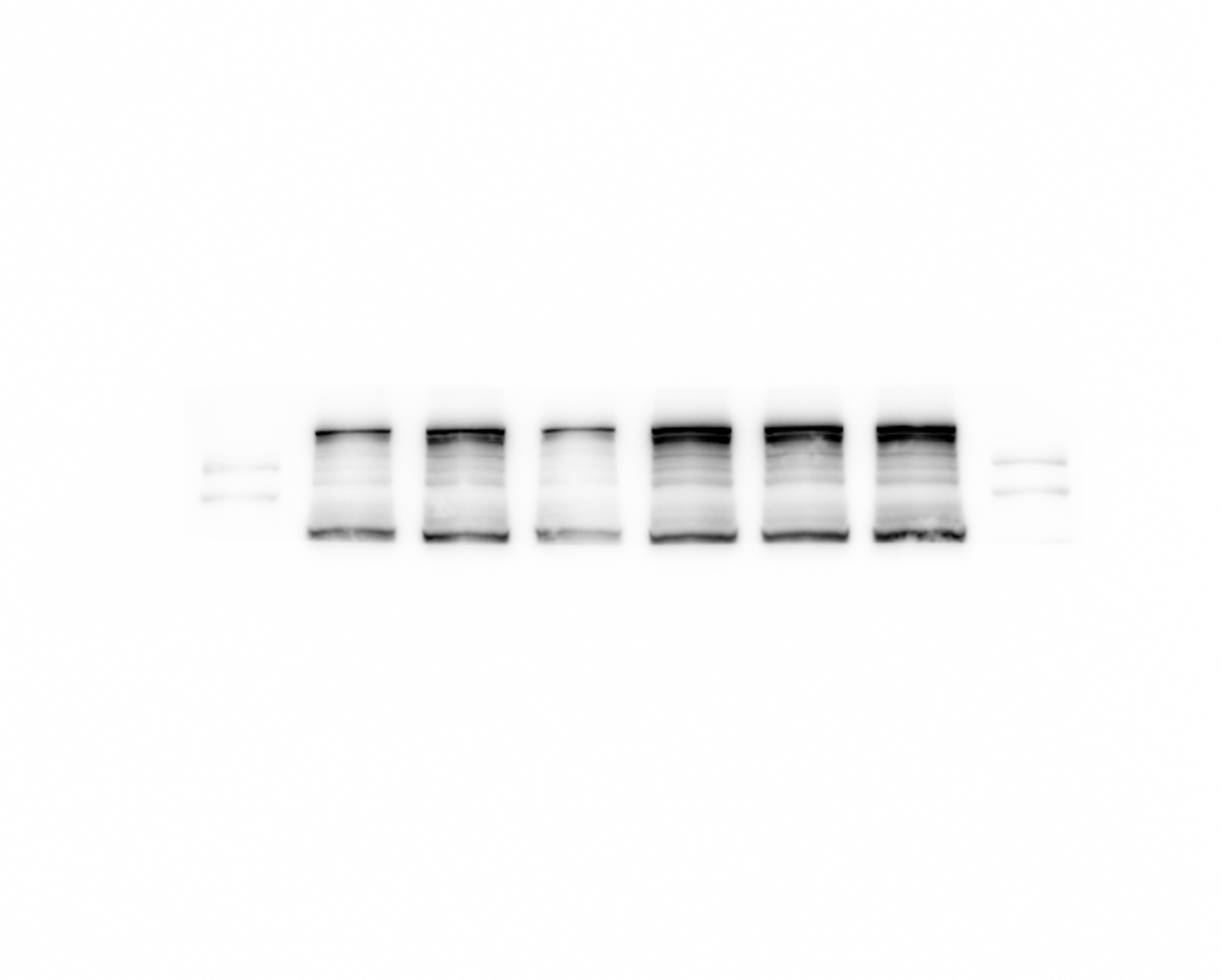

Supplement: Supplementary file 2 [file DataSheet8.zip › Fig.11/3-CASPASE-1/2-Caspase-1-30s.Tif]

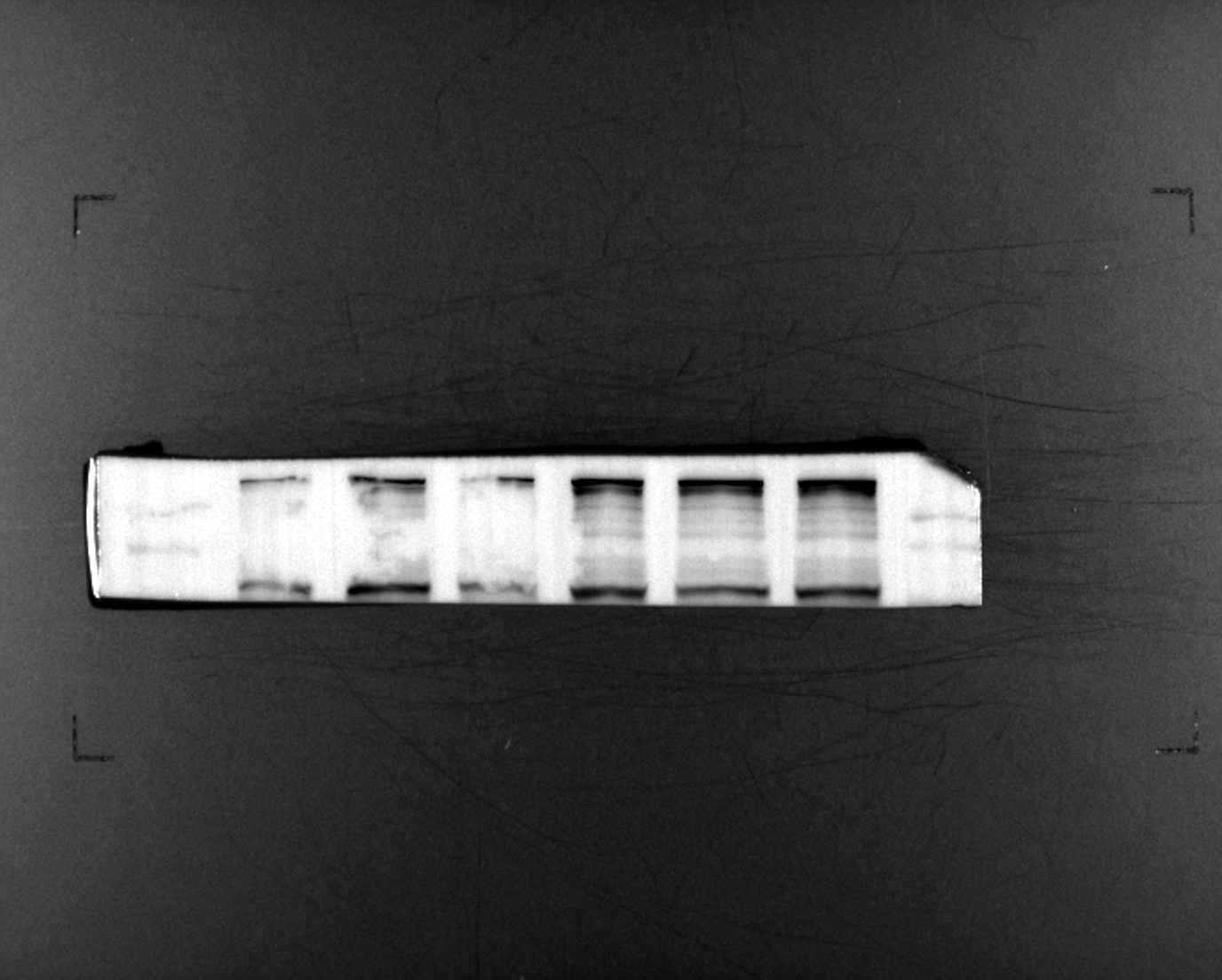

Supplement: Supplementary file 2 [file DataSheet8.zip › Fig.11/3-CASPASE-1/3-Caspase-1-30s YT.Tif]

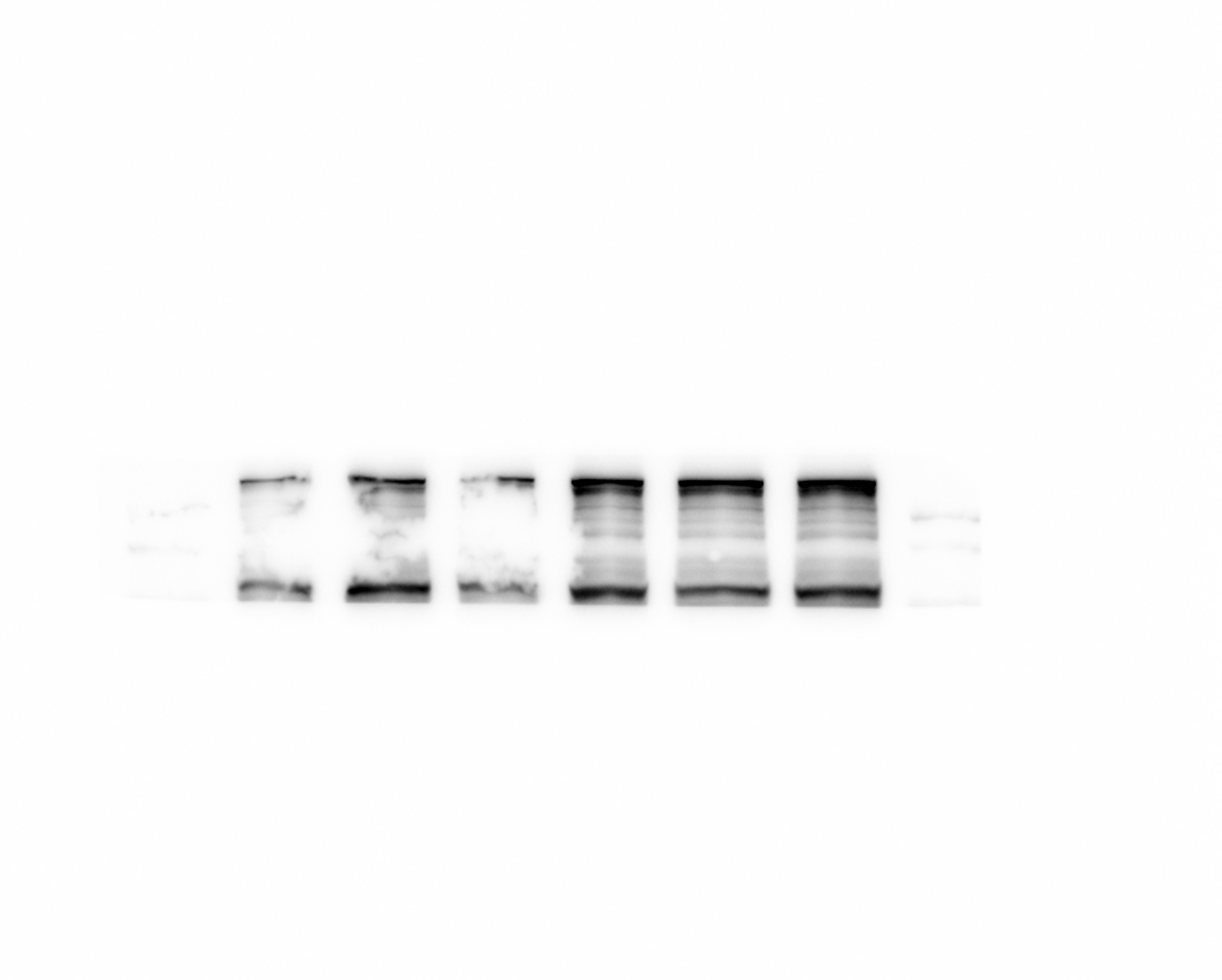

Supplement: Supplementary file 2 [file DataSheet8.zip › Fig.11/3-CASPASE-1/3-Caspase-1-30s.Tif]

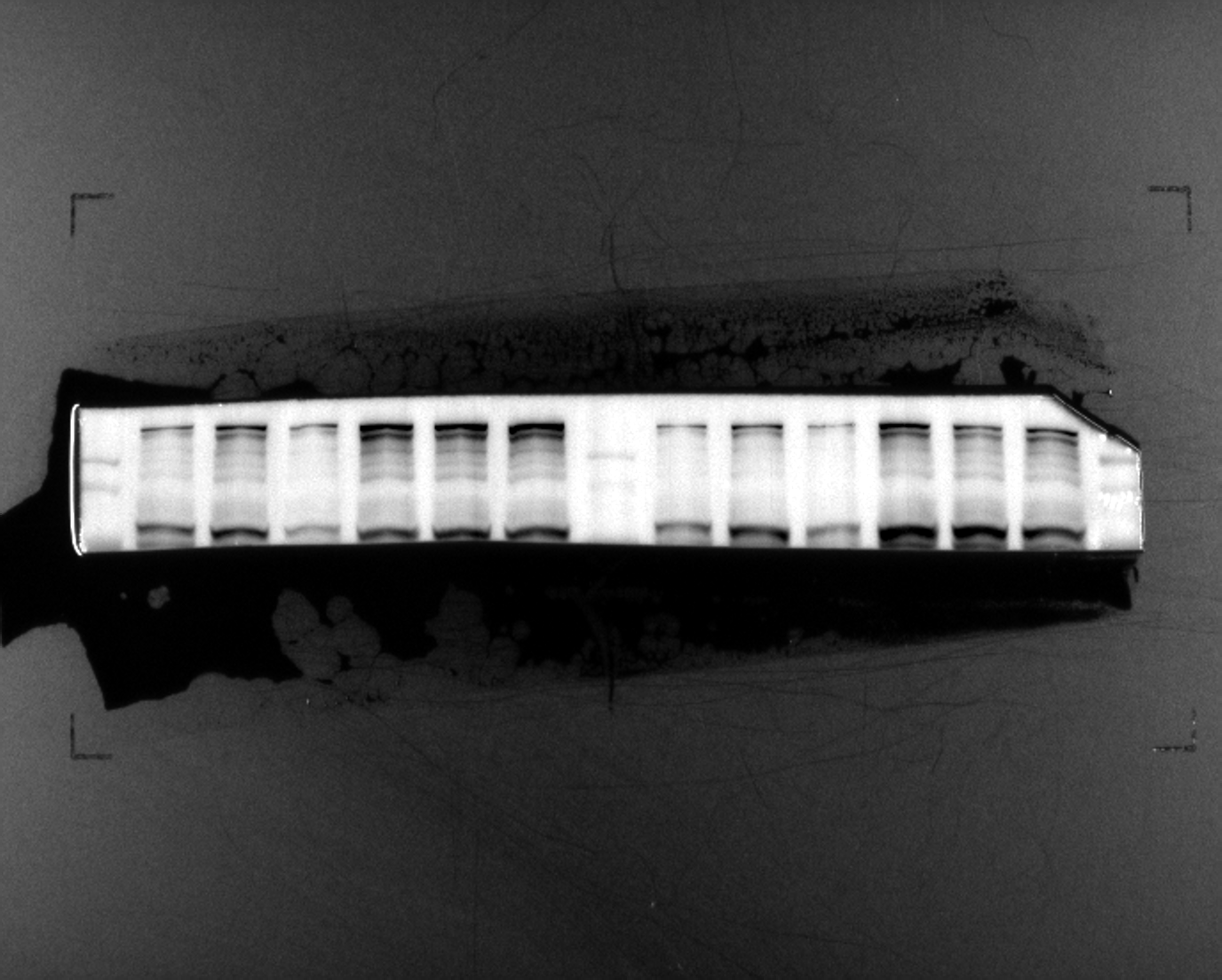

Supplement: Supplementary file 2 [file DataSheet8.zip › Fig.11/3-CASPASE-1/4-Caspase-1-30s YT.Tif]

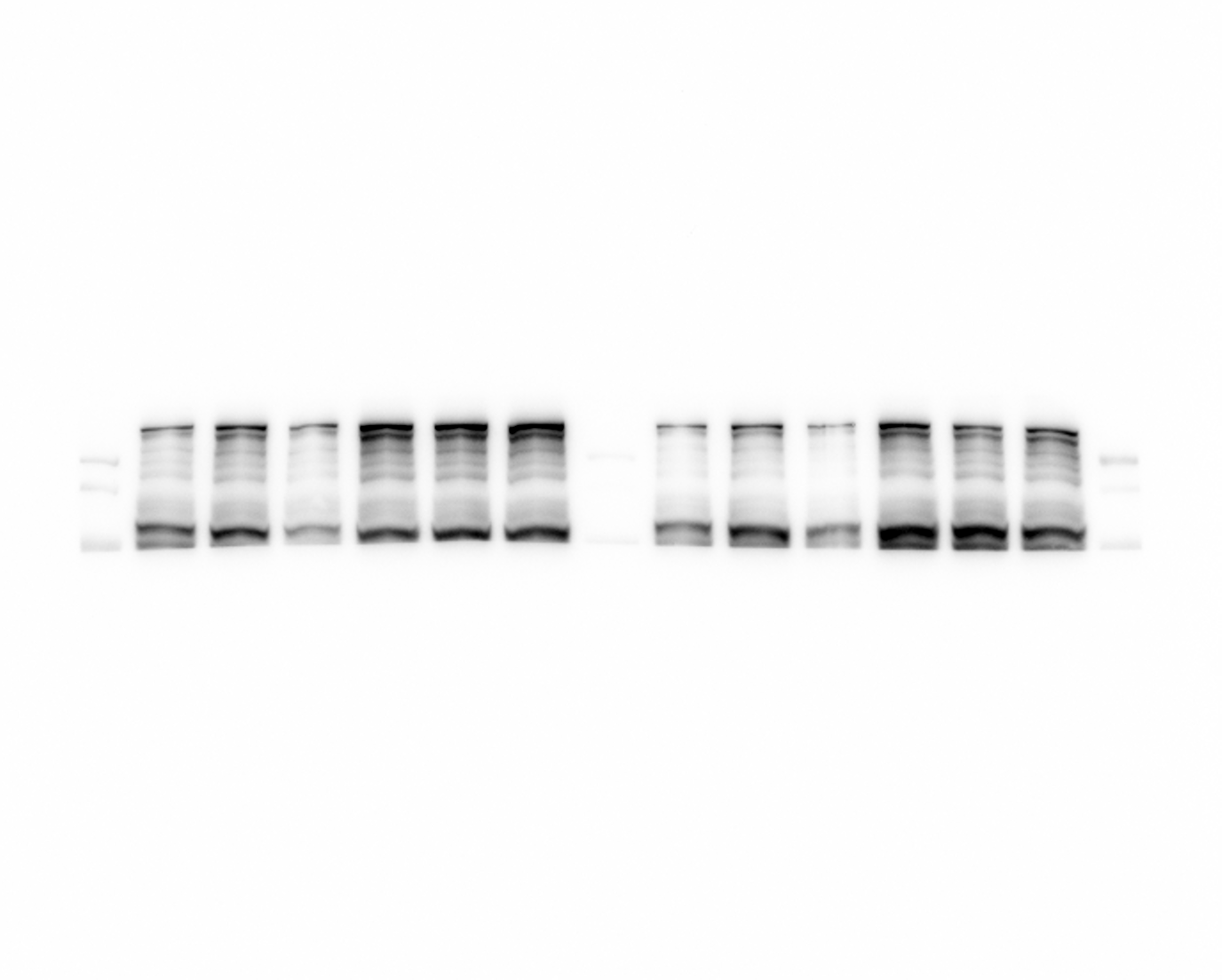

Supplement: Supplementary file 2 [file DataSheet8.zip › Fig.11/3-CASPASE-1/4-Caspase-1-30s.Tif]

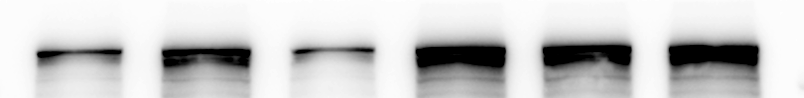

Supplement: Supplementary file 2 [file DataSheet8.zip › Fig.11/3-CASPASE-1/PS -2-Caspase-1-30s.tif]

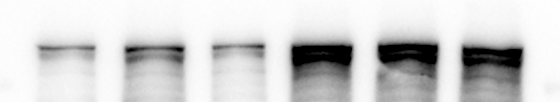

Supplement: Supplementary file 2 [file DataSheet8.zip › Fig.11/3-CASPASE-1/PS-右-1-Caspase-1-30s.tif]

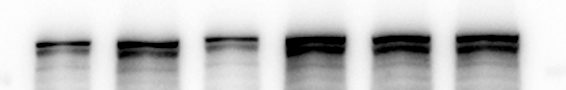

Supplement: Supplementary file 2 [file DataSheet8.zip › Fig.11/3-CASPASE-1/用 PS-左-1-Caspase-1-30s.tif]

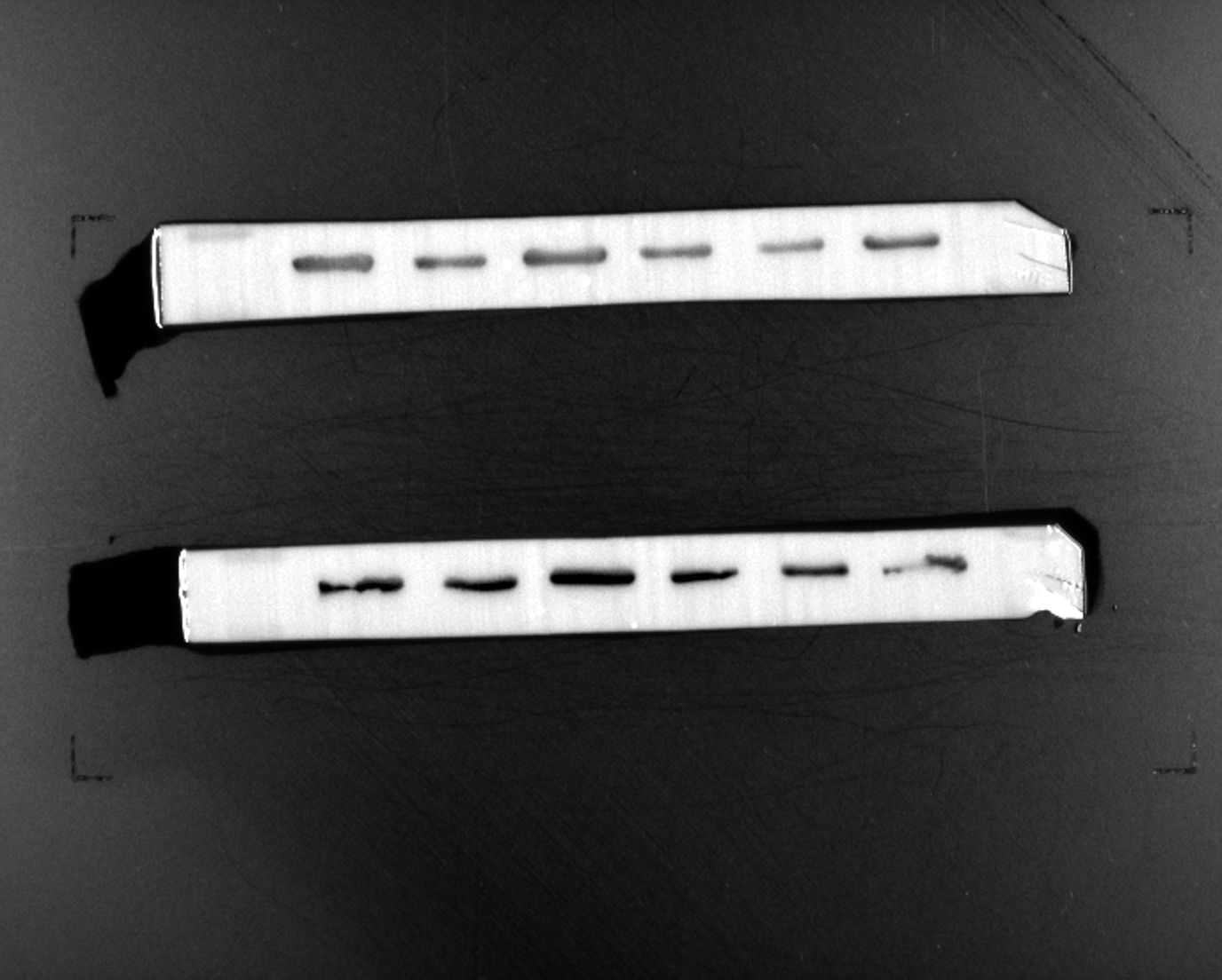

Supplement: Supplementary file 2 [file DataSheet8.zip › Fig.11/4-Nephrin/1-Nephrin-20s YT.Tif]

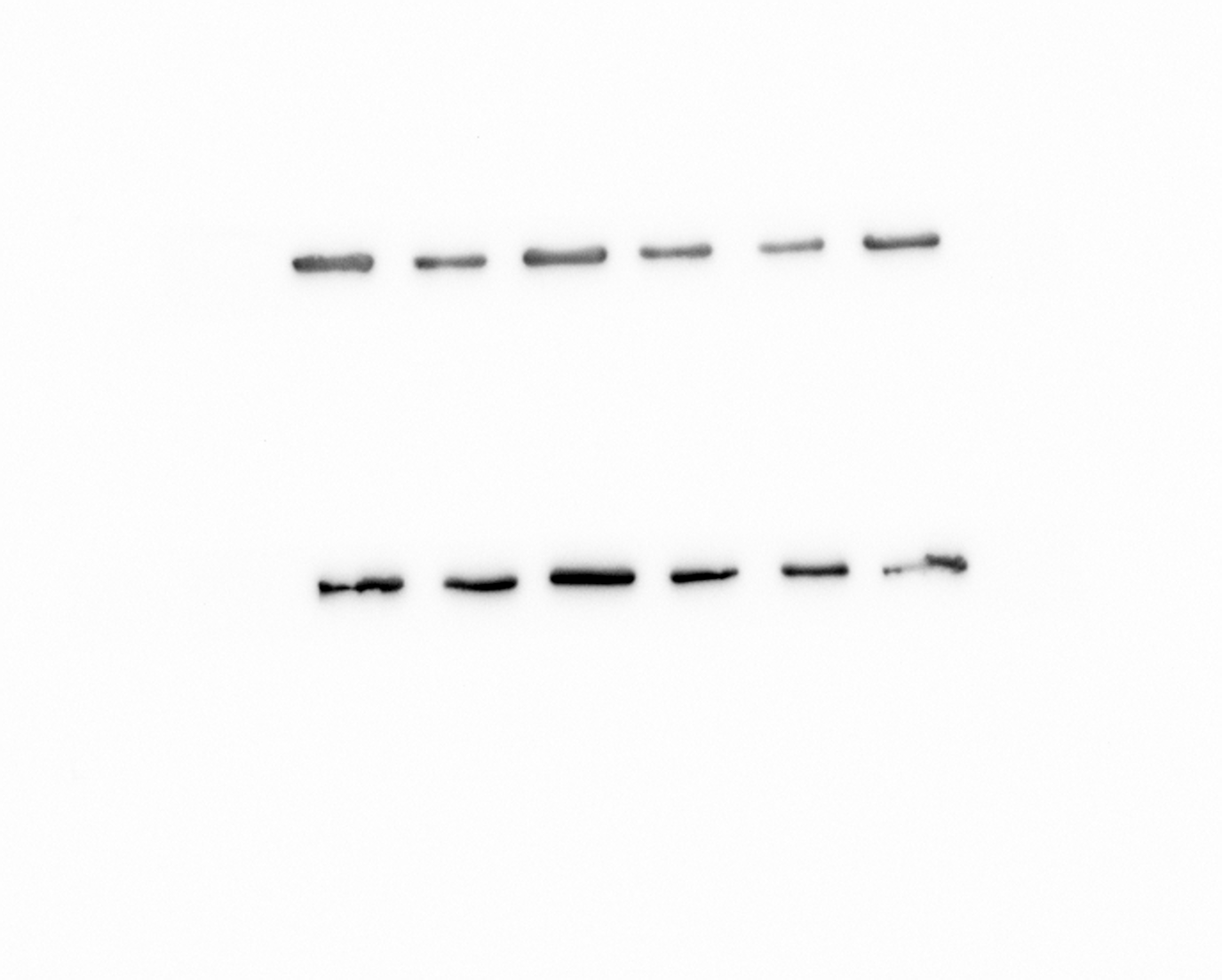

Supplement: Supplementary file 2 [file DataSheet8.zip › Fig.11/4-Nephrin/1-Nephrin-20s.Tif]

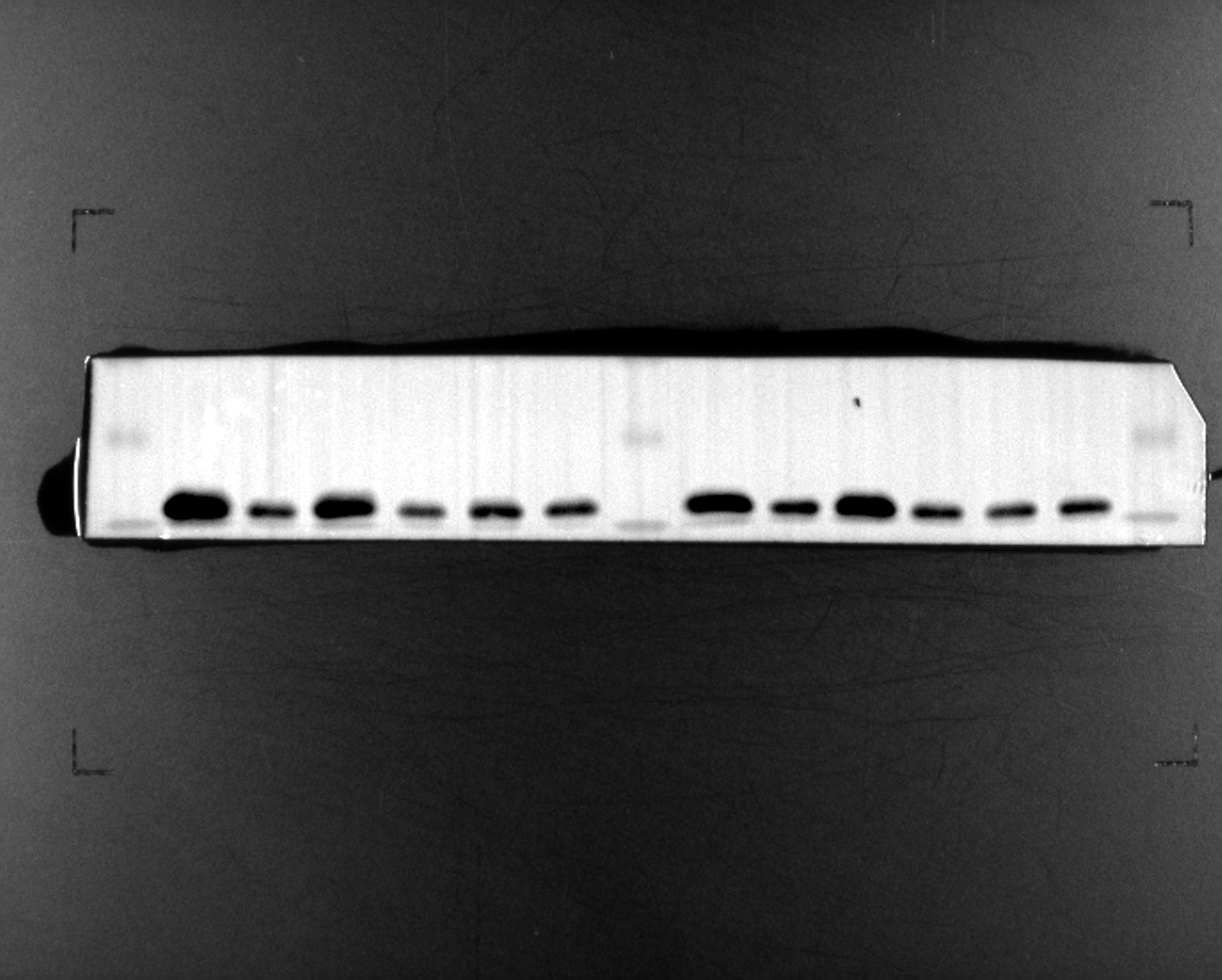

Supplement: Supplementary file 2 [file DataSheet8.zip › Fig.11/4-Nephrin/2-Nephrin-20s YT.Tif]

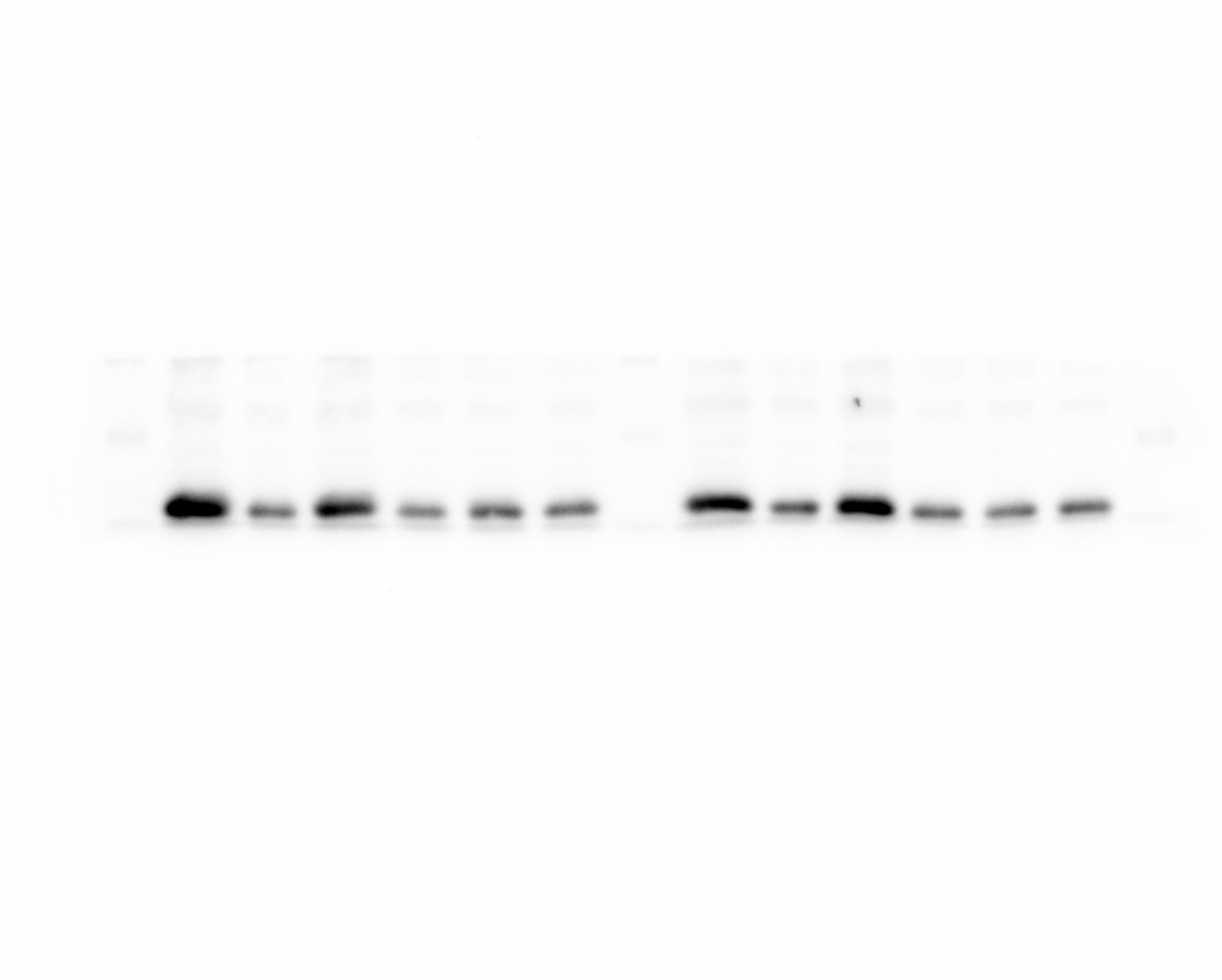

Supplement: Supplementary file 2 [file DataSheet8.zip › Fig.11/4-Nephrin/2-Nephrin-20s.Tif]

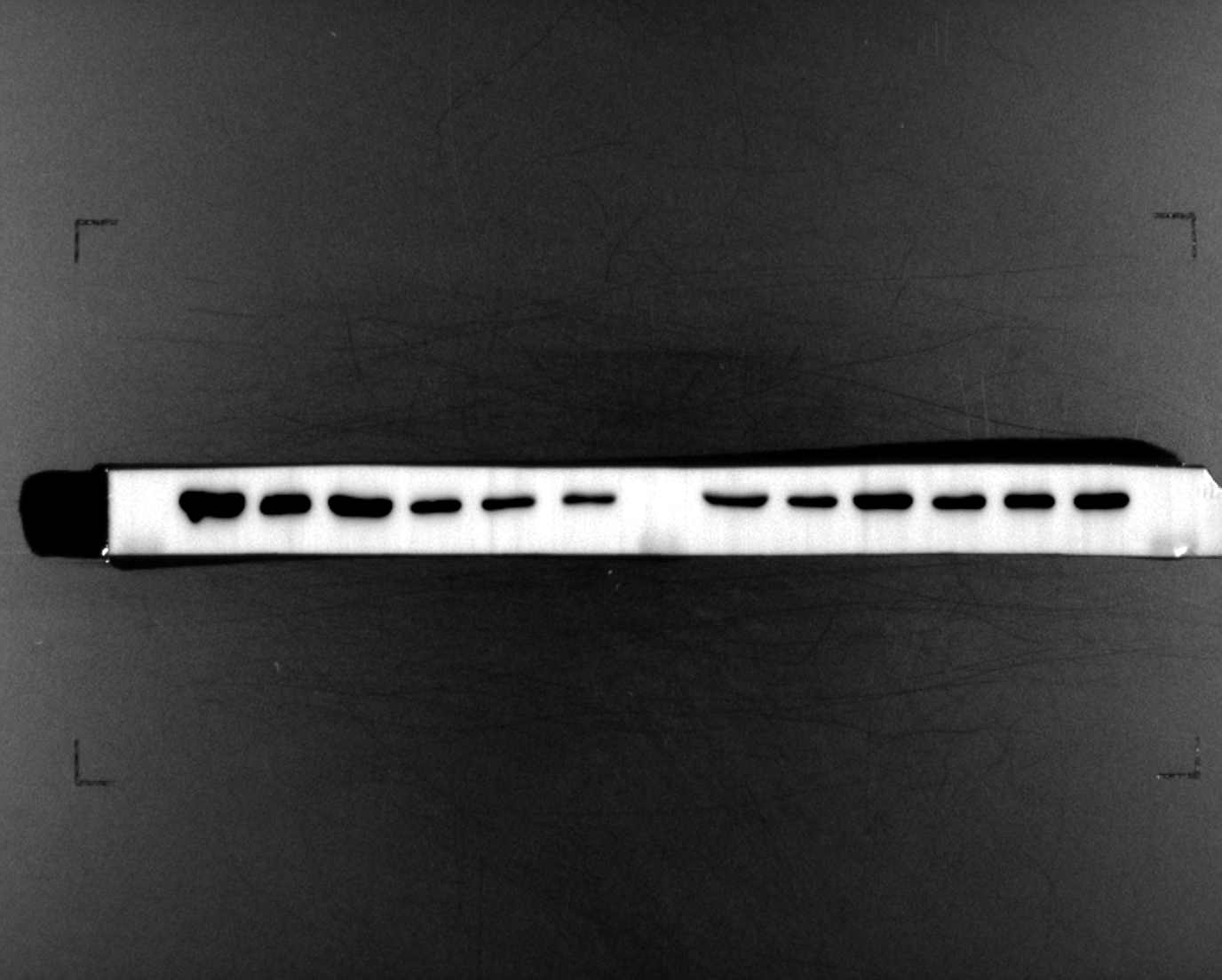

Supplement: Supplementary file 2 [file DataSheet8.zip › Fig.11/4-Nephrin/3-Nephrin-20s YT.Tif]

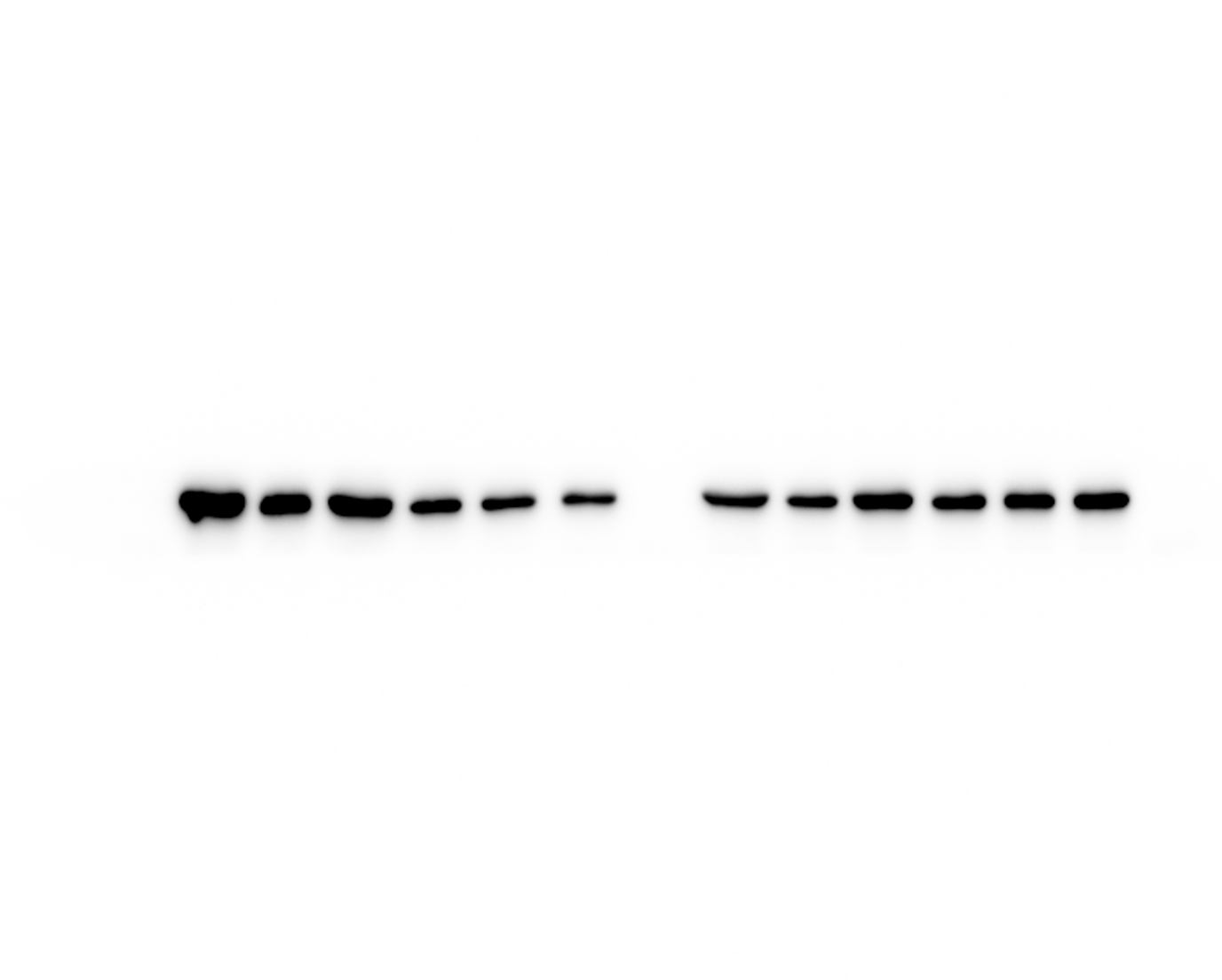

Supplement: Supplementary file 2 [file DataSheet8.zip › Fig.11/4-Nephrin/3-Nephrin-20s.Tif]

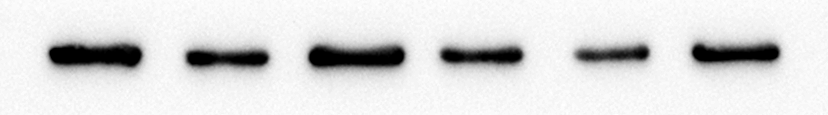

Supplement: Supplementary file 2 [file DataSheet8.zip › Fig.11/4-Nephrin/PS-上-1-Nephrin-20s.tif]

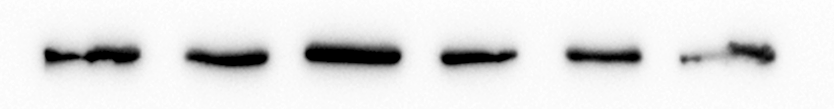

Supplement: Supplementary file 2 [file DataSheet8.zip › Fig.11/4-Nephrin/PS-下-1-Nephrin-20s.tif]

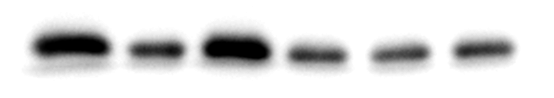

Supplement: Supplementary file 2 [file DataSheet8.zip › Fig.11/4-Nephrin/PS-右-2-Nephrin-20s.tif]

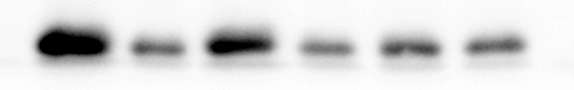

Supplement: Supplementary file 2 [file DataSheet8.zip › Fig.11/4-Nephrin/用 PS-左-2-Nephrin-20s.tif]

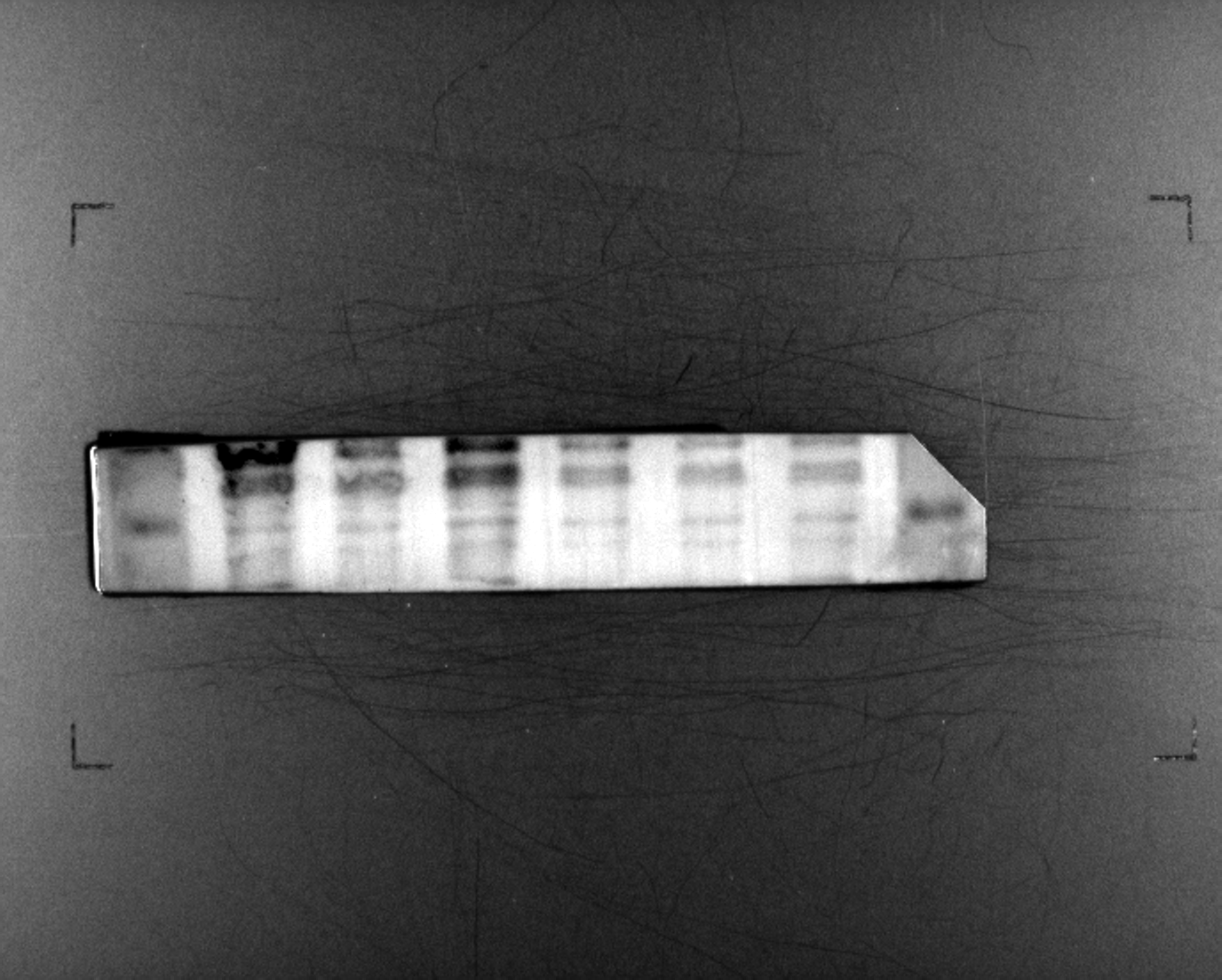

Supplement: Supplementary file 2 [file DataSheet8.zip › Fig.11/5-ZO-1/1-ZO-1-60s YT.Tif]

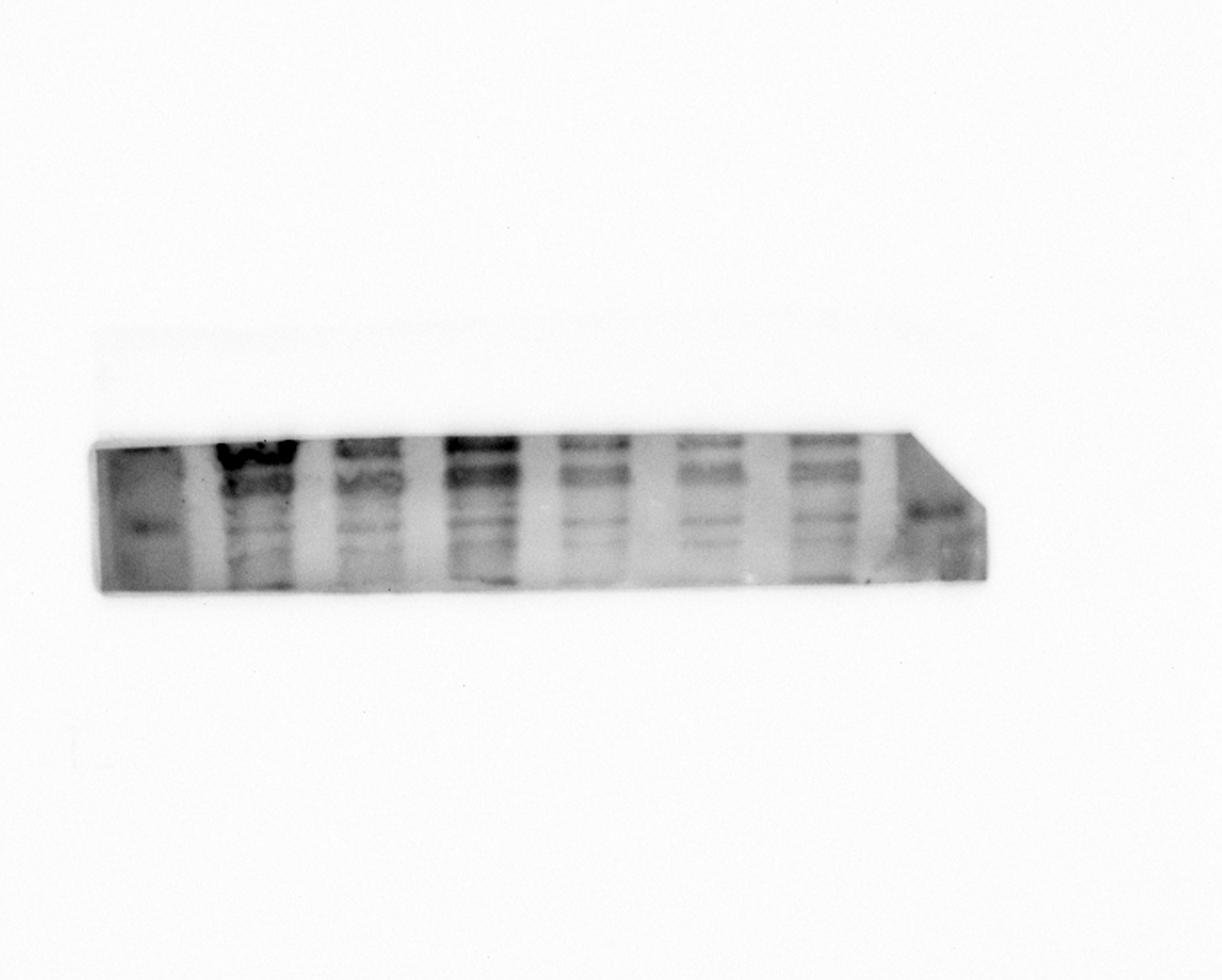

Supplement: Supplementary file 2 [file DataSheet8.zip › Fig.11/5-ZO-1/1-ZO-1-60s.Tif]

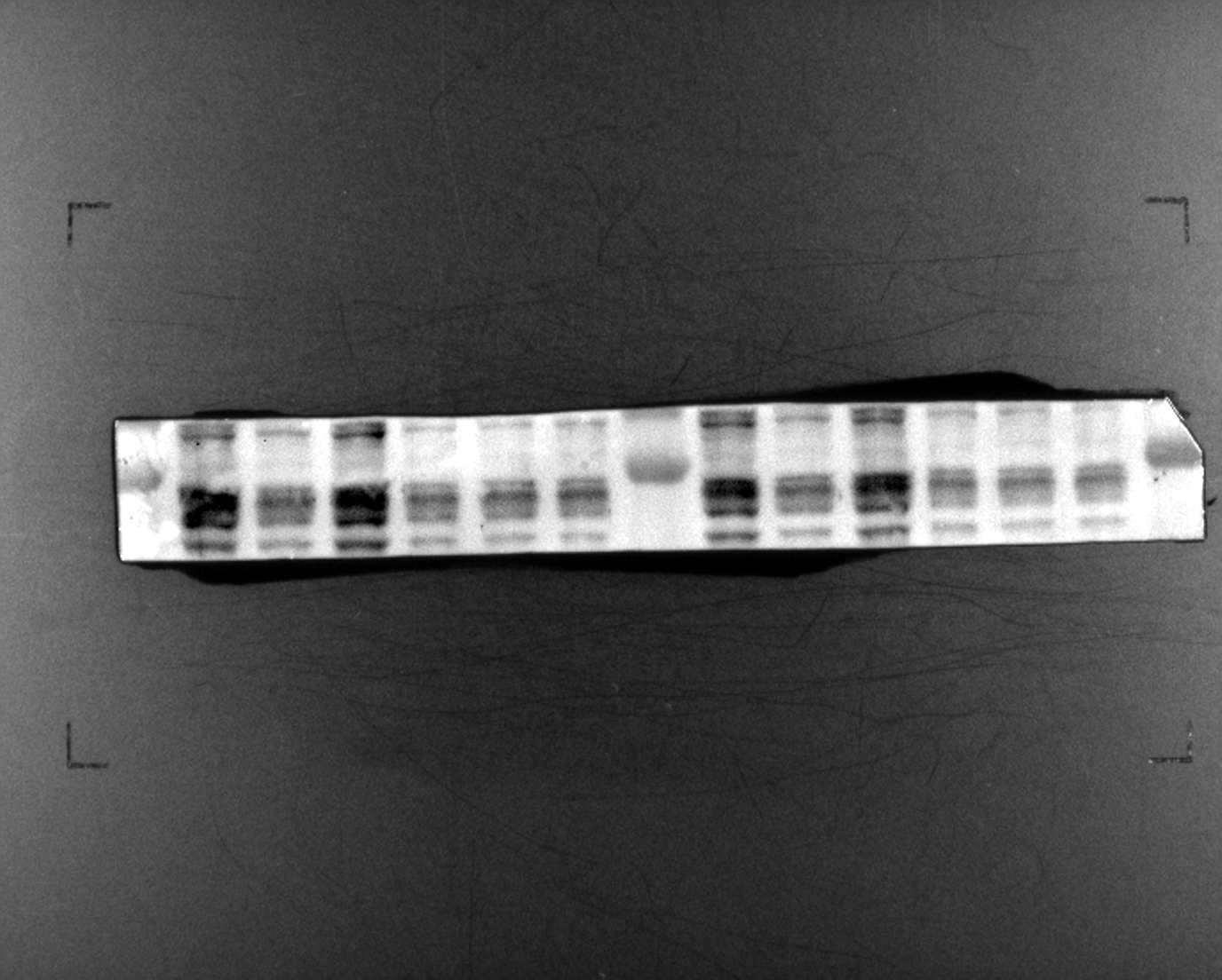

Supplement: Supplementary file 2 [file DataSheet8.zip › Fig.11/5-ZO-1/2-ZO-1-60s YT.Tif]

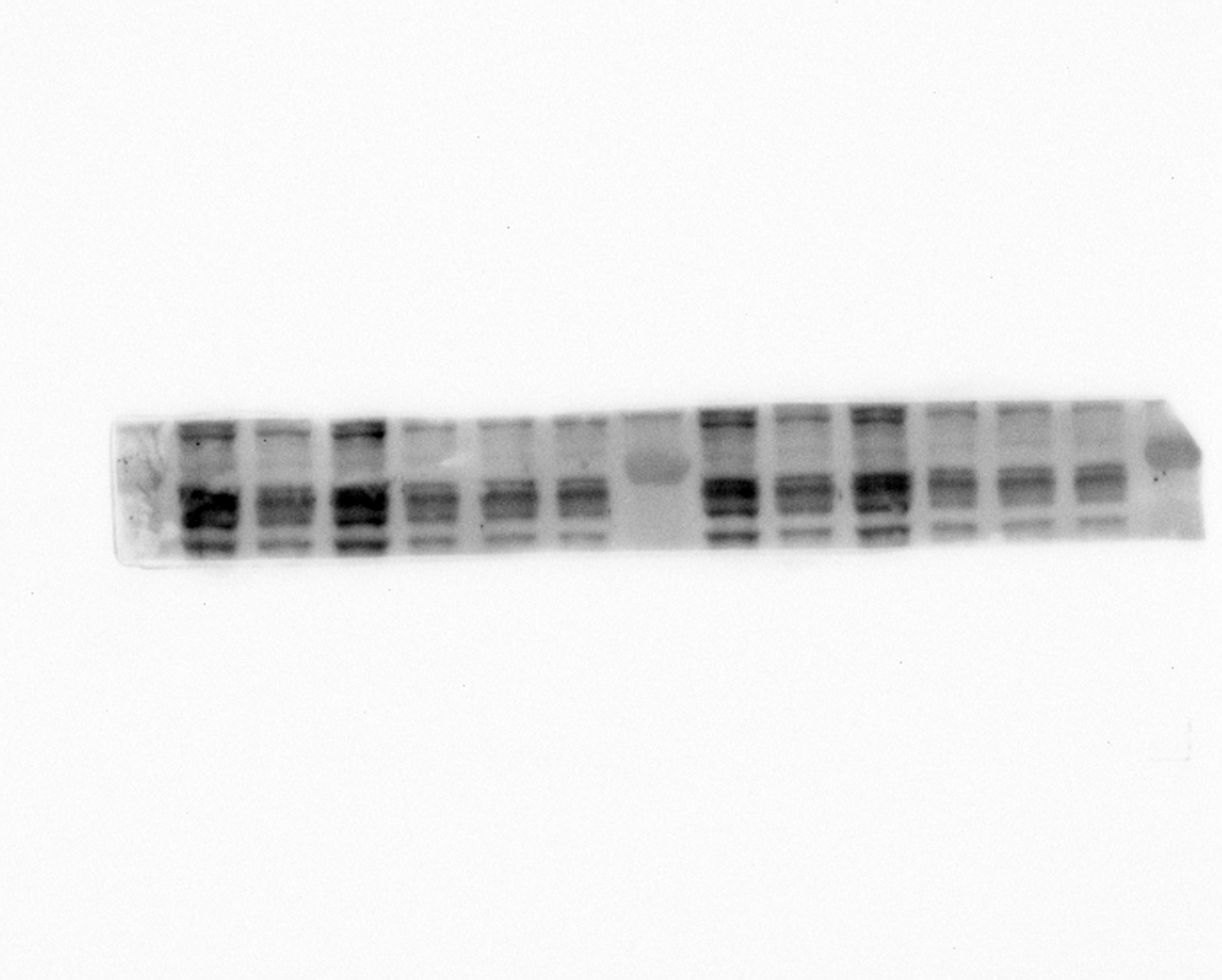

Supplement: Supplementary file 2 [file DataSheet8.zip › Fig.11/5-ZO-1/2-ZO-1-60s.Tif]

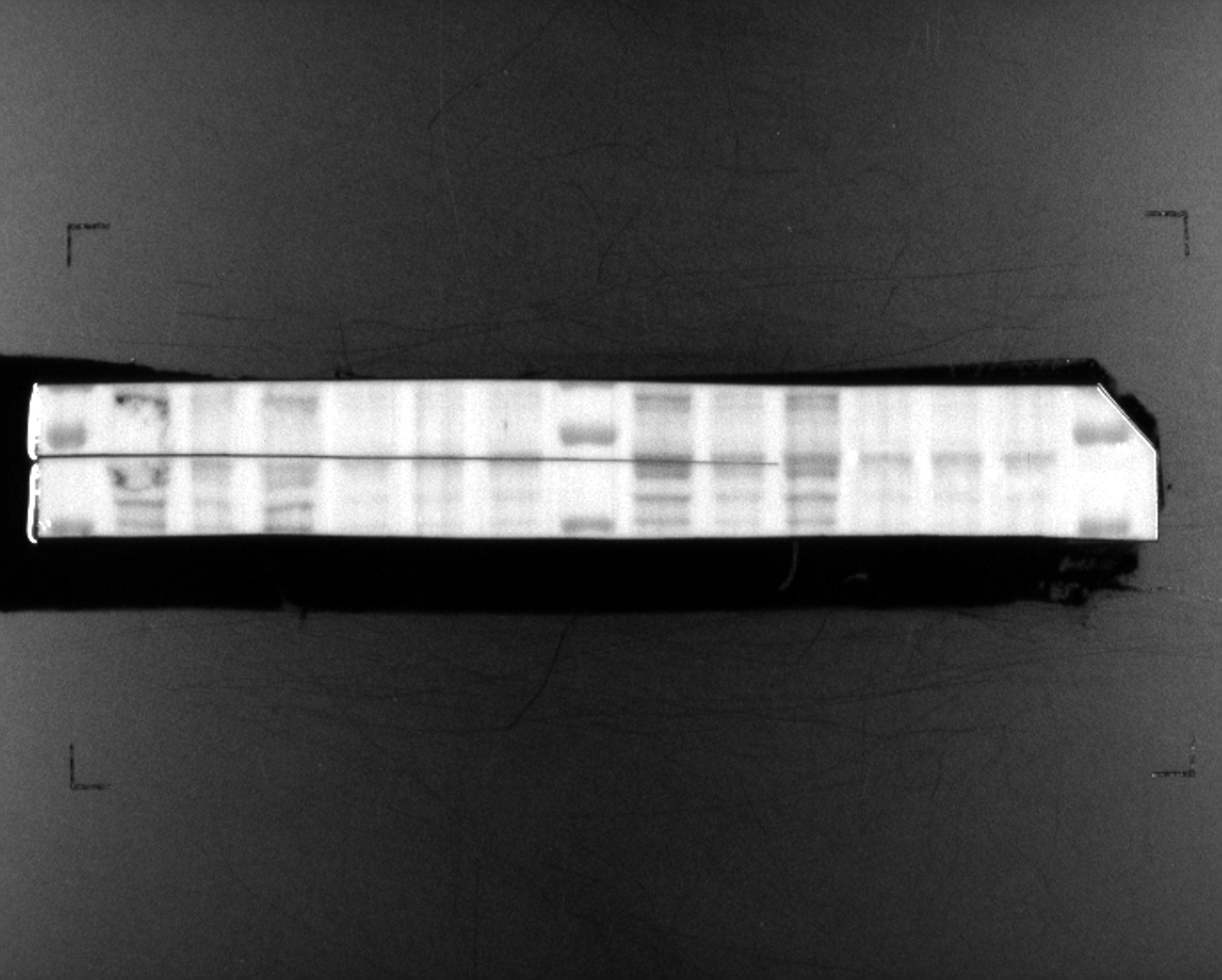

Supplement: Supplementary file 2 [file DataSheet8.zip › Fig.11/5-ZO-1/3-ZO-1-60s YT.Tif]

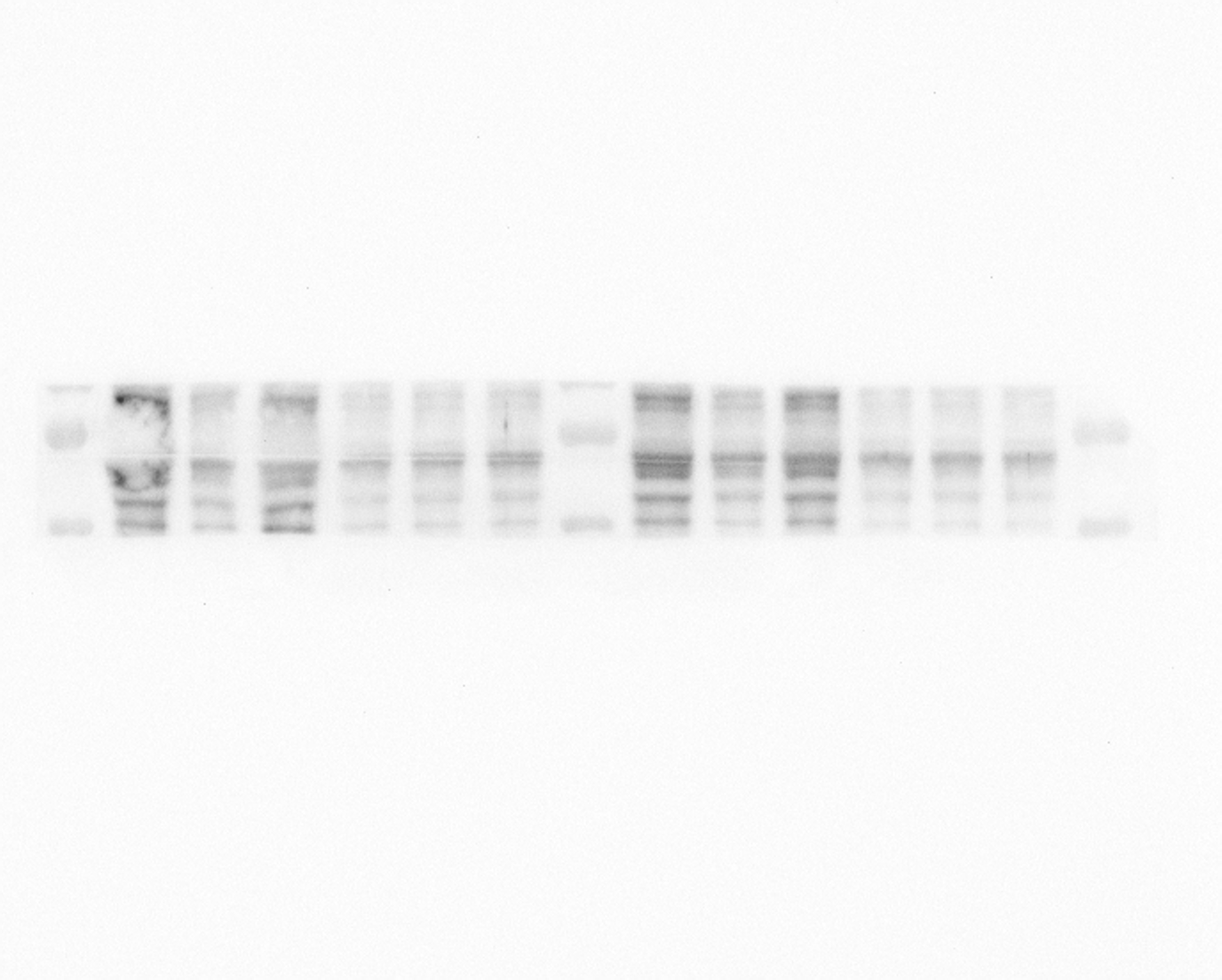

Supplement: Supplementary file 2 [file DataSheet8.zip › Fig.11/5-ZO-1/3-ZO-1-60s.Tif]

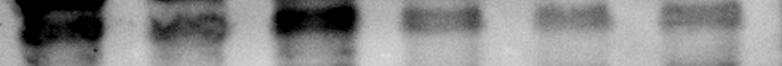

Supplement: Supplementary file 2 [file DataSheet8.zip › Fig.11/5-ZO-1/PS 1-ZO-1-60s.tif]

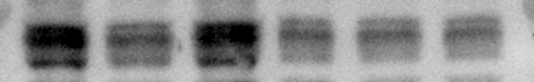

Supplement: Supplementary file 2 [file DataSheet8.zip › Fig.11/5-ZO-1/PS-右-2-ZO-1-60s.tif]

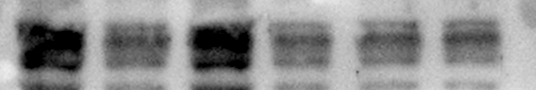

Supplement: Supplementary file 2 [file DataSheet8.zip › Fig.11/5-ZO-1/PS-左-2-ZO-1-60s.tif]

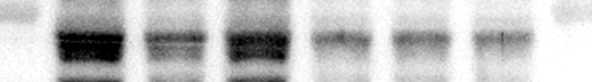

Supplement: Supplementary file 2 [file DataSheet8.zip › Fig.11/5-ZO-1/用 PS-右-3-ZO-1-60s.tif]

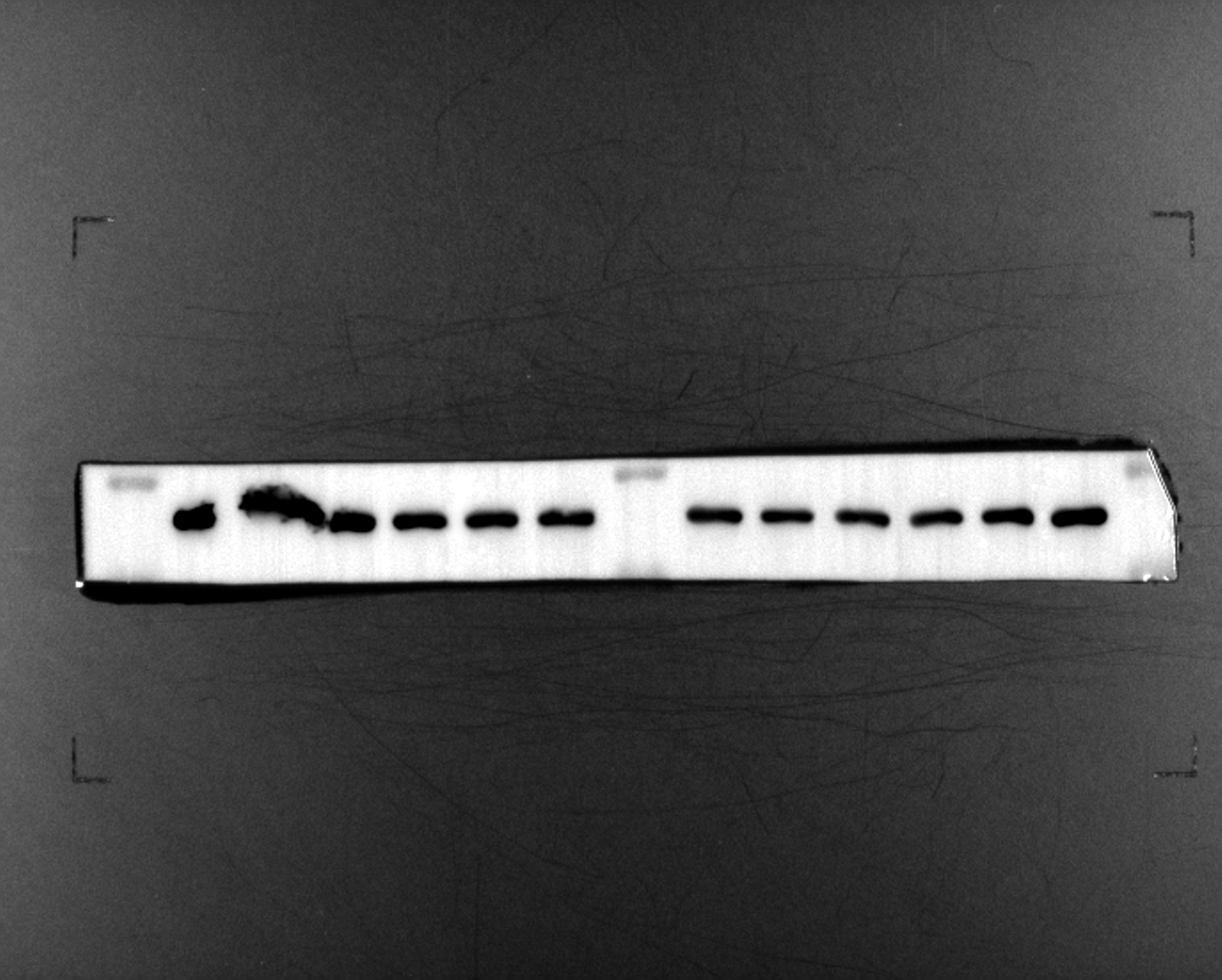

Supplement: Supplementary file 2 [file DataSheet8.zip › Fig.11/6-GAPDH/3-G-10S YT.Tif]

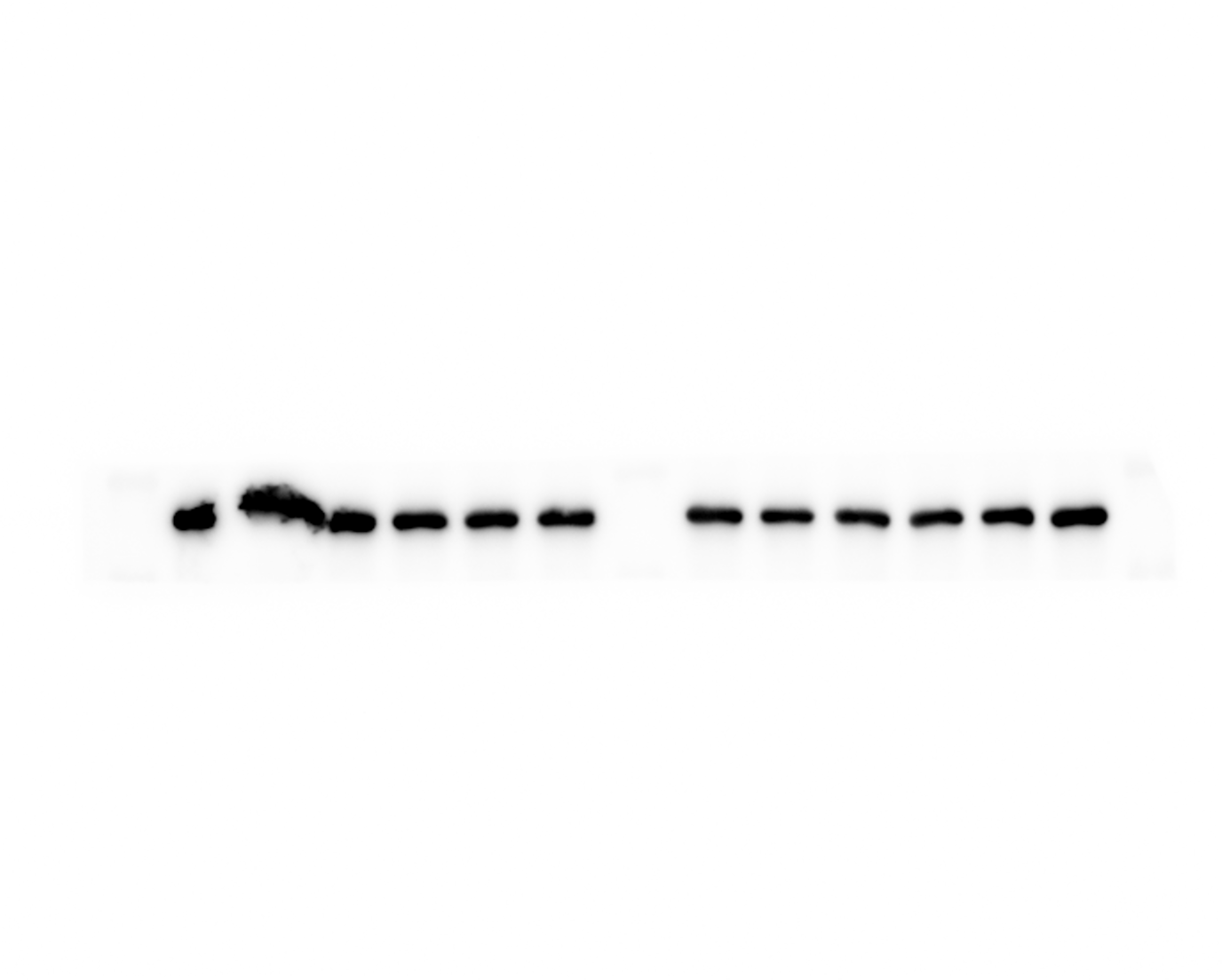

Supplement: Supplementary file 2 [file DataSheet8.zip › Fig.11/6-GAPDH/3-G-10S.Tif]

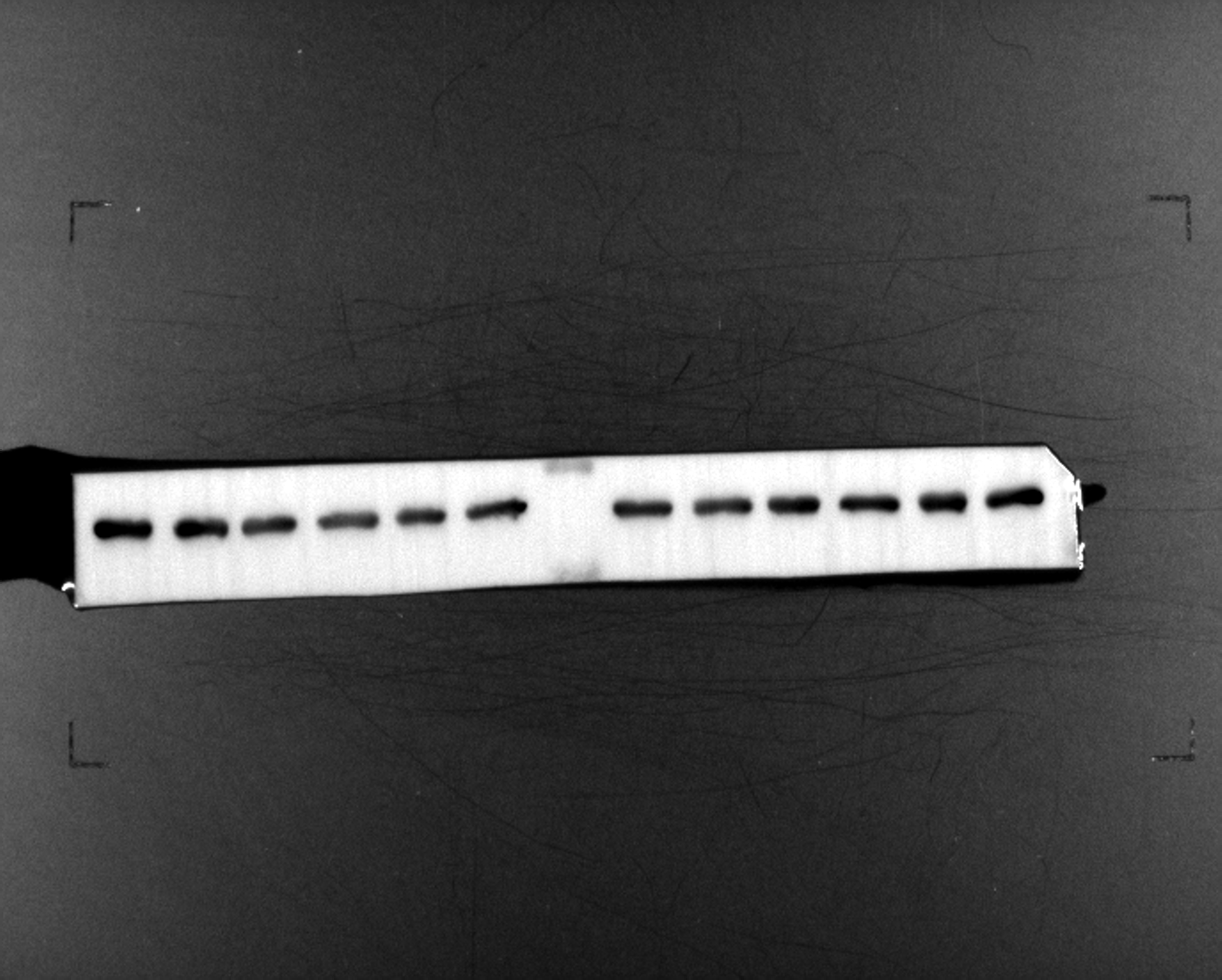

Supplement: Supplementary file 2 [file DataSheet8.zip › Fig.11/6-GAPDH/4-G-10S YT.Tif]

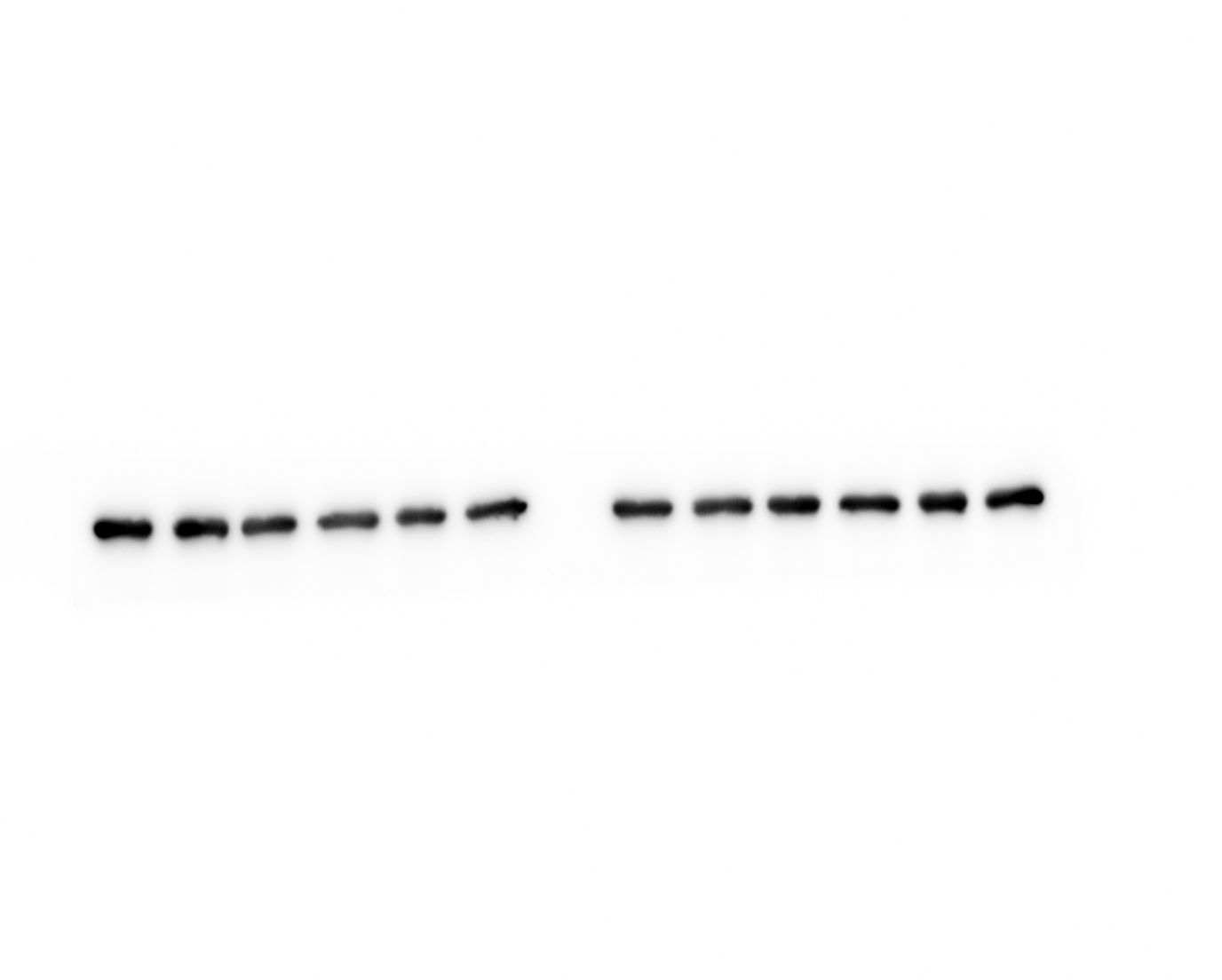

Supplement: Supplementary file 2 [file DataSheet8.zip › Fig.11/6-GAPDH/4-G-10S.Tif]

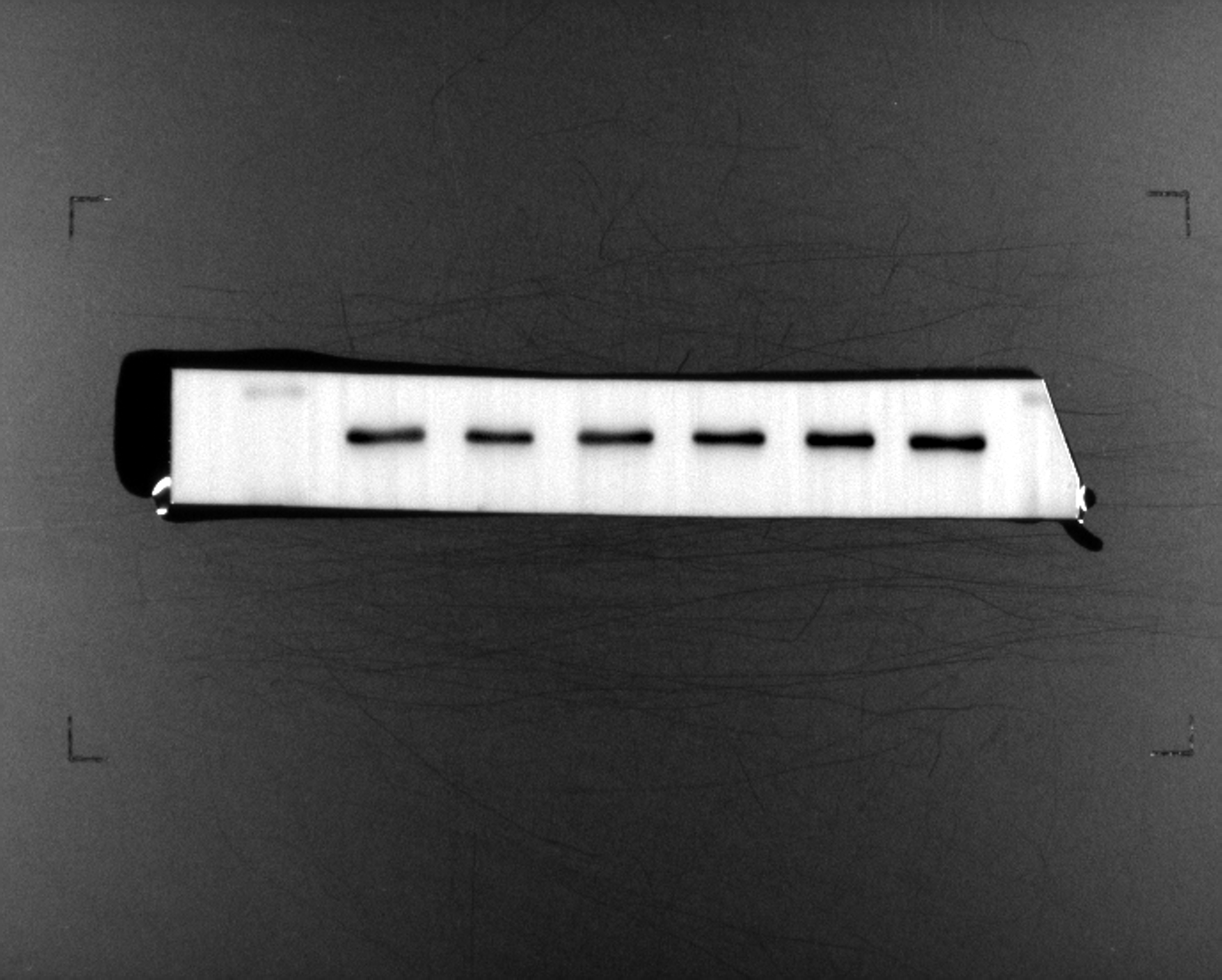

Supplement: Supplementary file 2 [file DataSheet8.zip › Fig.11/6-GAPDH/7-G-10S YT.Tif]

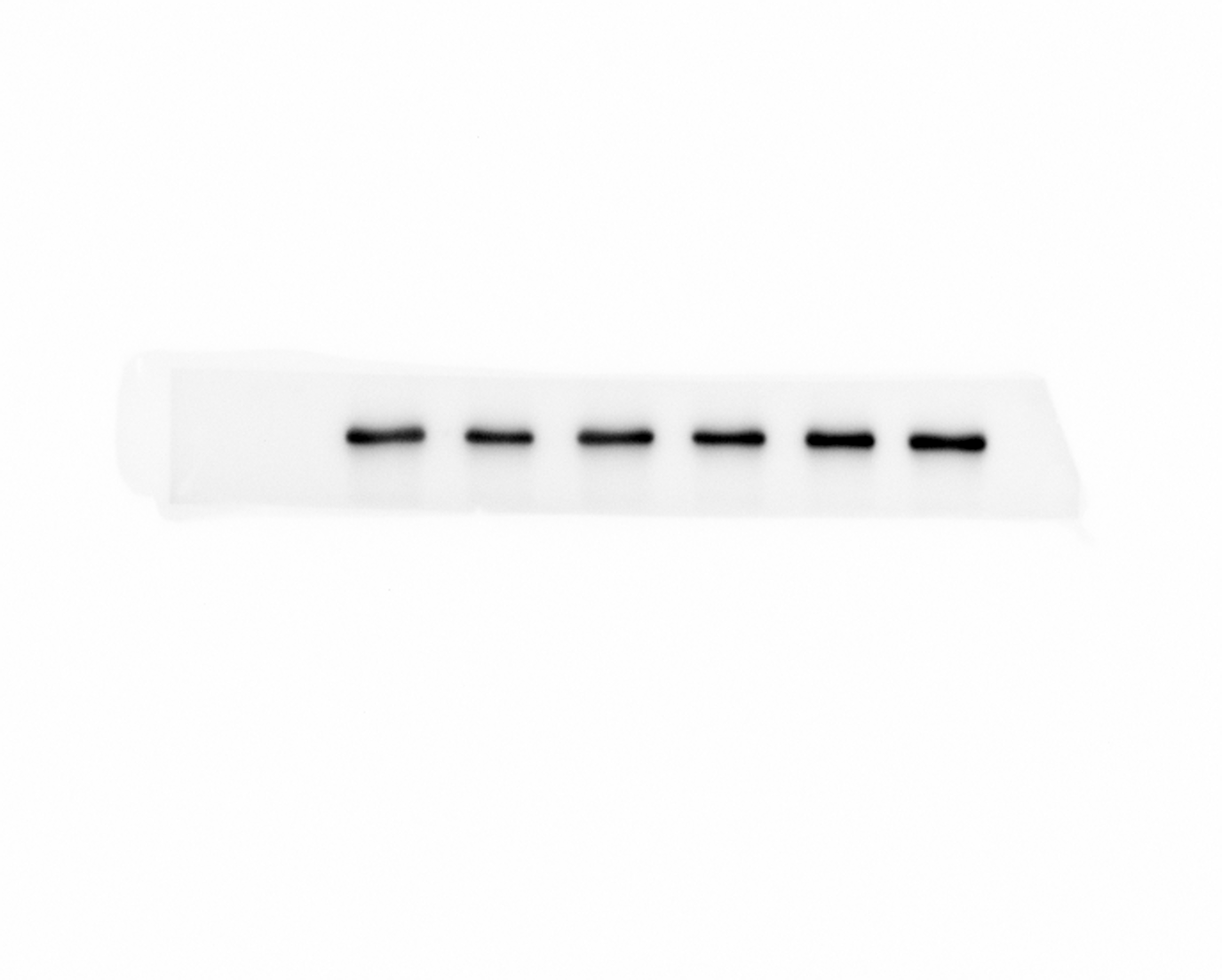

Supplement: Supplementary file 2 [file DataSheet8.zip › Fig.11/6-GAPDH/7-G-10S.Tif]

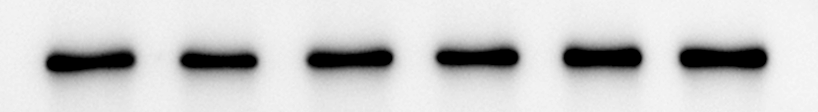

Supplement: Supplementary file 2 [file DataSheet8.zip › Fig.11/6-GAPDH/PS 7-G-10S.tif]

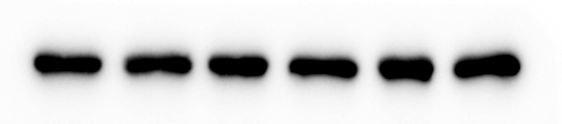

Supplement: Supplementary file 2 [file DataSheet8.zip › Fig.11/6-GAPDH/PS 右-4-G-10S.tif]

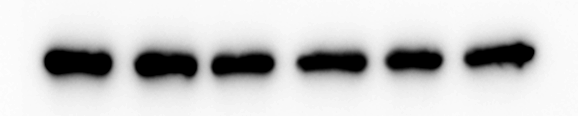

Supplement: Supplementary file 2 [file DataSheet8.zip › Fig.11/6-GAPDH/用 PS 左-4-G-10S.tif]

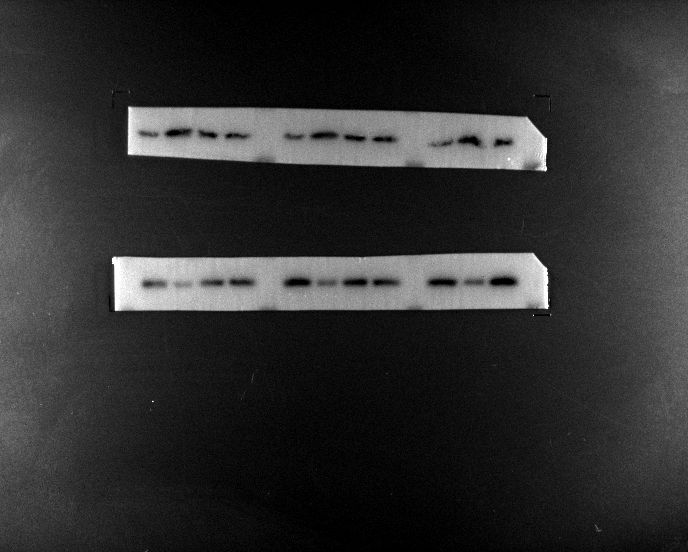

Supplement: Supplementary file 3 [file DataSheet4.zip › Fig.4/p-PI3K/2-p-PI3K YT.tif]

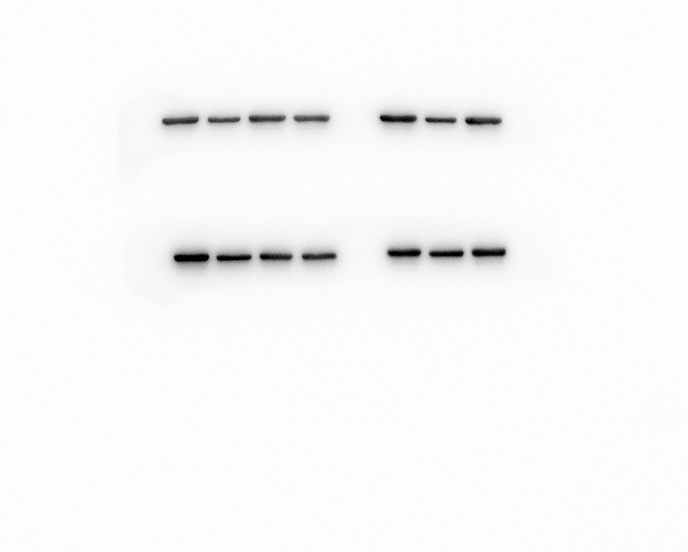

Supplement: Supplementary file 3 [file DataSheet4.zip › Fig.4/p-PI3K/3-p-PI3K.tif]

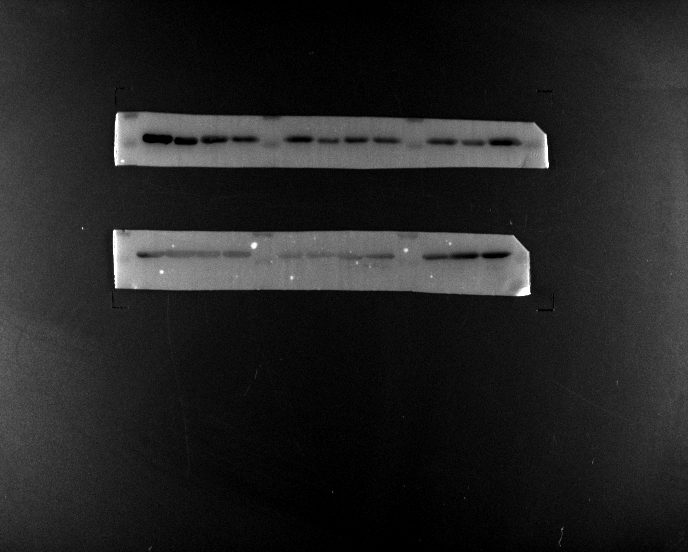

Supplement: Supplementary file 3 [file DataSheet4.zip › Fig.4/p-PI3K/1-p-PI3K YT.tif]

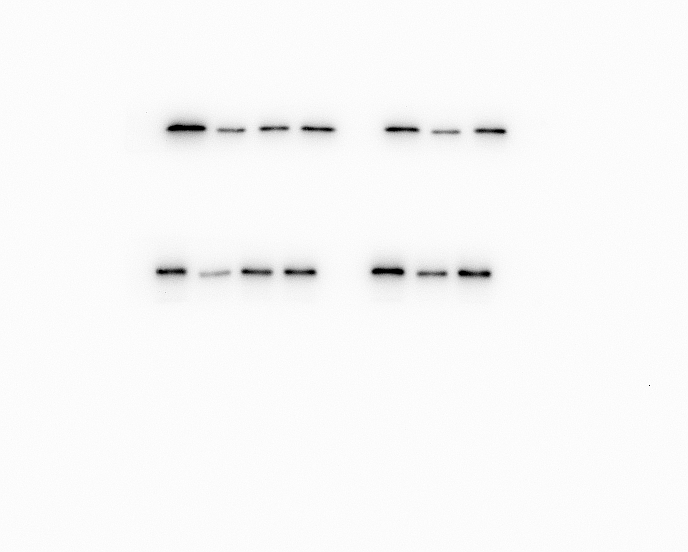

Supplement: Supplementary file 3 [file DataSheet4.zip › Fig.4/p-PI3K/4-p-PI3K.tif]

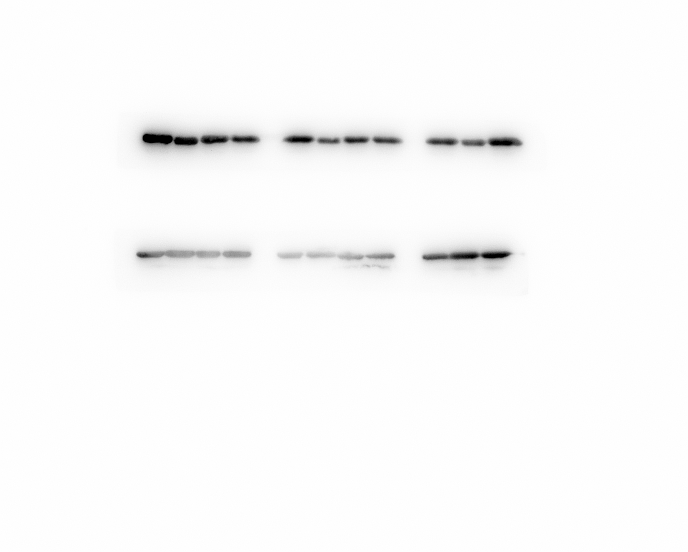

Supplement: Supplementary file 3 [file DataSheet4.zip › Fig.4/p-PI3K/1-p-PI3K.tif]

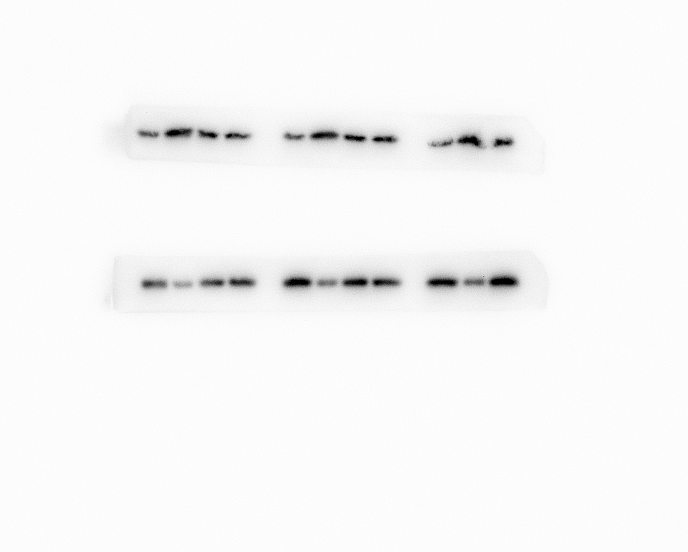

Supplement: Supplementary file 3 [file DataSheet4.zip › Fig.4/p-PI3K/2-p-PI3K.tif]

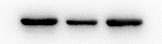

Supplement: Supplementary file 3 [file DataSheet4.zip › Fig.4/p-PI3K/3.tif]

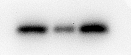

Supplement: Supplementary file 3 [file DataSheet4.zip › Fig.4/p-PI3K/2.tif]

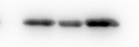

Supplement: Supplementary file 3 [file DataSheet4.zip › Fig.4/p-PI3K/1.tif]

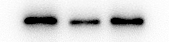

Supplement: Supplementary file 3 [file DataSheet4.zip › Fig.4/p-PI3K/4.tif]

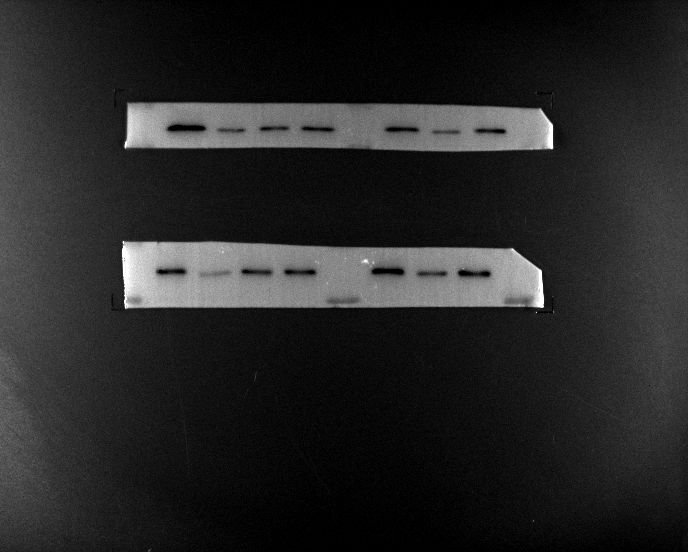

Supplement: Supplementary file 3 [file DataSheet4.zip › Fig.4/p-PI3K/4-p-PI3K YT.tif]

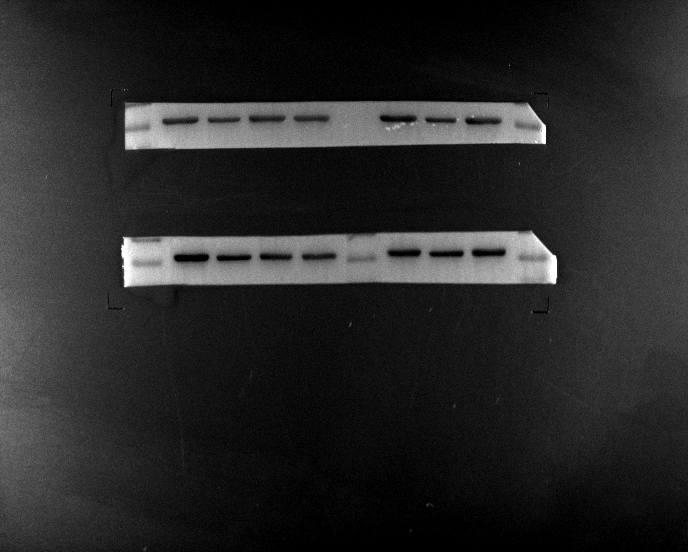

Supplement: Supplementary file 3 [file DataSheet4.zip › Fig.4/p-PI3K/3-p-PI3K YT.tif]

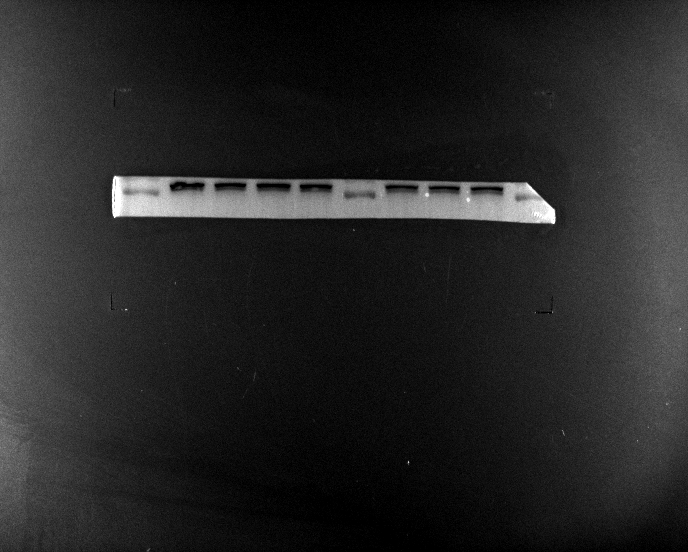

Supplement: Supplementary file 3 [file DataSheet4.zip › Fig.4/PI3K/3-PI3K YT.tif]

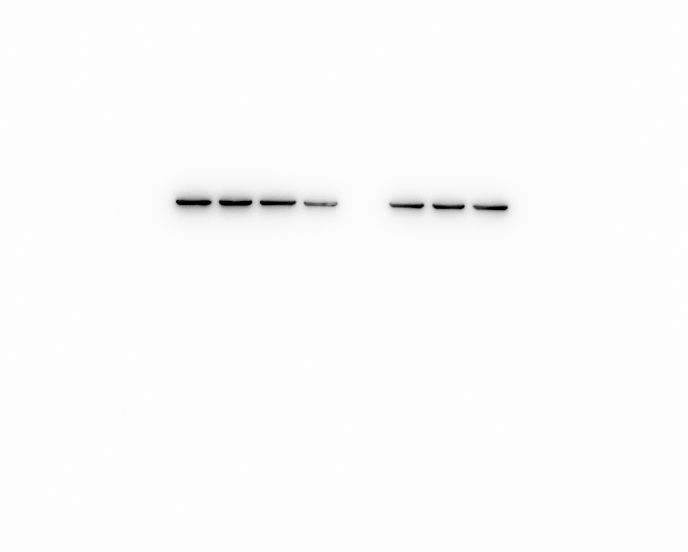

Supplement: Supplementary file 3 [file DataSheet4.zip › Fig.4/PI3K/2-PI3K.tif]

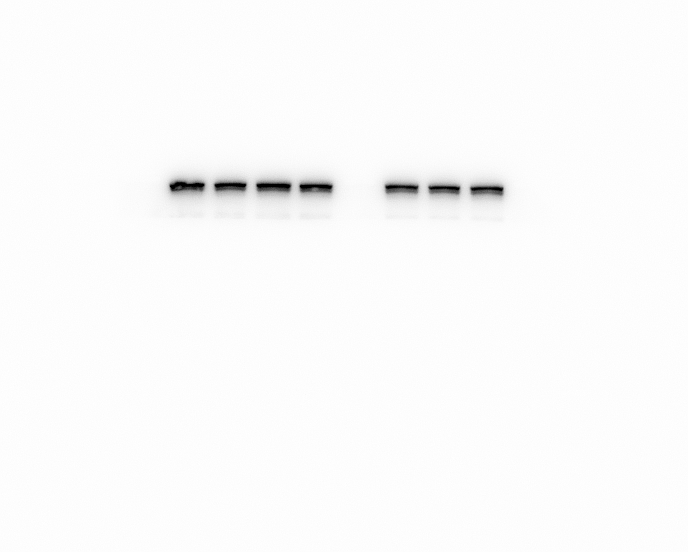

Supplement: Supplementary file 3 [file DataSheet4.zip › Fig.4/PI3K/3-PI3K.tif]

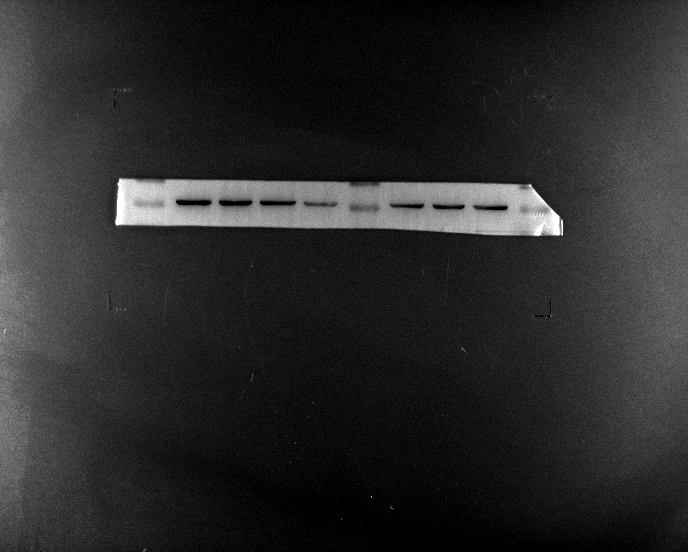

Supplement: Supplementary file 3 [file DataSheet4.zip › Fig.4/PI3K/2-PI3K YT.tif]

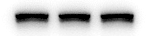

Supplement: Supplementary file 3 [file DataSheet4.zip › Fig.4/PI3K/3.tif]

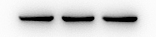

Supplement: Supplementary file 3 [file DataSheet4.zip › Fig.4/PI3K/2.tif]

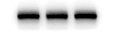

Supplement: Supplementary file 3 [file DataSheet4.zip › Fig.4/PI3K/1.tif]

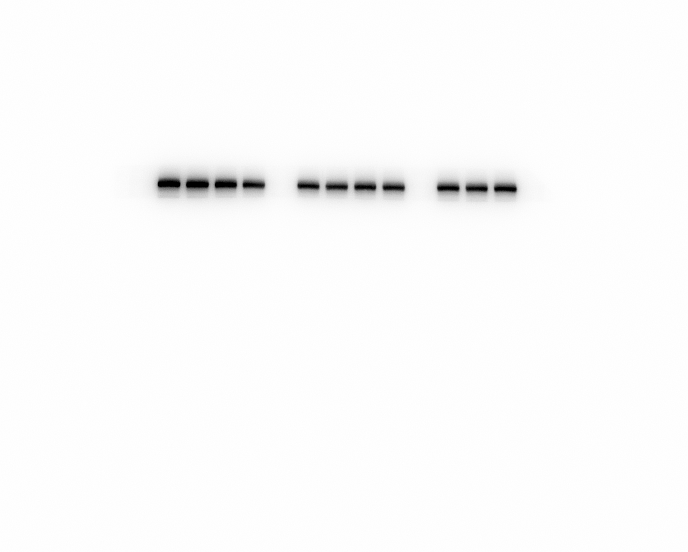

Supplement: Supplementary file 3 [file DataSheet4.zip › Fig.4/PI3K/1-PI3K.tif]

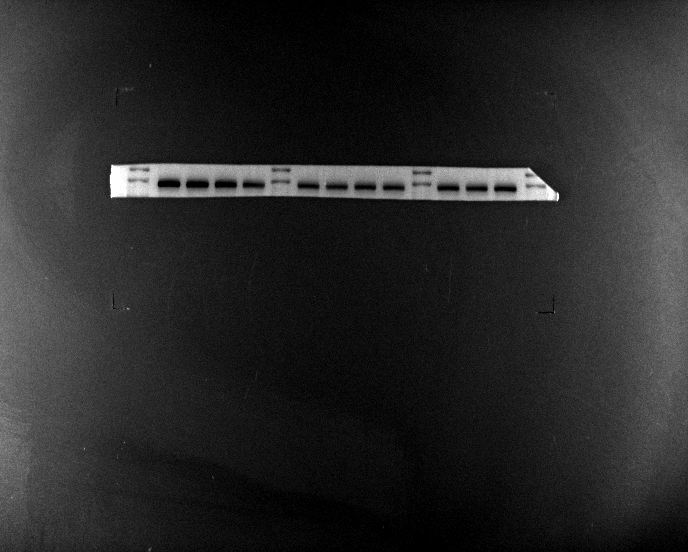

Supplement: Supplementary file 3 [file DataSheet4.zip › Fig.4/PI3K/1-PI3K YT.tif]

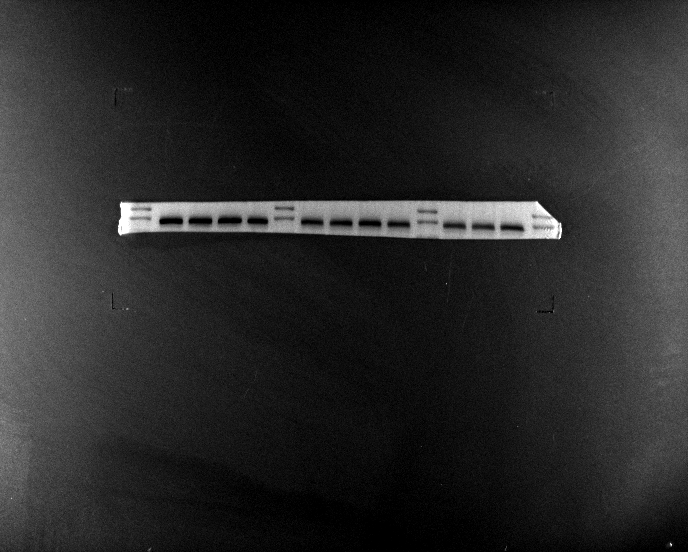

Supplement: Supplementary file 3 [file DataSheet4.zip › Fig.4/AKT/3-AKT YT.tif]

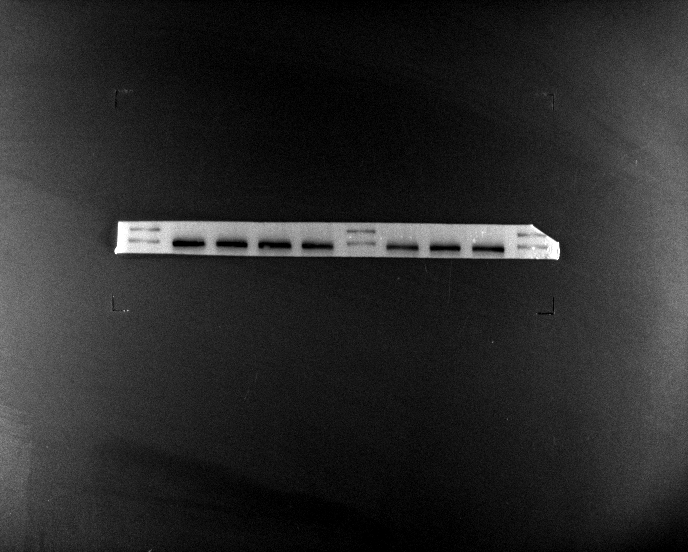

Supplement: Supplementary file 3 [file DataSheet4.zip › Fig.4/AKT/1-AKT YT.tif]

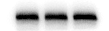

Supplement: Supplementary file 3 [file DataSheet4.zip › Fig.4/AKT/3.tif]

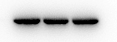

Supplement: Supplementary file 3 [file DataSheet4.zip › Fig.4/AKT/2.tif]

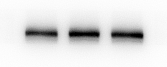

Supplement: Supplementary file 3 [file DataSheet4.zip › Fig.4/AKT/1.tif]

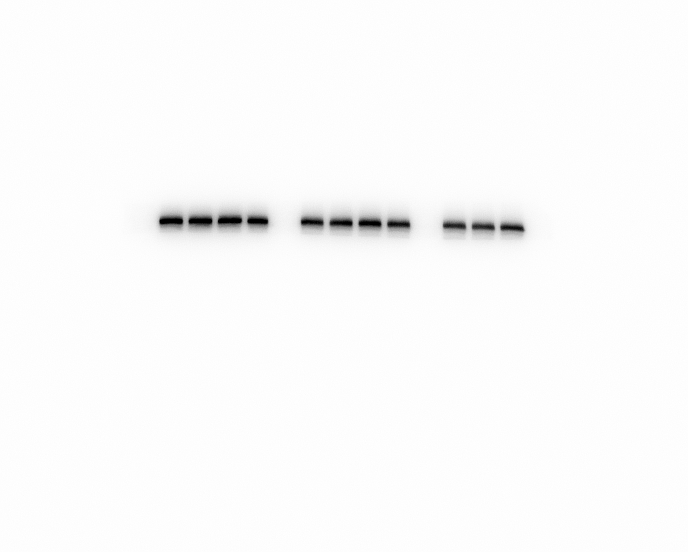

Supplement: Supplementary file 3 [file DataSheet4.zip › Fig.4/AKT/3-AKT.tif]

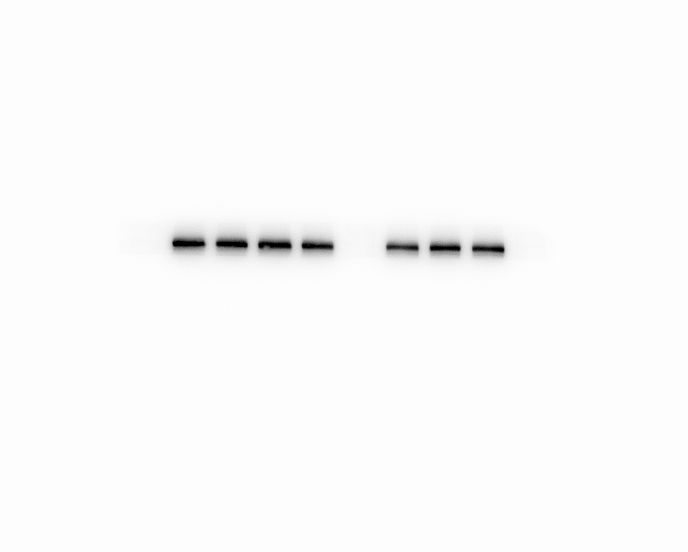

Supplement: Supplementary file 3 [file DataSheet4.zip › Fig.4/AKT/1-AKT.tif]

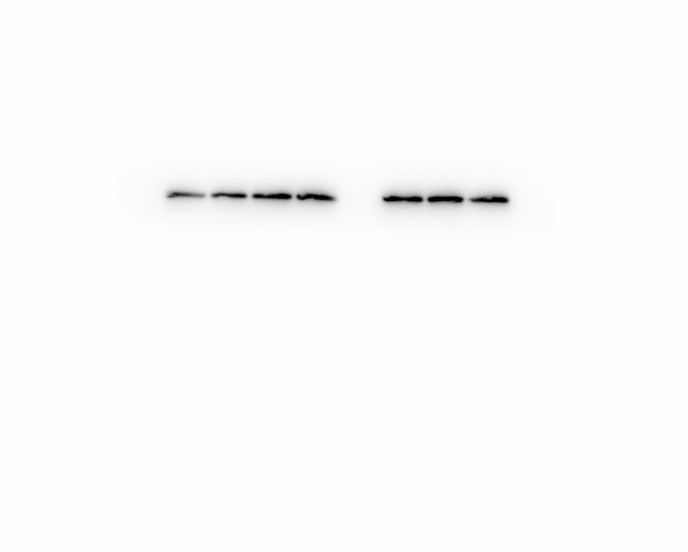

Supplement: Supplementary file 3 [file DataSheet4.zip › Fig.4/GAPDH/3-GAPDH.tif]

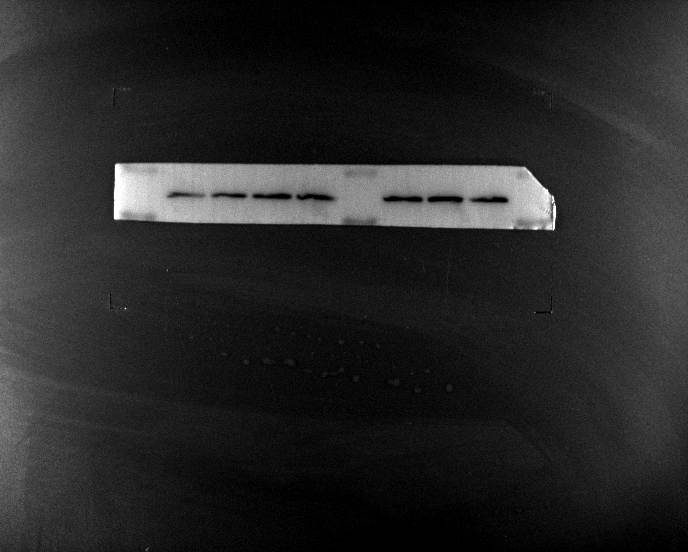

Supplement: Supplementary file 3 [file DataSheet4.zip › Fig.4/GAPDH/3-GAPDH YT.tif]

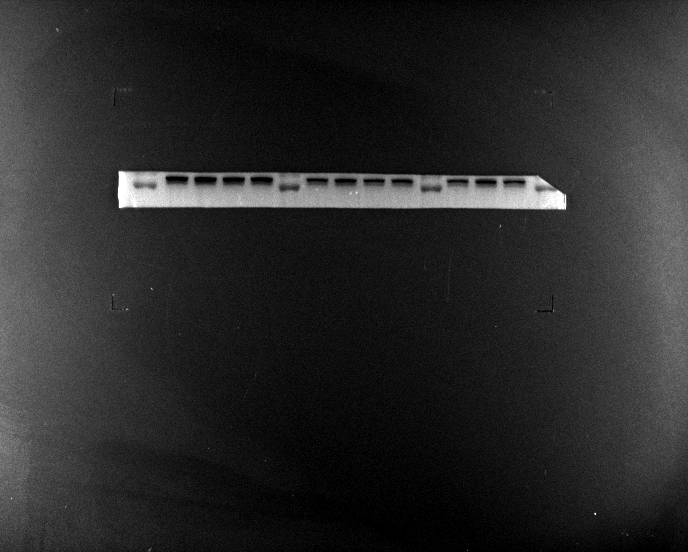

Supplement: Supplementary file 3 [file DataSheet4.zip › Fig.4/GAPDH/2-GAPDH YT.tif]

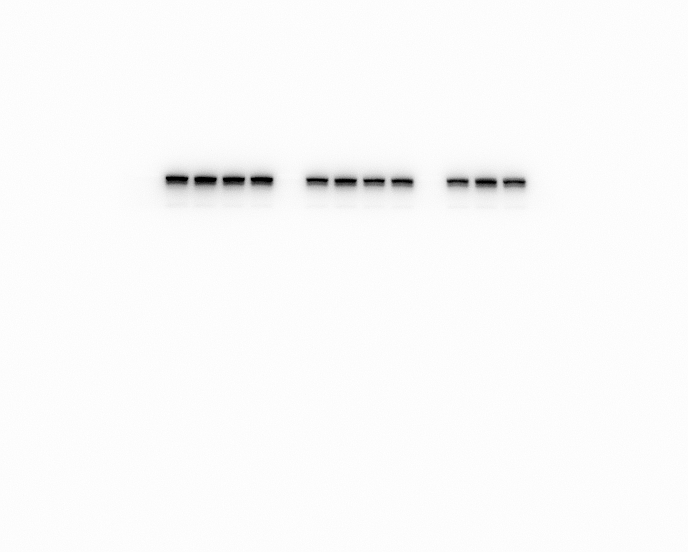

Supplement: Supplementary file 3 [file DataSheet4.zip › Fig.4/GAPDH/2-GAPDH.tif]

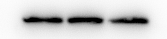

Supplement: Supplementary file 3 [file DataSheet4.zip › Fig.4/GAPDH/3.tif]

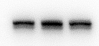

Supplement: Supplementary file 3 [file DataSheet4.zip › Fig.4/GAPDH/2.tif]

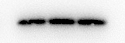

Supplement: Supplementary file 3 [file DataSheet4.zip › Fig.4/GAPDH/1.tif]

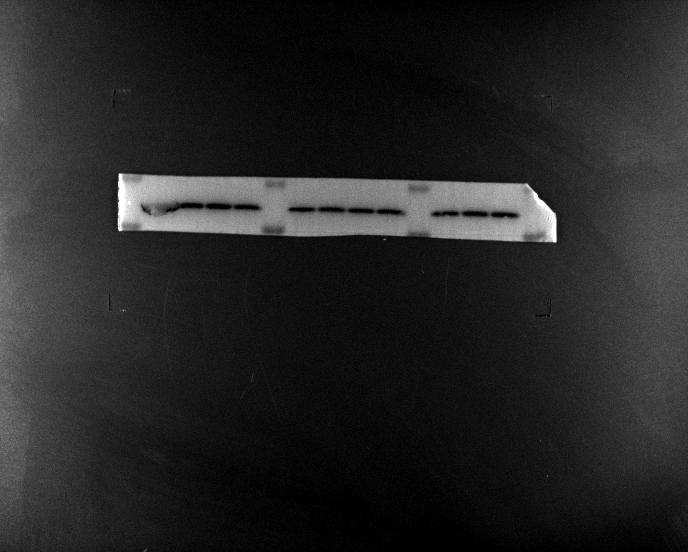

Supplement: Supplementary file 3 [file DataSheet4.zip › Fig.4/GAPDH/1-GAPDH YT.tif]

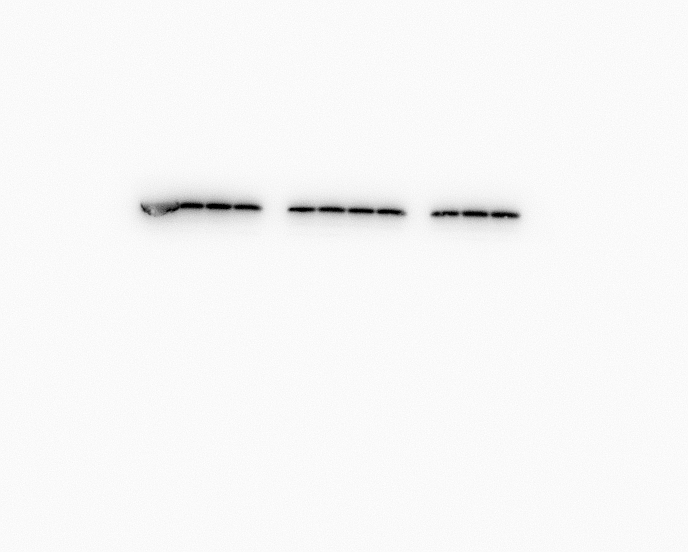

Supplement: Supplementary file 3 [file DataSheet4.zip › Fig.4/GAPDH/1-GAPDH.tif]

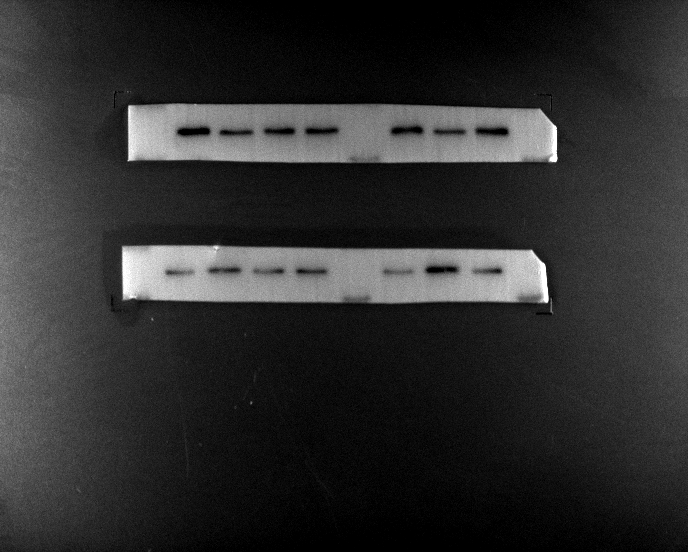

Supplement: Supplementary file 3 [file DataSheet4.zip › Fig.4/p-AKT/3-p-AKT YT.tif]

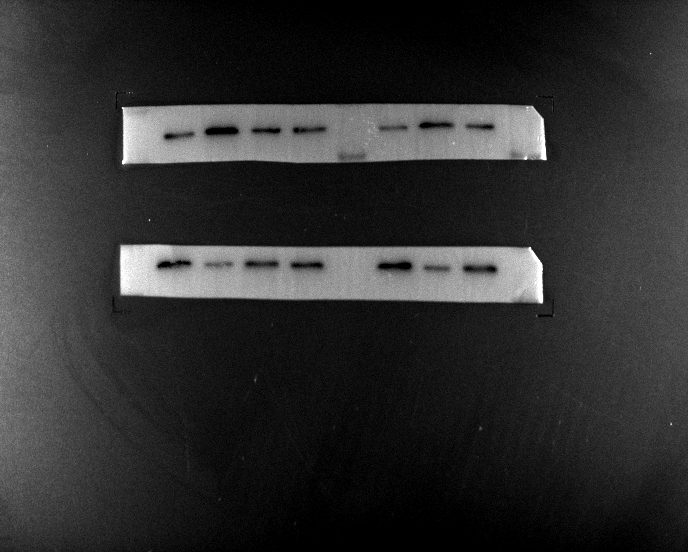

Supplement: Supplementary file 3 [file DataSheet4.zip › Fig.4/p-AKT/2-p-AKT YT.tif]

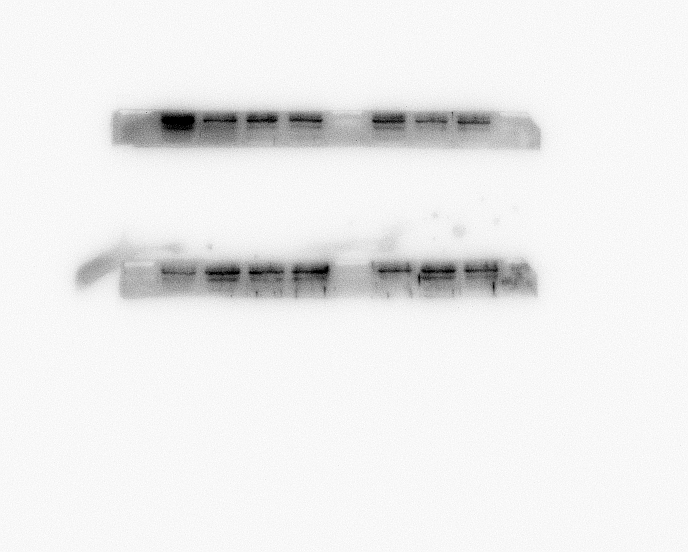

Supplement: Supplementary file 3 [file DataSheet4.zip › Fig.4/p-AKT/1-p-AKT.tif]

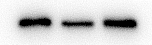

Supplement: Supplementary file 3 [file DataSheet4.zip › Fig.4/p-AKT/3.tif]

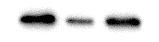

Supplement: Supplementary file 3 [file DataSheet4.zip › Fig.4/p-AKT/2.tif]

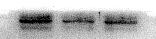

Supplement: Supplementary file 3 [file DataSheet4.zip › Fig.4/p-AKT/1.tif]

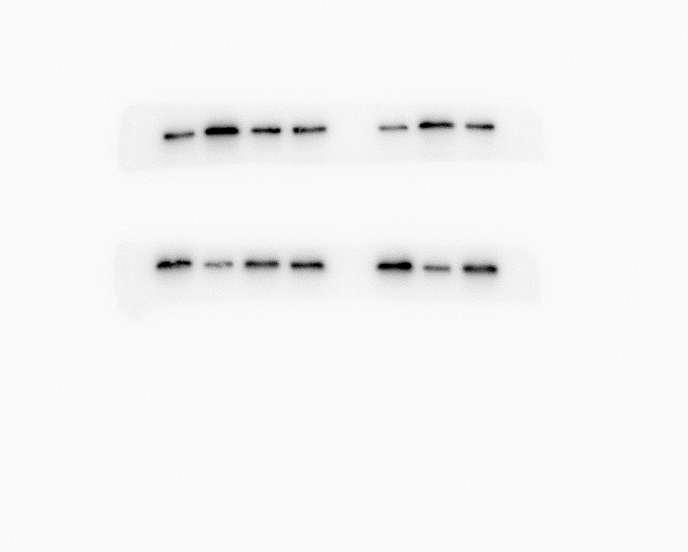

Supplement: Supplementary file 3 [file DataSheet4.zip › Fig.4/p-AKT/2-p-AKT.tif]

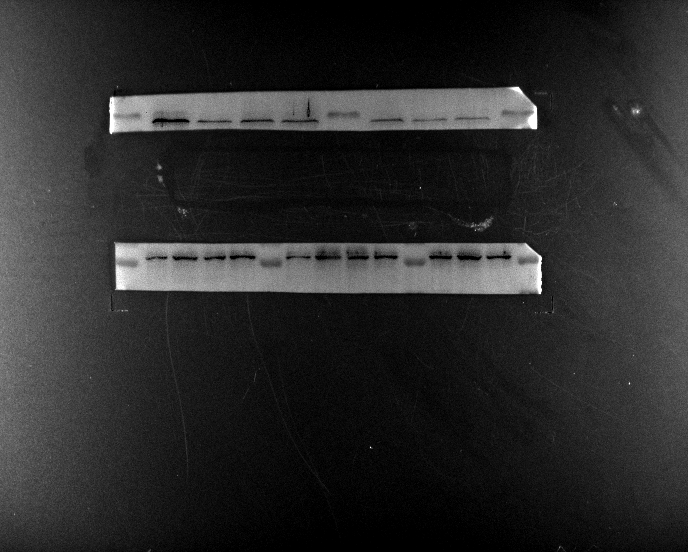

Supplement: Supplementary file 3 [file DataSheet4.zip › Fig.4/p-AKT/1-p-AKT YT.tif]

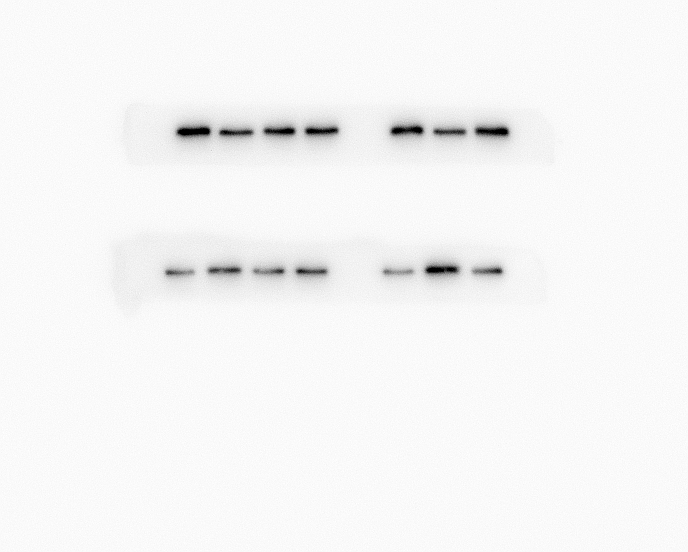

Supplement: Supplementary file 3 [file DataSheet4.zip › Fig.4/p-AKT/3-p-AKT.tif]

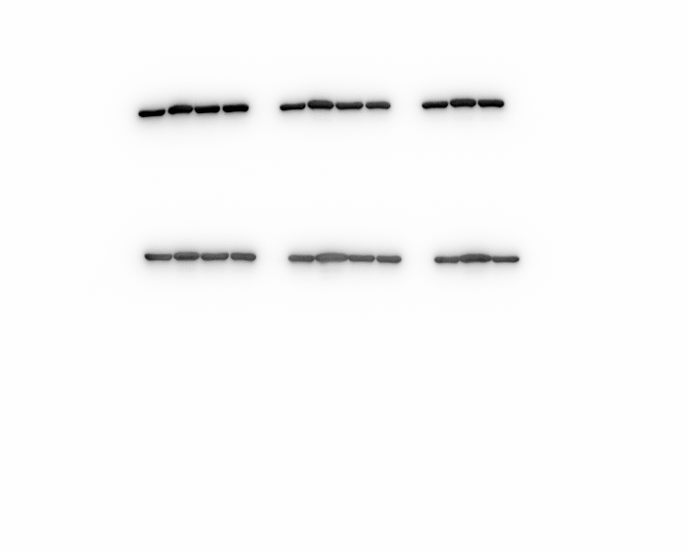

Supplement: Supplementary file 3 [file DataSheet4.zip › Fig.5/ASC/ASC.tif]
