# Supplementary figures and images for: Total Flavones of Abelmoschus manihot Ameliorates Podocyte Pyroptosis and Injury in High Glucose Conditions by Targeting METTL3-Dependent m6A Modification-Mediated NLRP3-Inflammasome Activation and PTEN/PI3K/Akt Signaling (part 3 of 6)
Source: Front Pharmacol. 2021 Jul 15;12:667644. doi: 10.3389/fphar.2021.667644 (PMC8319635; doi:10.3389/fphar.2021.667644)

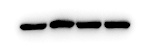

Supplement: Supplementary file 3 [file DataSheet4.zip › Fig.5/ASC/3.tif]

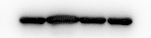

Supplement: Supplementary file 3 [file DataSheet4.zip › Fig.5/ASC/2.tif]

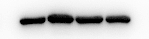

Supplement: Supplementary file 3 [file DataSheet4.zip › Fig.5/ASC/1.tif]

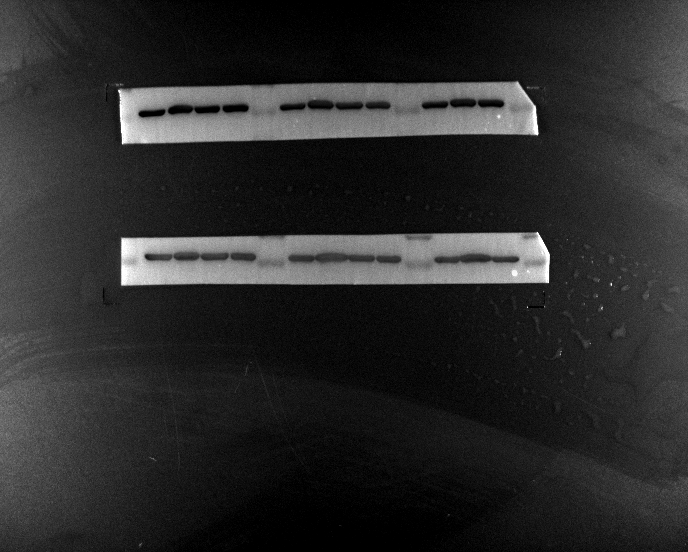

Supplement: Supplementary file 3 [file DataSheet4.zip › Fig.5/ASC/ASC YT.tif]

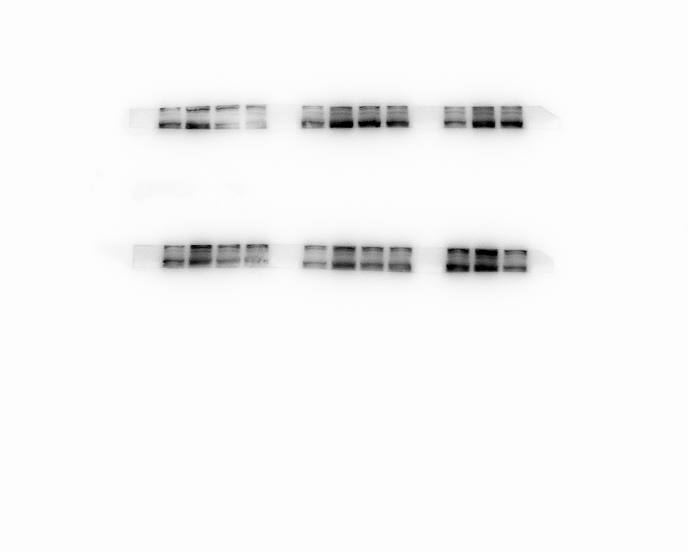

Supplement: Supplementary file 3 [file DataSheet4.zip › Fig.5/NLRP3/1-2-NLRP3.tif]

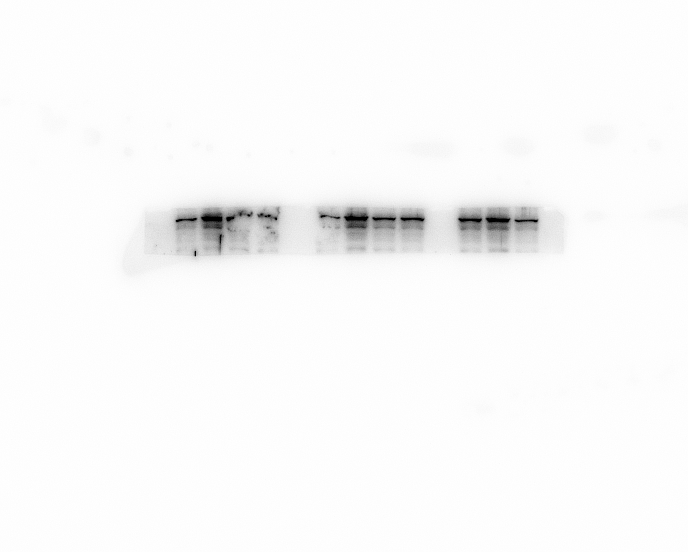

Supplement: Supplementary file 3 [file DataSheet4.zip › Fig.5/NLRP3/3-NLRP3.tif]

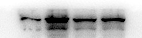

Supplement: Supplementary file 3 [file DataSheet4.zip › Fig.5/NLRP3/3.tif]

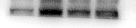

Supplement: Supplementary file 3 [file DataSheet4.zip › Fig.5/NLRP3/2.tif]

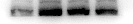

Supplement: Supplementary file 3 [file DataSheet4.zip › Fig.5/NLRP3/1.tif]

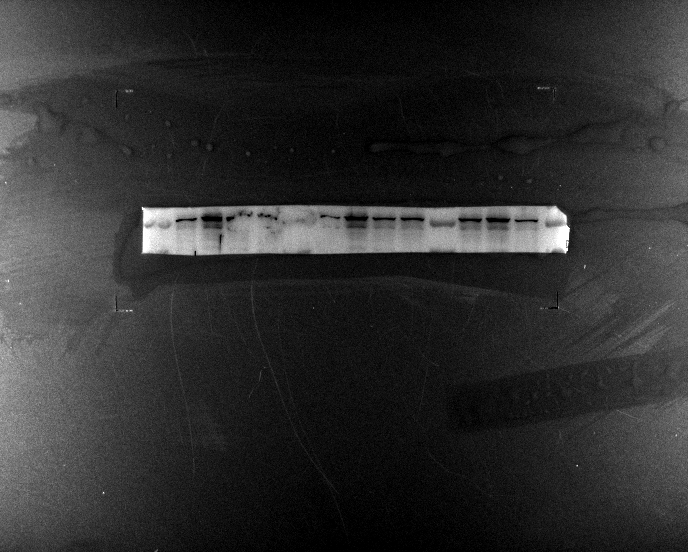

Supplement: Supplementary file 3 [file DataSheet4.zip › Fig.5/NLRP3/3-NLRP3 YT.tif]

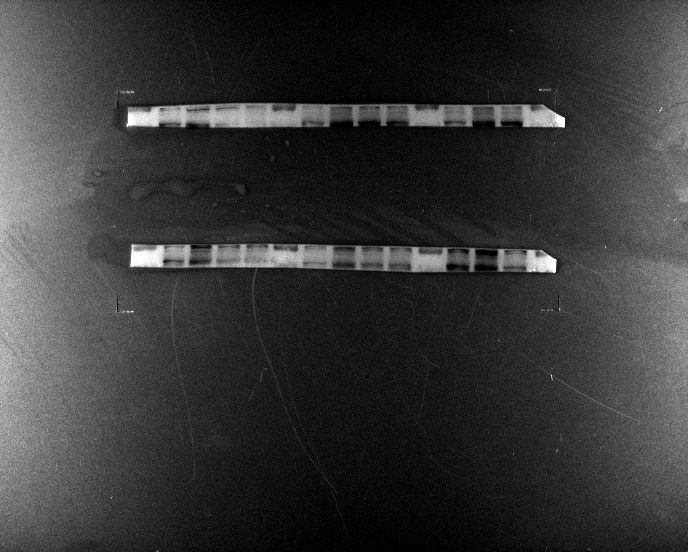

Supplement: Supplementary file 3 [file DataSheet4.zip › Fig.5/NLRP3/1-2-NLRP3 YT.tif]

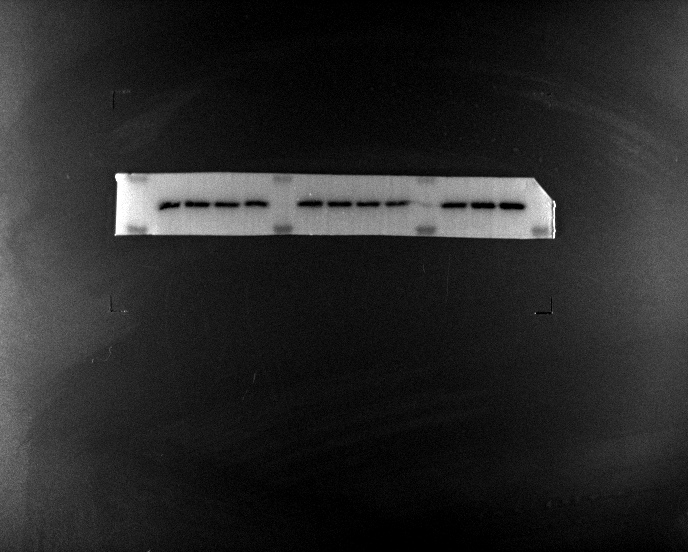

Supplement: Supplementary file 3 [file DataSheet4.zip › Fig.5/C-GAPDH/2-3-GAPDH YT.tif]

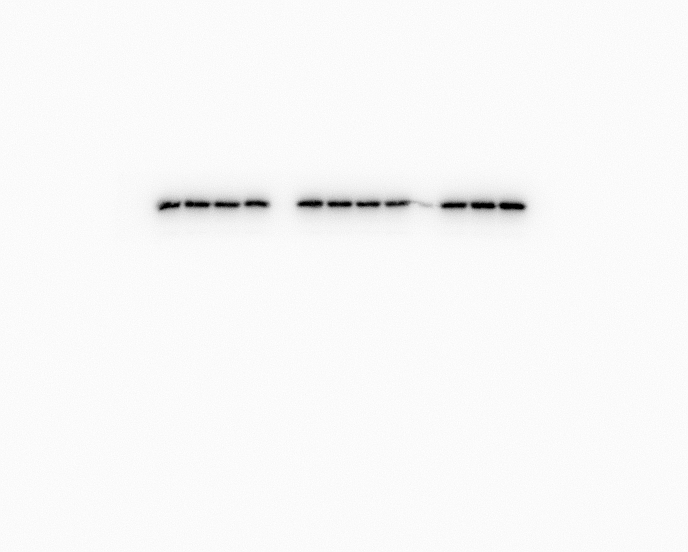

Supplement: Supplementary file 3 [file DataSheet4.zip › Fig.5/C-GAPDH/2-3-GAPDH.tif]

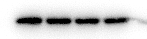

Supplement: Supplementary file 3 [file DataSheet4.zip › Fig.5/C-GAPDH/3.tif]

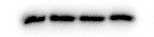

Supplement: Supplementary file 3 [file DataSheet4.zip › Fig.5/C-GAPDH/2.tif]

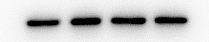

Supplement: Supplementary file 3 [file DataSheet4.zip › Fig.5/C-GAPDH/1.tif]

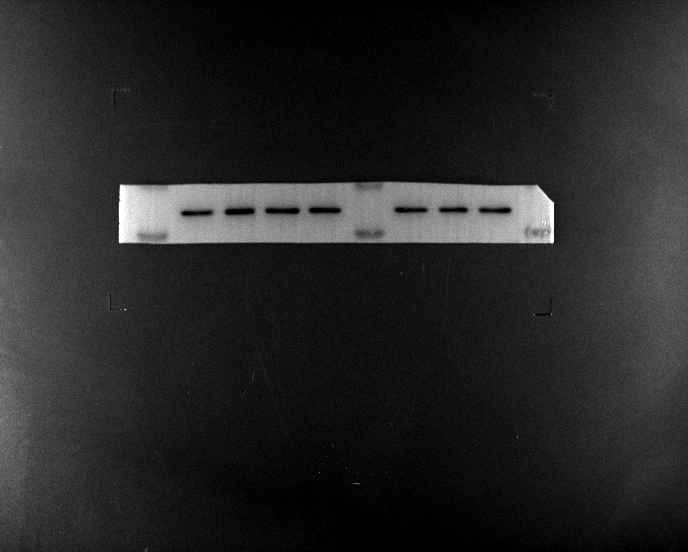

Supplement: Supplementary file 3 [file DataSheet4.zip › Fig.5/C-GAPDH/1-GAPDH YT.tif]

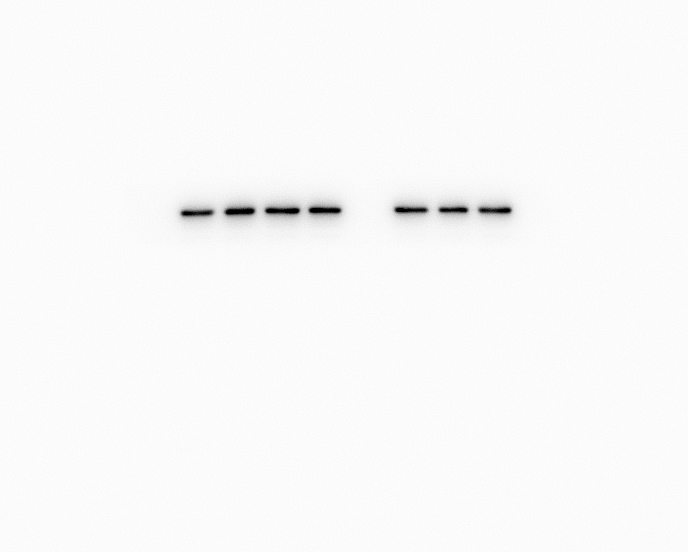

Supplement: Supplementary file 3 [file DataSheet4.zip › Fig.5/C-GAPDH/1-GAPDH.tif]

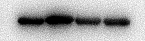

Supplement: Supplementary file 3 [file DataSheet4.zip › Fig.5/NEK7/3.tif]

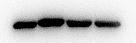

Supplement: Supplementary file 3 [file DataSheet4.zip › Fig.5/NEK7/2.tif]

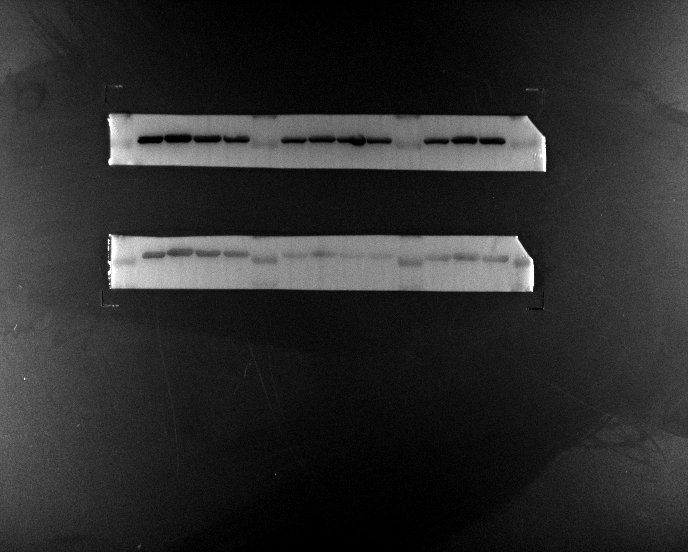

Supplement: Supplementary file 3 [file DataSheet4.zip › Fig.5/NEK7/NEK7 YT.tif]

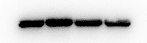

Supplement: Supplementary file 3 [file DataSheet4.zip › Fig.5/NEK7/1.tif]

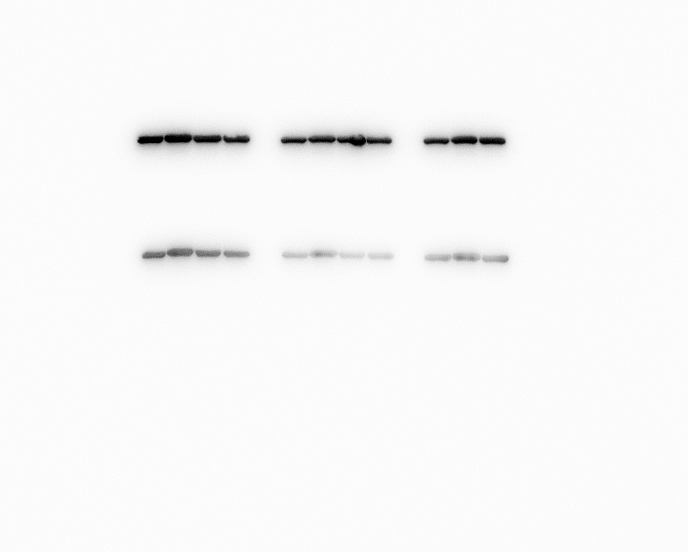

Supplement: Supplementary file 3 [file DataSheet4.zip › Fig.5/NEK7/NEK7.tif]

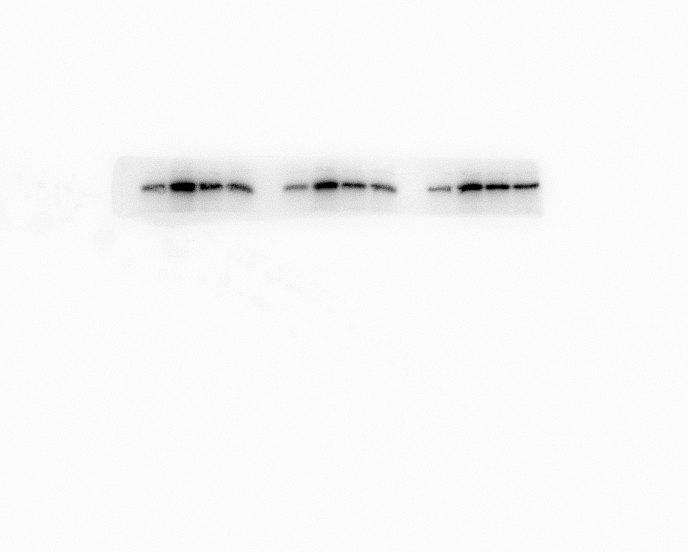

Supplement: Supplementary file 3 [file DataSheet4.zip › Fig.5/Caspase-1/Caspase 1.tif]

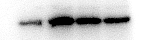

Supplement: Supplementary file 3 [file DataSheet4.zip › Fig.5/Caspase-1/3.tif]

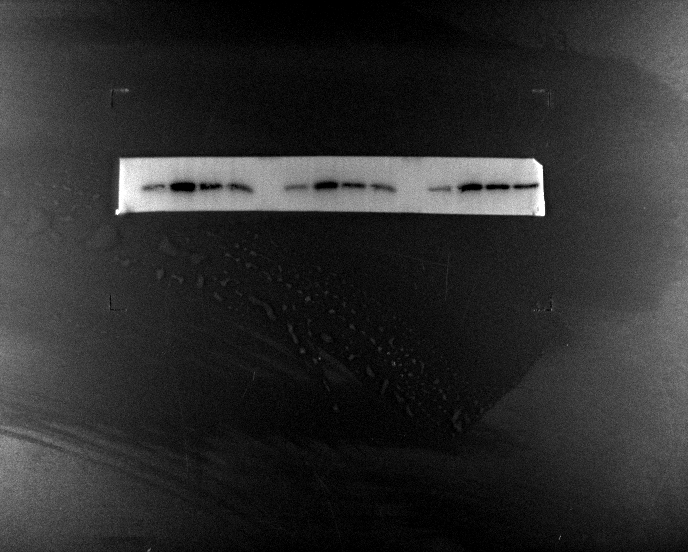

Supplement: Supplementary file 3 [file DataSheet4.zip › Fig.5/Caspase-1/Caspase 1 YT.tif]

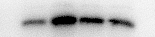

Supplement: Supplementary file 3 [file DataSheet4.zip › Fig.5/Caspase-1/2.tif]

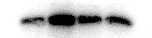

Supplement: Supplementary file 3 [file DataSheet4.zip › Fig.5/Caspase-1/1.tif]

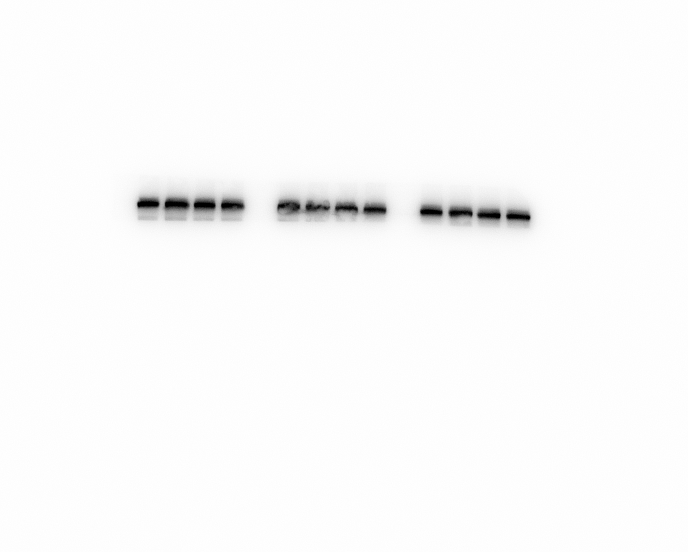

Supplement: Supplementary file 3 [file DataSheet4.zip › Fig.6/2-PI3K/PI3K.tif]

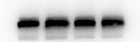

Supplement: Supplementary file 3 [file DataSheet4.zip › Fig.6/2-PI3K/3.tif]

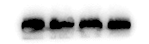

Supplement: Supplementary file 3 [file DataSheet4.zip › Fig.6/2-PI3K/2.tif]

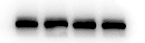

Supplement: Supplementary file 3 [file DataSheet4.zip › Fig.6/2-PI3K/1.tif]

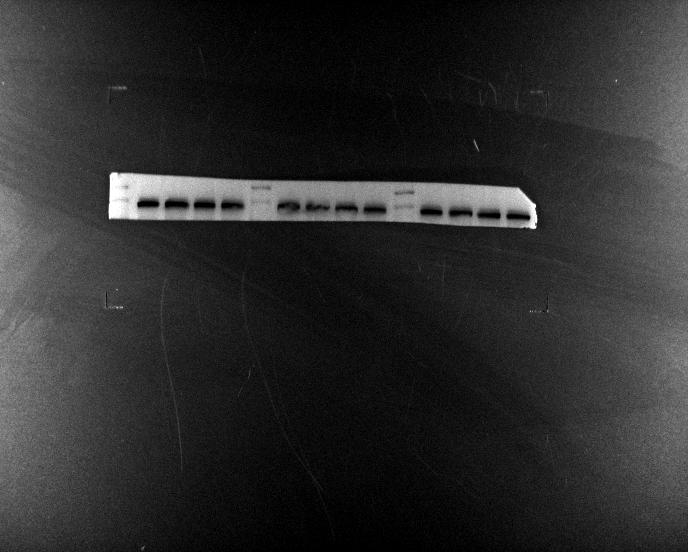

Supplement: Supplementary file 3 [file DataSheet4.zip › Fig.6/2-PI3K/PI3K YT.tif]

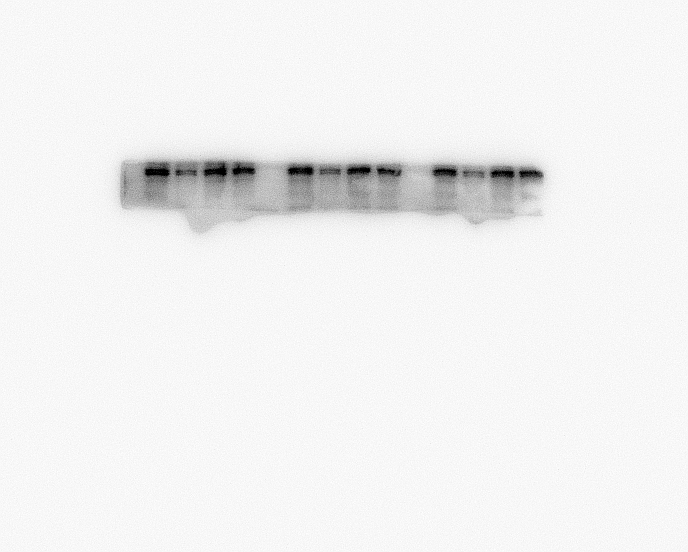

Supplement: Supplementary file 3 [file DataSheet4.zip › Fig.6/1-p-PI3K/PI3K.tif]

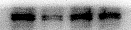

Supplement: Supplementary file 3 [file DataSheet4.zip › Fig.6/1-p-PI3K/3.tif]

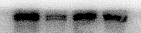

Supplement: Supplementary file 3 [file DataSheet4.zip › Fig.6/1-p-PI3K/2.tif]

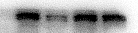

Supplement: Supplementary file 3 [file DataSheet4.zip › Fig.6/1-p-PI3K/1.tif]

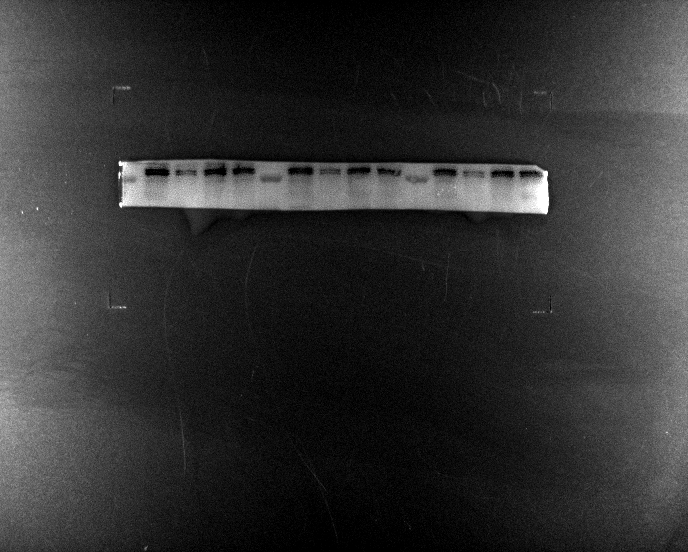

Supplement: Supplementary file 3 [file DataSheet4.zip › Fig.6/1-p-PI3K/PI3K YT.tif]

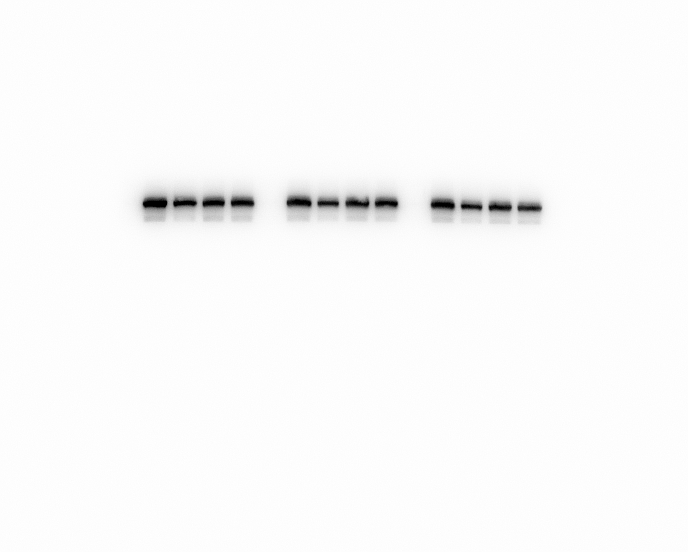

Supplement: Supplementary file 3 [file DataSheet4.zip › Fig.6/3-P-AKT/p-AKT.tif]

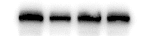

Supplement: Supplementary file 3 [file DataSheet4.zip › Fig.6/3-P-AKT/3.tif]

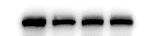

Supplement: Supplementary file 3 [file DataSheet4.zip › Fig.6/3-P-AKT/2.tif]

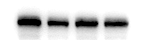

Supplement: Supplementary file 3 [file DataSheet4.zip › Fig.6/3-P-AKT/1.tif]

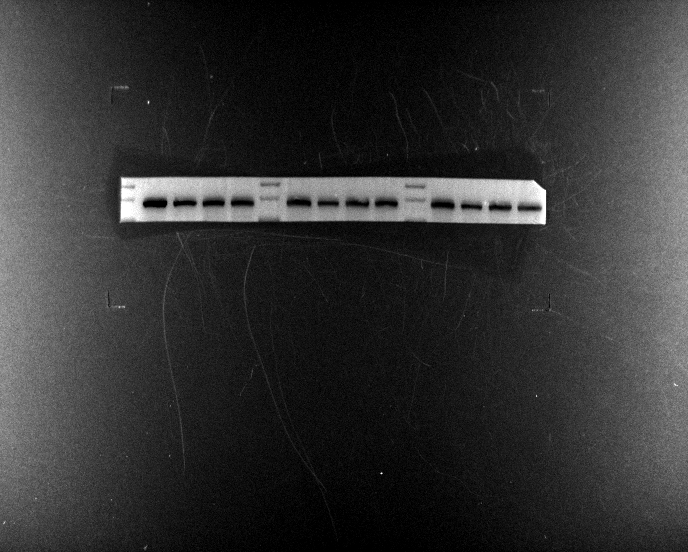

Supplement: Supplementary file 3 [file DataSheet4.zip › Fig.6/3-P-AKT/p-AKT YT.tif]

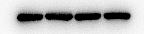

Supplement: Supplementary file 3 [file DataSheet4.zip › Fig.6/4-AKT/3.tif]

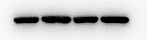

Supplement: Supplementary file 3 [file DataSheet4.zip › Fig.6/4-AKT/2.tif]

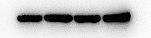

Supplement: Supplementary file 3 [file DataSheet4.zip › Fig.6/4-AKT/1.tif]

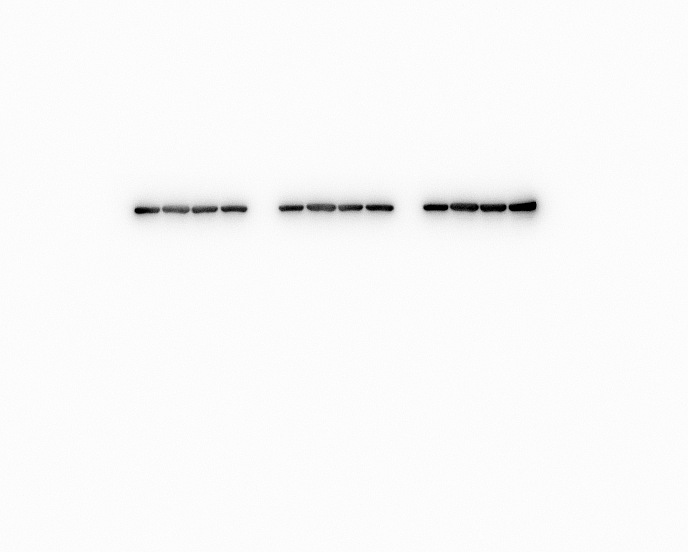

Supplement: Supplementary file 3 [file DataSheet4.zip › Fig.6/4-AKT/AKT.tif]

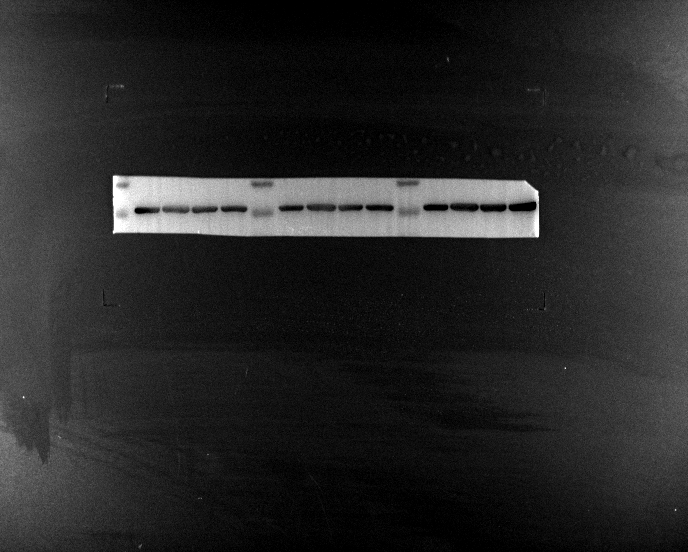

Supplement: Supplementary file 3 [file DataSheet4.zip › Fig.6/4-AKT/AKT YT.tif]

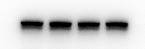

Supplement: Supplementary file 3 [file DataSheet4.zip › Fig.6/5-GAPDH/3.tif]

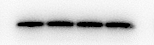

Supplement: Supplementary file 3 [file DataSheet4.zip › Fig.6/5-GAPDH/2.tif]

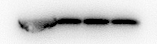

Supplement: Supplementary file 3 [file DataSheet4.zip › Fig.6/5-GAPDH/1.tif]

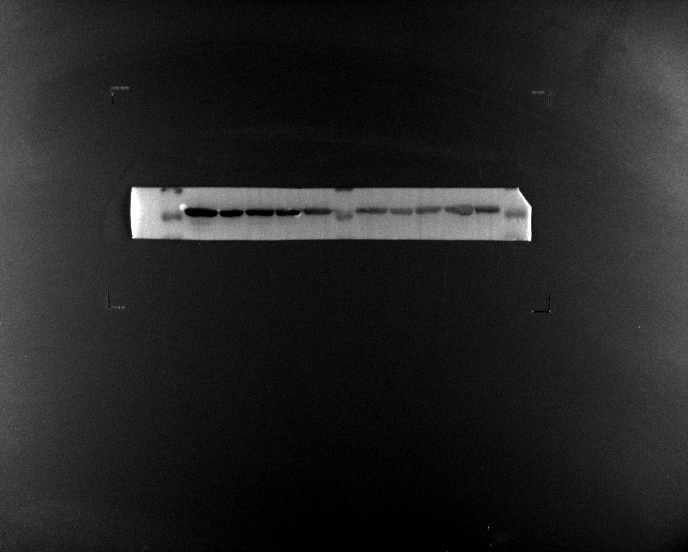

Supplement: Supplementary file 3 [file DataSheet4.zip › Fig.7/WTAP/1-2-WTAP YT.tif]

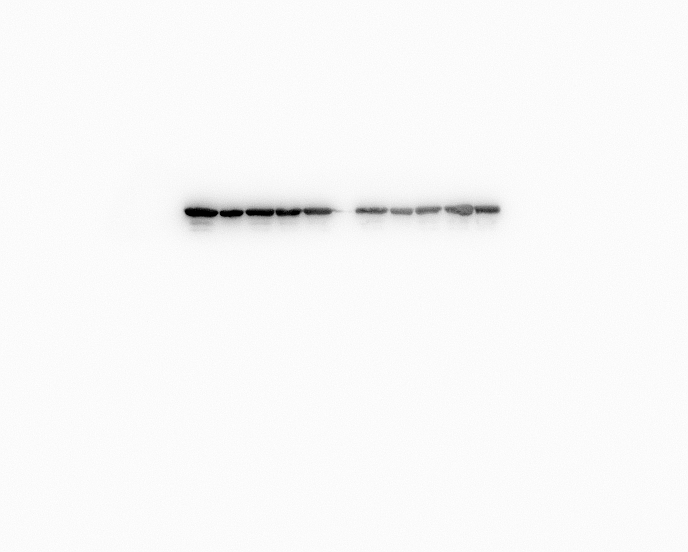

Supplement: Supplementary file 3 [file DataSheet4.zip › Fig.7/WTAP/1-2-WTAP.tif]

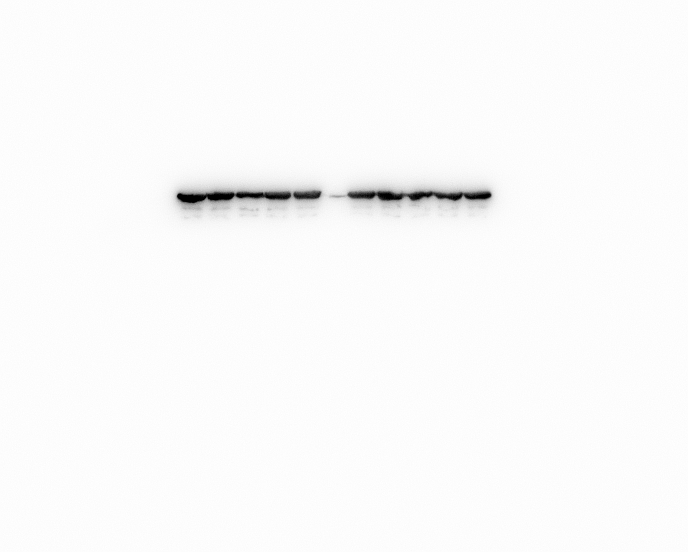

Supplement: Supplementary file 3 [file DataSheet4.zip › Fig.7/WTAP/3-4-WTAP.tif]

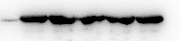

Supplement: Supplementary file 3 [file DataSheet4.zip › Fig.7/WTAP/3.tif]

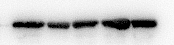

Supplement: Supplementary file 3 [file DataSheet4.zip › Fig.7/WTAP/2.tif]

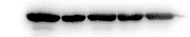

Supplement: Supplementary file 3 [file DataSheet4.zip › Fig.7/WTAP/1.tif]

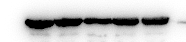

Supplement: Supplementary file 3 [file DataSheet4.zip › Fig.7/WTAP/4.tif]

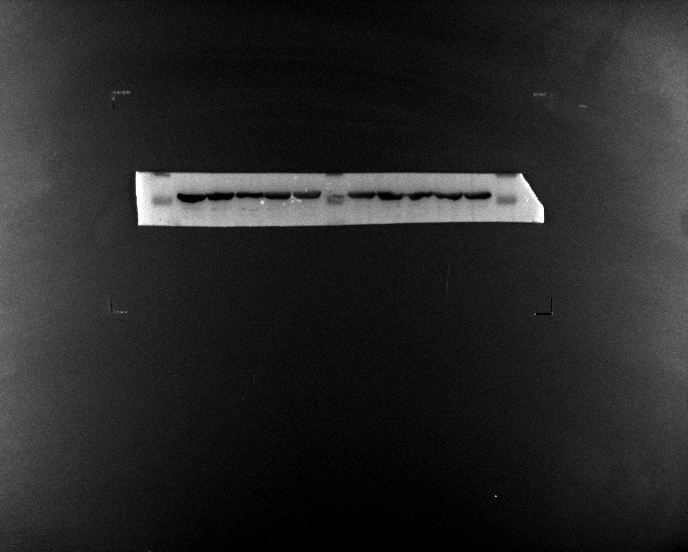

Supplement: Supplementary file 3 [file DataSheet4.zip › Fig.7/WTAP/3-4-WTAP YT.tif]

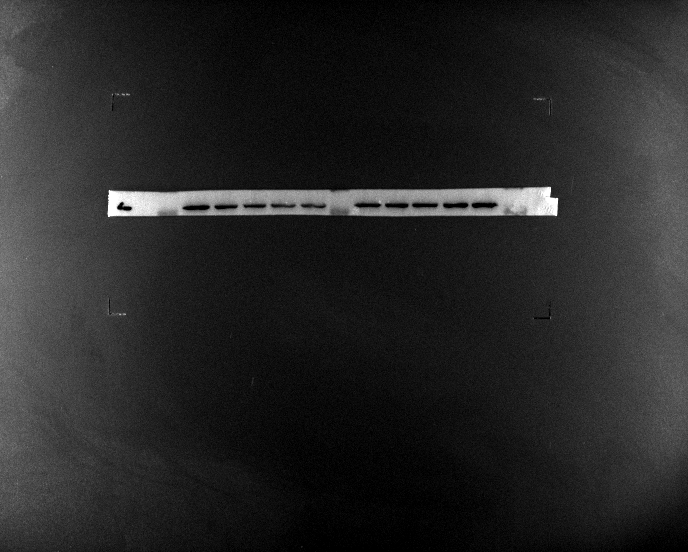

Supplement: Supplementary file 3 [file DataSheet4.zip › Fig.7/Fig.7-GAPDH/1-2-GAPDH YT.tif]

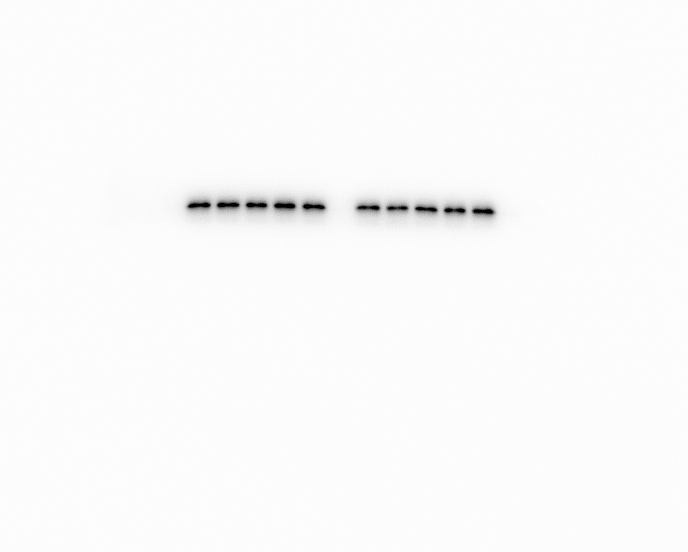

Supplement: Supplementary file 3 [file DataSheet4.zip › Fig.7/Fig.7-GAPDH/3-GAPDH.tif]

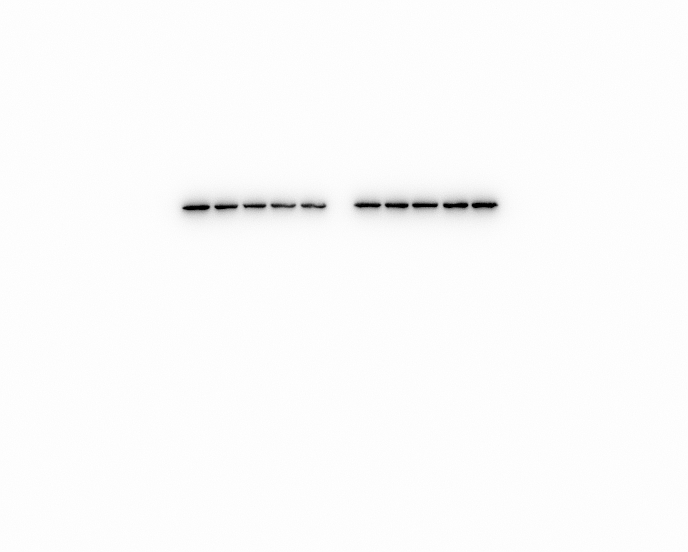

Supplement: Supplementary file 3 [file DataSheet4.zip › Fig.7/Fig.7-GAPDH/1-2-GAPDH.tif]

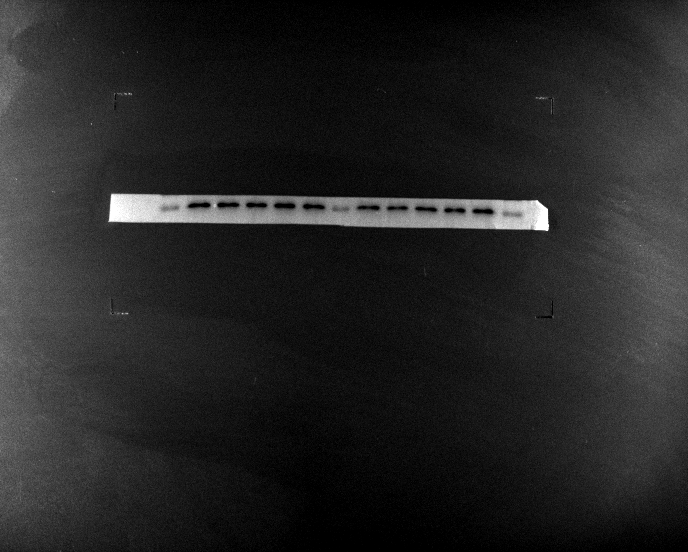

Supplement: Supplementary file 3 [file DataSheet4.zip › Fig.7/Fig.7-GAPDH/3-GAPDH YT.tif]

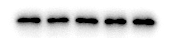

Supplement: Supplementary file 3 [file DataSheet4.zip › Fig.7/Fig.7-GAPDH/3.tif]

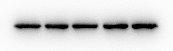

Supplement: Supplementary file 3 [file DataSheet4.zip › Fig.7/Fig.7-GAPDH/2.tif]

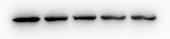

Supplement: Supplementary file 3 [file DataSheet4.zip › Fig.7/Fig.7-GAPDH/1.tif]

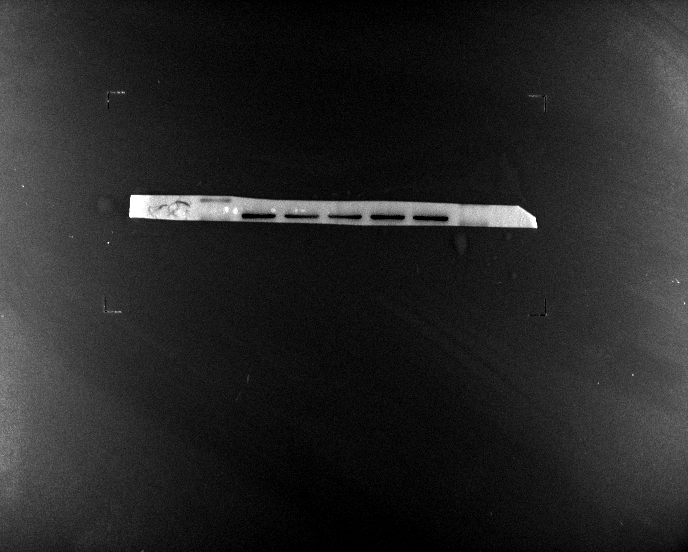

Supplement: Supplementary file 3 [file DataSheet4.zip › Fig.7/Fig.7-METTL3/1-METTL3 YT.tif]

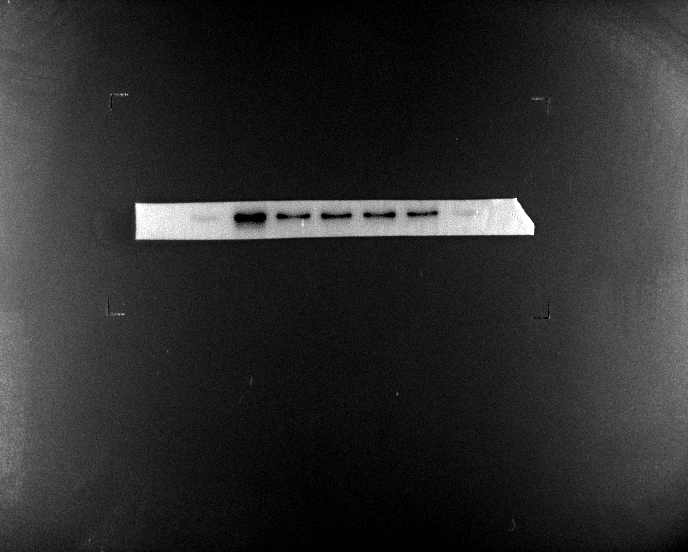

Supplement: Supplementary file 3 [file DataSheet4.zip › Fig.7/Fig.7-METTL3/2-METTL3 YT.tif]

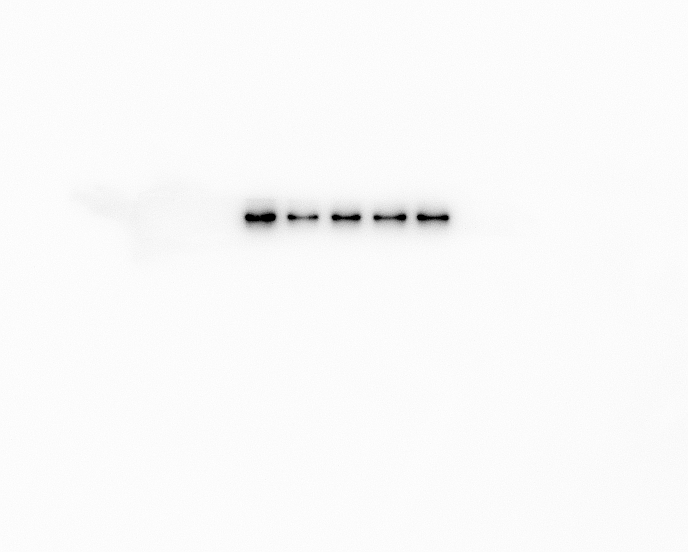

Supplement: Supplementary file 3 [file DataSheet4.zip › Fig.7/Fig.7-METTL3/3- METTL3.tif]

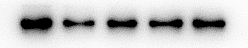

Supplement: Supplementary file 3 [file DataSheet4.zip › Fig.7/Fig.7-METTL3/3.tif]

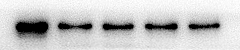

Supplement: Supplementary file 3 [file DataSheet4.zip › Fig.7/Fig.7-METTL3/2.tif]

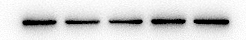

Supplement: Supplementary file 3 [file DataSheet4.zip › Fig.7/Fig.7-METTL3/1.tif]

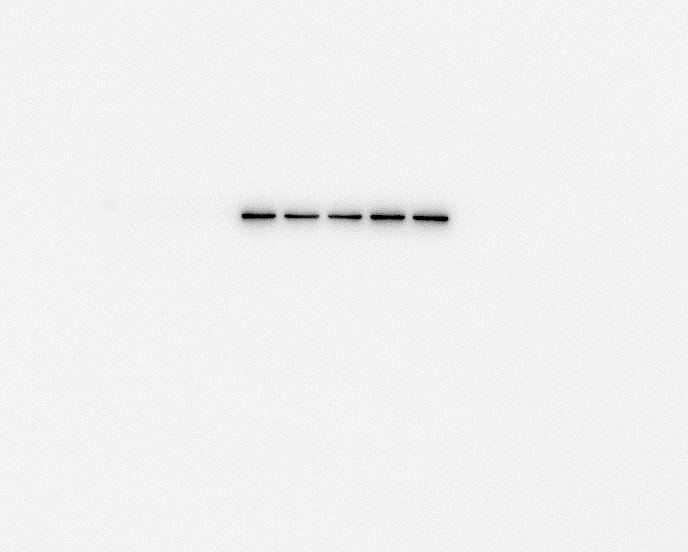

Supplement: Supplementary file 3 [file DataSheet4.zip › Fig.7/Fig.7-METTL3/1- METTL3.tif]

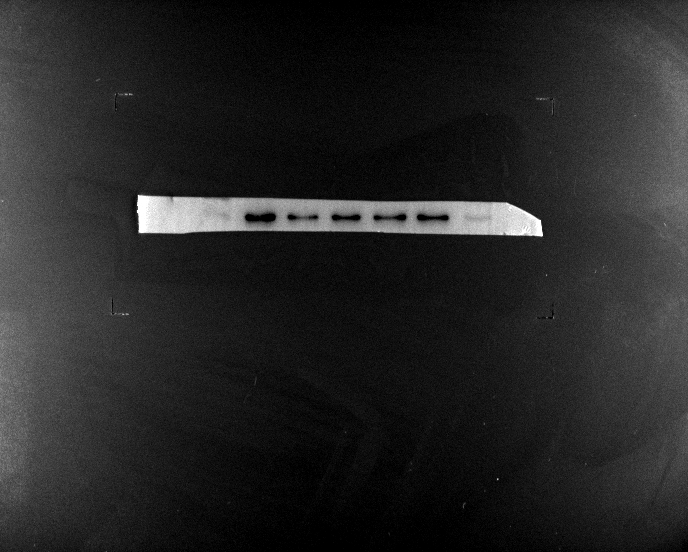

Supplement: Supplementary file 3 [file DataSheet4.zip › Fig.7/Fig.7-METTL3/3-METTL3 YT.tif]

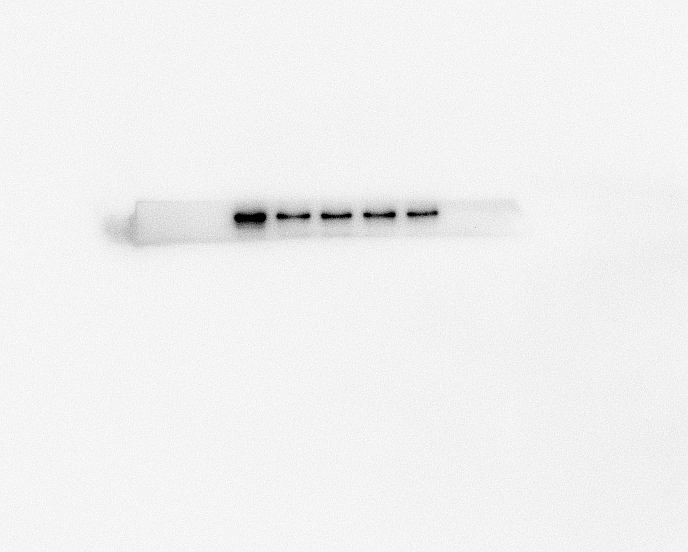

Supplement: Supplementary file 3 [file DataSheet4.zip › Fig.7/Fig.7-METTL3/2-METTL3.tif]

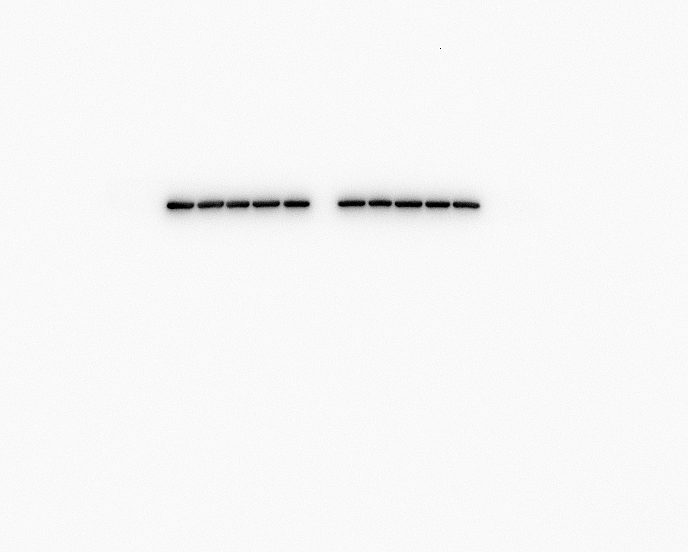

Supplement: Supplementary file 3 [file DataSheet4.zip › Fig.7/METTL14/2-3-METTL14.tif]

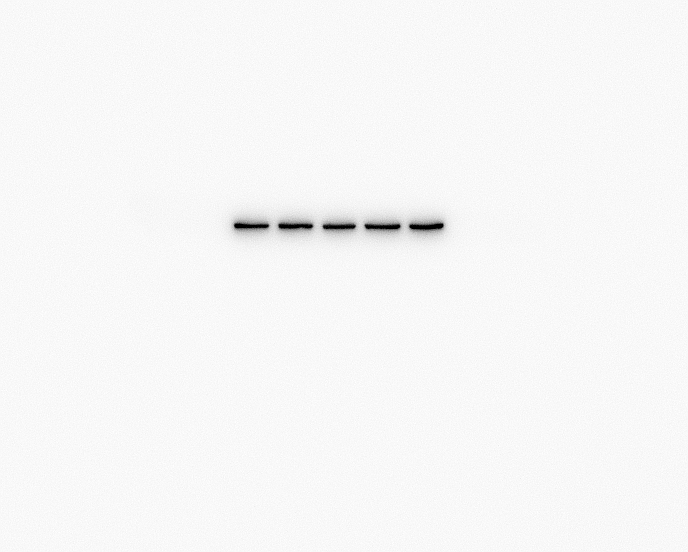

Supplement: Supplementary file 3 [file DataSheet4.zip › Fig.7/METTL14/1-METTL14.tif]

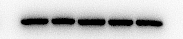

Supplement: Supplementary file 3 [file DataSheet4.zip › Fig.7/METTL14/3.tif]

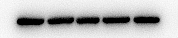

Supplement: Supplementary file 3 [file DataSheet4.zip › Fig.7/METTL14/2.tif]

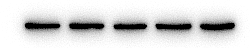

Supplement: Supplementary file 3 [file DataSheet4.zip › Fig.7/METTL14/1.tif]

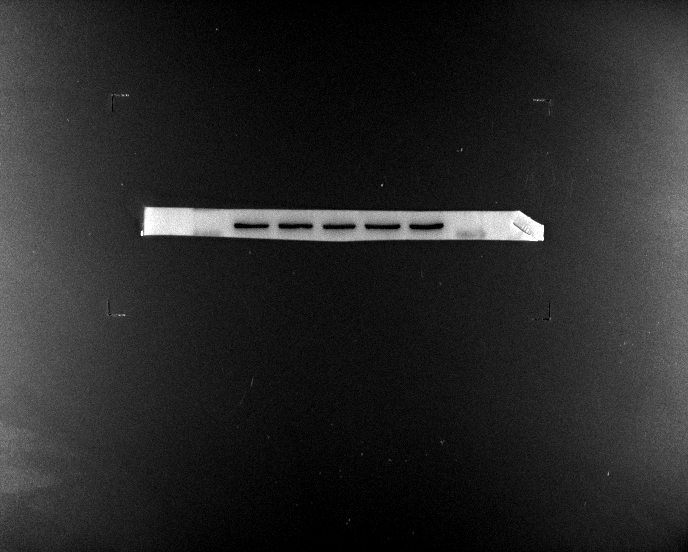

Supplement: Supplementary file 3 [file DataSheet4.zip › Fig.7/METTL14/1-METTL14 YT.tif]

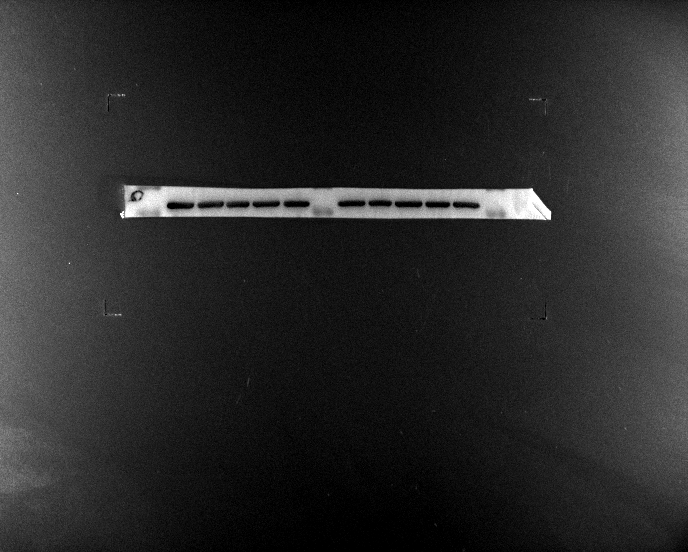

Supplement: Supplementary file 3 [file DataSheet4.zip › Fig.7/METTL14/2-3-METTL14 YT.tif]

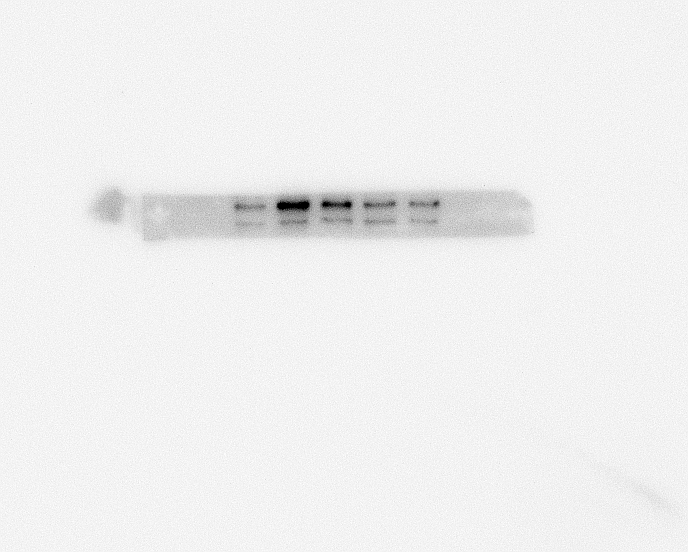

Supplement: Supplementary file 3 [file DataSheet4.zip › Fig.7/Fig.7-PTEN/PTEN/3-PTEN.tif]

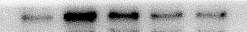

Supplement: Supplementary file 3 [file DataSheet4.zip › Fig.7/Fig.7-PTEN/PTEN/3.tif]

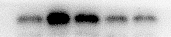

Supplement: Supplementary file 3 [file DataSheet4.zip › Fig.7/Fig.7-PTEN/PTEN/2.tif]

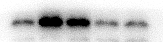

Supplement: Supplementary file 3 [file DataSheet4.zip › Fig.7/Fig.7-PTEN/PTEN/1.tif]

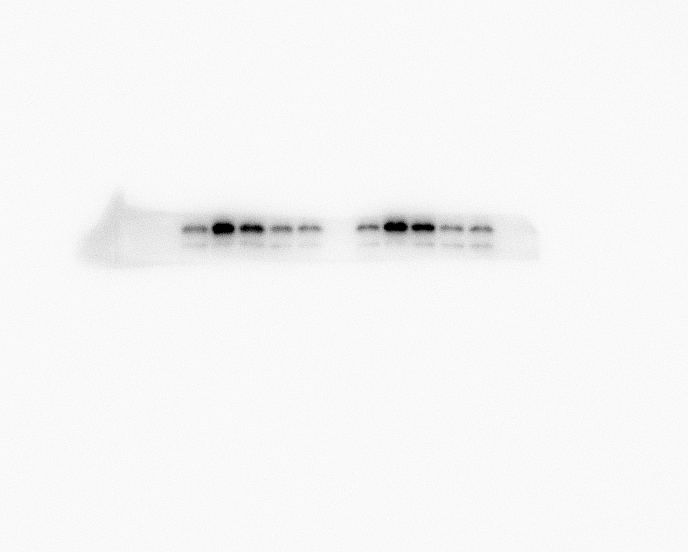

Supplement: Supplementary file 3 [file DataSheet4.zip › Fig.7/Fig.7-PTEN/PTEN/1-2-PTEN.tif]

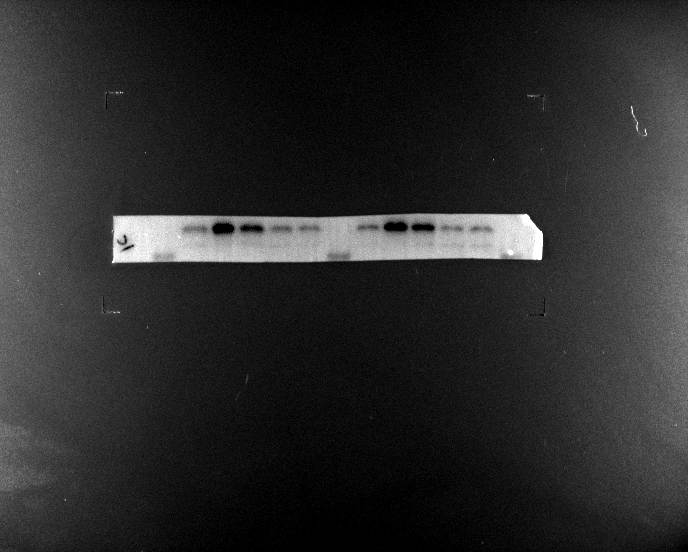

Supplement: Supplementary file 3 [file DataSheet4.zip › Fig.7/Fig.7-PTEN/PTEN/1-2-PTEN YT.tif]

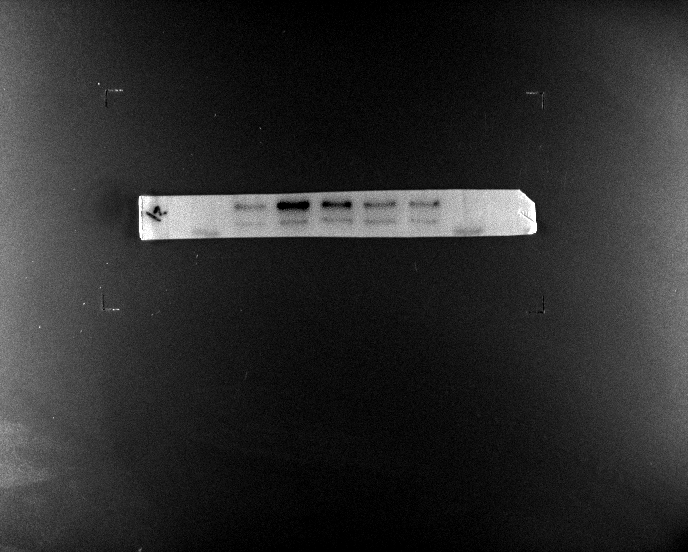

Supplement: Supplementary file 3 [file DataSheet4.zip › Fig.7/Fig.7-PTEN/PTEN/3-PTEN YT.tif]

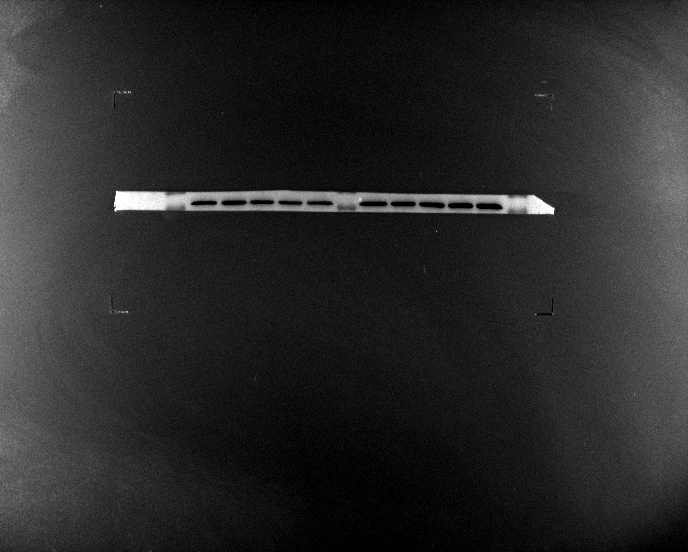

Supplement: Supplementary file 3 [file DataSheet4.zip › Fig.7/Fig.7-PTEN/GAPDH/2-3-GAPDH YT.tif]

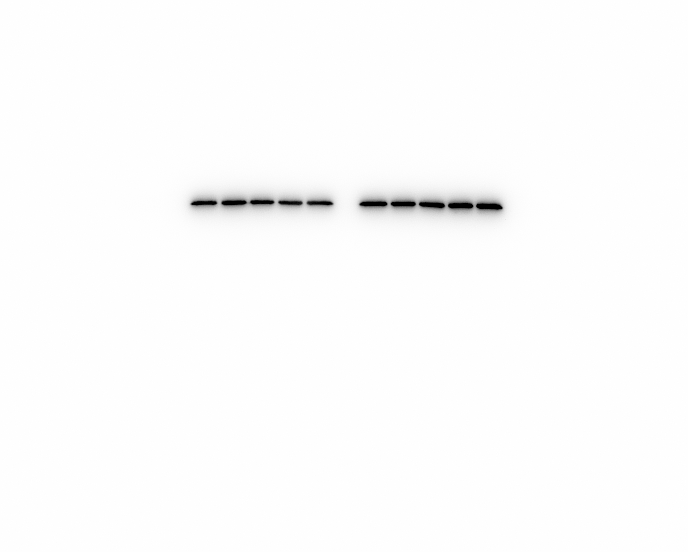

Supplement: Supplementary file 3 [file DataSheet4.zip › Fig.7/Fig.7-PTEN/GAPDH/2-3-GAPDH.tif]

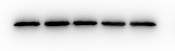

Supplement: Supplementary file 3 [file DataSheet4.zip › Fig.7/Fig.7-PTEN/GAPDH/3.tif]

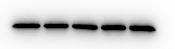

Supplement: Supplementary file 3 [file DataSheet4.zip › Fig.7/Fig.7-PTEN/GAPDH/2.tif]

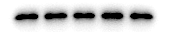

Supplement: Supplementary file 3 [file DataSheet4.zip › Fig.7/Fig.7-PTEN/GAPDH/1.tif]

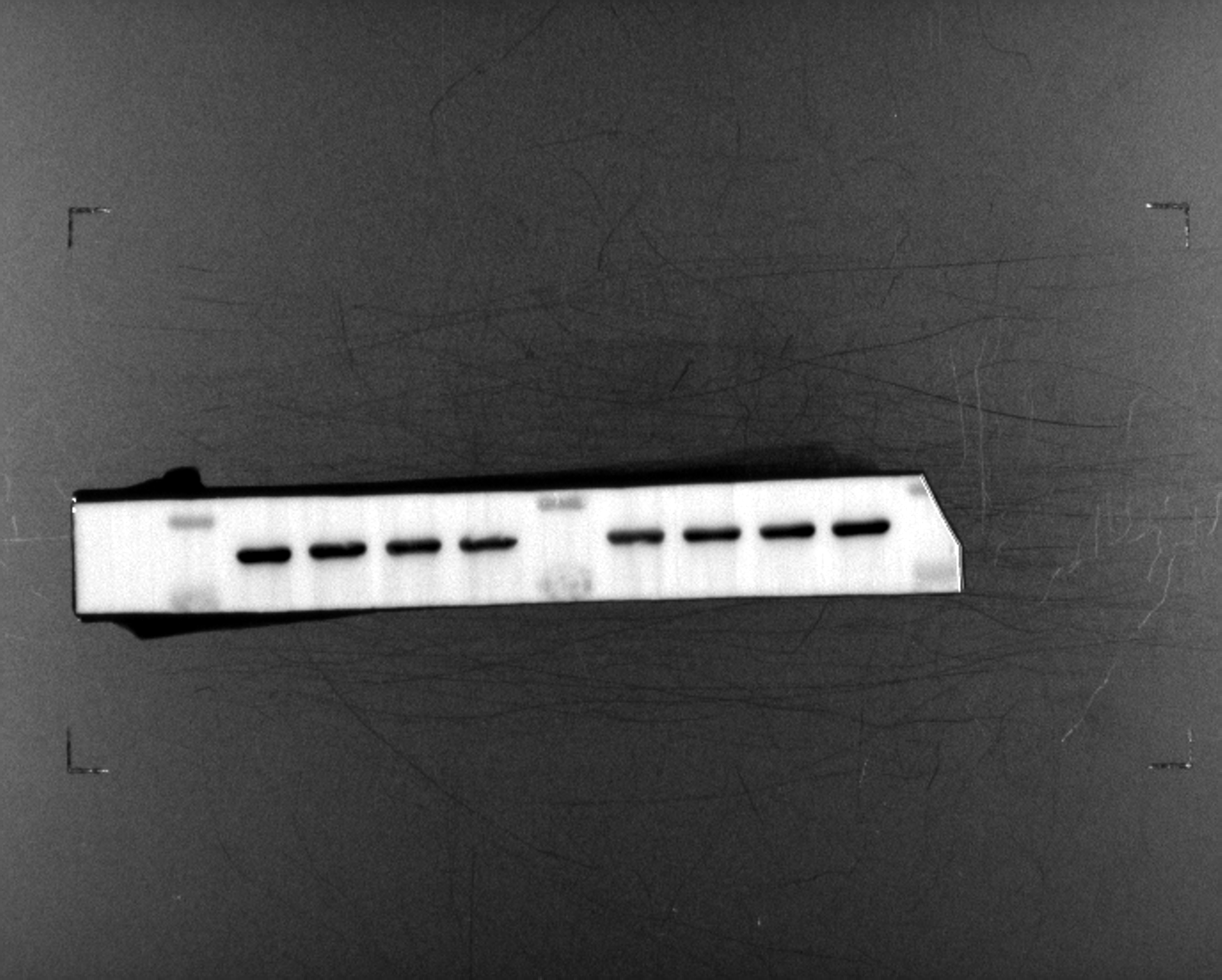

Supplement: Supplementary file 4 [file DataSheet6.zip › Fig.9/Fig.9 A/GAPDH/1-10s yt.Tif]

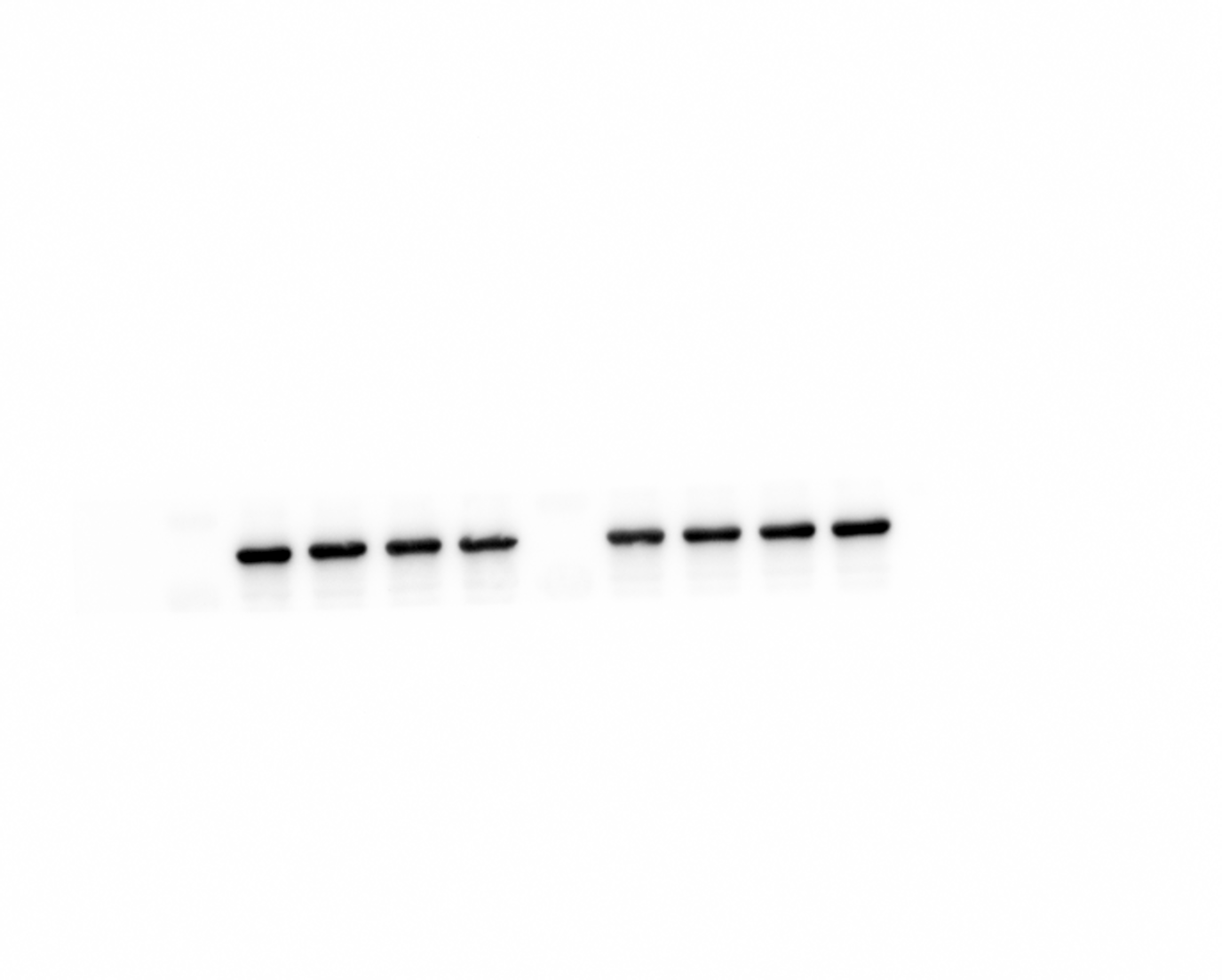

Supplement: Supplementary file 4 [file DataSheet6.zip › Fig.9/Fig.9 A/GAPDH/1-10s.Tif]

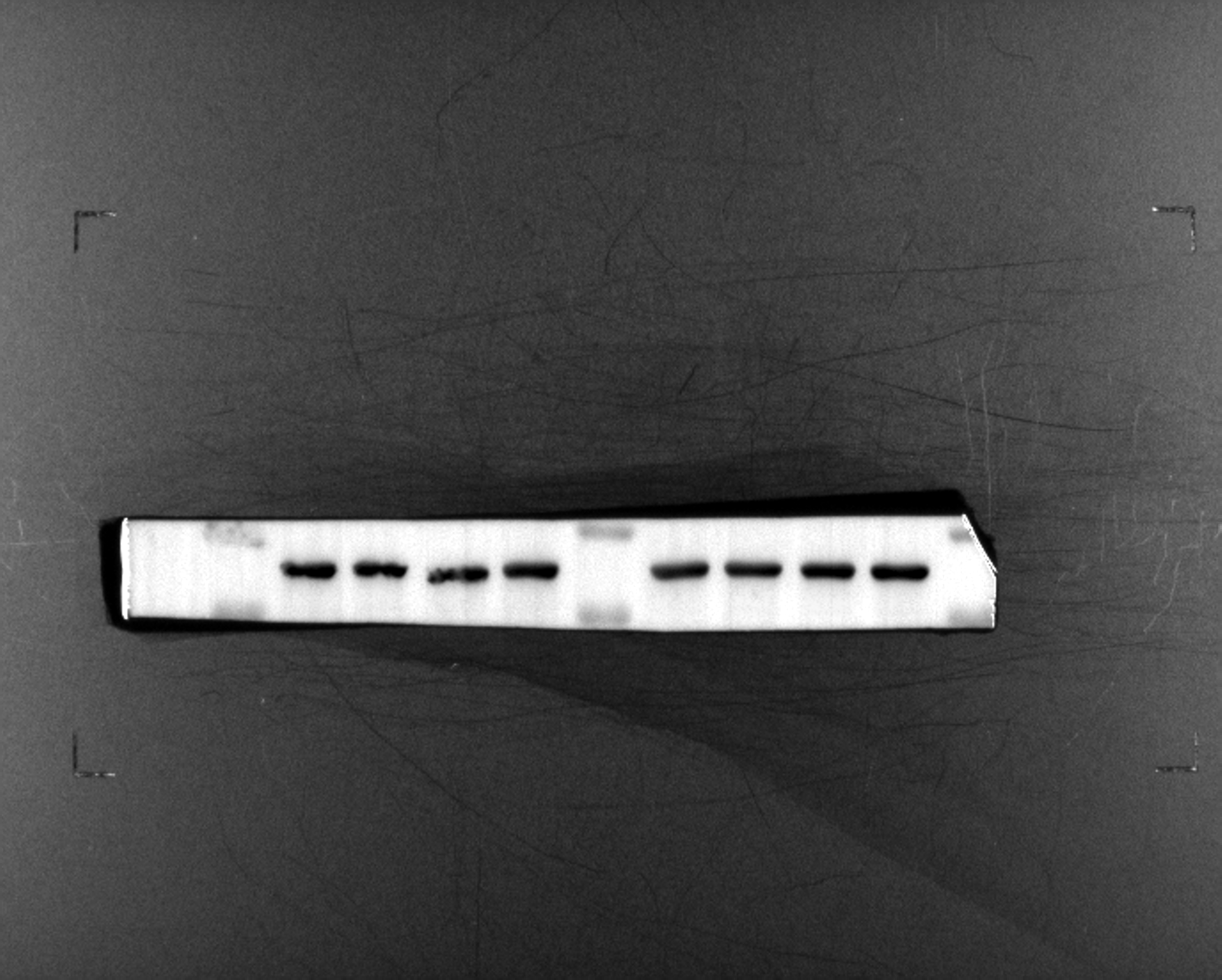

Supplement: Supplementary file 4 [file DataSheet6.zip › Fig.9/Fig.9 A/GAPDH/2-10s YT.Tif]

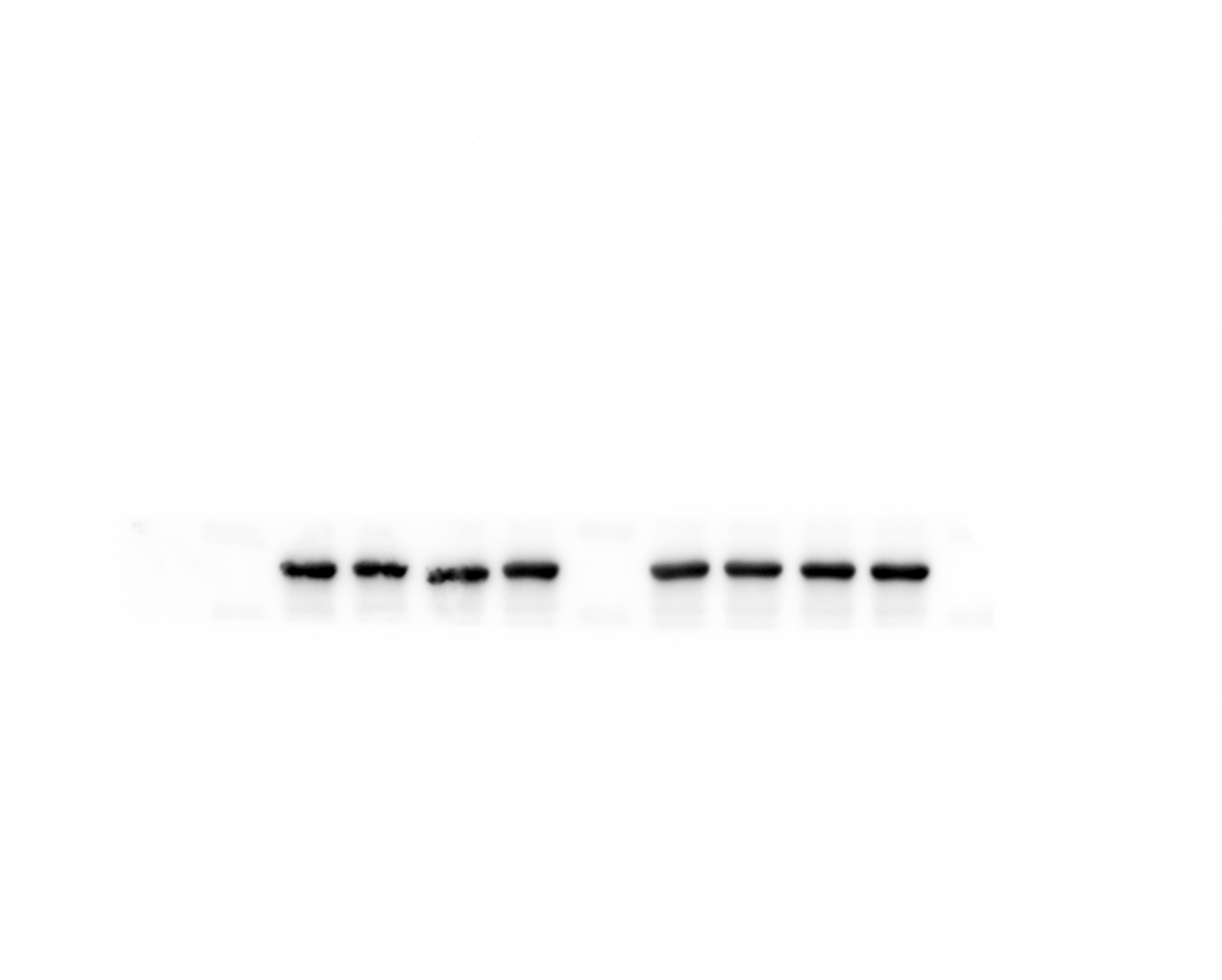

Supplement: Supplementary file 4 [file DataSheet6.zip › Fig.9/Fig.9 A/GAPDH/2-10s.Tif]

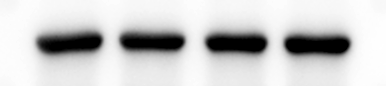

Supplement: Supplementary file 4 [file DataSheet6.zip › Fig.9/Fig.9 A/GAPDH/PS-右-2-10s.tif]

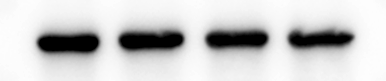

Supplement: Supplementary file 4 [file DataSheet6.zip › Fig.9/Fig.9 A/GAPDH/PS-左-1-10s.tif]
