# Supplementary figures and images for: Total Flavones of Abelmoschus manihot Ameliorates Podocyte Pyroptosis and Injury in High Glucose Conditions by Targeting METTL3-Dependent m6A Modification-Mediated NLRP3-Inflammasome Activation and PTEN/PI3K/Akt Signaling (part 4 of 6)
Source: Front Pharmacol. 2021 Jul 15;12:667644. doi: 10.3389/fphar.2021.667644 (PMC8319635; doi:10.3389/fphar.2021.667644)

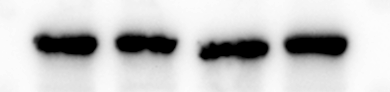

Supplement: Supplementary file 4 [file DataSheet6.zip › Fig.9/Fig.9 A/GAPDH/PS-左-2-10s.tif]

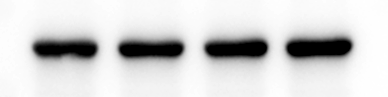

Supplement: Supplementary file 4 [file DataSheet6.zip › Fig.9/Fig.9 A/GAPDH/用 PS-右-1-10s.tif]

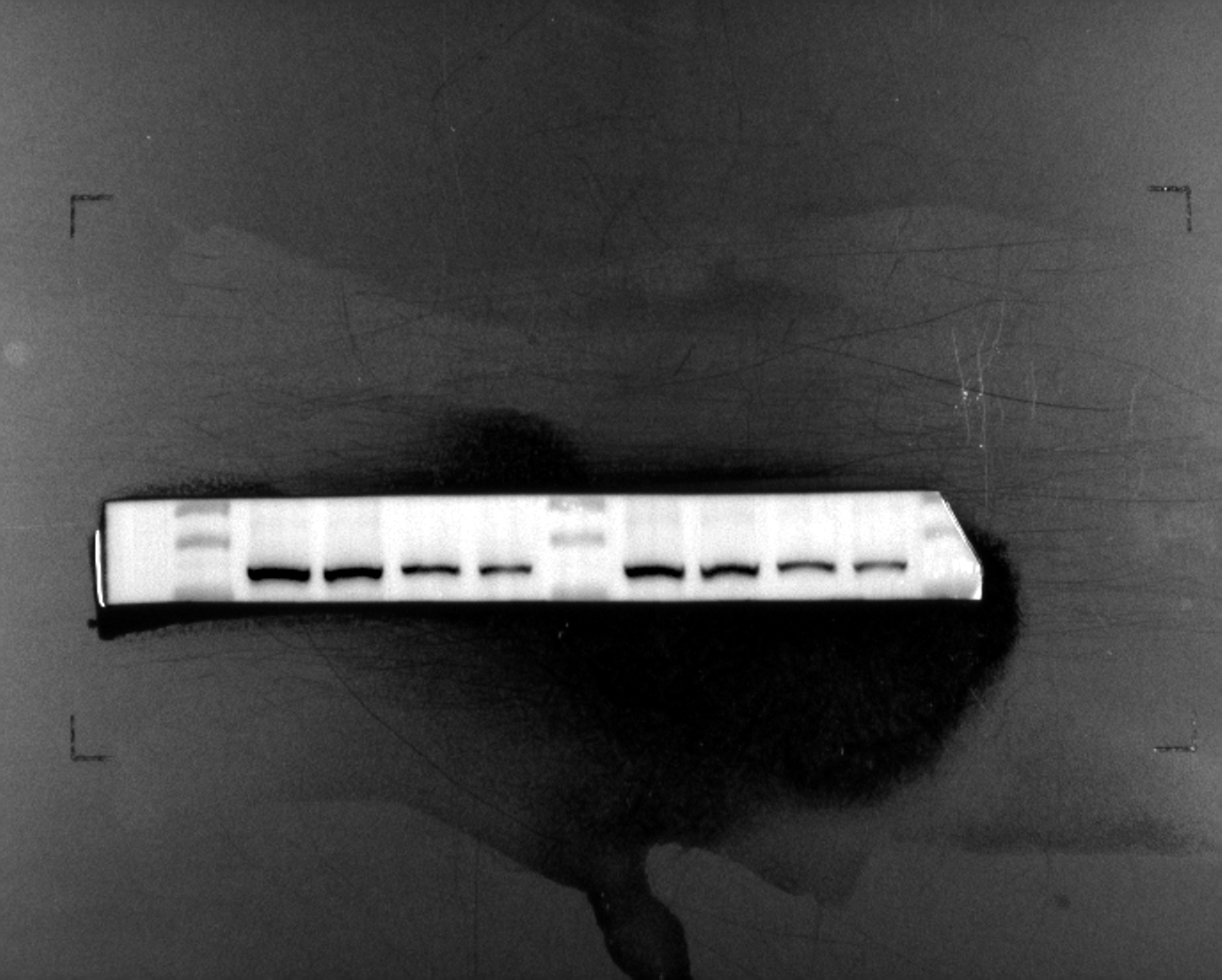

Supplement: Supplementary file 4 [file DataSheet6.zip › Fig.9/Fig.9 A/METTL3 siRNA/1-METTL3-30s YT.Tif]

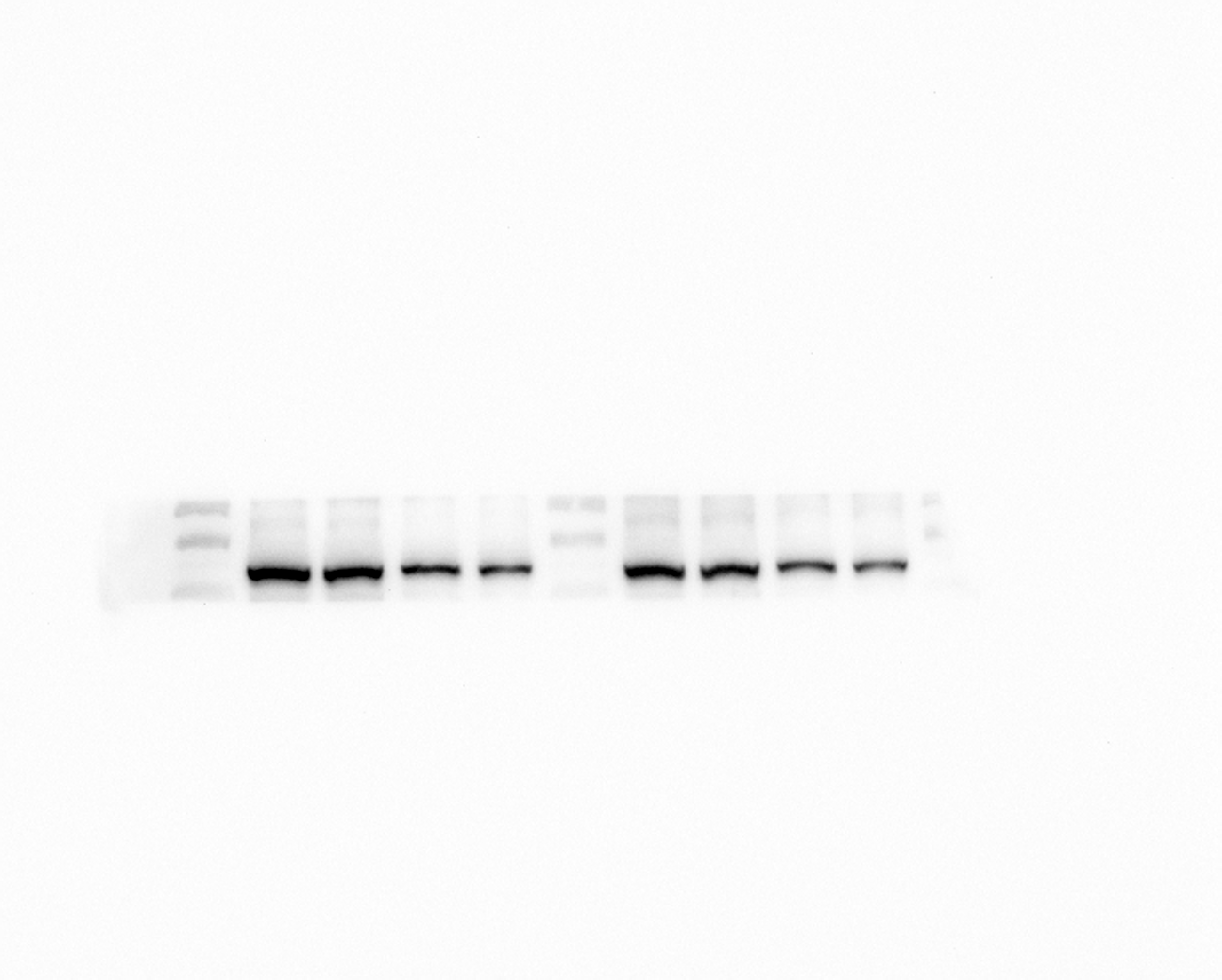

Supplement: Supplementary file 4 [file DataSheet6.zip › Fig.9/Fig.9 A/METTL3 siRNA/1-METTL3-30s.Tif]

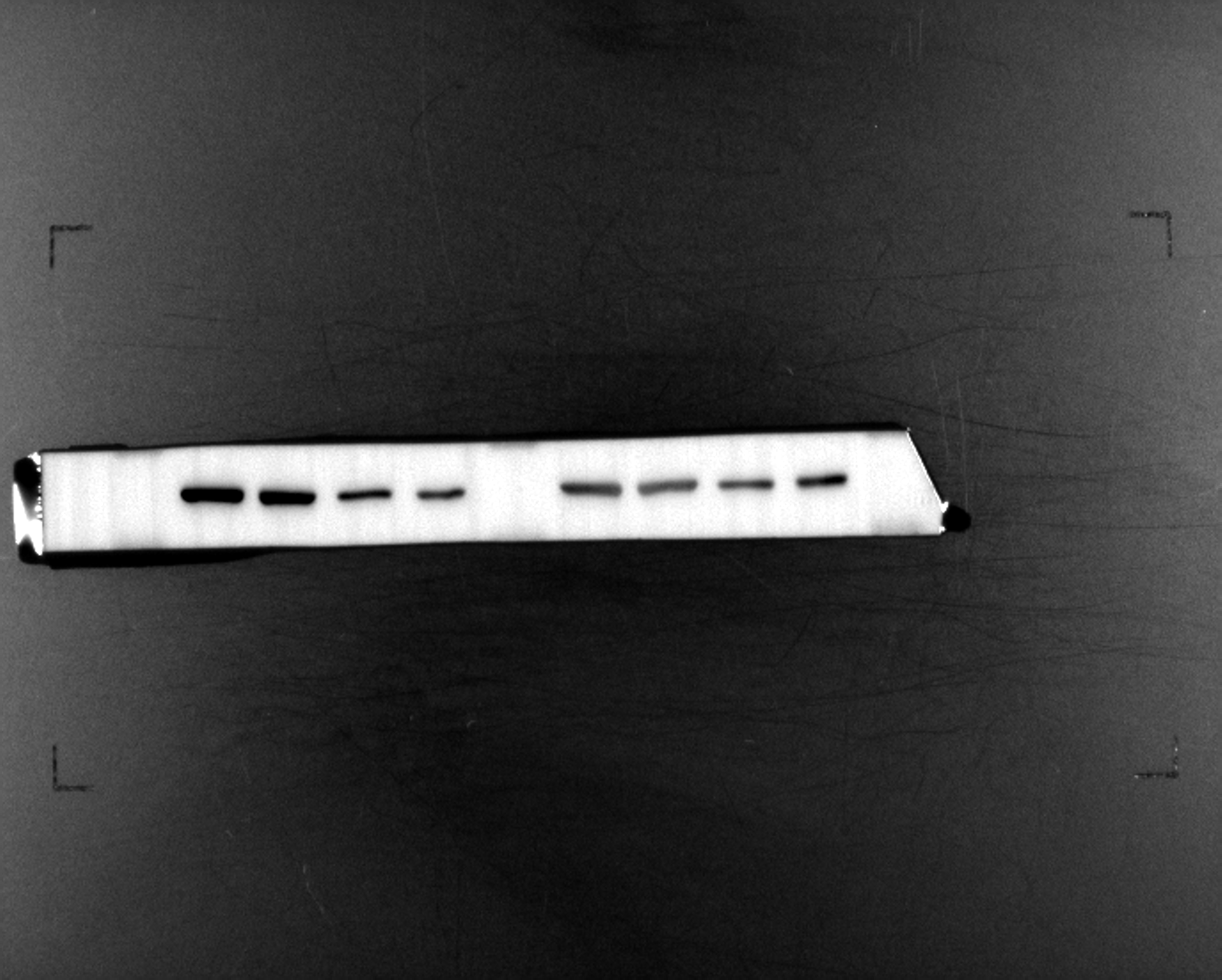

Supplement: Supplementary file 4 [file DataSheet6.zip › Fig.9/Fig.9 A/METTL3 siRNA/2-METTL3-30s YT.Tif]

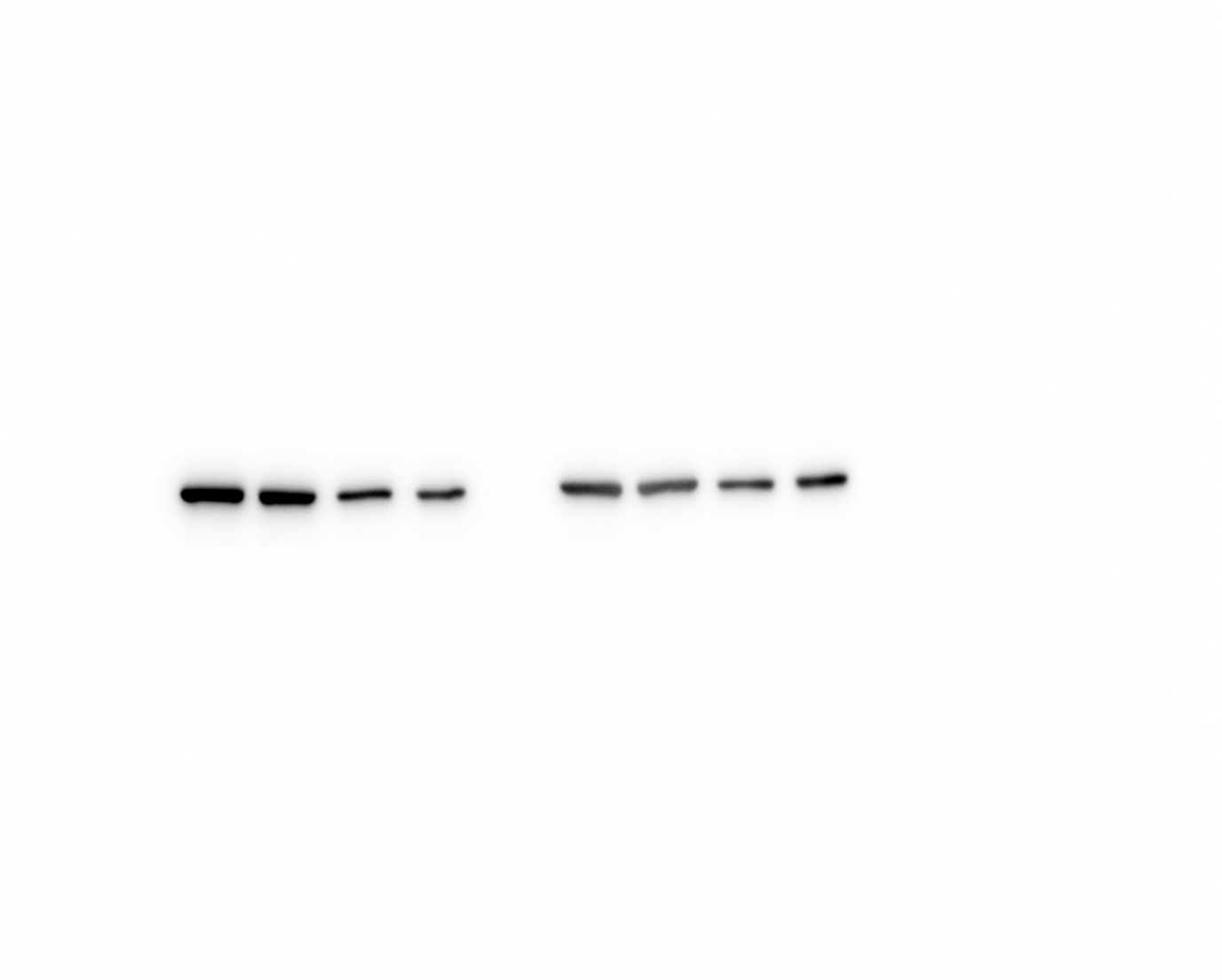

Supplement: Supplementary file 4 [file DataSheet6.zip › Fig.9/Fig.9 A/METTL3 siRNA/2-METTL3-30s.Tif]

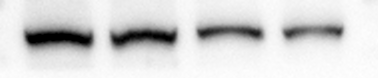

Supplement: Supplementary file 4 [file DataSheet6.zip › Fig.9/Fig.9 A/METTL3 siRNA/PS-右-1-METTL3-30s.tif]

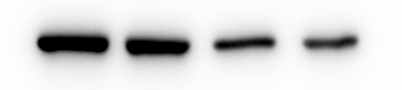

Supplement: Supplementary file 4 [file DataSheet6.zip › Fig.9/Fig.9 A/METTL3 siRNA/PS-左-2-METTL3-30s.tif]

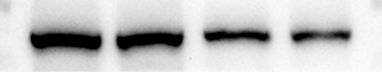

Supplement: Supplementary file 4 [file DataSheet6.zip › Fig.9/Fig.9 A/METTL3 siRNA/用 PS-左-1-METTL3-30s.tif]

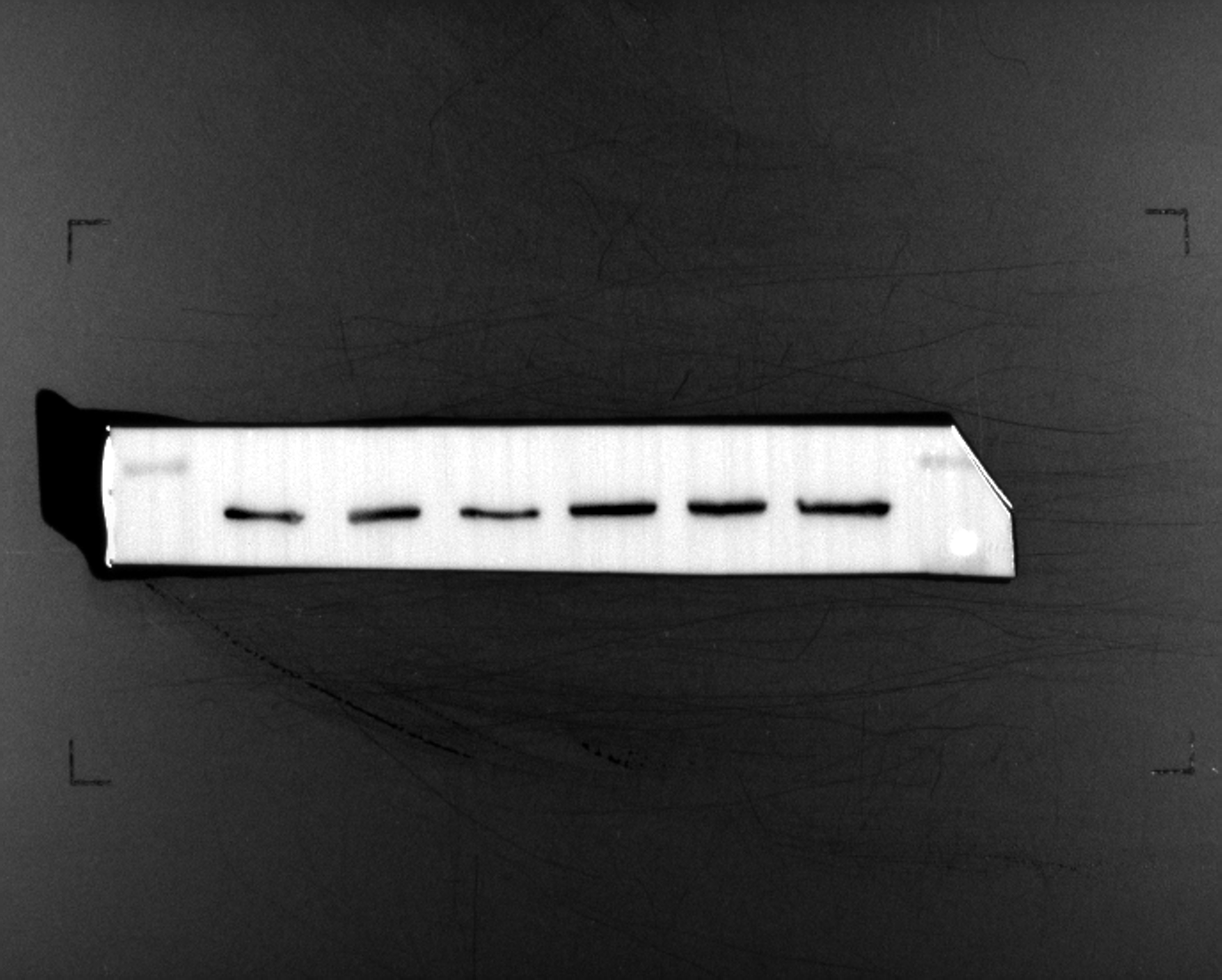

Supplement: Supplementary file 4 [file DataSheet6.zip › Fig.9/Fig.9 B/1-PTEN/1-PTEN-60s YT.Tif]

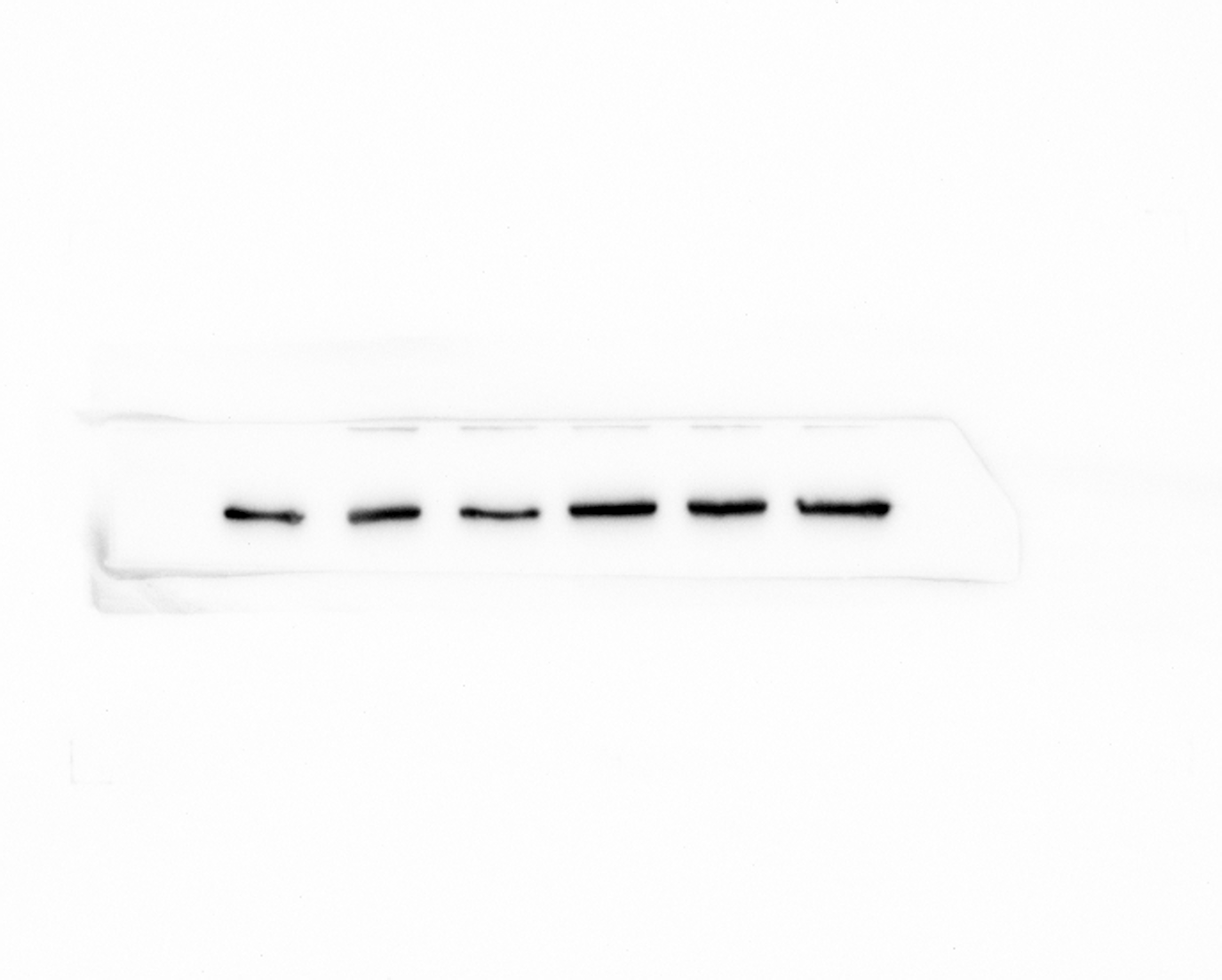

Supplement: Supplementary file 4 [file DataSheet6.zip › Fig.9/Fig.9 B/1-PTEN/1-PTEN-60s.Tif]

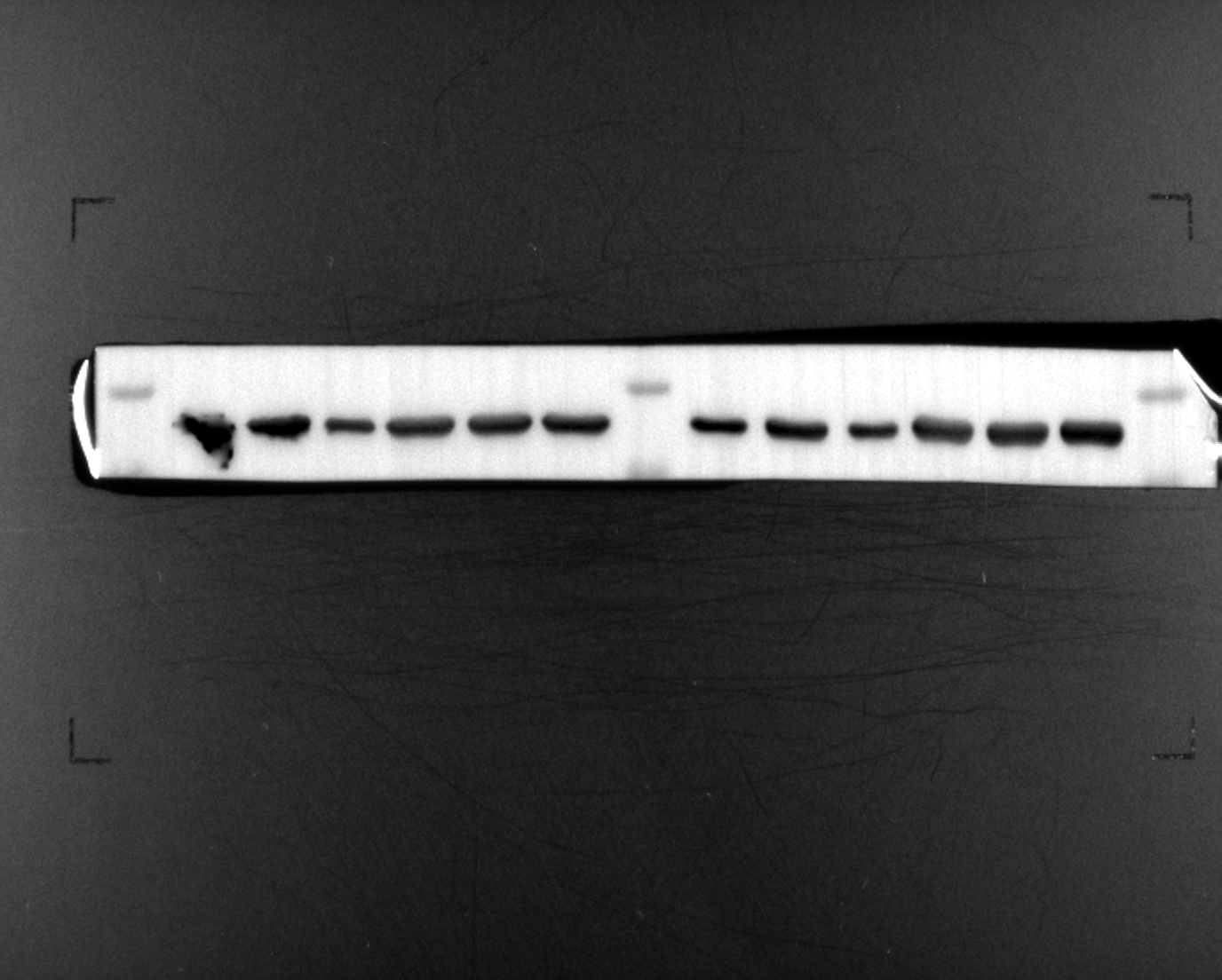

Supplement: Supplementary file 4 [file DataSheet6.zip › Fig.9/Fig.9 B/1-PTEN/2-PTEN-60s YT.Tif]

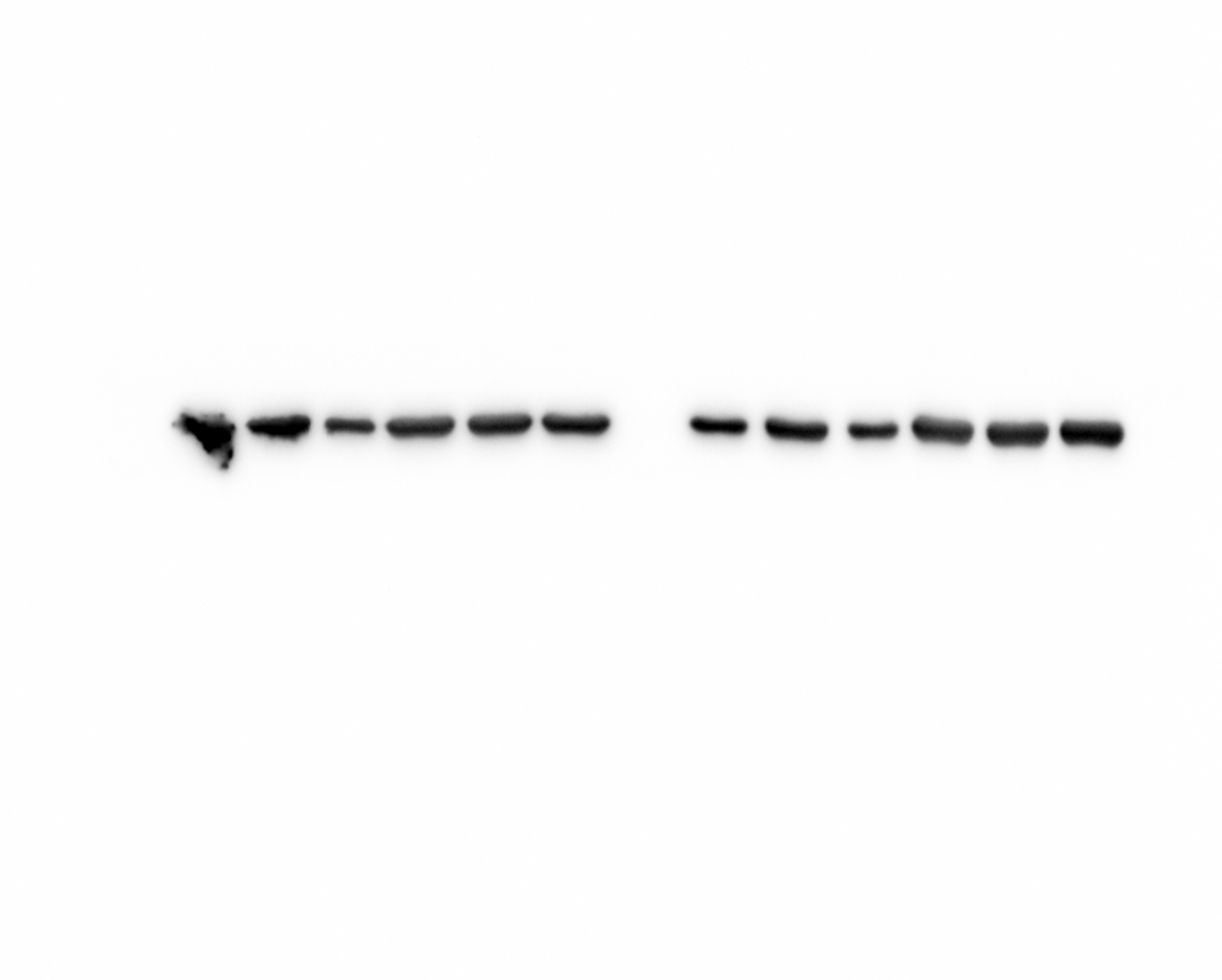

Supplement: Supplementary file 4 [file DataSheet6.zip › Fig.9/Fig.9 B/1-PTEN/2-PTEN-60s.Tif]

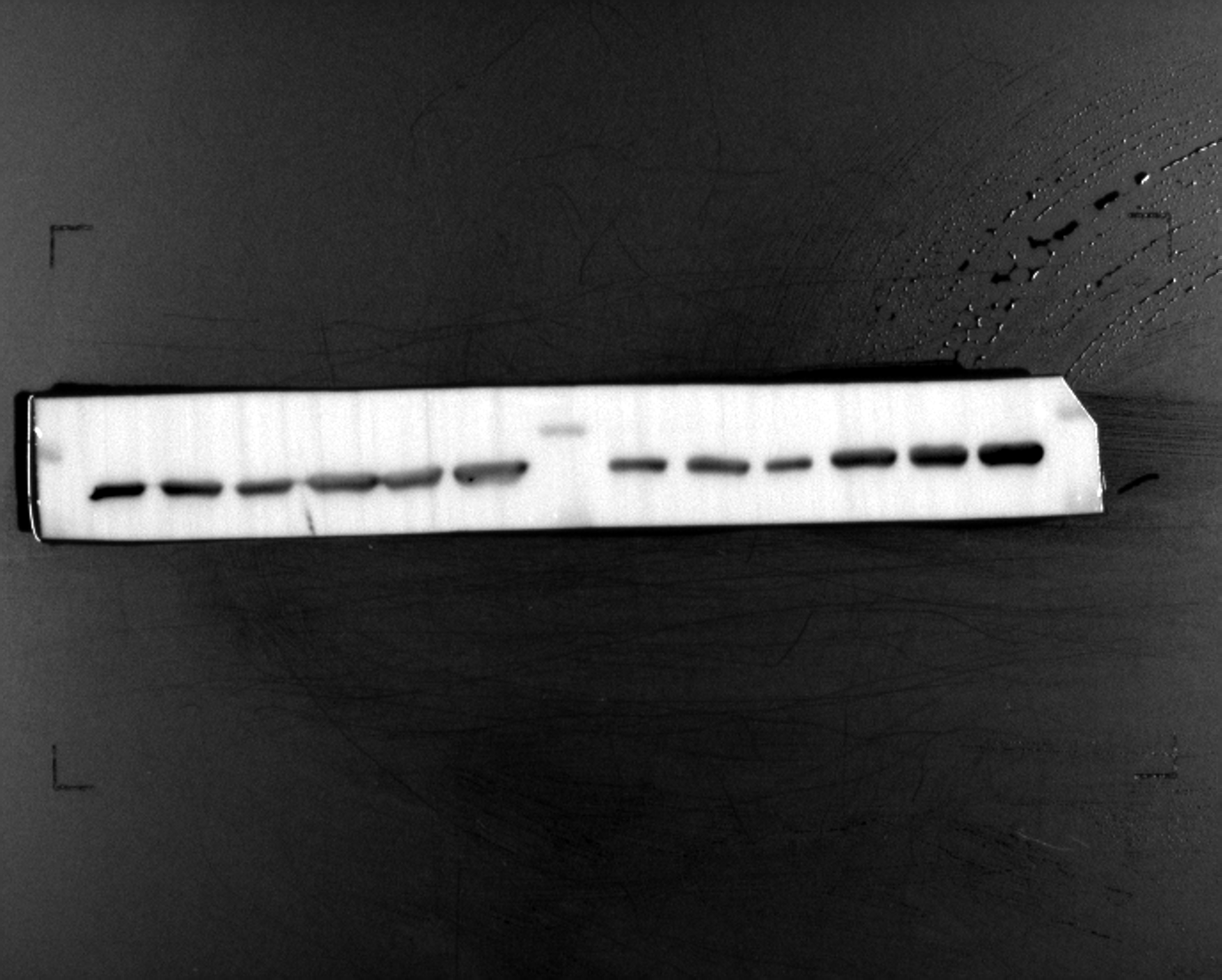

Supplement: Supplementary file 4 [file DataSheet6.zip › Fig.9/Fig.9 B/1-PTEN/3-PTEN-60s YT.Tif]

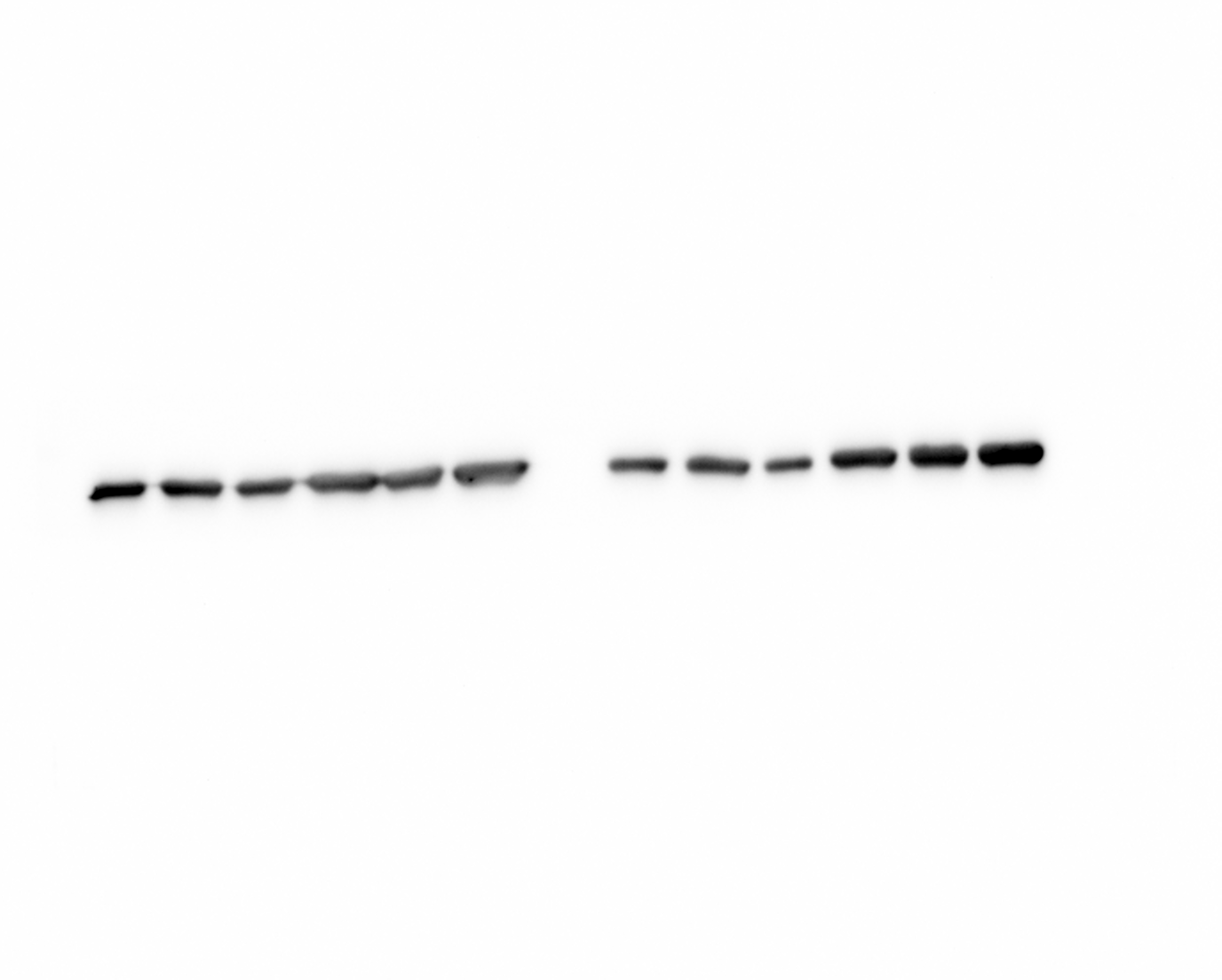

Supplement: Supplementary file 4 [file DataSheet6.zip › Fig.9/Fig.9 B/1-PTEN/3-PTEN-60s.Tif]

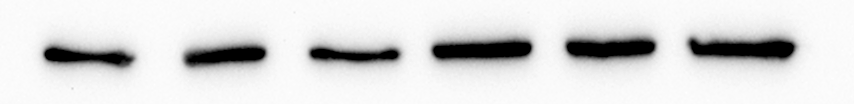

Supplement: Supplementary file 4 [file DataSheet6.zip › Fig.9/Fig.9 B/1-PTEN/PS 1-PTEN-60s.tif]

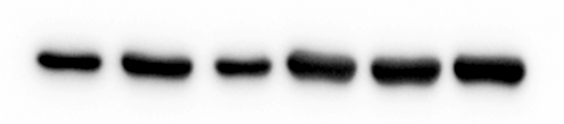

Supplement: Supplementary file 4 [file DataSheet6.zip › Fig.9/Fig.9 B/1-PTEN/PS 右-2-PTEN-60s.tif]

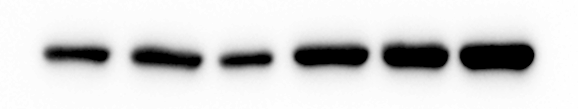

Supplement: Supplementary file 4 [file DataSheet6.zip › Fig.9/Fig.9 B/1-PTEN/PS 右3-PTEN-60s.tif]

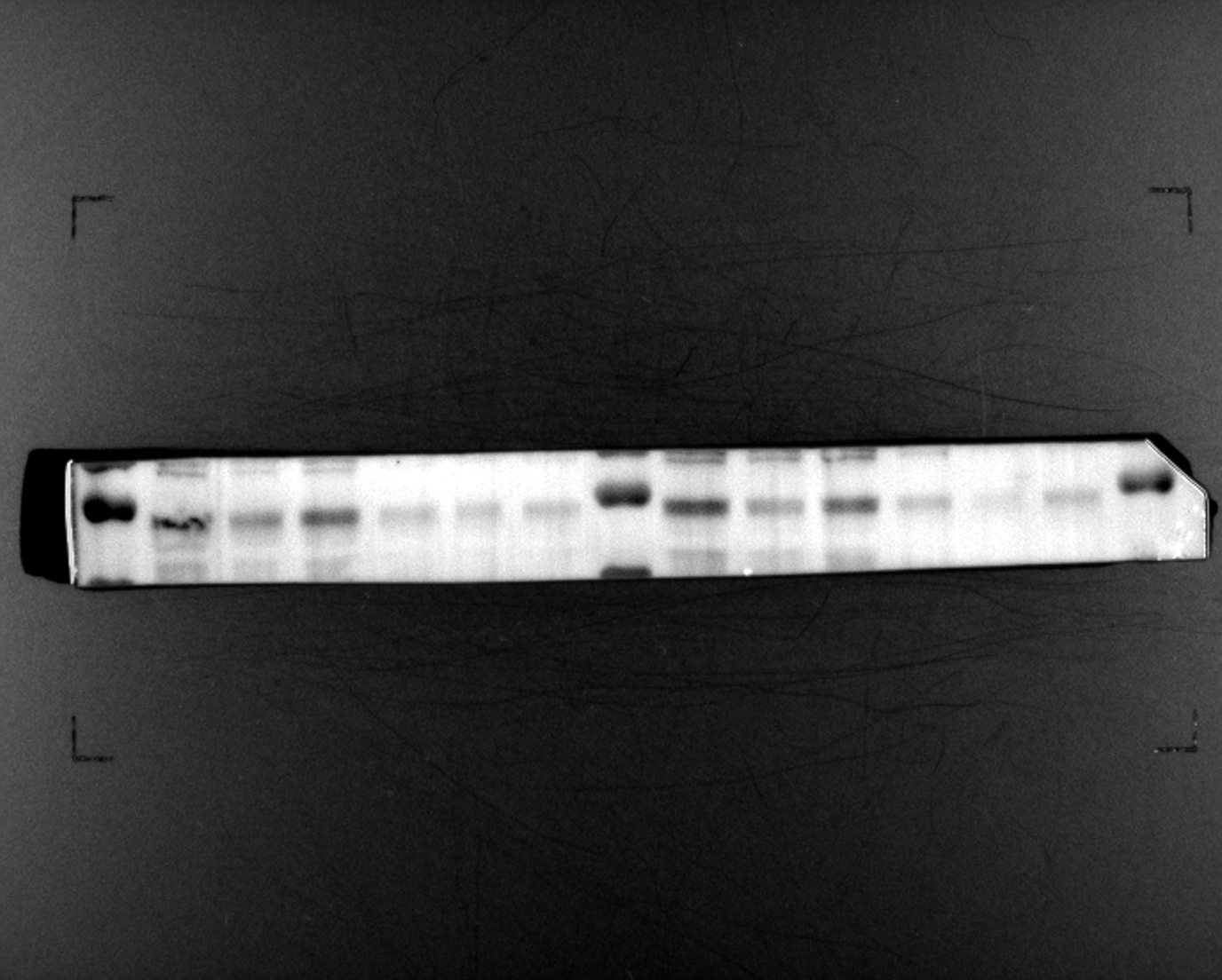

Supplement: Supplementary file 4 [file DataSheet6.zip › Fig.9/Fig.9 B/2-p-PI3K/1-p-PI3K-120S YT.Tif]

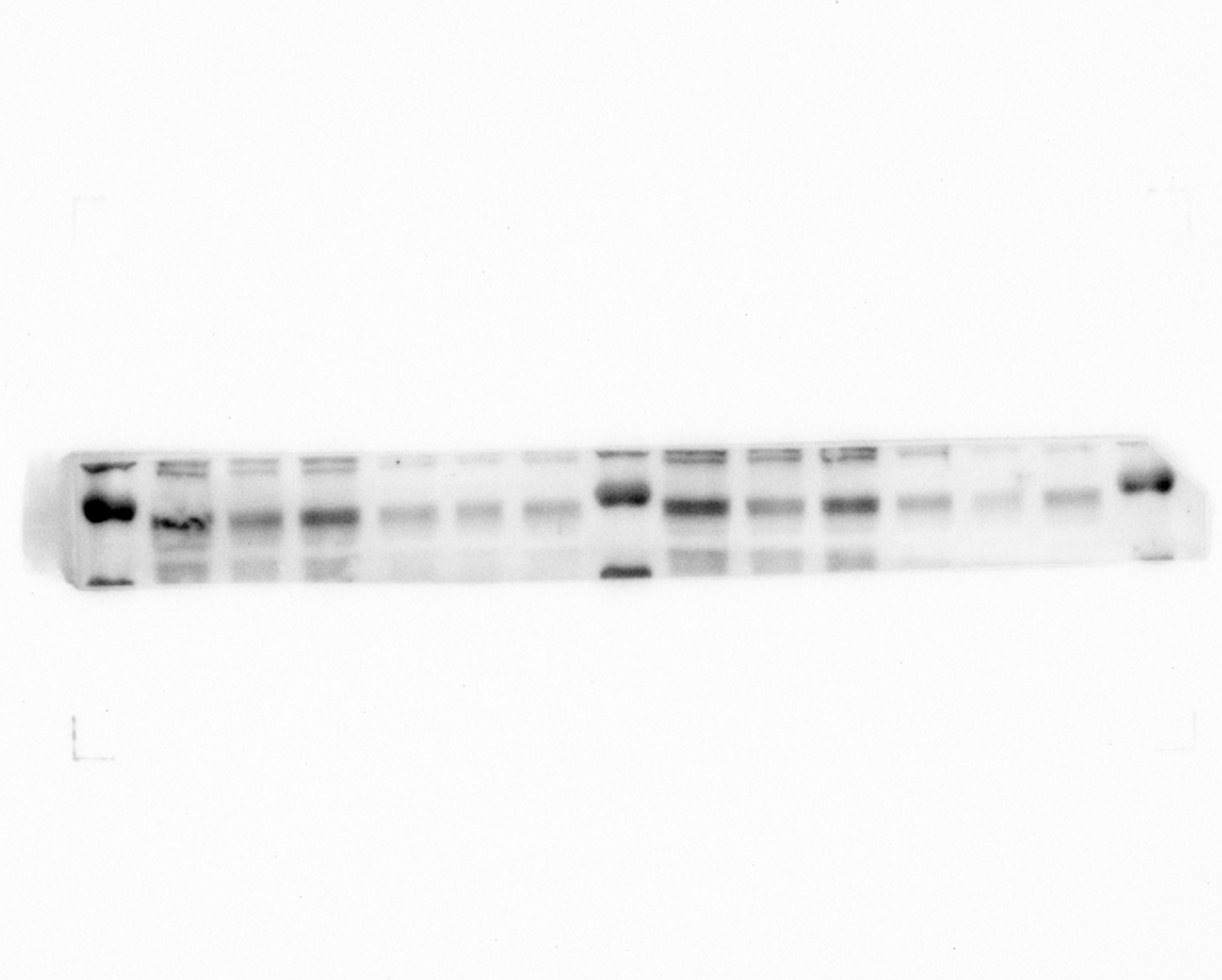

Supplement: Supplementary file 4 [file DataSheet6.zip › Fig.9/Fig.9 B/2-p-PI3K/1-p-PI3K-120S.Tif]

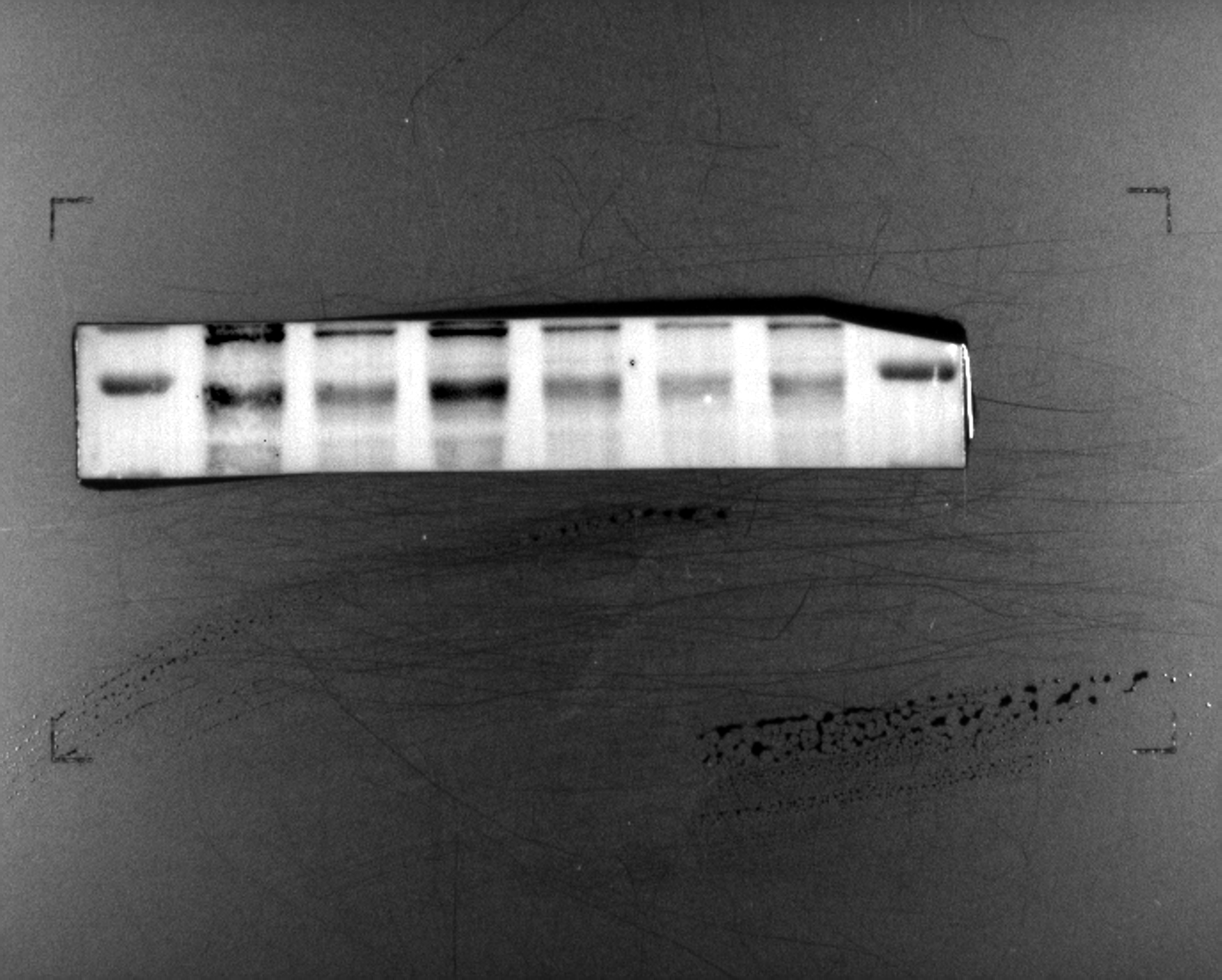

Supplement: Supplementary file 4 [file DataSheet6.zip › Fig.9/Fig.9 B/2-p-PI3K/2-p-PI3K-120S YT.Tif]

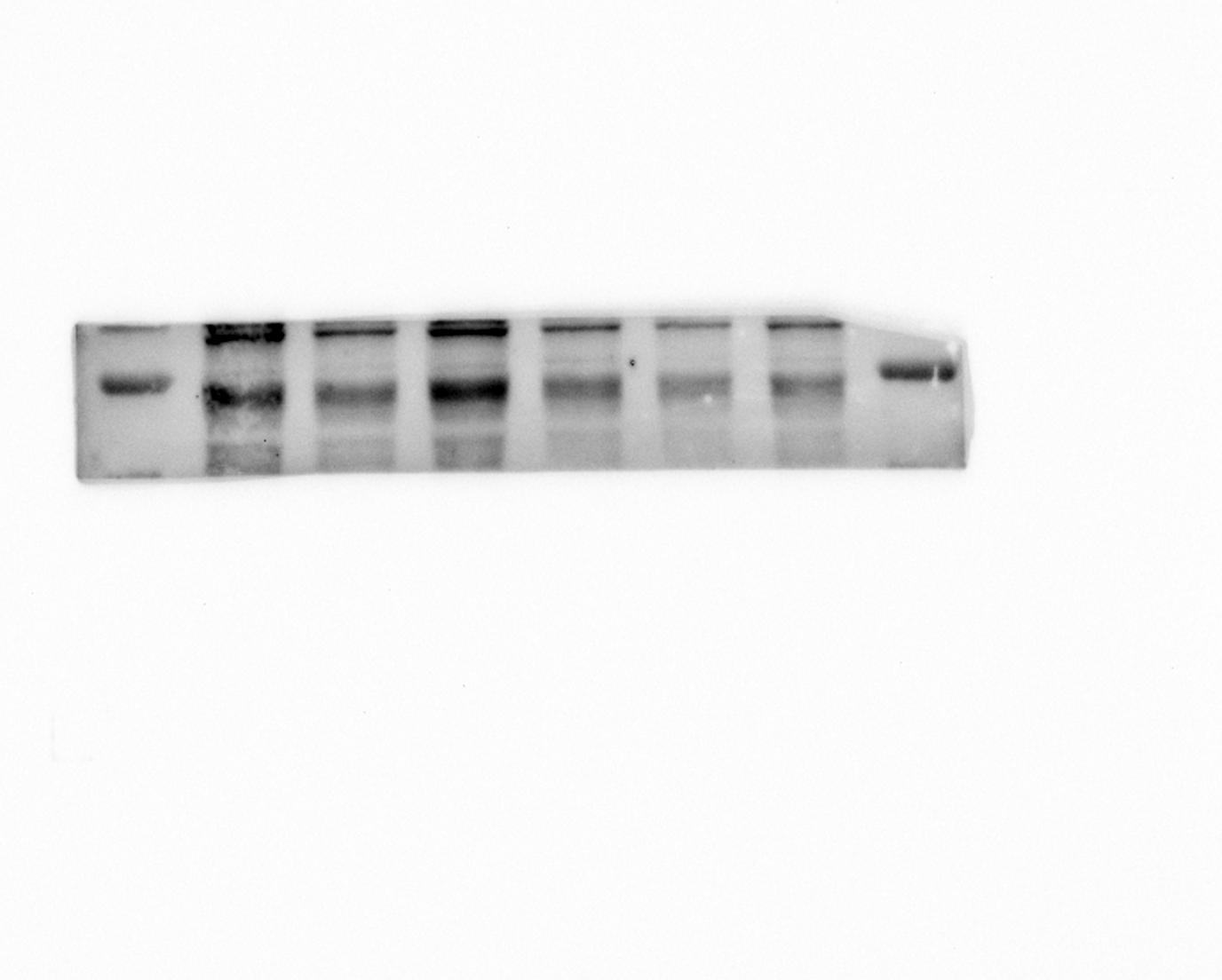

Supplement: Supplementary file 4 [file DataSheet6.zip › Fig.9/Fig.9 B/2-p-PI3K/2-p-PI3K-120S.Tif]

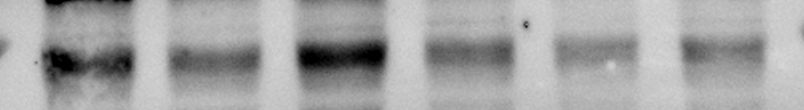

Supplement: Supplementary file 4 [file DataSheet6.zip › Fig.9/Fig.9 B/2-p-PI3K/PS 2-p-PI3K-120S.tif]

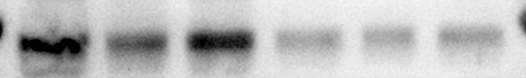

Supplement: Supplementary file 4 [file DataSheet6.zip › Fig.9/Fig.9 B/2-p-PI3K/PS-左-1-p-PI3K-120S.tif]

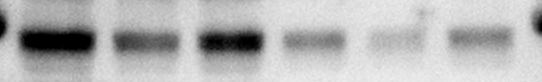

Supplement: Supplementary file 4 [file DataSheet6.zip › Fig.9/Fig.9 B/2-p-PI3K/用 PS-右-2-p-PI3K-120S.tif]

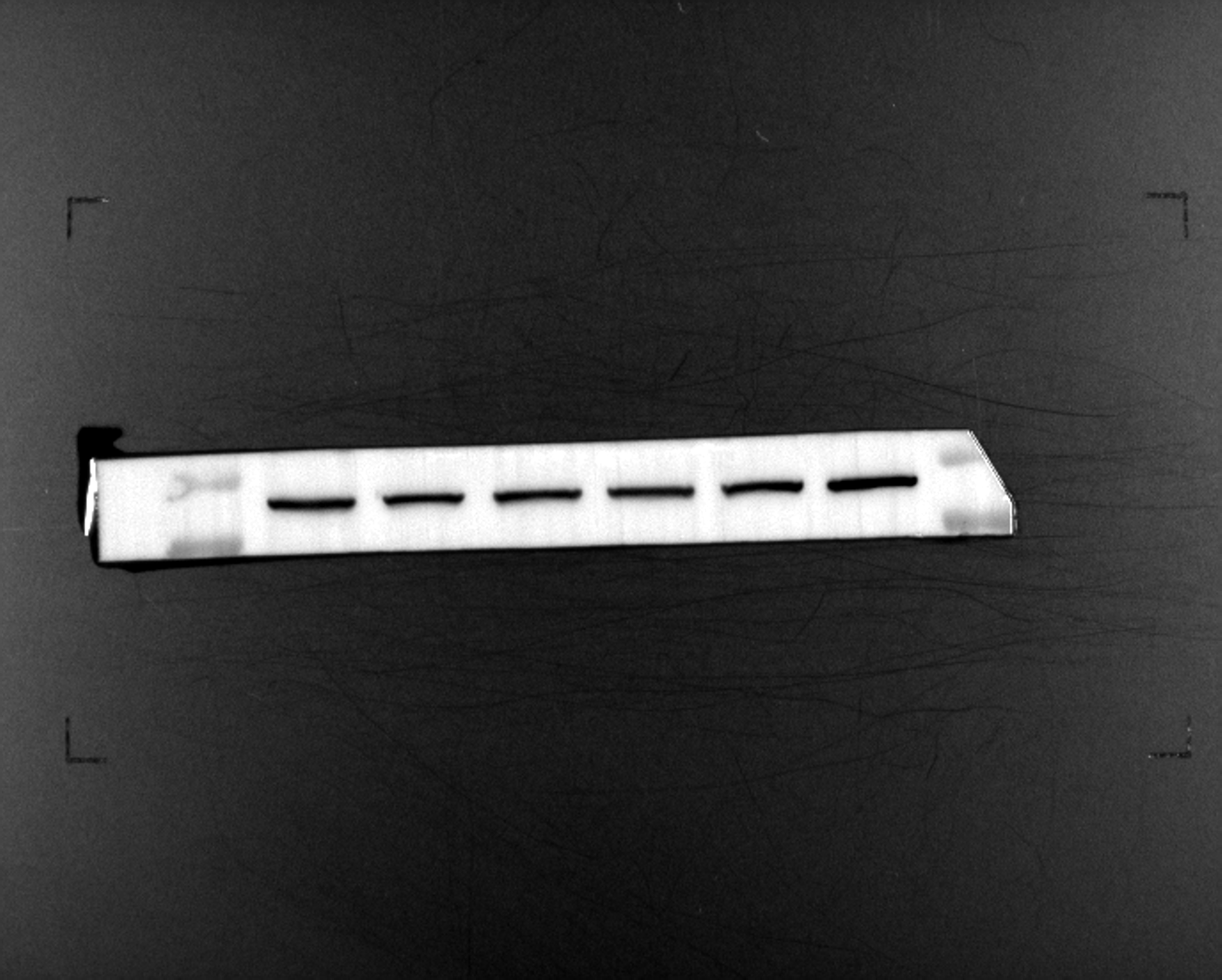

Supplement: Supplementary file 4 [file DataSheet6.zip › Fig.9/Fig.9 B/3-PI3K/1-PI3K-60S YT.Tif]

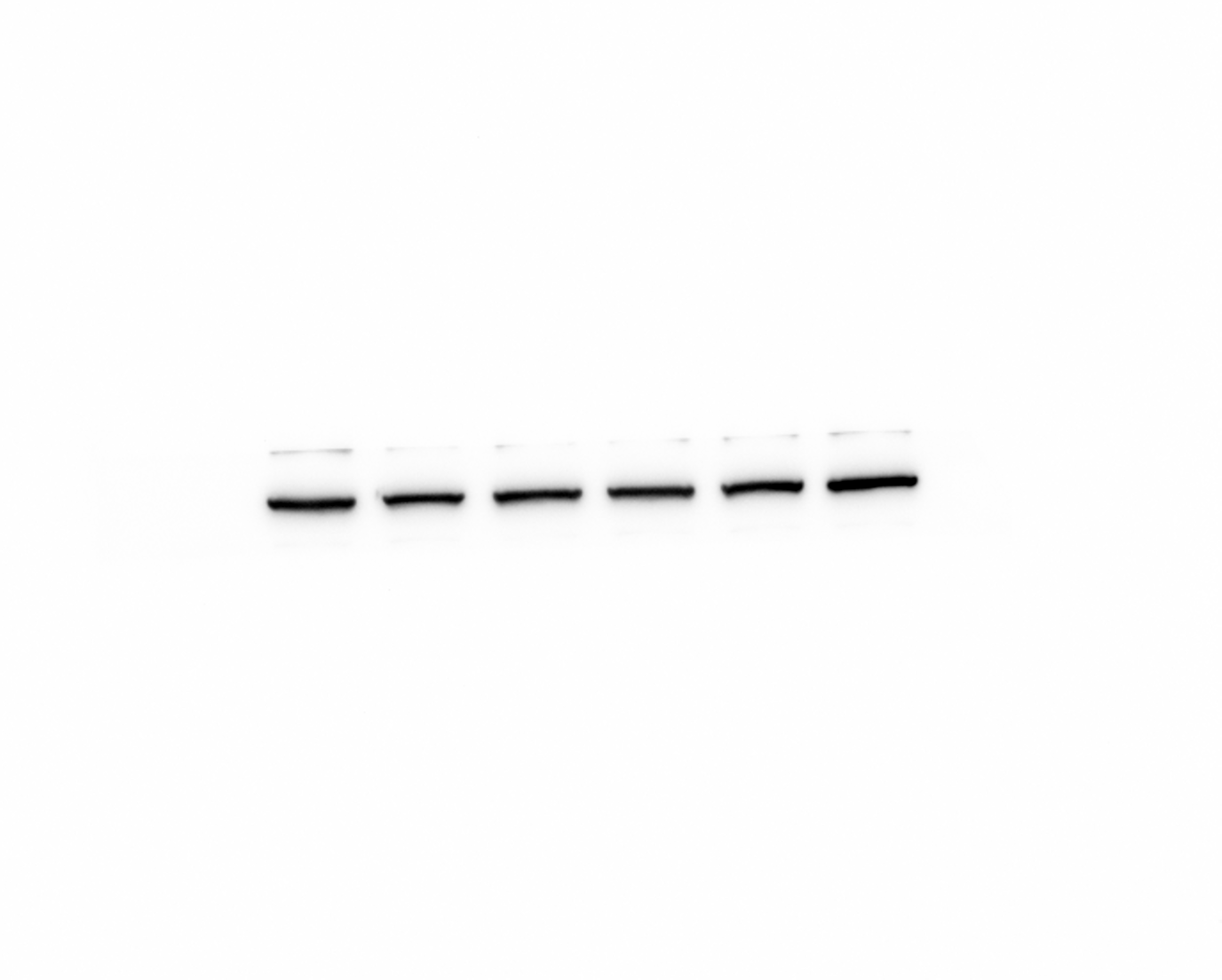

Supplement: Supplementary file 4 [file DataSheet6.zip › Fig.9/Fig.9 B/3-PI3K/1-PI3K-60S.Tif]

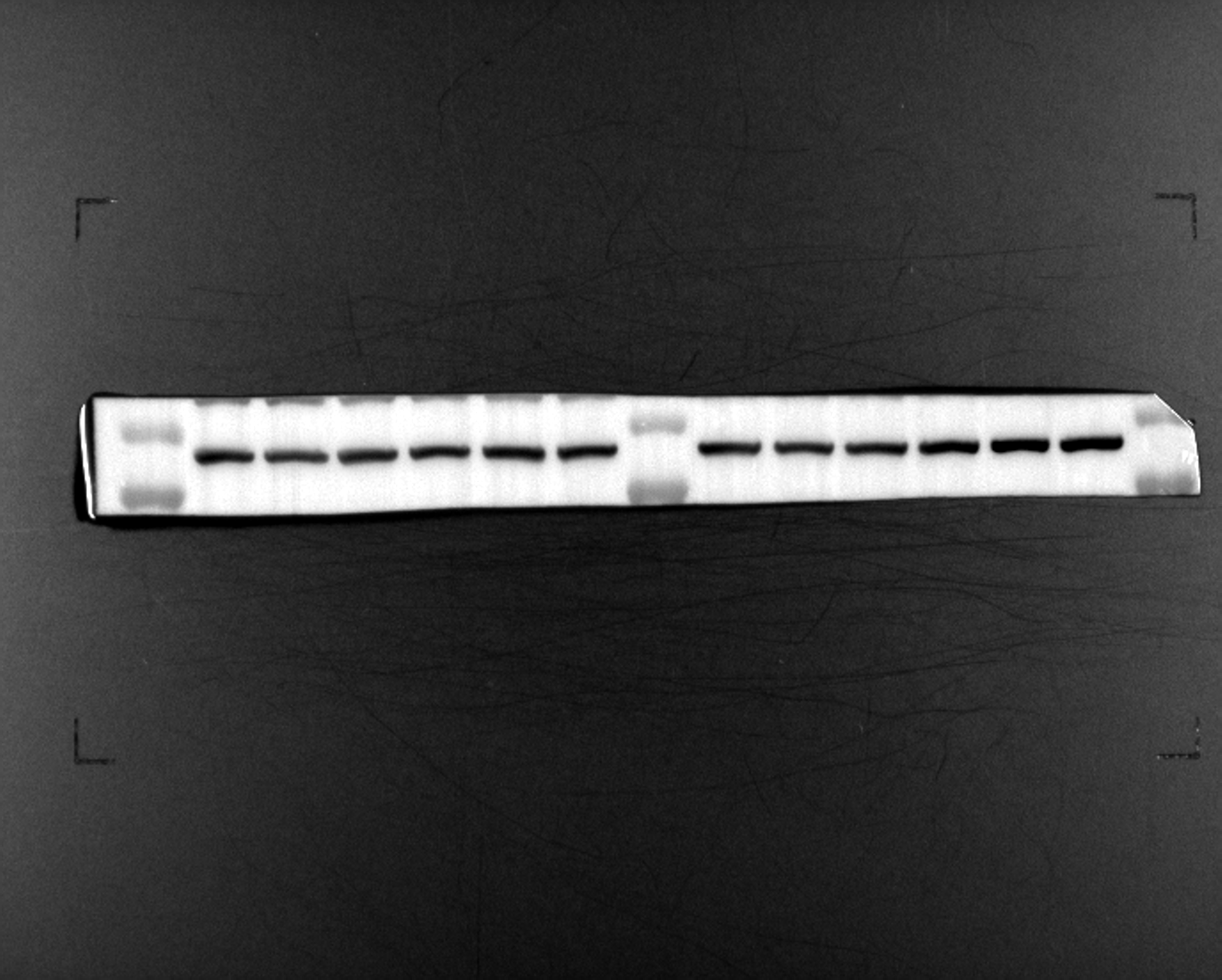

Supplement: Supplementary file 4 [file DataSheet6.zip › Fig.9/Fig.9 B/3-PI3K/2-PI3K-60S YT.Tif]

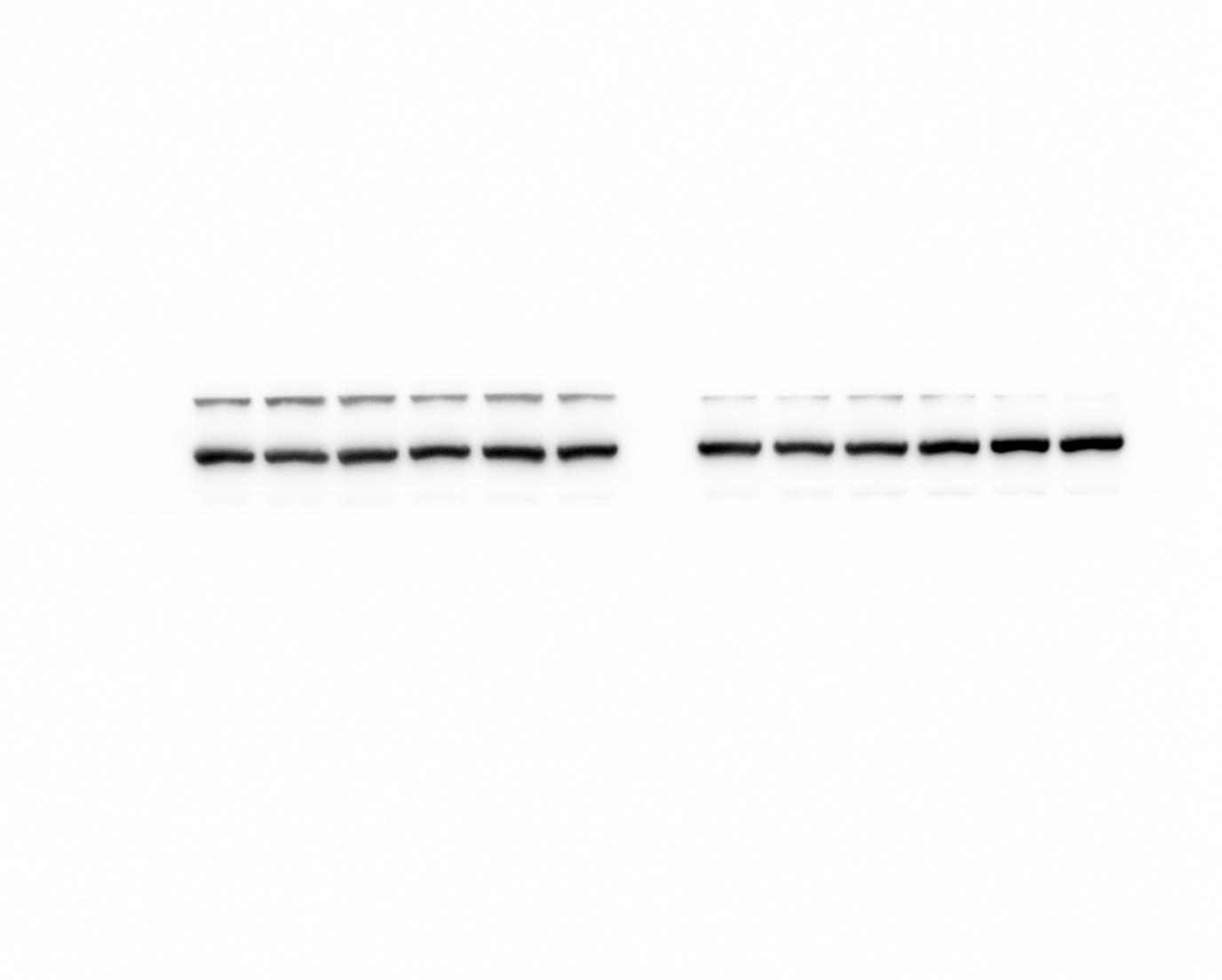

Supplement: Supplementary file 4 [file DataSheet6.zip › Fig.9/Fig.9 B/3-PI3K/2-PI3K-60S.Tif]

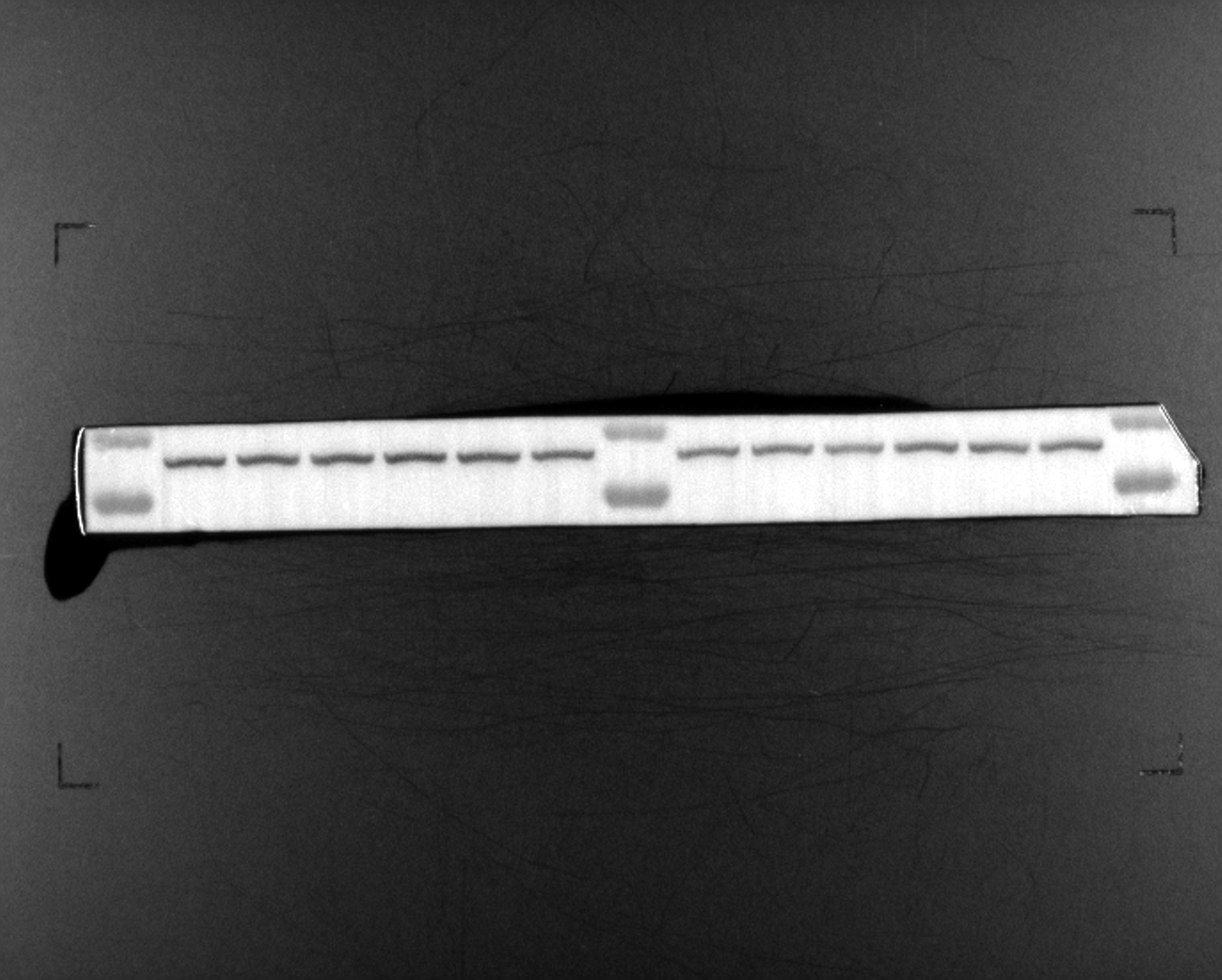

Supplement: Supplementary file 4 [file DataSheet6.zip › Fig.9/Fig.9 B/3-PI3K/3-PI3K-60S YT.Tif]

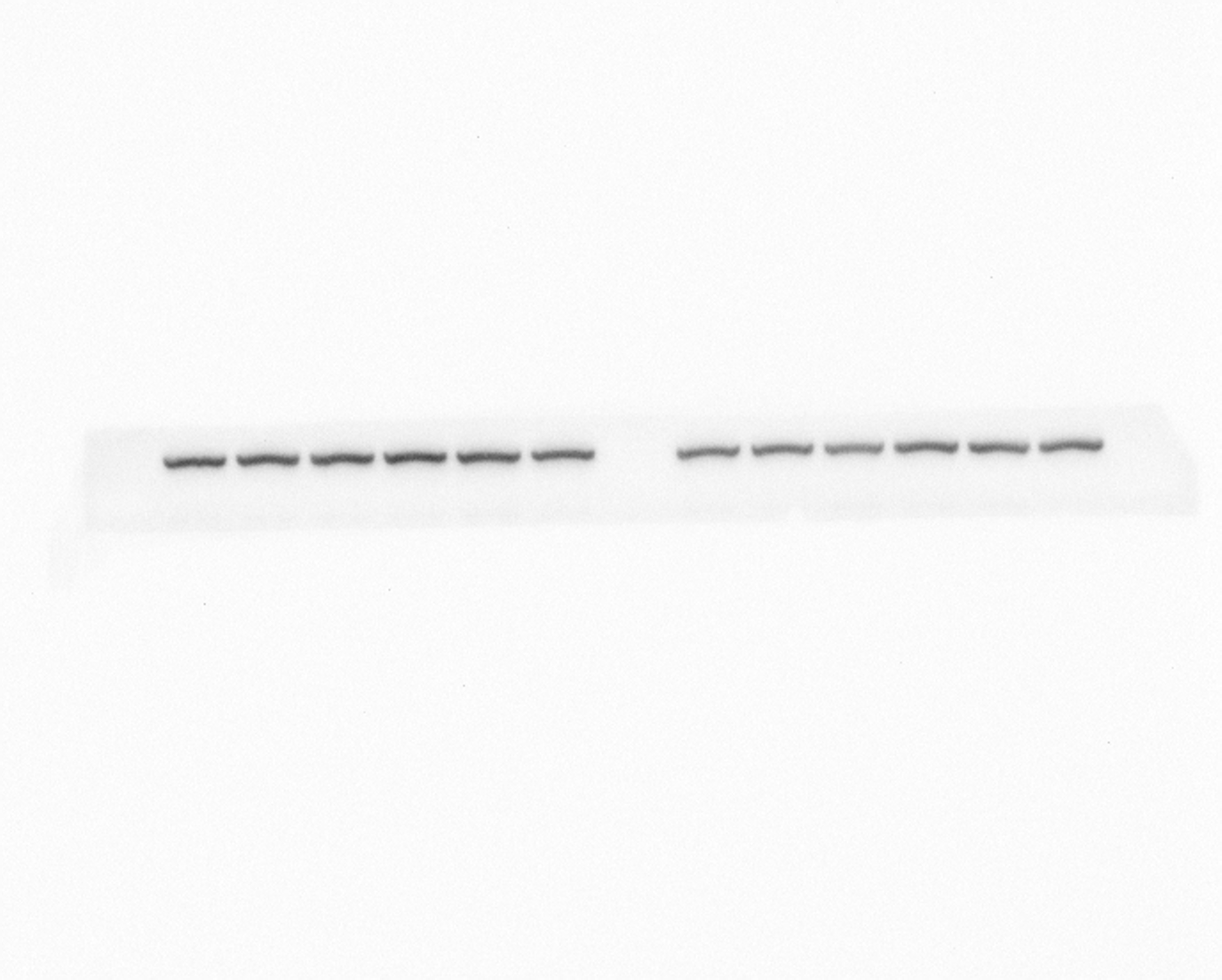

Supplement: Supplementary file 4 [file DataSheet6.zip › Fig.9/Fig.9 B/3-PI3K/3-PI3K-60S.Tif]

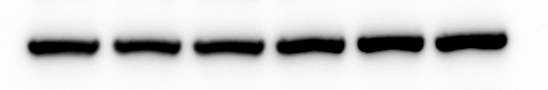

Supplement: Supplementary file 4 [file DataSheet6.zip › Fig.9/Fig.9 B/3-PI3K/PS-右-2-PI3K-60S.tif]

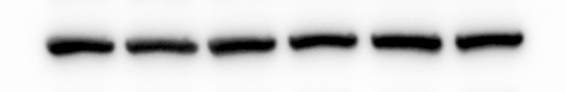

Supplement: Supplementary file 4 [file DataSheet6.zip › Fig.9/Fig.9 B/3-PI3K/PS-左-2-PI3K-60S.tif]

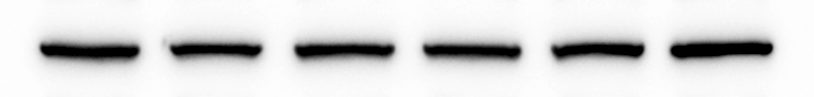

Supplement: Supplementary file 4 [file DataSheet6.zip › Fig.9/Fig.9 B/3-PI3K/用 PS 1-PI3K-60S.tif]

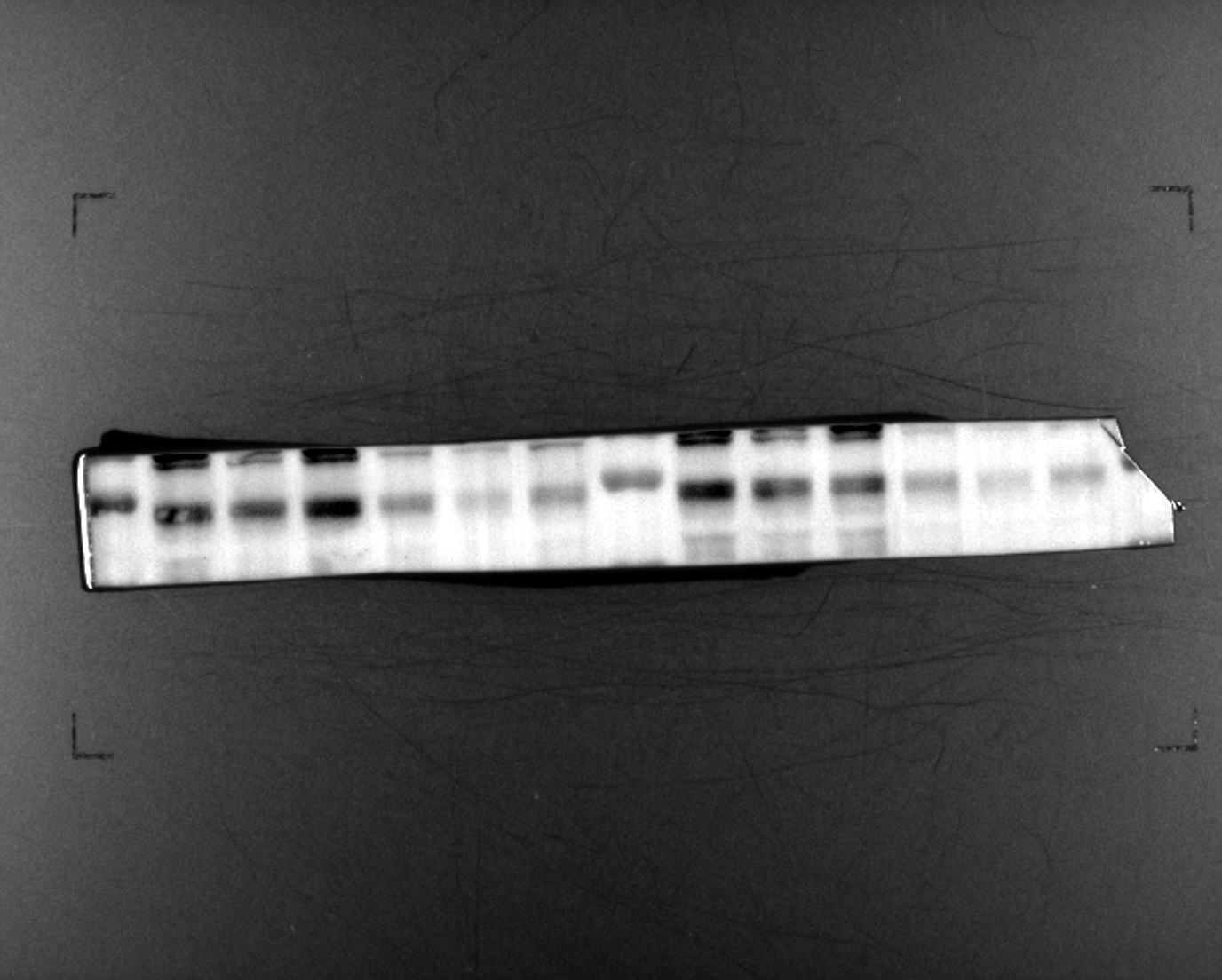

Supplement: Supplementary file 4 [file DataSheet6.zip › Fig.9/Fig.9 B/4-p-Akt/1-p-Akt-30s YT.Tif]

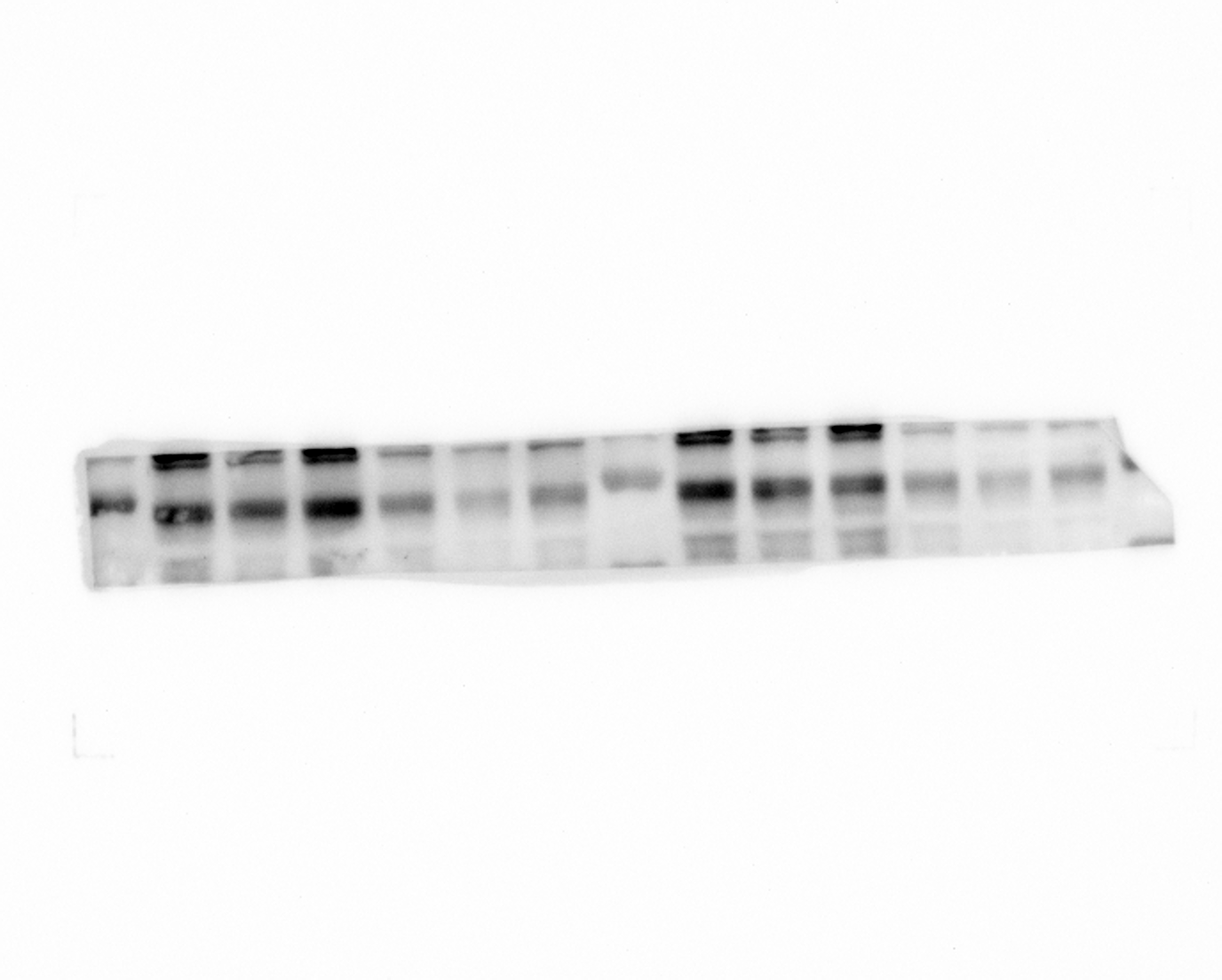

Supplement: Supplementary file 4 [file DataSheet6.zip › Fig.9/Fig.9 B/4-p-Akt/1-p-Akt-30s.Tif]

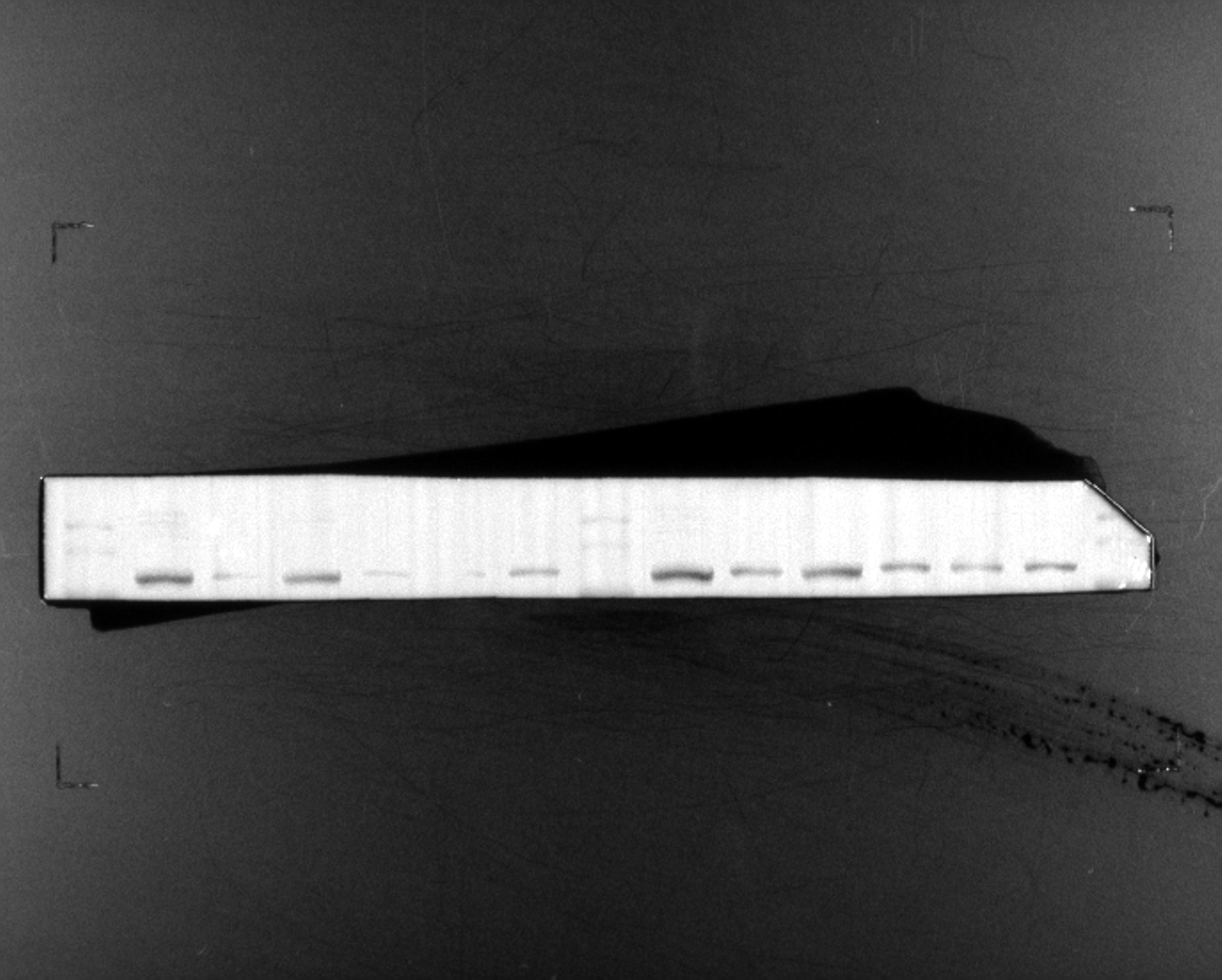

Supplement: Supplementary file 4 [file DataSheet6.zip › Fig.9/Fig.9 B/4-p-Akt/2-p-Akt-30s YT.Tif]

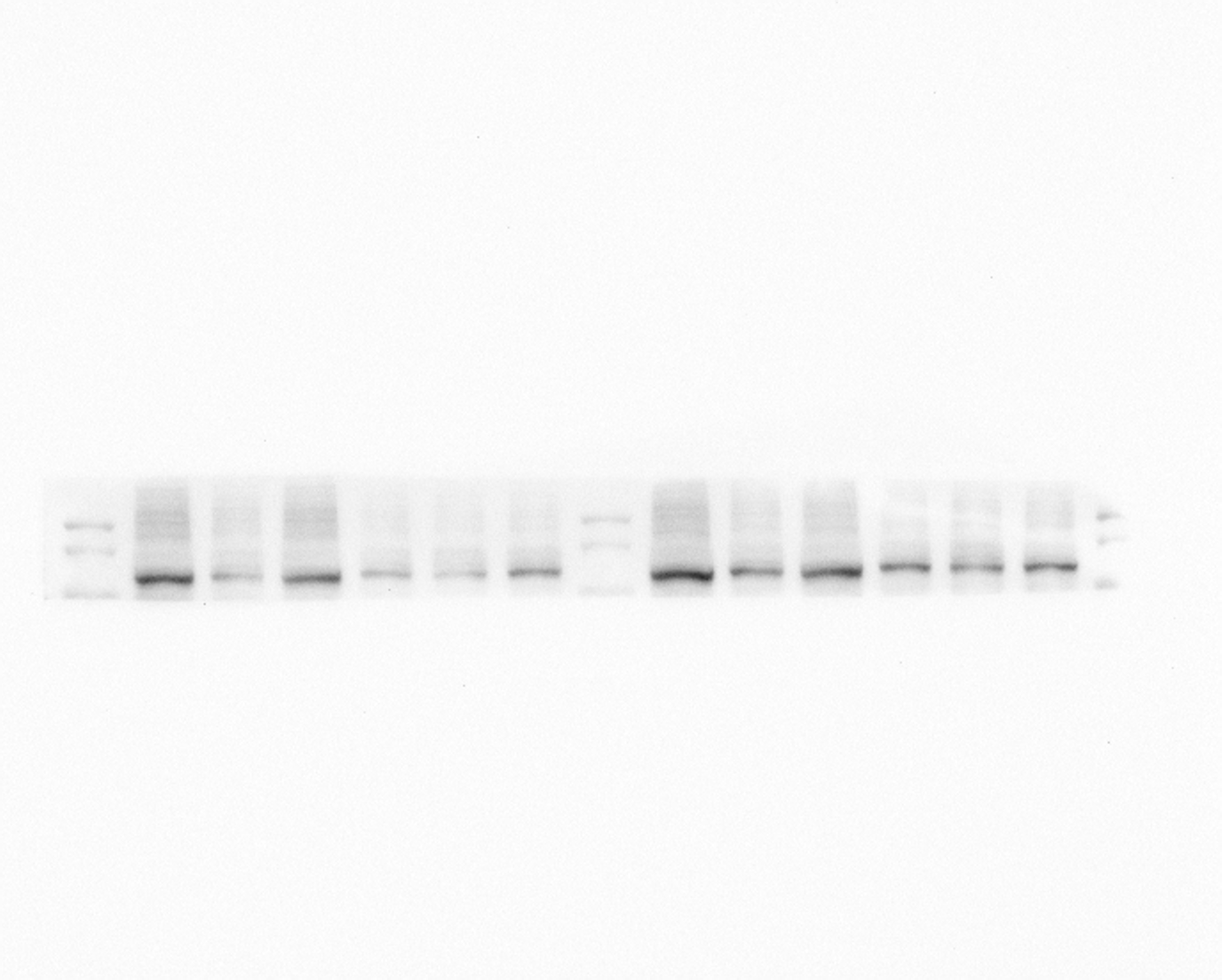

Supplement: Supplementary file 4 [file DataSheet6.zip › Fig.9/Fig.9 B/4-p-Akt/2-p-Akt-30s.Tif]

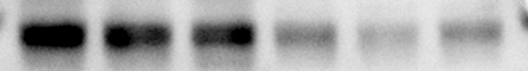

Supplement: Supplementary file 4 [file DataSheet6.zip › Fig.9/Fig.9 B/4-p-Akt/PS-右-1-p-Akt-30s.tif]

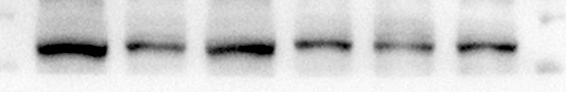

Supplement: Supplementary file 4 [file DataSheet6.zip › Fig.9/Fig.9 B/4-p-Akt/PS-右-2-p-Akt-30s.tif]

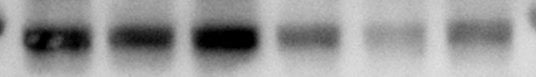

Supplement: Supplementary file 4 [file DataSheet6.zip › Fig.9/Fig.9 B/4-p-Akt/PS-左-1-p-Akt-30s.tif]

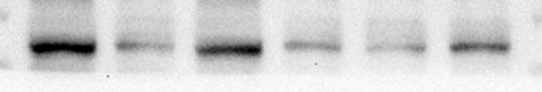

Supplement: Supplementary file 4 [file DataSheet6.zip › Fig.9/Fig.9 B/4-p-Akt/用 PS-左-2-p-Akt-30s.tif]

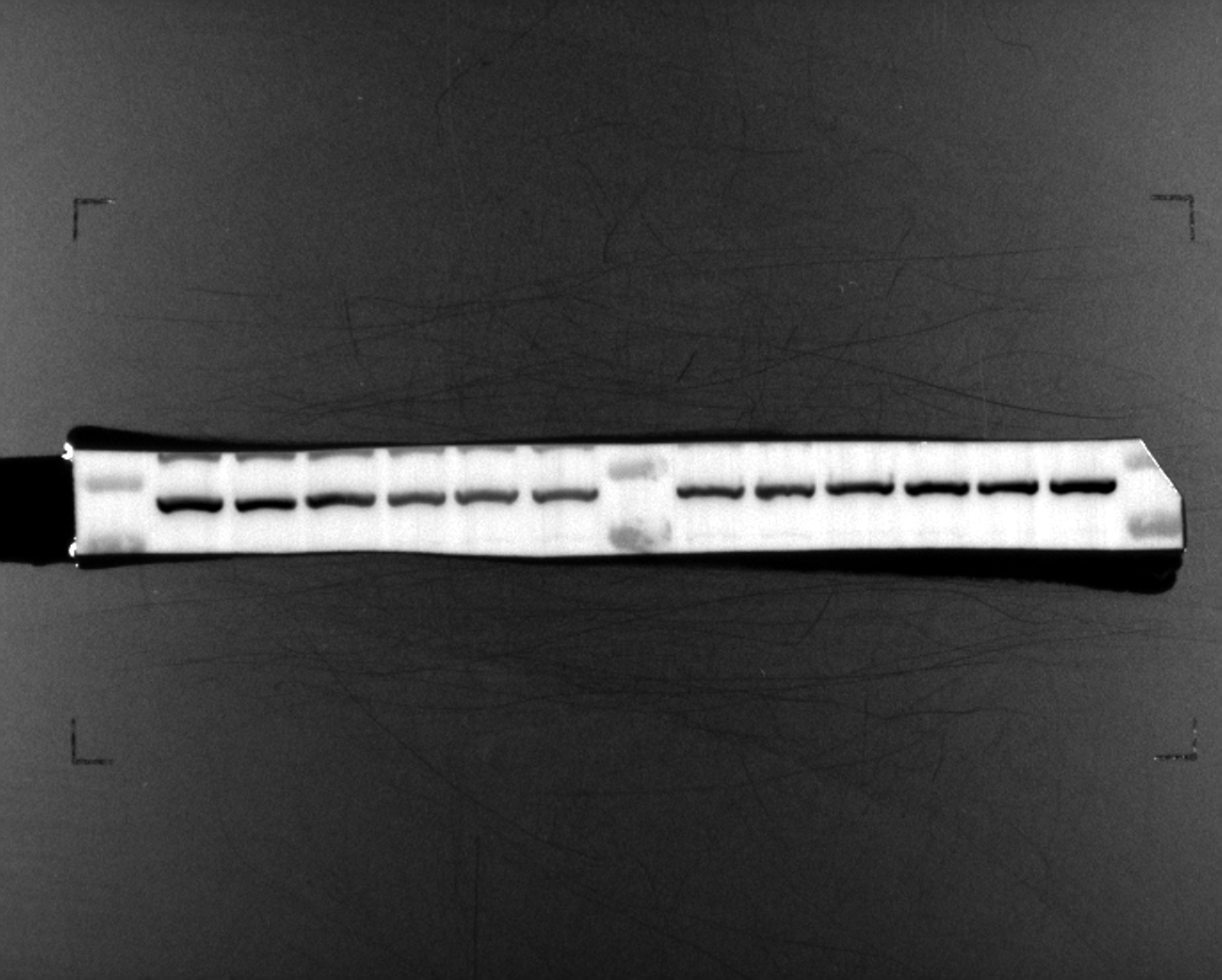

Supplement: Supplementary file 4 [file DataSheet6.zip › Fig.9/Fig.9 B/5-Akt/1-Akt-30s YT.Tif]

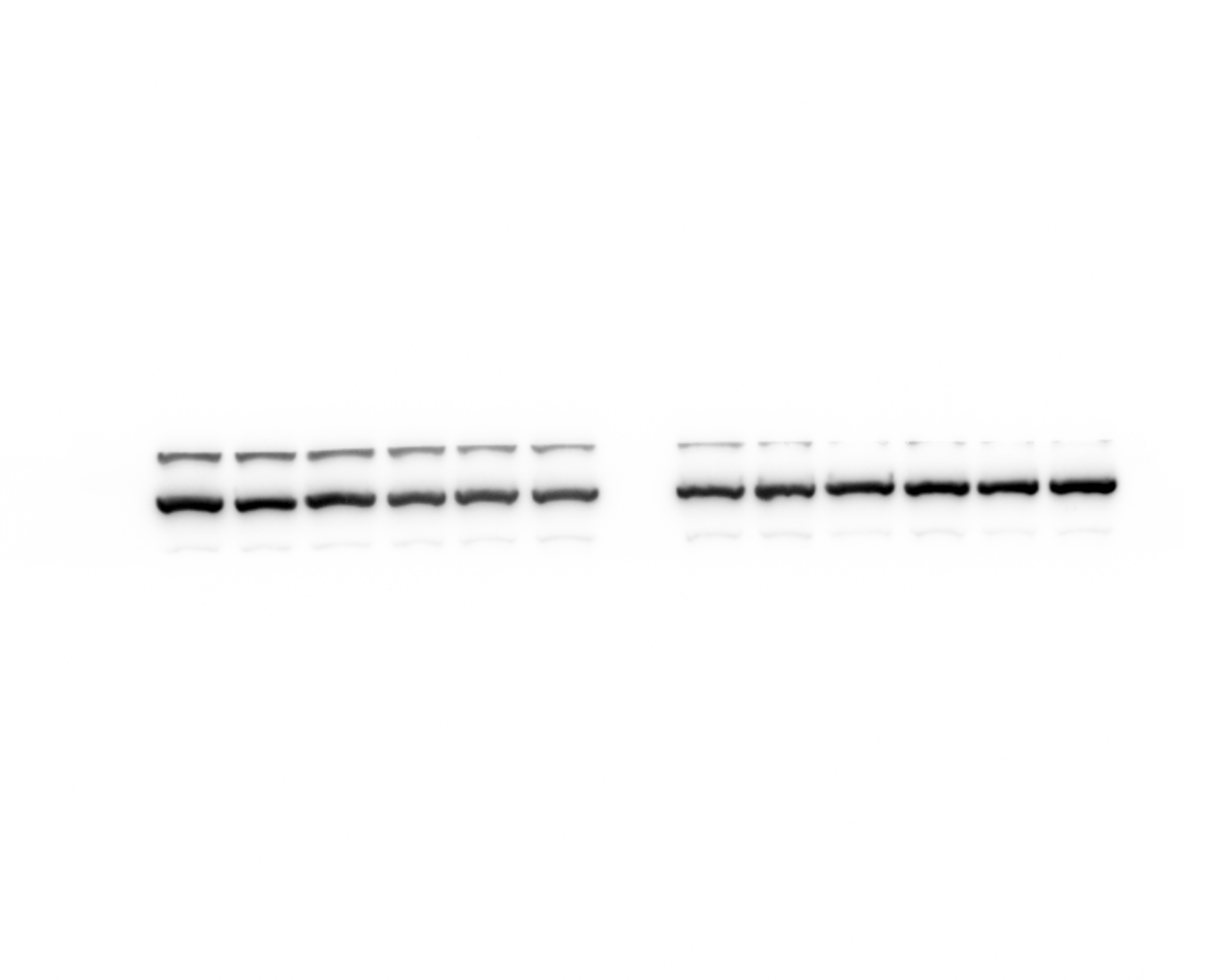

Supplement: Supplementary file 4 [file DataSheet6.zip › Fig.9/Fig.9 B/5-Akt/1-Akt-30s.Tif]

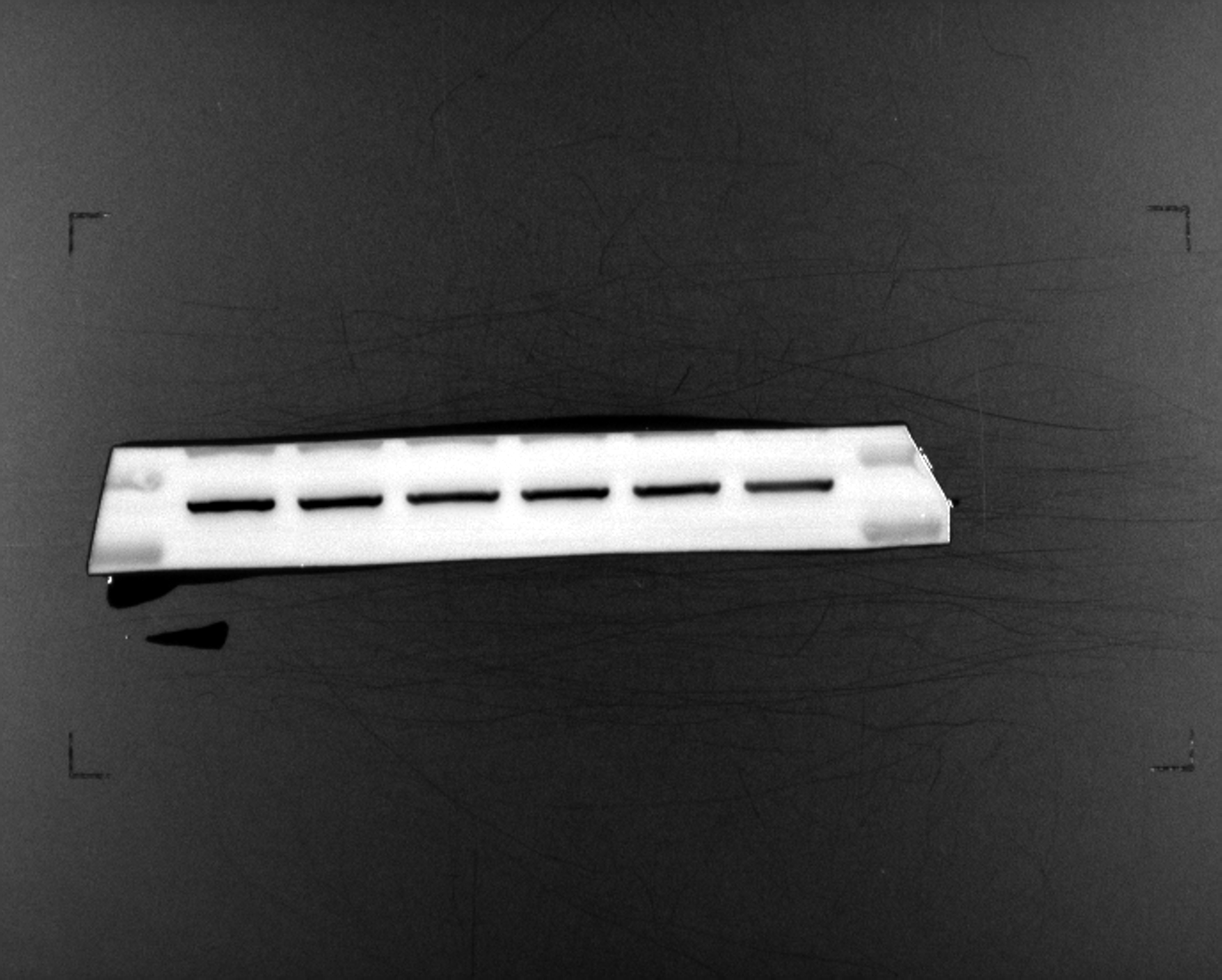

Supplement: Supplementary file 4 [file DataSheet6.zip › Fig.9/Fig.9 B/5-Akt/2-Akt-30s YT.Tif]

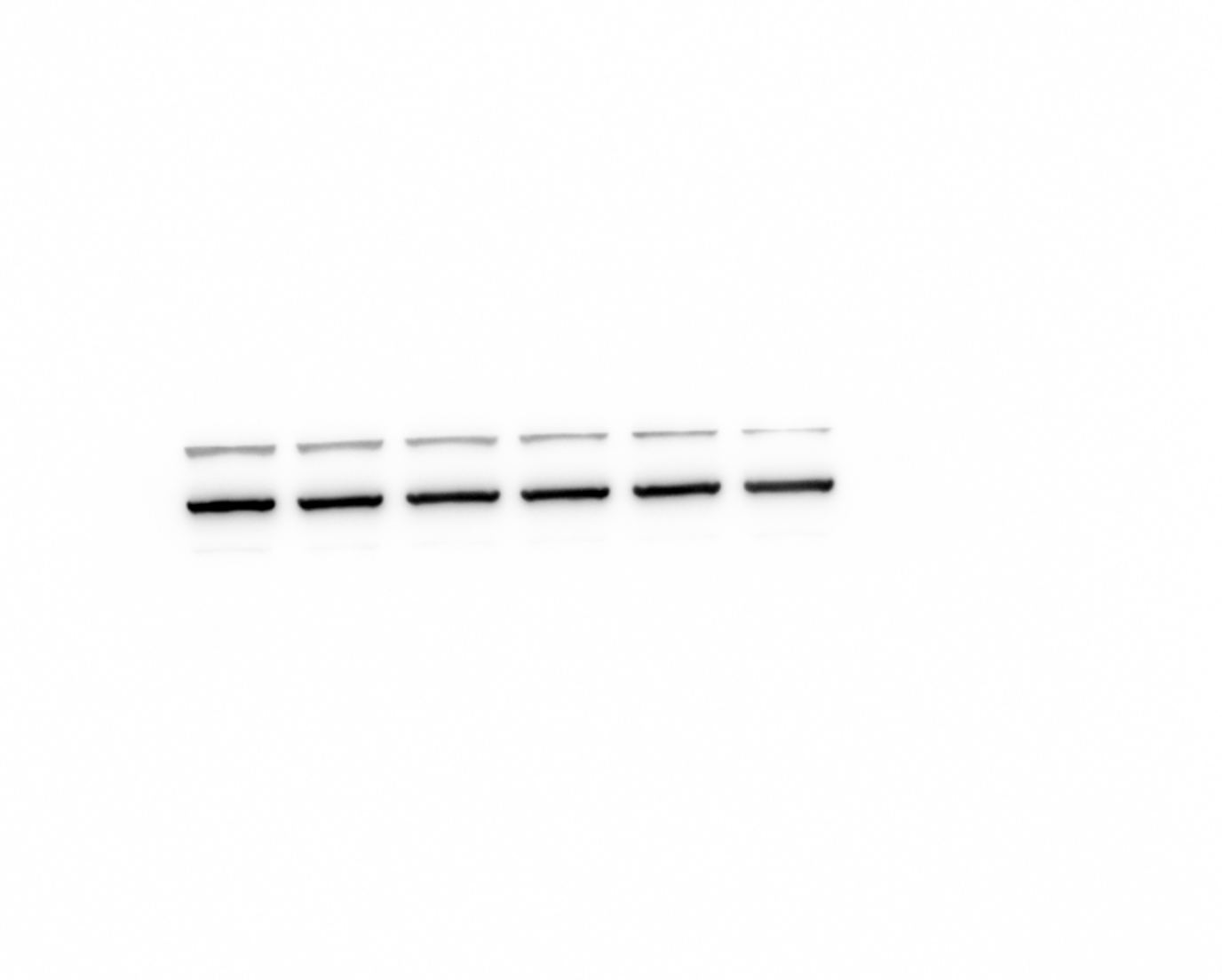

Supplement: Supplementary file 4 [file DataSheet6.zip › Fig.9/Fig.9 B/5-Akt/2-Akt-30s.Tif]

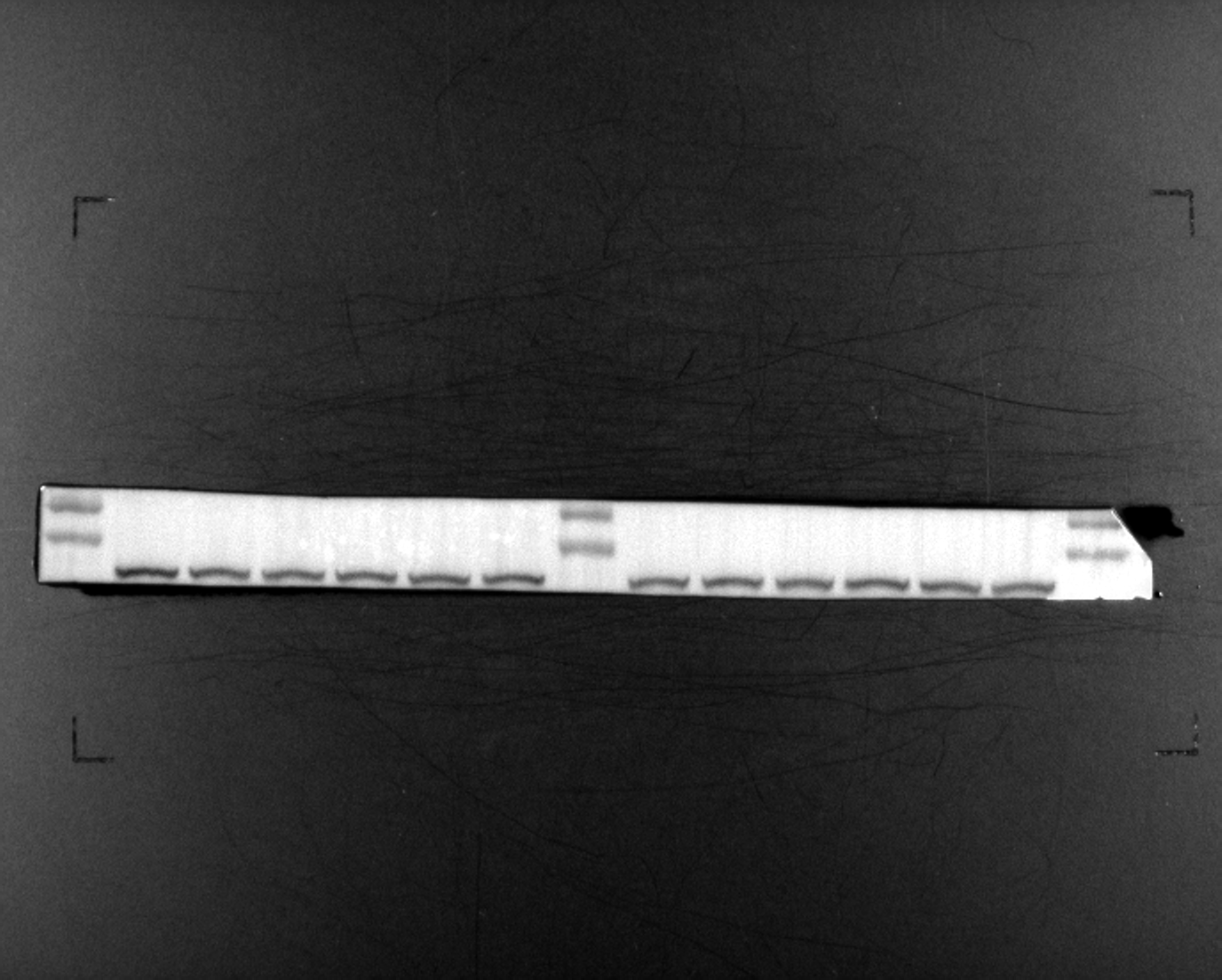

Supplement: Supplementary file 4 [file DataSheet6.zip › Fig.9/Fig.9 B/5-Akt/3-Akt-30s YT.Tif]

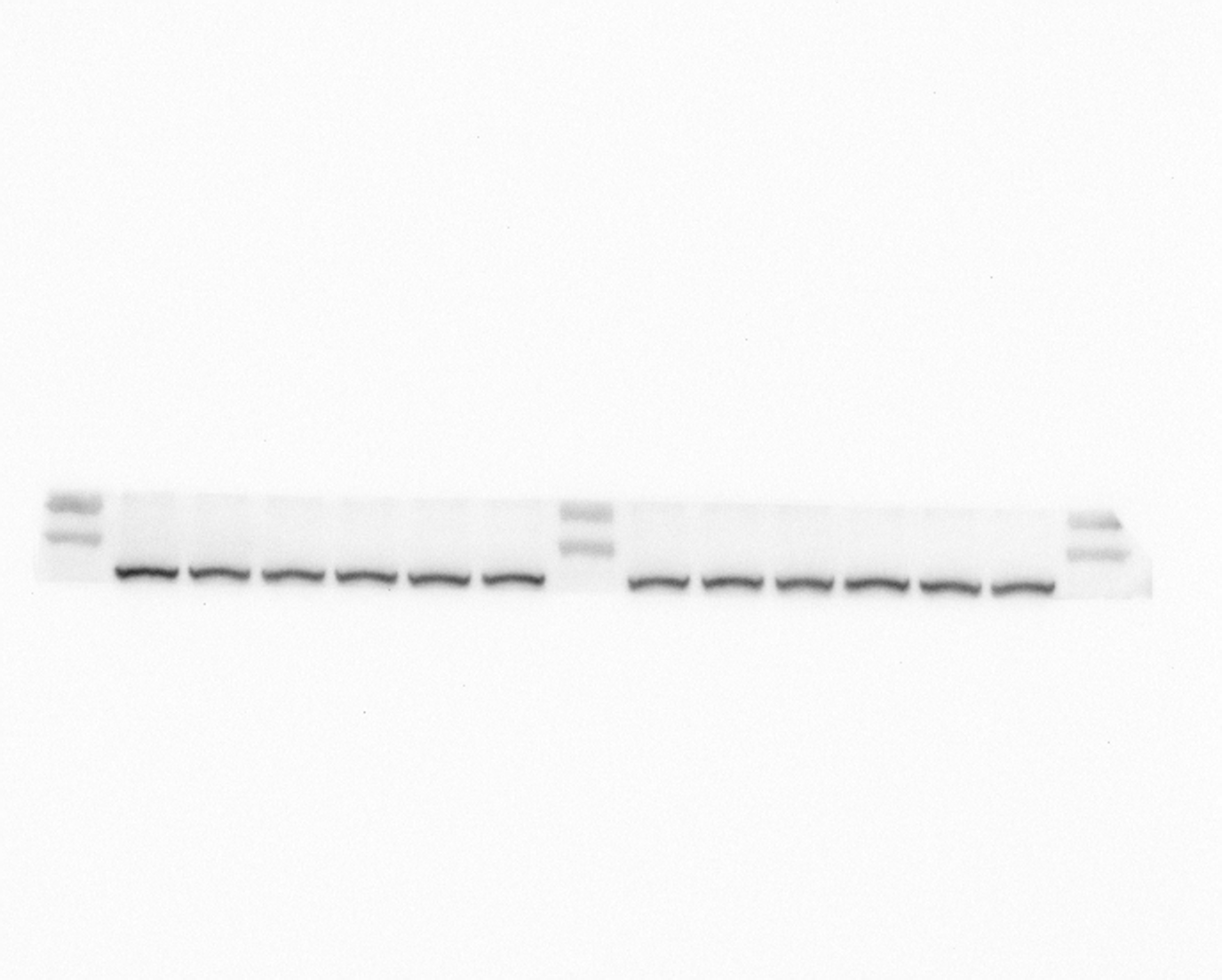

Supplement: Supplementary file 4 [file DataSheet6.zip › Fig.9/Fig.9 B/5-Akt/3-Akt-30s.Tif]

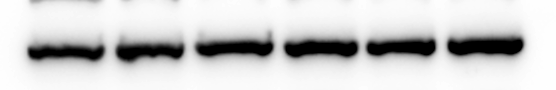

Supplement: Supplementary file 4 [file DataSheet6.zip › Fig.9/Fig.9 B/5-Akt/PS-右-1-Akt-30s.tif]

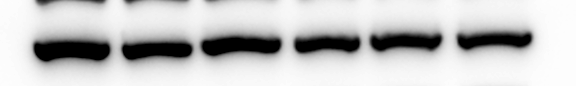

Supplement: Supplementary file 4 [file DataSheet6.zip › Fig.9/Fig.9 B/5-Akt/PS-左-1-Akt-30s.tif]

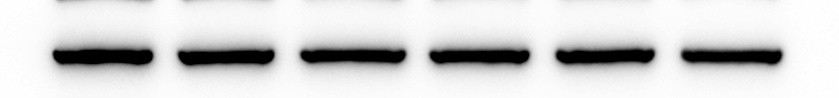

Supplement: Supplementary file 4 [file DataSheet6.zip › Fig.9/Fig.9 B/5-Akt/用 PS 2-Akt-30s.tif]

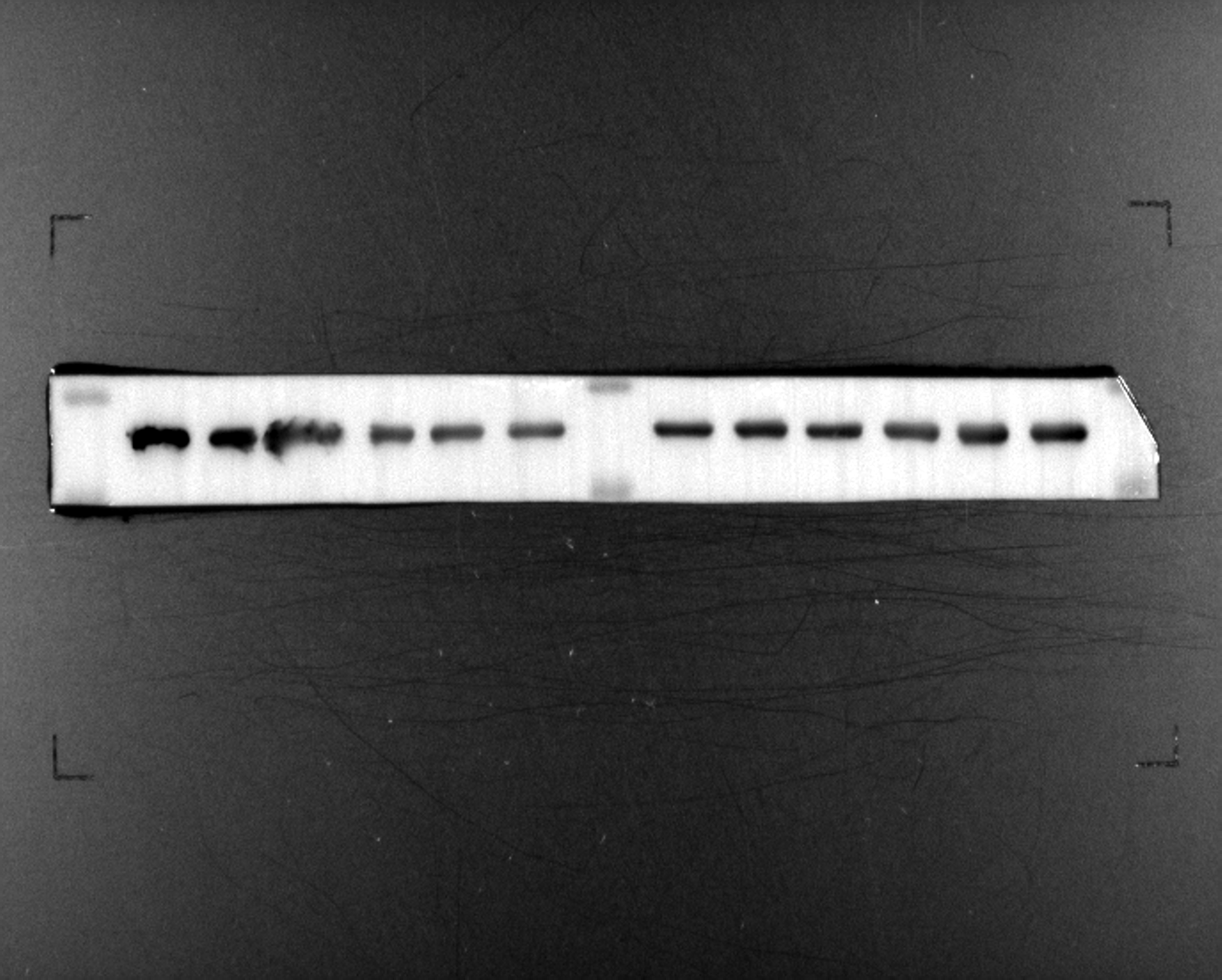

Supplement: Supplementary file 4 [file DataSheet6.zip › Fig.9/Fig.9 B/6-GAPDH/1-G-10S YT.Tif]

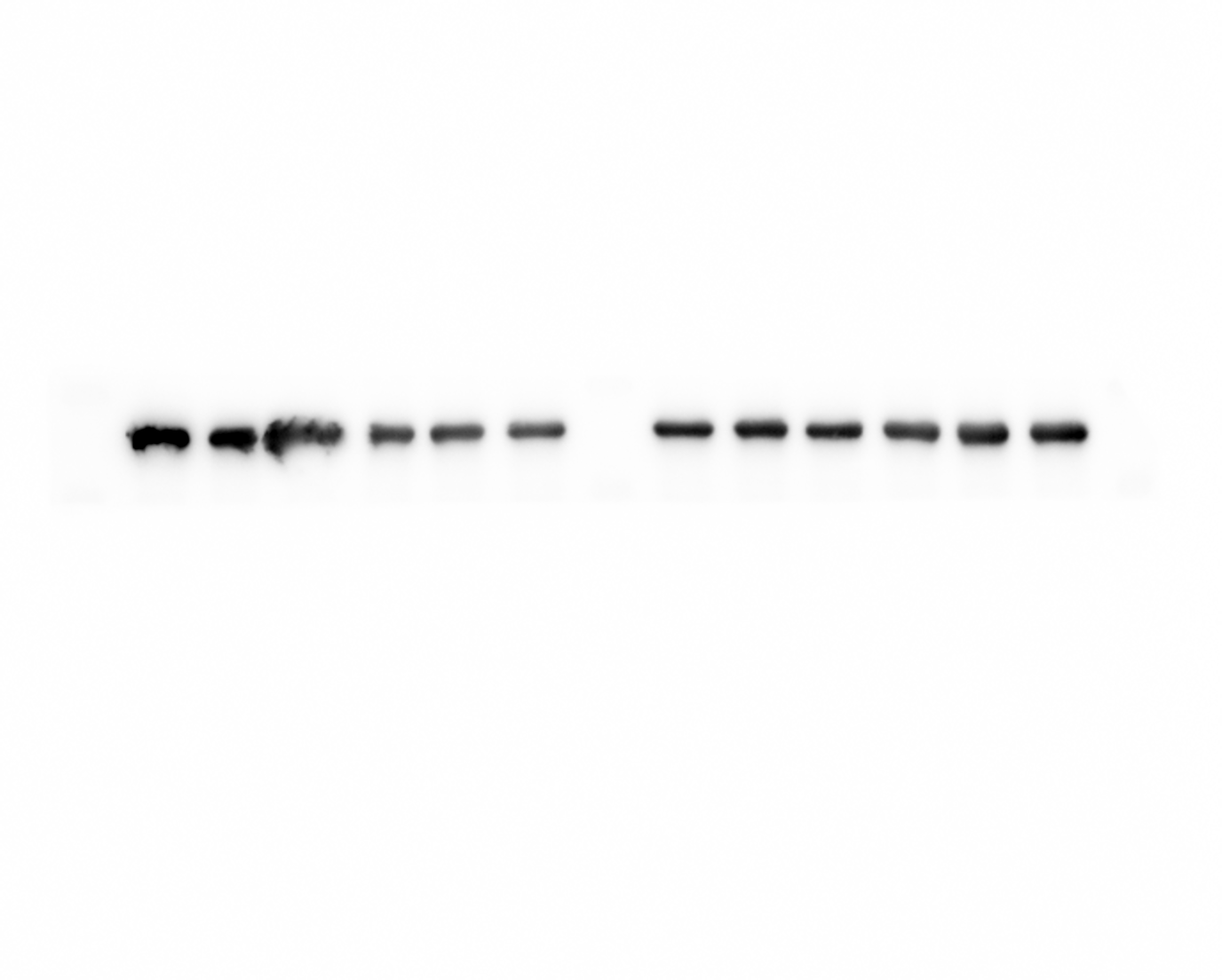

Supplement: Supplementary file 4 [file DataSheet6.zip › Fig.9/Fig.9 B/6-GAPDH/1-G-10S.Tif]

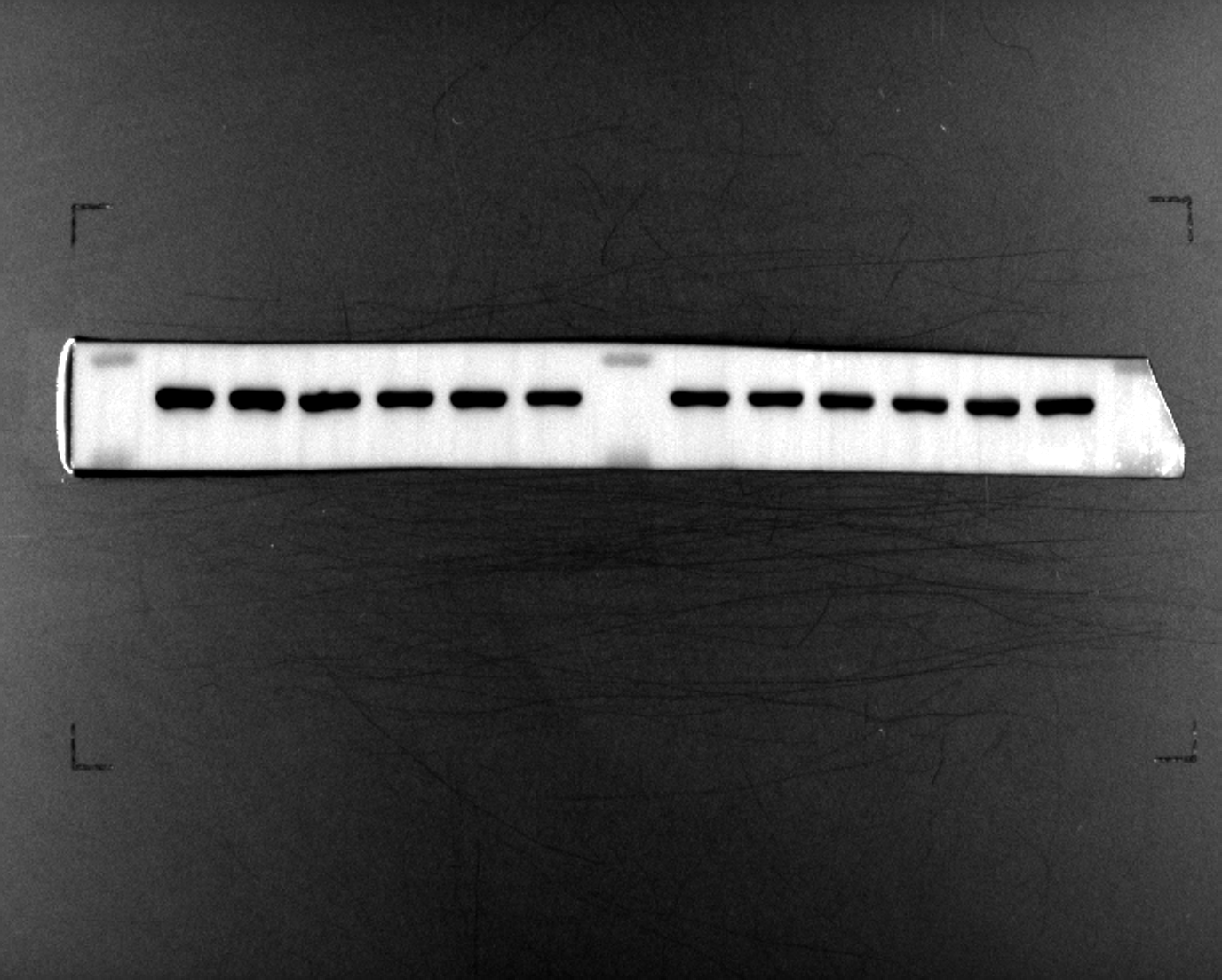

Supplement: Supplementary file 4 [file DataSheet6.zip › Fig.9/Fig.9 B/6-GAPDH/2-G-10S YT.Tif]

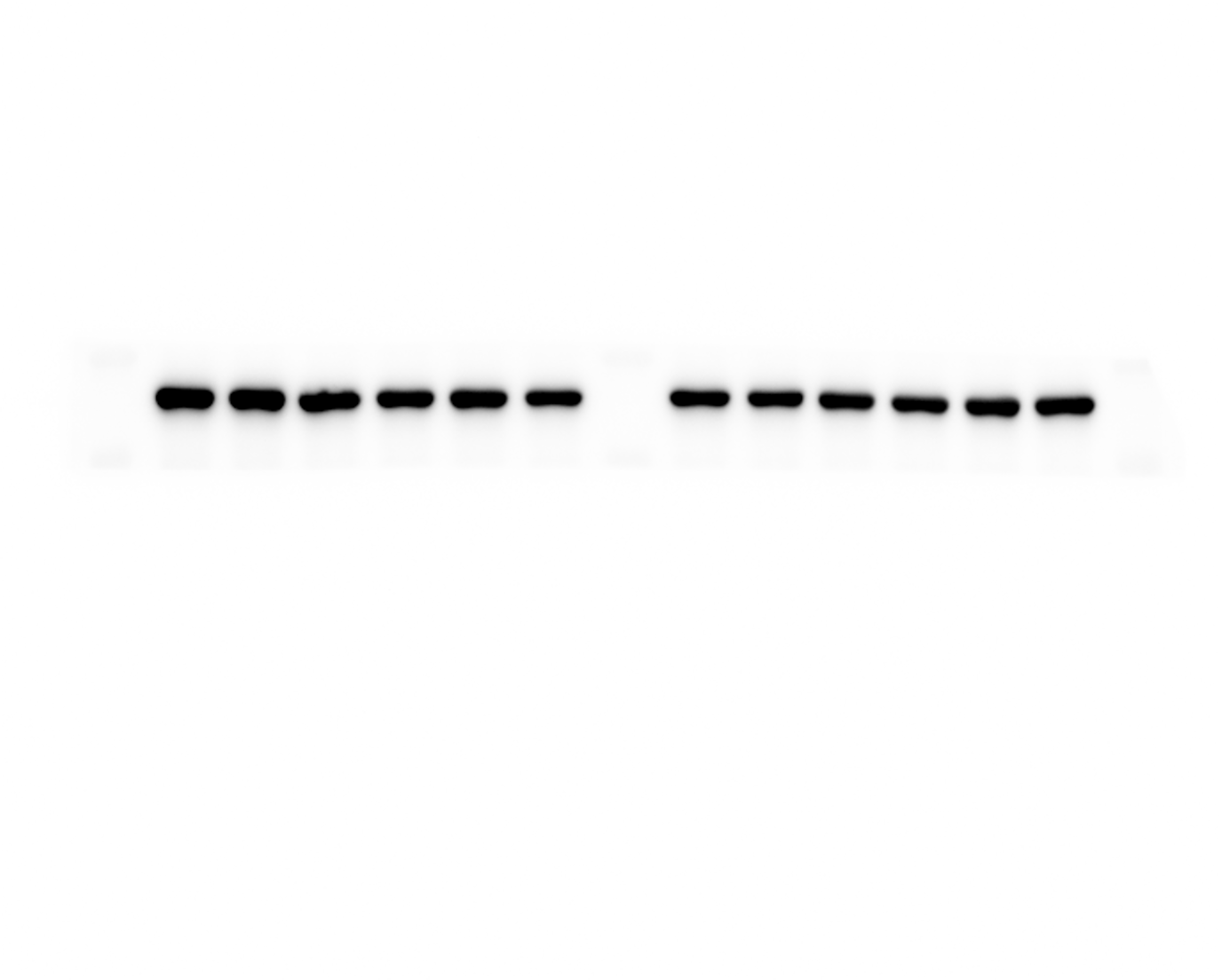

Supplement: Supplementary file 4 [file DataSheet6.zip › Fig.9/Fig.9 B/6-GAPDH/2-G-10S.Tif]

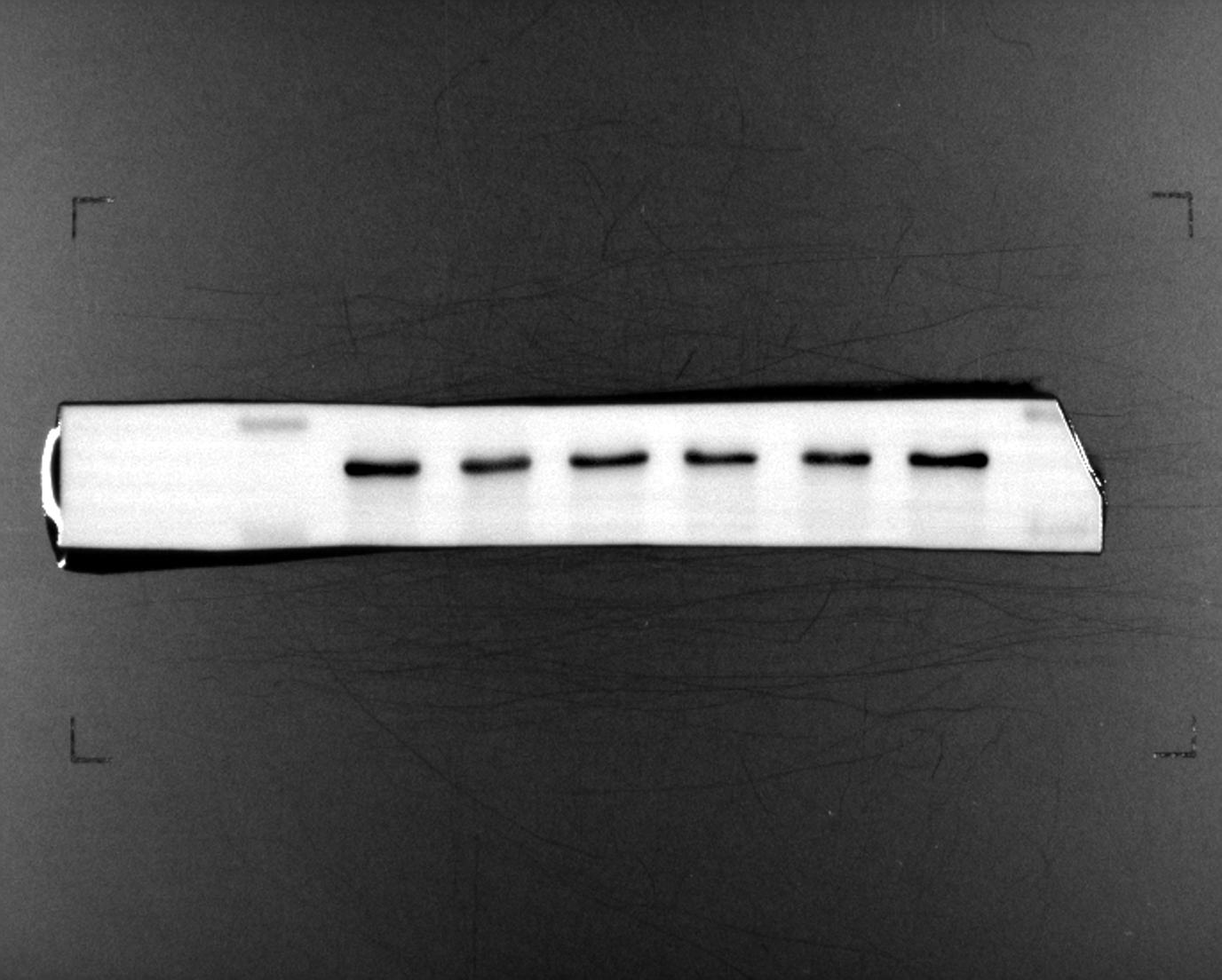

Supplement: Supplementary file 4 [file DataSheet6.zip › Fig.9/Fig.9 B/6-GAPDH/8-G-10S YT.Tif]

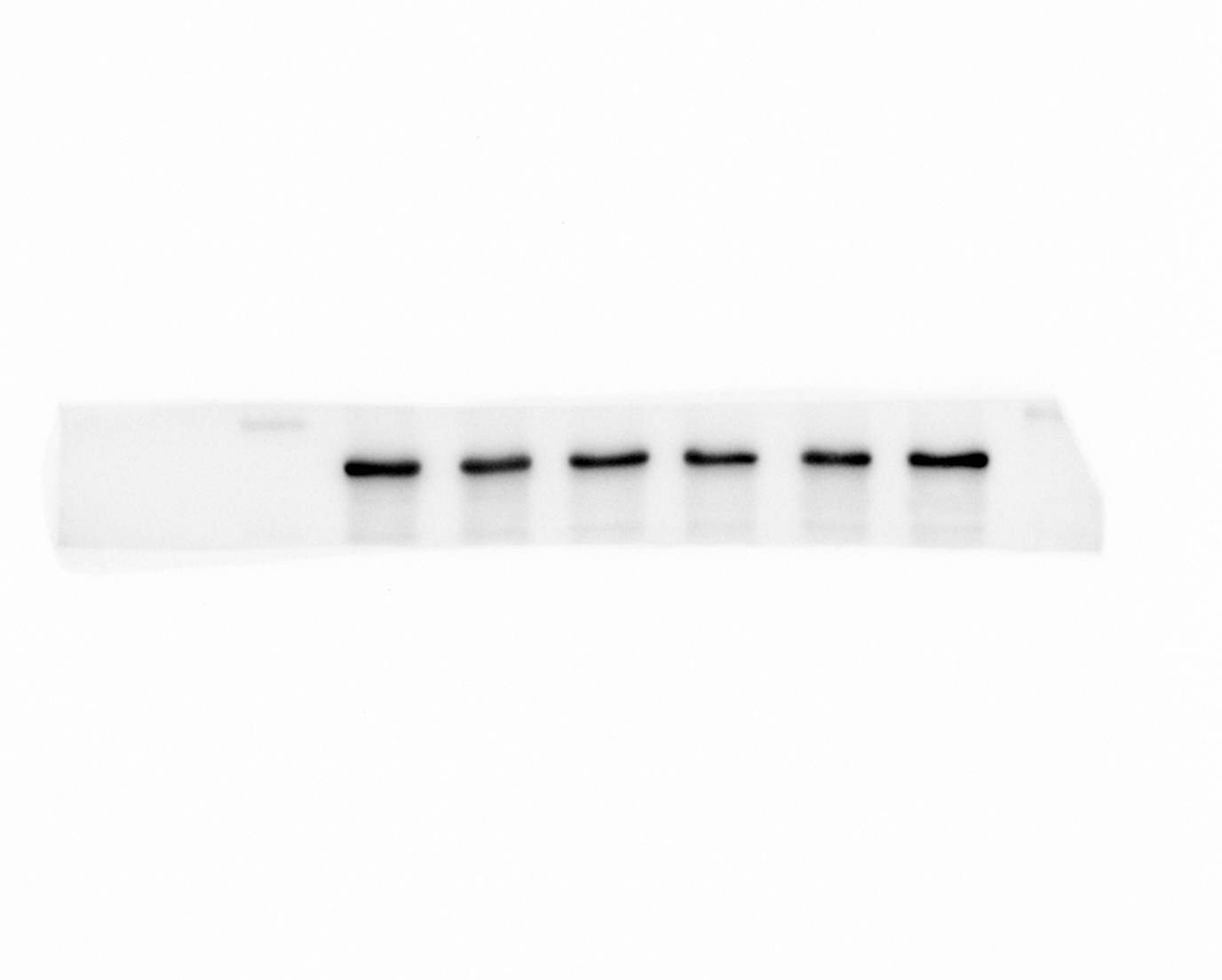

Supplement: Supplementary file 4 [file DataSheet6.zip › Fig.9/Fig.9 B/6-GAPDH/8-G-10S.Tif]

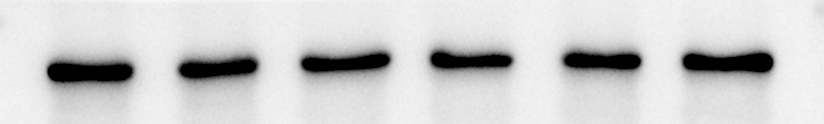

Supplement: Supplementary file 4 [file DataSheet6.zip › Fig.9/Fig.9 B/6-GAPDH/PS 8-G-10S.tif]

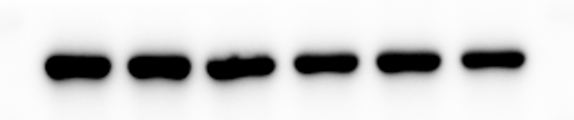

Supplement: Supplementary file 4 [file DataSheet6.zip › Fig.9/Fig.9 B/6-GAPDH/PS-左-2-G-10S.tif]

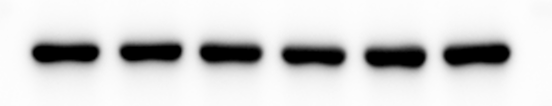

Supplement: Supplementary file 4 [file DataSheet6.zip › Fig.9/Fig.9 B/6-GAPDH/用 PS-右-2-G-10S.tif]

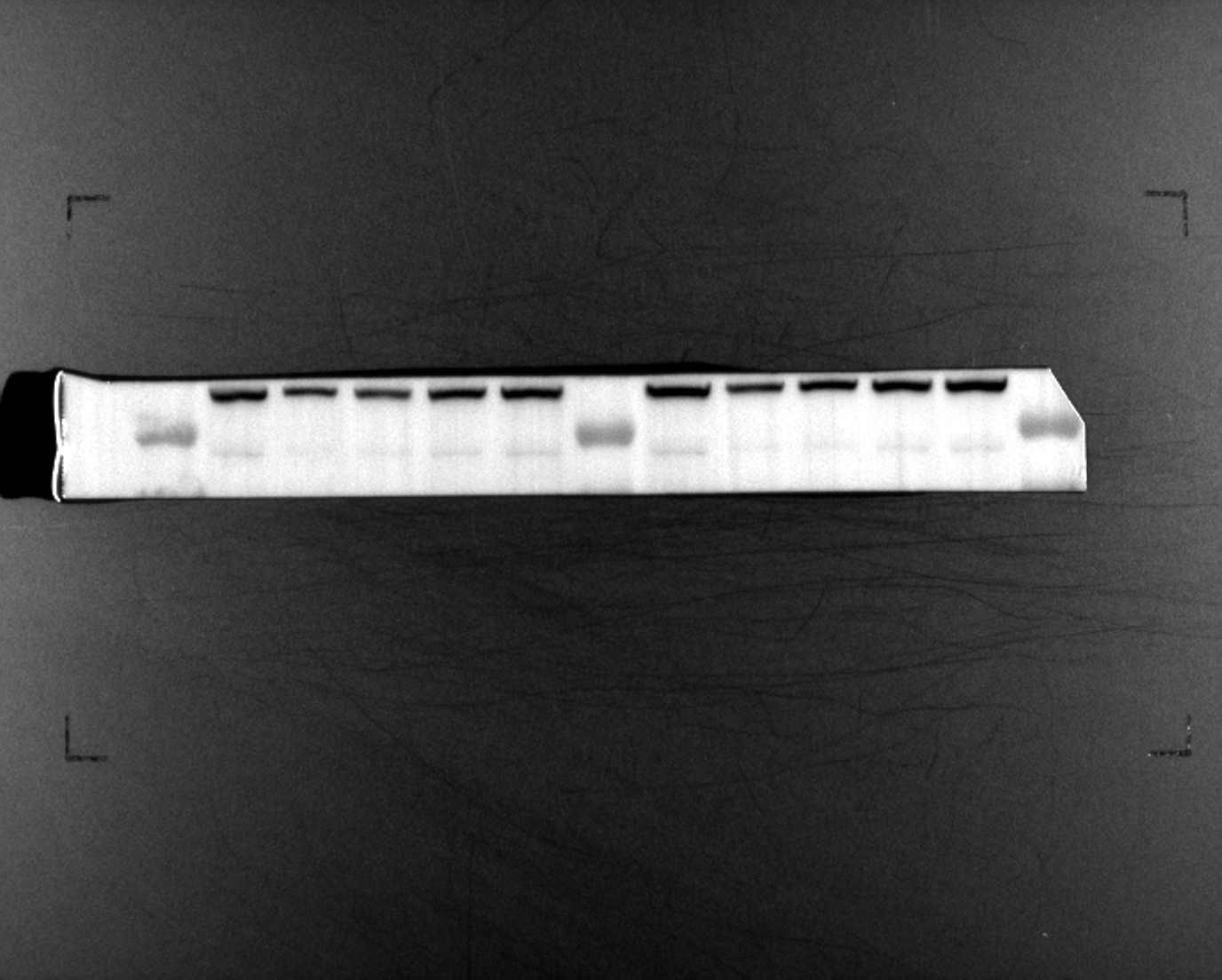

Supplement: Supplementary file 6 [file DataSheet2.zip › Fig.1/WBμ¥íσ╕a/Podocalyxin/1-Podocalyxin-10s YT.Tif]

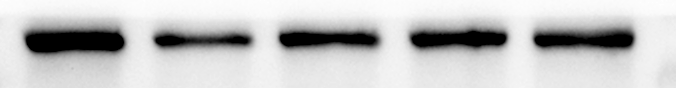

Supplement: Supplementary file 6 [file DataSheet2.zip › Fig.1/WBμ¥íσ╕a/Podocalyxin/PS-2-Podocalyxin-10s.tif]

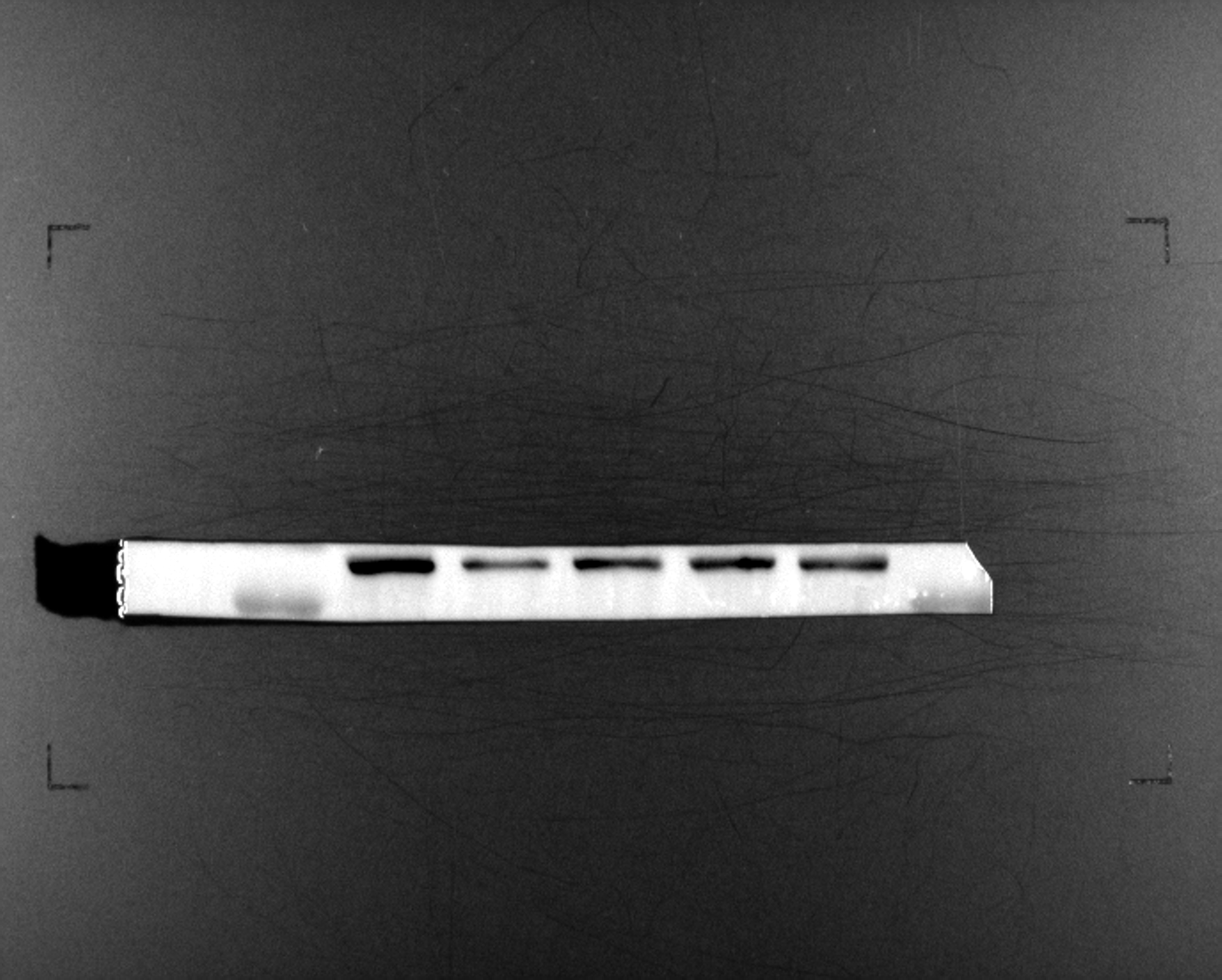

Supplement: Supplementary file 6 [file DataSheet2.zip › Fig.1/WBμ¥íσ╕a/Podocalyxin/2-Podocalyxin-10s YT.Tif]

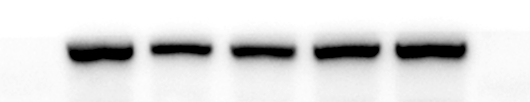

Supplement: Supplementary file 6 [file DataSheet2.zip › Fig.1/WBμ¥íσ╕a/Podocalyxin/τö¿-PS-σ╖a-1-Podocalyxin-10s.tif]

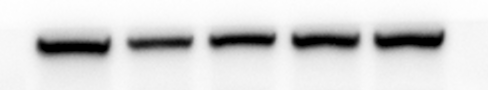

Supplement: Supplementary file 6 [file DataSheet2.zip › Fig.1/WBμ¥íσ╕a/Podocalyxin/PS-σÅ│-1-Podocalyxin-10s.tif]

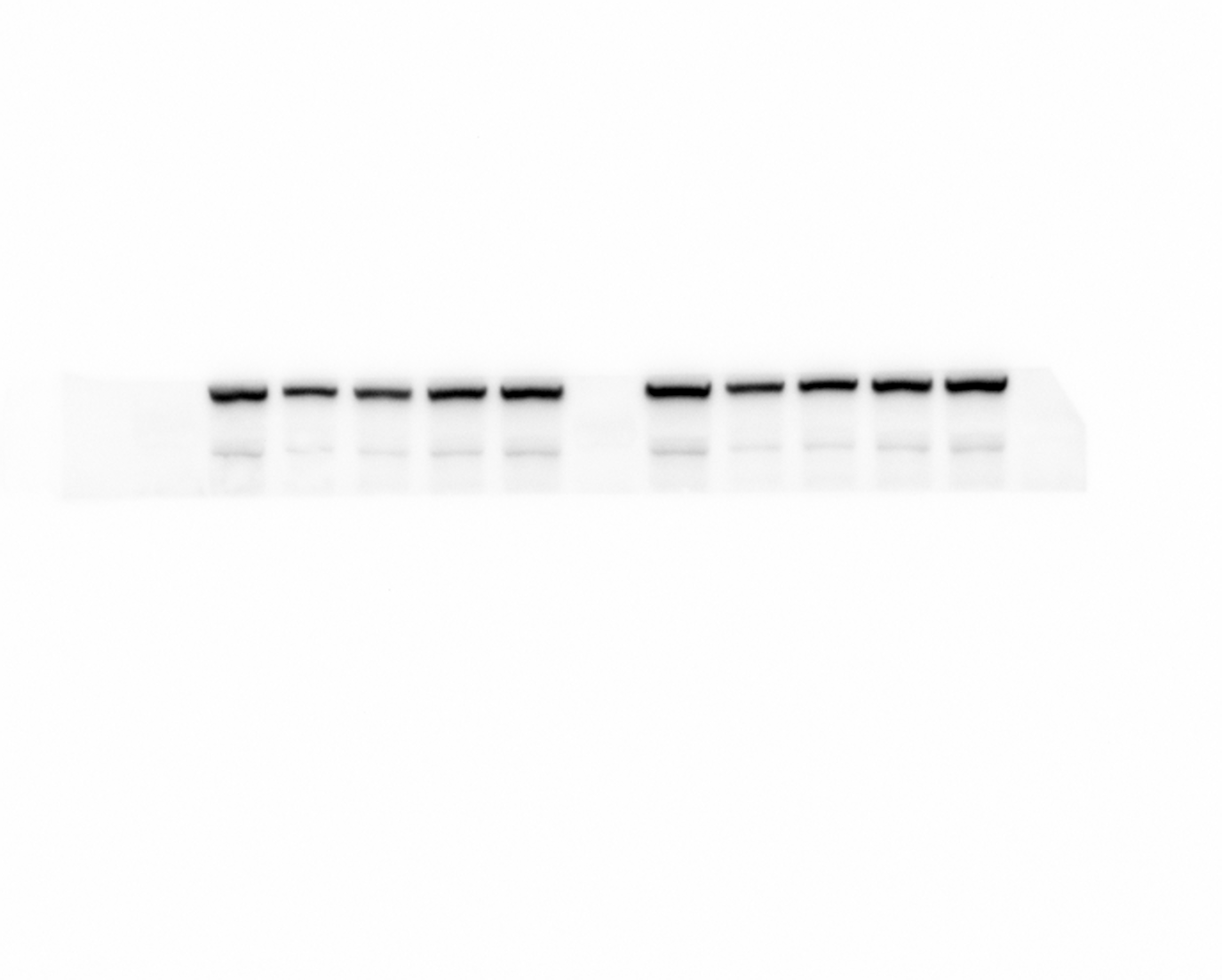

Supplement: Supplementary file 6 [file DataSheet2.zip › Fig.1/WBμ¥íσ╕a/Podocalyxin/1-Podocalyxin-10s.Tif]

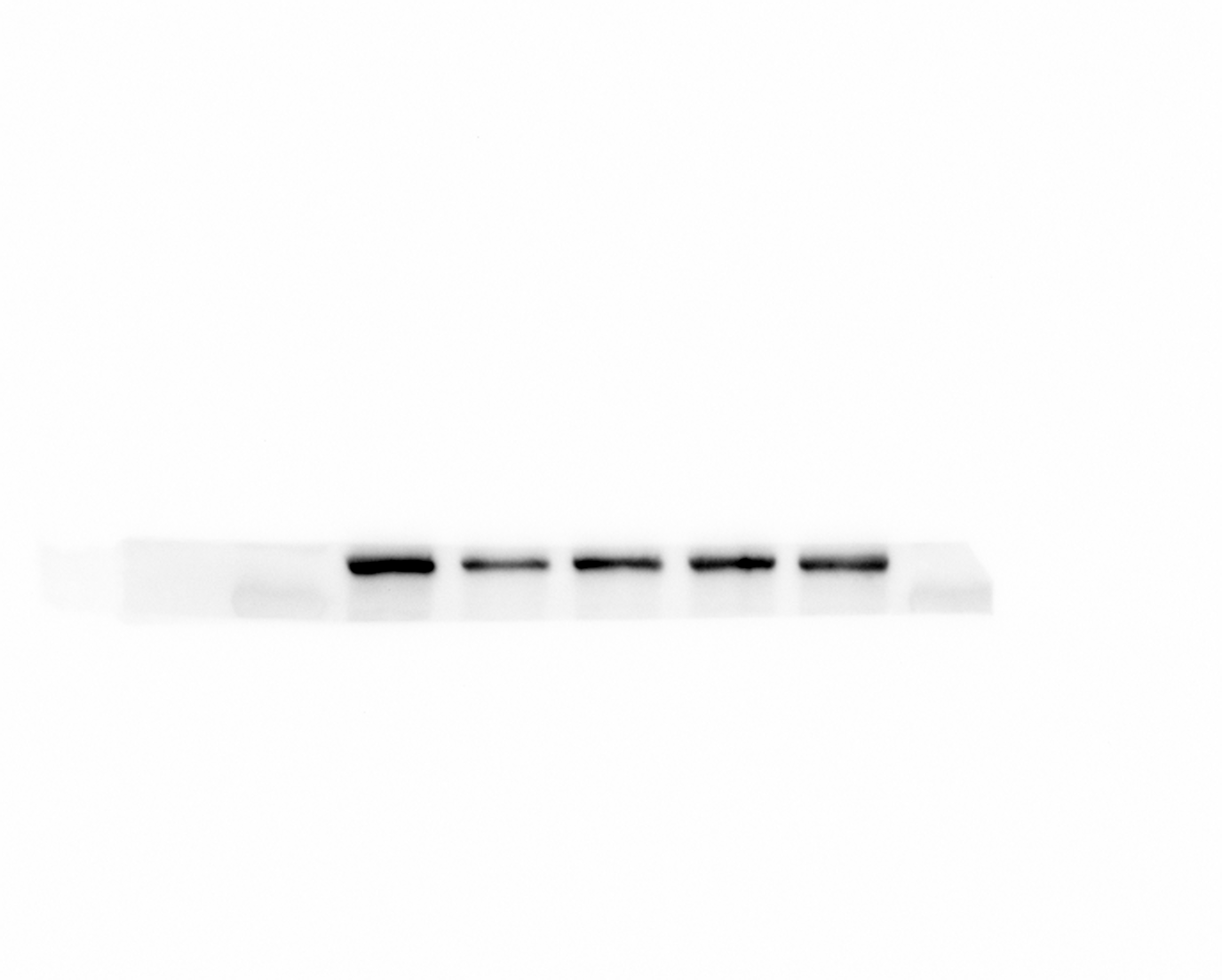

Supplement: Supplementary file 6 [file DataSheet2.zip › Fig.1/WBμ¥íσ╕a/Podocalyxin/2-Podocalyxin-10s.Tif]

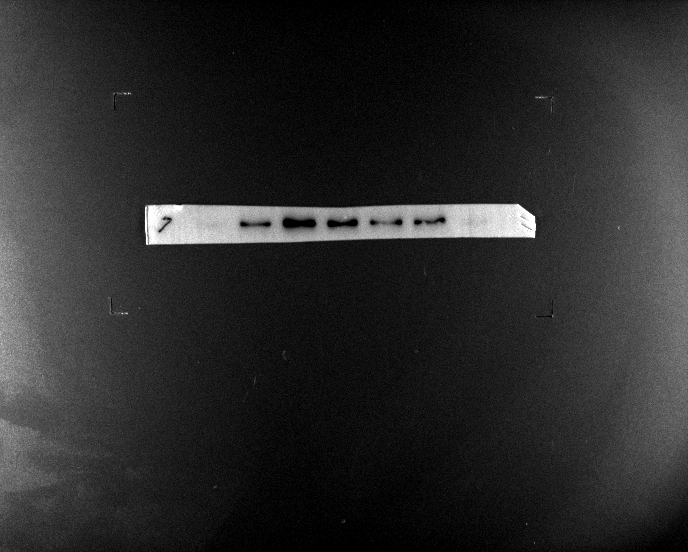

Supplement: Supplementary file 6 [file DataSheet2.zip › Fig.1/WBμ¥íσ╕a/IL-18/1-IL-18 YT.tif]

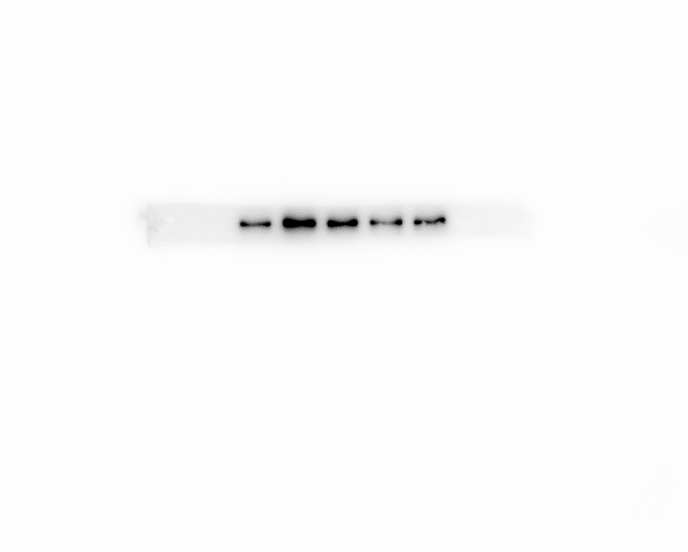

Supplement: Supplementary file 6 [file DataSheet2.zip › Fig.1/WBμ¥íσ╕a/IL-18/1-IL-18.tif]

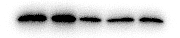

Supplement: Supplementary file 6 [file DataSheet2.zip › Fig.1/WBμ¥íσ╕a/IL-18/3.tif]

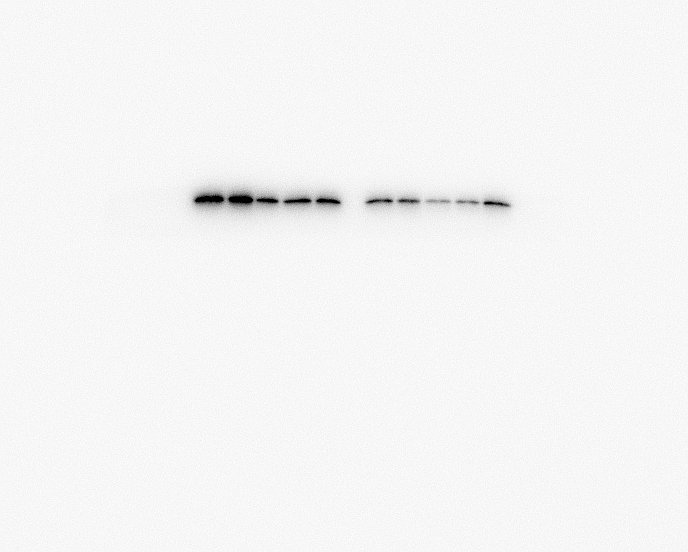

Supplement: Supplementary file 6 [file DataSheet2.zip › Fig.1/WBμ¥íσ╕a/IL-18/2-3-IL-18.tif]

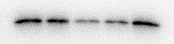

Supplement: Supplementary file 6 [file DataSheet2.zip › Fig.1/WBμ¥íσ╕a/IL-18/2.tif]

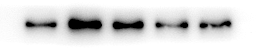

Supplement: Supplementary file 6 [file DataSheet2.zip › Fig.1/WBμ¥íσ╕a/IL-18/1.tif]

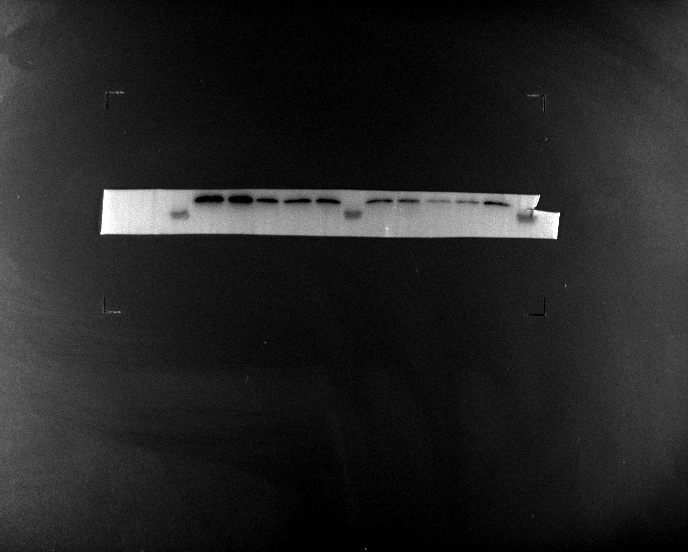

Supplement: Supplementary file 6 [file DataSheet2.zip › Fig.1/WBμ¥íσ╕a/IL-18/2-3-IL-18 YT.tif]

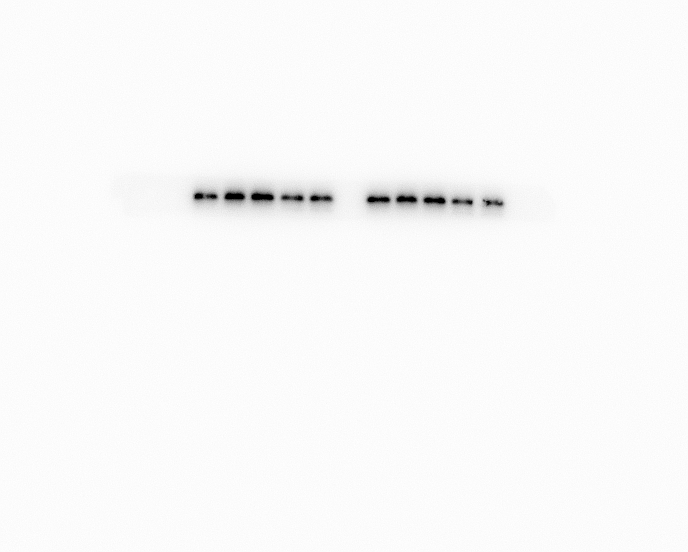

Supplement: Supplementary file 6 [file DataSheet2.zip › Fig.1/WBμ¥íσ╕a/GSDMD/3-GSDMD-N.tif]

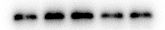

Supplement: Supplementary file 6 [file DataSheet2.zip › Fig.1/WBμ¥íσ╕a/GSDMD/3.tif]

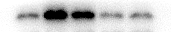

Supplement: Supplementary file 6 [file DataSheet2.zip › Fig.1/WBμ¥íσ╕a/GSDMD/2.tif]

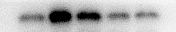

Supplement: Supplementary file 6 [file DataSheet2.zip › Fig.1/WBμ¥íσ╕a/GSDMD/1.tif]

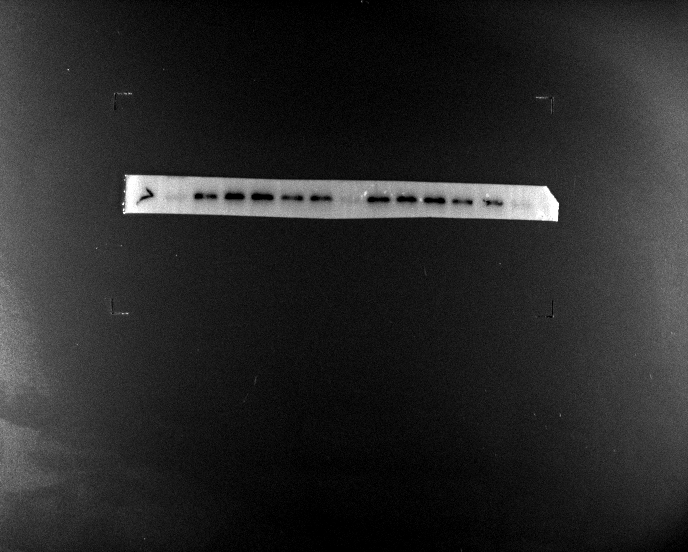

Supplement: Supplementary file 6 [file DataSheet2.zip › Fig.1/WBμ¥íσ╕a/GSDMD/3-GSDMD-N YT.tif]

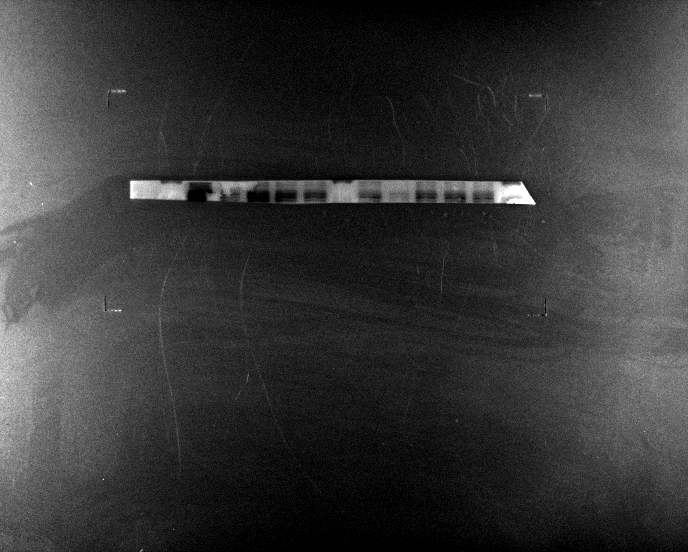

Supplement: Supplementary file 6 [file DataSheet2.zip › Fig.1/WBμ¥íσ╕a/ZO-1/3-4-ZO1 YT.tif]

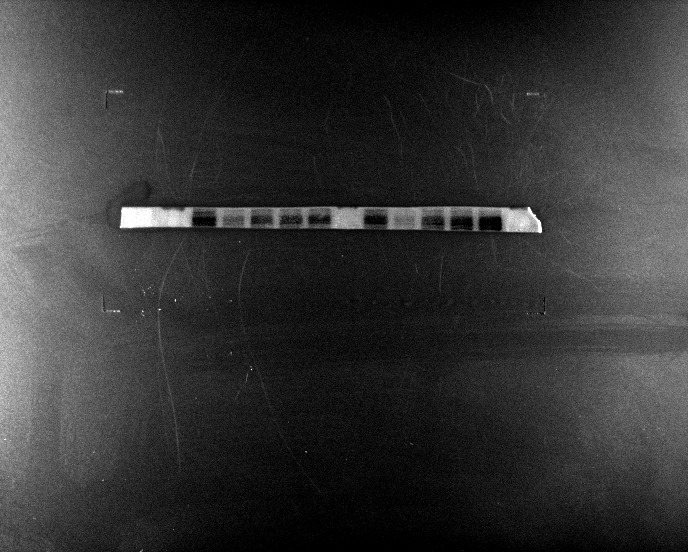

Supplement: Supplementary file 6 [file DataSheet2.zip › Fig.1/WBμ¥íσ╕a/ZO-1/1-2-ZO1 YT.tif]

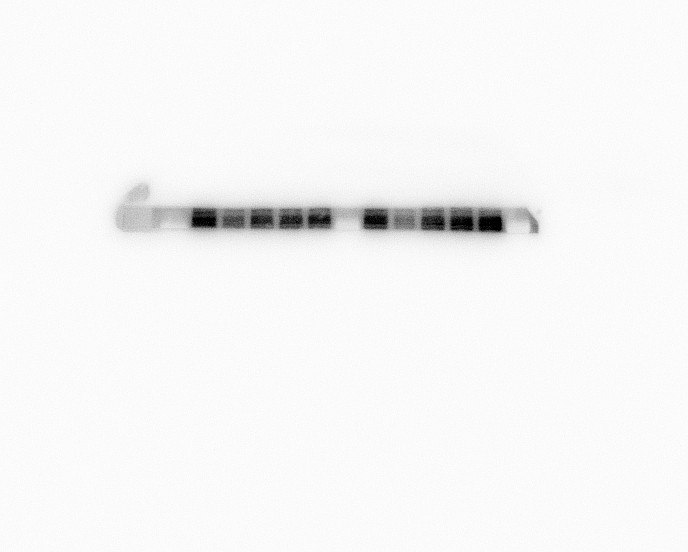

Supplement: Supplementary file 6 [file DataSheet2.zip › Fig.1/WBμ¥íσ╕a/ZO-1/1-2-ZO1.tif]

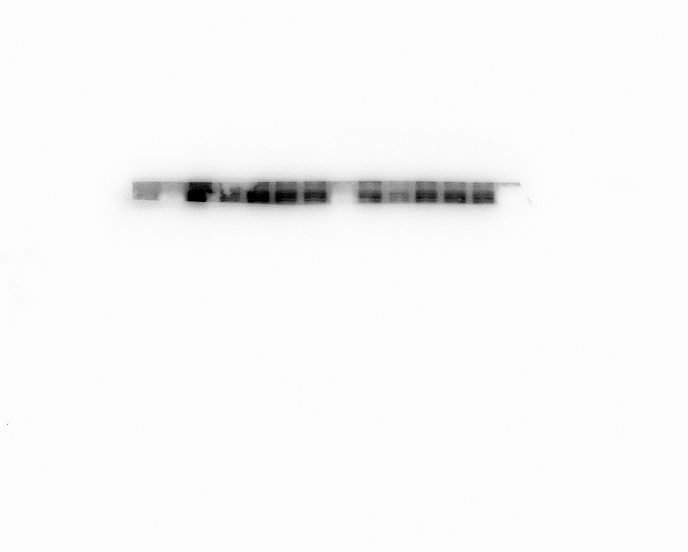

Supplement: Supplementary file 6 [file DataSheet2.zip › Fig.1/WBμ¥íσ╕a/ZO-1/3-4-ZO1.tif]

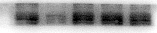

Supplement: Supplementary file 6 [file DataSheet2.zip › Fig.1/WBμ¥íσ╕a/ZO-1/3.tif]

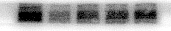

Supplement: Supplementary file 6 [file DataSheet2.zip › Fig.1/WBμ¥íσ╕a/ZO-1/2.tif]

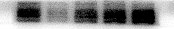

Supplement: Supplementary file 6 [file DataSheet2.zip › Fig.1/WBμ¥íσ╕a/ZO-1/1.tif]

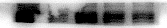

Supplement: Supplementary file 6 [file DataSheet2.zip › Fig.1/WBμ¥íσ╕a/ZO-1/4.tif]

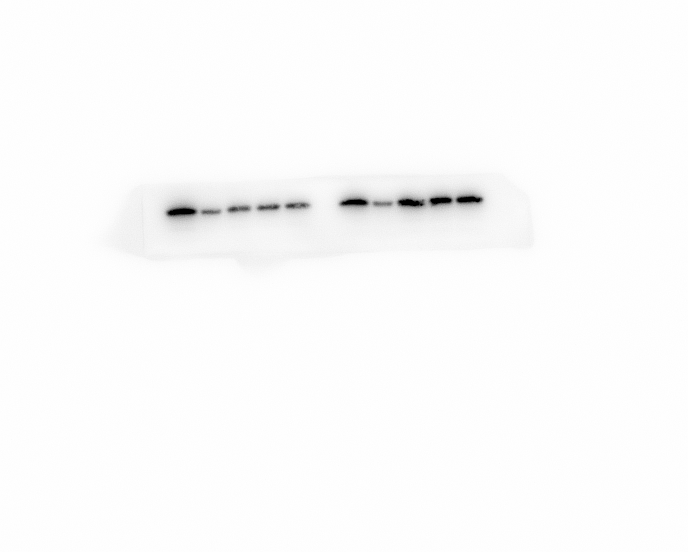

Supplement: Supplementary file 6 [file DataSheet2.zip › Fig.1/WBμ¥íσ╕a/nephrin/2-3-nephrin.tif]

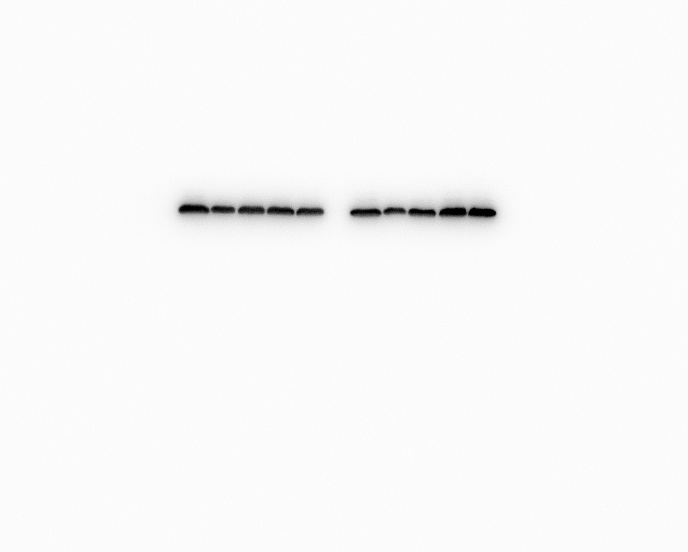

Supplement: Supplementary file 6 [file DataSheet2.zip › Fig.1/WBμ¥íσ╕a/nephrin/1-nephrin.tif]

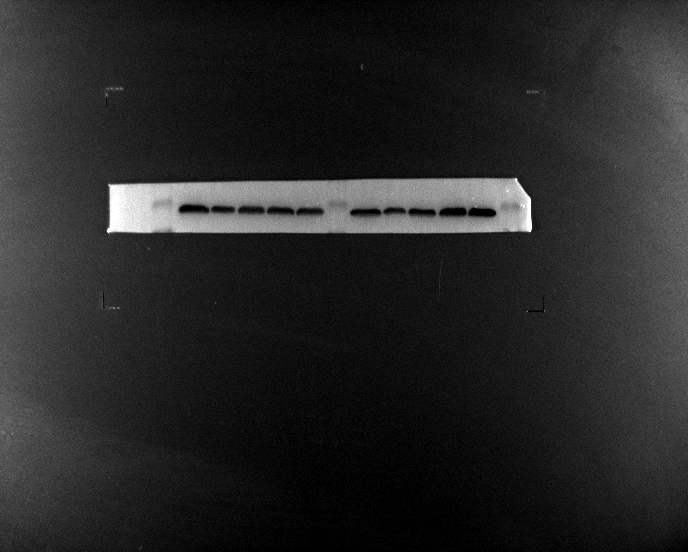

Supplement: Supplementary file 6 [file DataSheet2.zip › Fig.1/WBμ¥íσ╕a/nephrin/1-nephrin YT.tif]

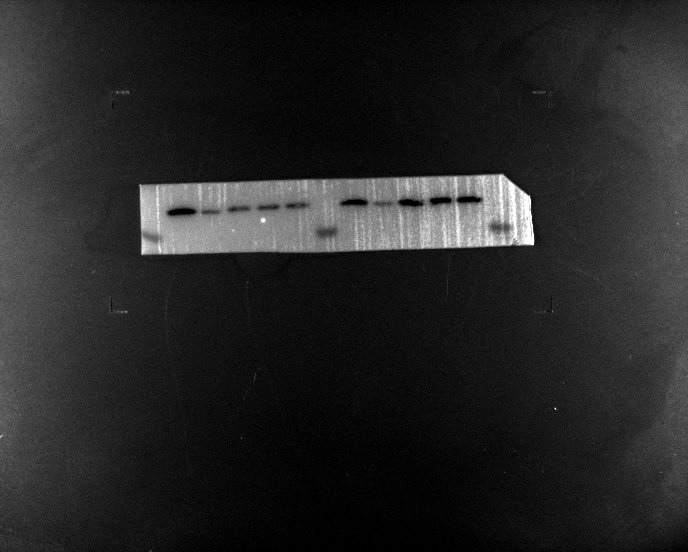

Supplement: Supplementary file 6 [file DataSheet2.zip › Fig.1/WBμ¥íσ╕a/nephrin/2-3-nephrin YT.tif]

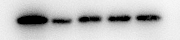

Supplement: Supplementary file 6 [file DataSheet2.zip › Fig.1/WBμ¥íσ╕a/nephrin/3.tif]

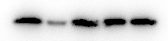

Supplement: Supplementary file 6 [file DataSheet2.zip › Fig.1/WBμ¥íσ╕a/nephrin/2.tif]

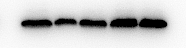

Supplement: Supplementary file 6 [file DataSheet2.zip › Fig.1/WBμ¥íσ╕a/nephrin/1.tif]

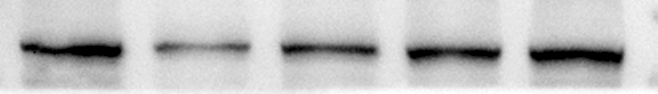

Supplement: Supplementary file 6 [file DataSheet2.zip › Fig.1/WBμ¥íσ╕a/WT1/PS 2-WT1-30s.tif]

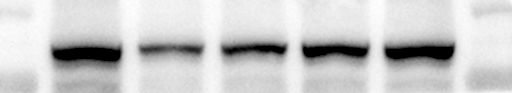

Supplement: Supplementary file 6 [file DataSheet2.zip › Fig.1/WBμ¥íσ╕a/WT1/τö¿-PS-σÅ│-1-WT1-30s.tif]

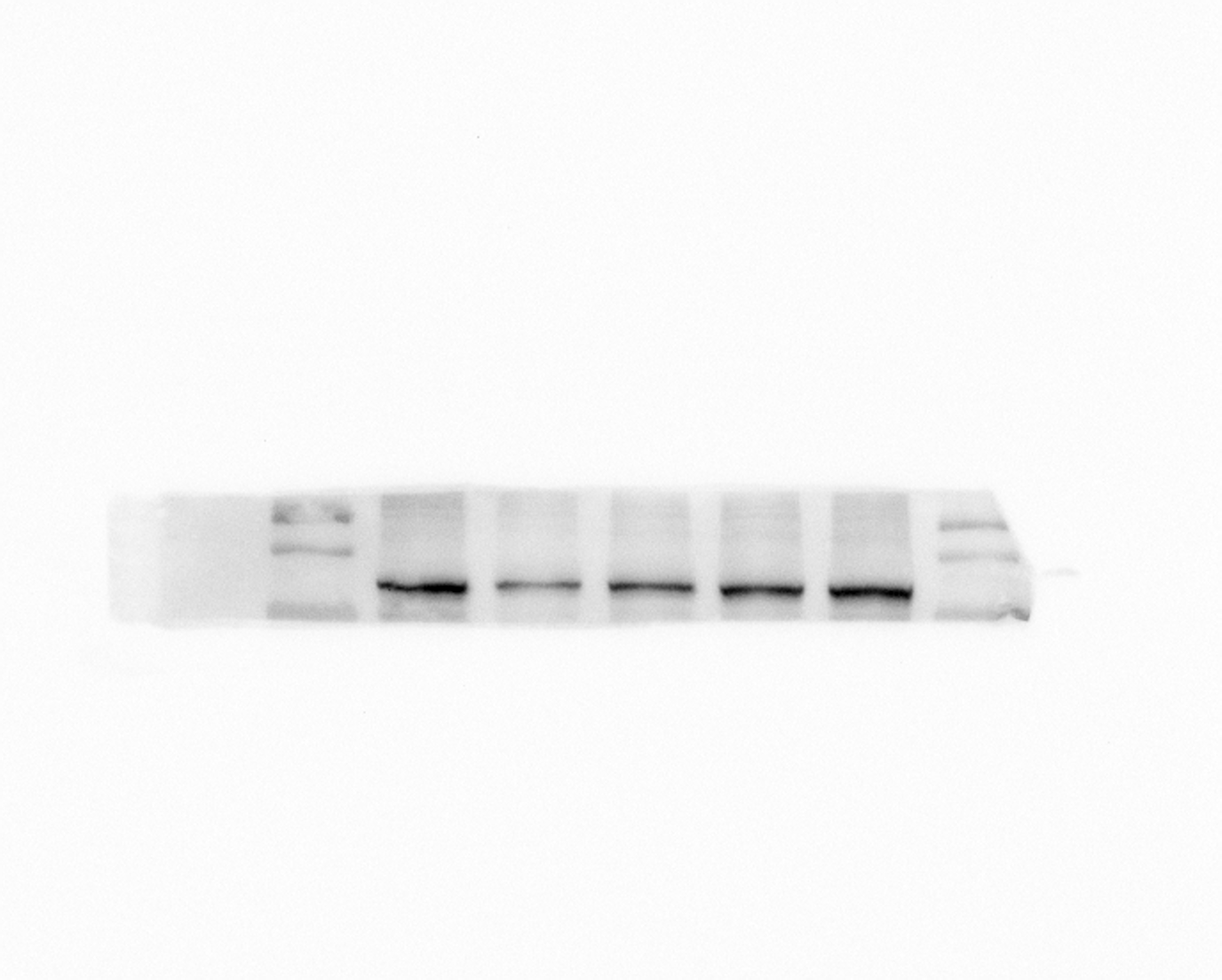

Supplement: Supplementary file 6 [file DataSheet2.zip › Fig.1/WBμ¥íσ╕a/WT1/2-WT1-30s.Tif]

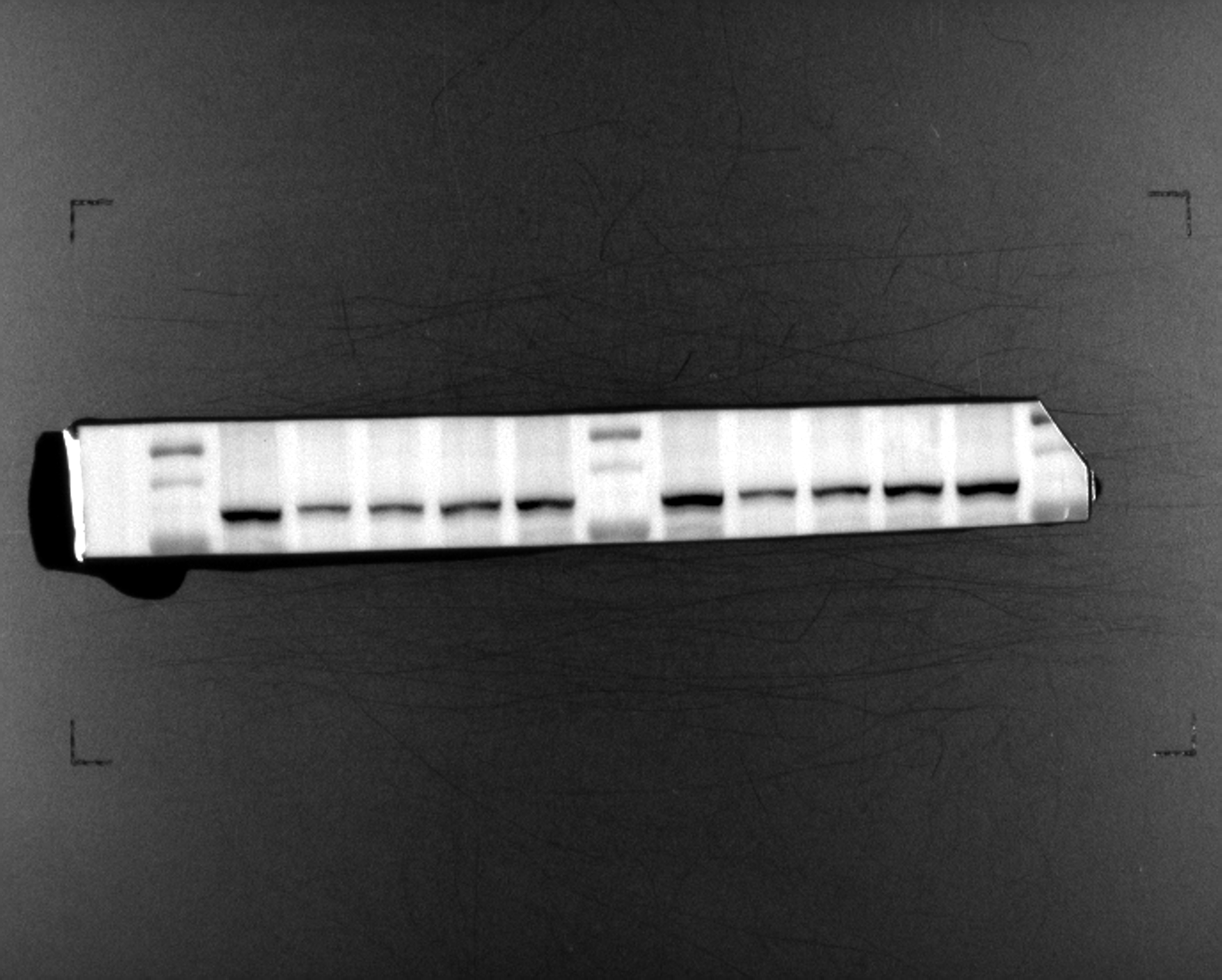

Supplement: Supplementary file 6 [file DataSheet2.zip › Fig.1/WBμ¥íσ╕a/WT1/1-WT1-30s YT.Tif]

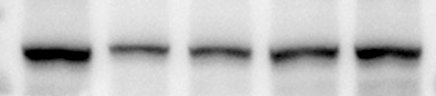

Supplement: Supplementary file 6 [file DataSheet2.zip › Fig.1/WBμ¥íσ╕a/WT1/PS-σ╖a-1-WT1-30s.tif]

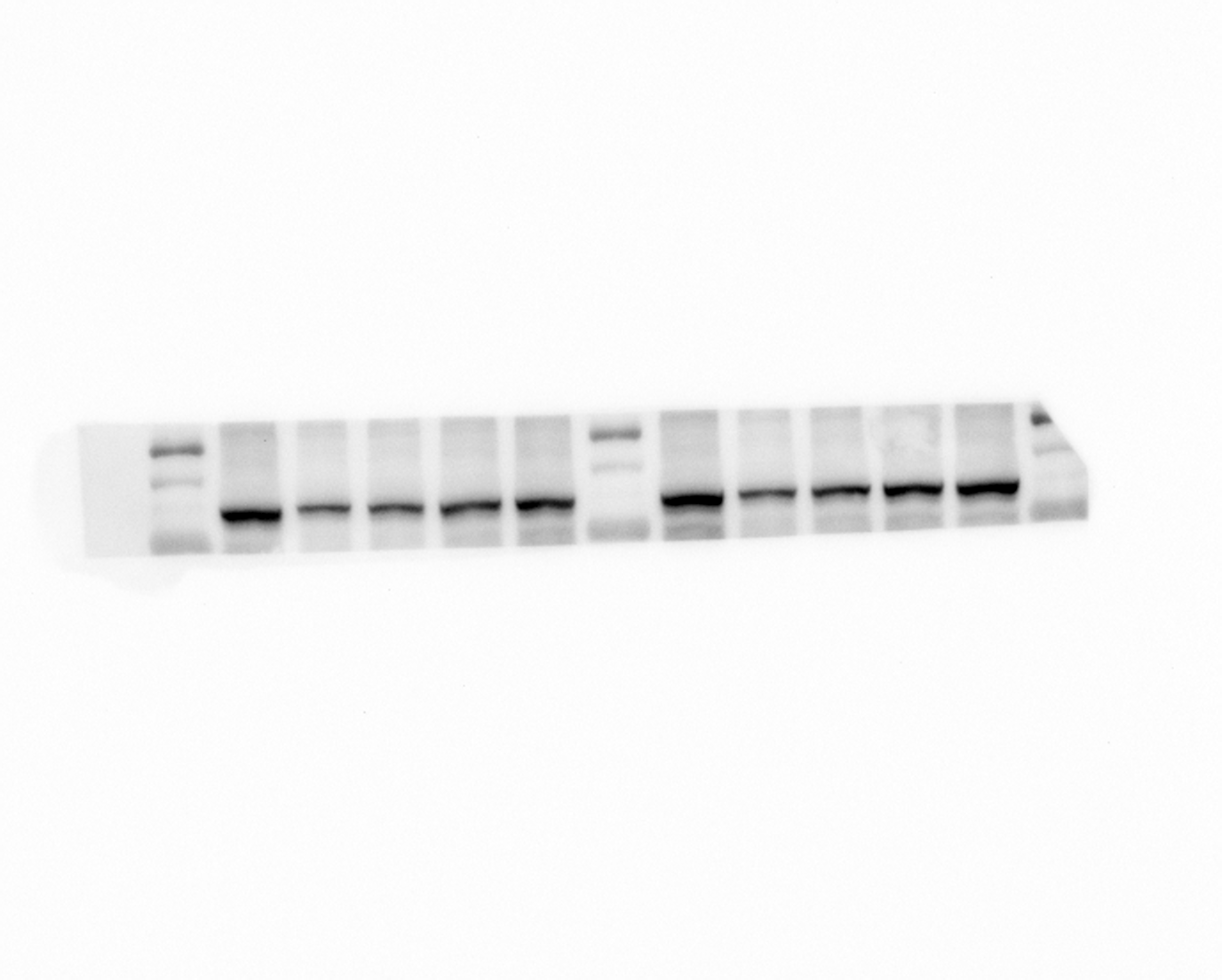

Supplement: Supplementary file 6 [file DataSheet2.zip › Fig.1/WBμ¥íσ╕a/WT1/1-WT1-30s.Tif]
