# Supplementary material for: Total Flavones of Abelmoschus manihot Ameliorates Podocyte Pyroptosis and Injury in High Glucose Conditions by Targeting METTL3-Dependent m6A Modification-Mediated NLRP3-Inflammasome Activation and PTEN/PI3K/Akt Signaling
Source: Front Pharmacol. 2021 Jul 15;12:667644. doi: 10.3389/fphar.2021.667644 (PMC8319635; doi:10.3389/fphar.2021.667644)
Supplement: Supplementary file 5 [file DataSheet1.pdf]

Supplementary Information

Materials and Methods

1. Quantitative Real-Time PCR

Total RNA was extracted using TRIzol (Invitrogen, Thermo Fisher Scientific, Waltham, MA, USA). Complementary DNA (cDNA) was synthesized and processed by quantitative real-time polymerase chain reaction (qRT-PCR) analysis using a QuantiNova SYBR Green PCR Kit (QIAGEN, Hilden, Germany). All experiments were performed according to the manufacturer's instructions. qRT-PCR analysis was performed using an ABI Step One Plus Real-Time PCR instrument (Applied Biosystems, Inc., Thermo Fisher Scientific, Foster City, CA, USA). The relative expression levels of PTEN mRNA were determined by the Ct ($2^{-\Delta\Delta Ct}$) method. GAPDH was used for normalization. The primer pair sequences were as follows:

Table 1 Mouse primers used in Quantitative Real-Time PCR

Name	Forward primer (5'->3')	Reverse primer (5'->3')
PTEN	TGGCGGAACTTGCAATCCTCAGT	TCCCGTCGTGTGGGTCCTGA
IL-1β	GAAATGCCACCTTTTGACAGTG	TGGATGCTCTCATCAGGACAG
IL-18	GACTCTTGCGTCAACTTCAAGG	CAGGCTGTCTTTTGTCAACGA
NLRP3	ATTACCCGCCCGAGAAAGG	TCGCAGCAAAGATCCACACAG
GSDMD	CCAGCATG GAAGCCTTAGAG	CAGAGTCGAGCACCAGACAC
Nephrin	CAGCGATGATGCGGAGTACG	CAGCTACCCAGGTAACTGTGC
WT1	GAGAGCCAGCCTACCATCC	GGGTCCTCGTGTTTGAAGGAA
GAPDH	AACAGCCTCAAGATCATCAGCA	ATGAGTCCTTCCACGATACCA

2. ELISA Assay

After the intervention of the podocytes, the cell protein was extracted with an ultrasonic cell crusher. Then, according to the manufacturer's instructions, the levels of GSDMD and NLRP3 were detected with ELISA kits (Abcam, USA), the levels of IL-1β and IL-18 were detected with ELISA kits (Elabscience, Wuhan, China), and the levels of nephrin and ZO-1 were detected with ELISA kits (Multisciences, Hangzhou, China).

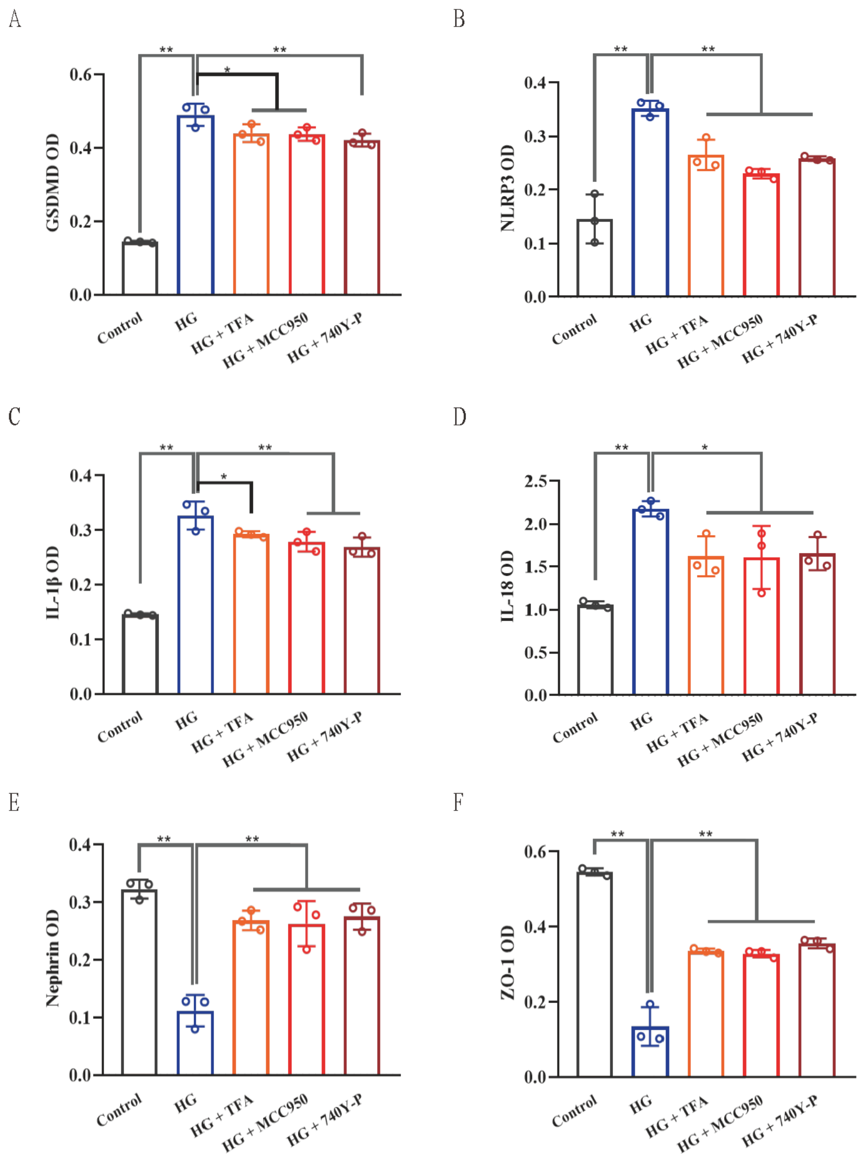

Figure S1. The levels of GSDMD, NLRP3, IL-1β, IL-18, nephrin and ZO-1 were detected by ELISA.

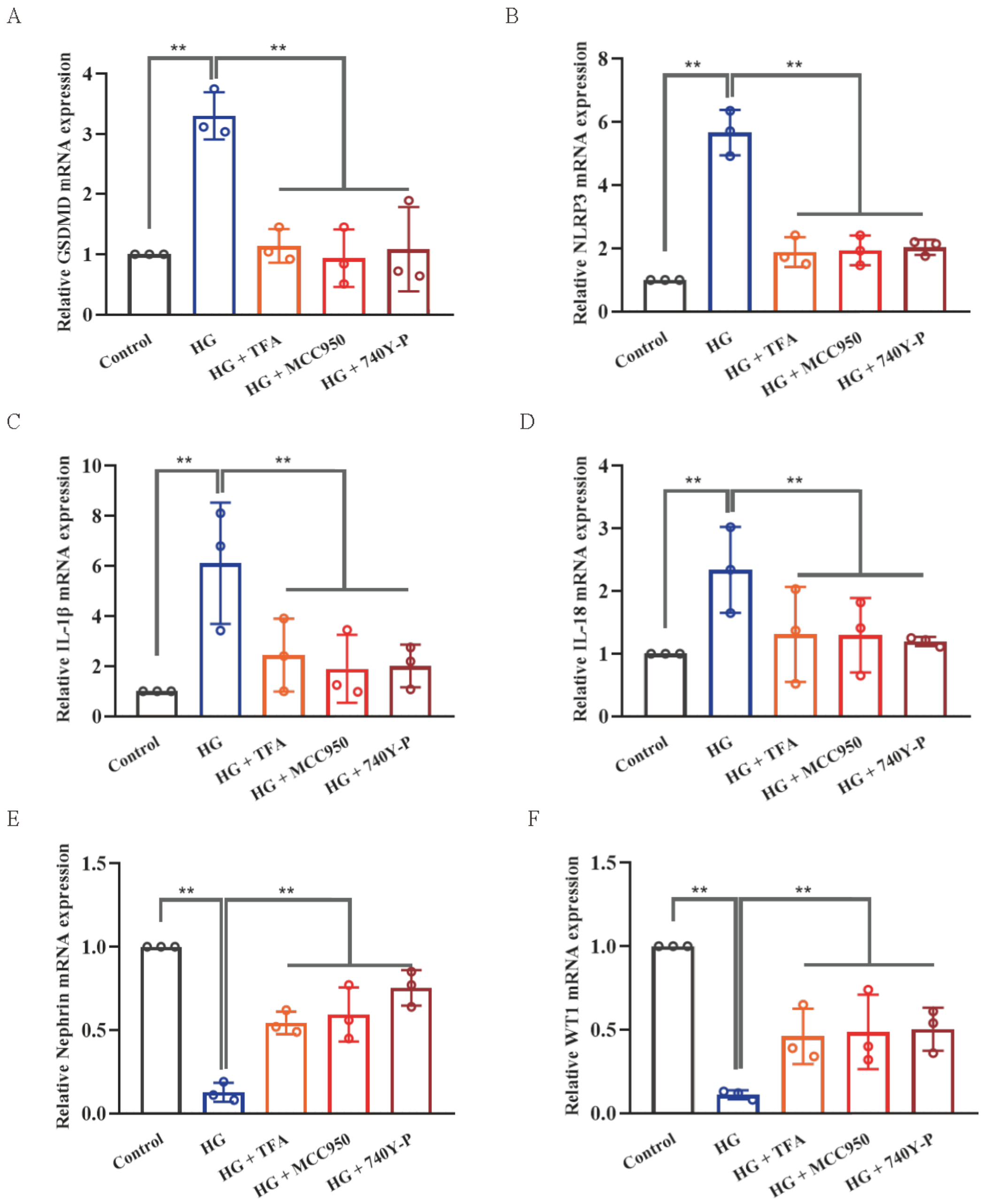

Figure S2. The mRNA levels of GSDMD, NLRP3, IL-1β, IL-18, nephrin and WT1 were detected by qRT-PCR.
