# Supplementary figures and images for: Total Flavones of Abelmoschus manihot Ameliorates Podocyte Pyroptosis and Injury in High Glucose Conditions by Targeting METTL3-Dependent m6A Modification-Mediated NLRP3-Inflammasome Activation and PTEN/PI3K/Akt Signaling (part 5 of 6)
Source: Front Pharmacol. 2021 Jul 15;12:667644. doi: 10.3389/fphar.2021.667644 (PMC8319635; doi:10.3389/fphar.2021.667644)

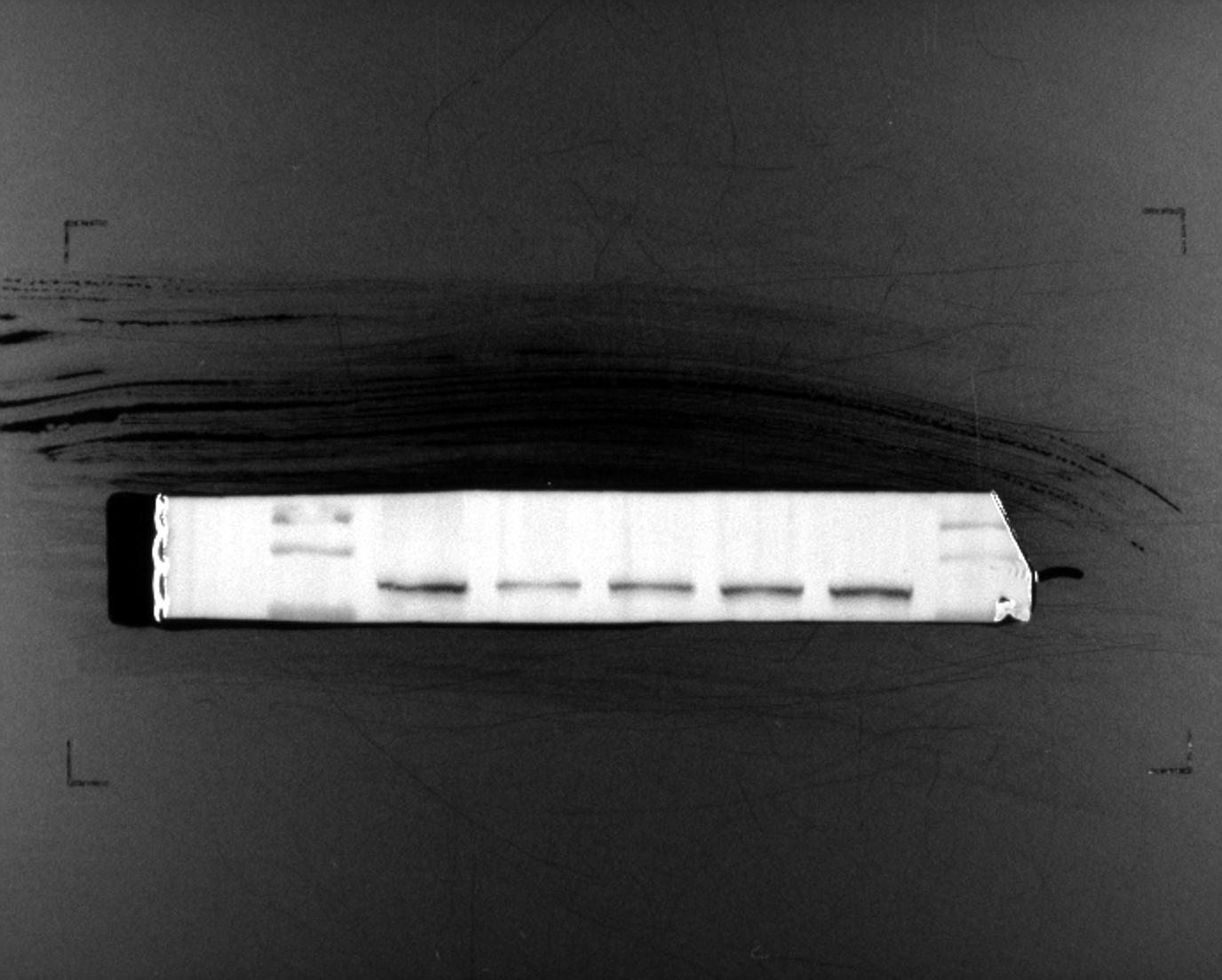

Supplement: Supplementary file 6 [file DataSheet2.zip › Fig.1/WBμ¥íσ╕a/WT1/2-WT1-30s YT.Tif]

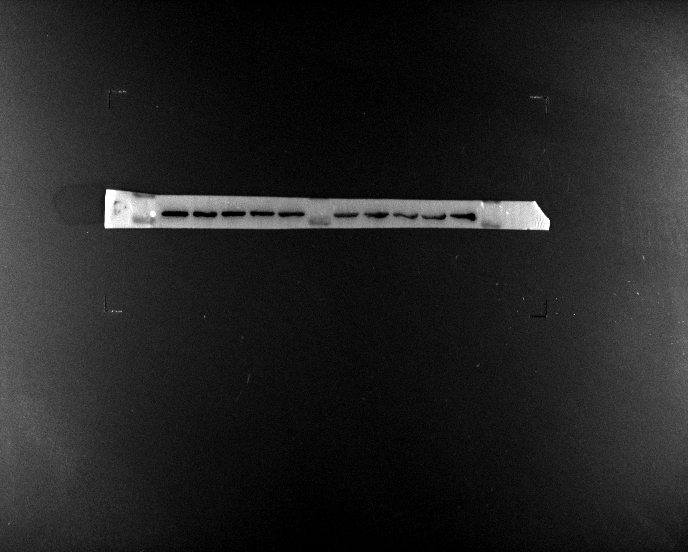

Supplement: Supplementary file 6 [file DataSheet2.zip › Fig.1/WBμ¥íσ╕a/GAPDH/1-2-GAPDH YT.tif]

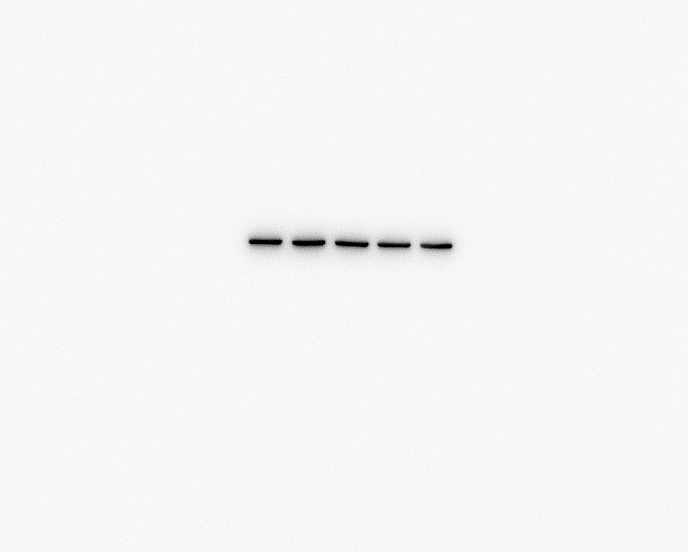

Supplement: Supplementary file 6 [file DataSheet2.zip › Fig.1/WBμ¥íσ╕a/GAPDH/3-GAPDH.tif]

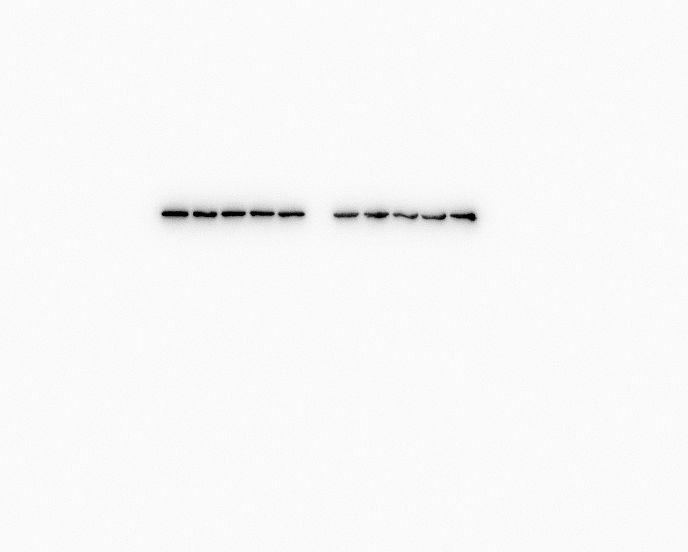

Supplement: Supplementary file 6 [file DataSheet2.zip › Fig.1/WBμ¥íσ╕a/GAPDH/1-2-GAPDH.tif]

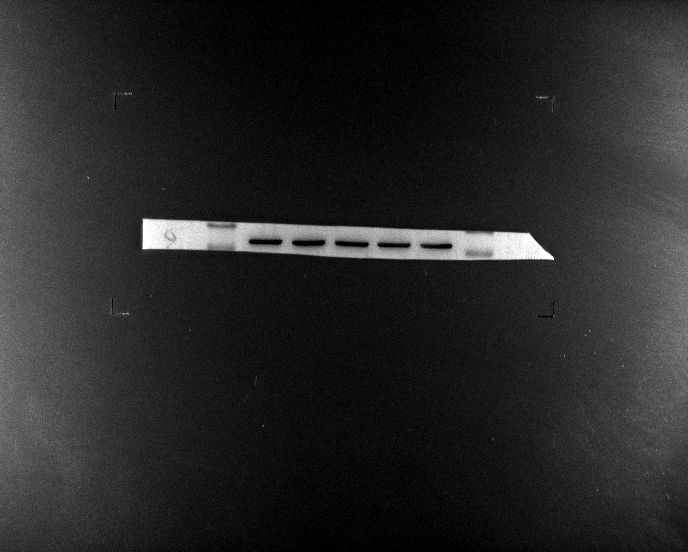

Supplement: Supplementary file 6 [file DataSheet2.zip › Fig.1/WBμ¥íσ╕a/GAPDH/3-GAPDH YT.tif]

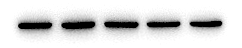

Supplement: Supplementary file 6 [file DataSheet2.zip › Fig.1/WBμ¥íσ╕a/GAPDH/3.tif]

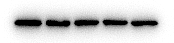

Supplement: Supplementary file 6 [file DataSheet2.zip › Fig.1/WBμ¥íσ╕a/GAPDH/2.tif]

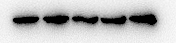

Supplement: Supplementary file 6 [file DataSheet2.zip › Fig.1/WBμ¥íσ╕a/GAPDH/1.tif]

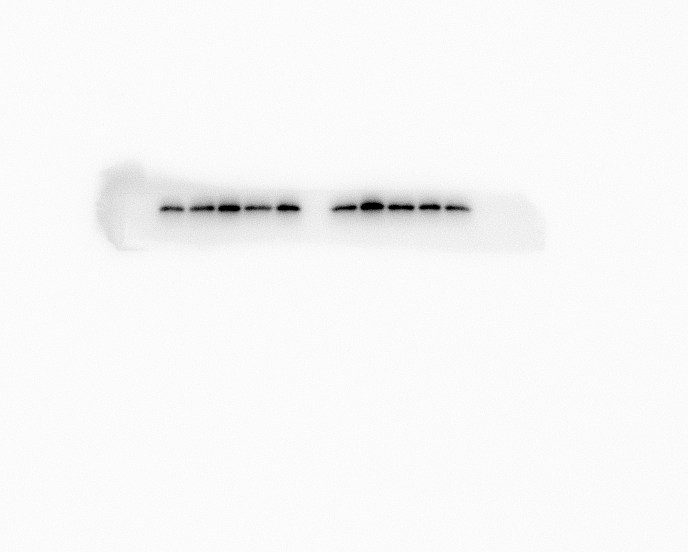

Supplement: Supplementary file 6 [file DataSheet2.zip › Fig.1/WBμ¥íσ╕a/IL-1/3-IL-1.tif]

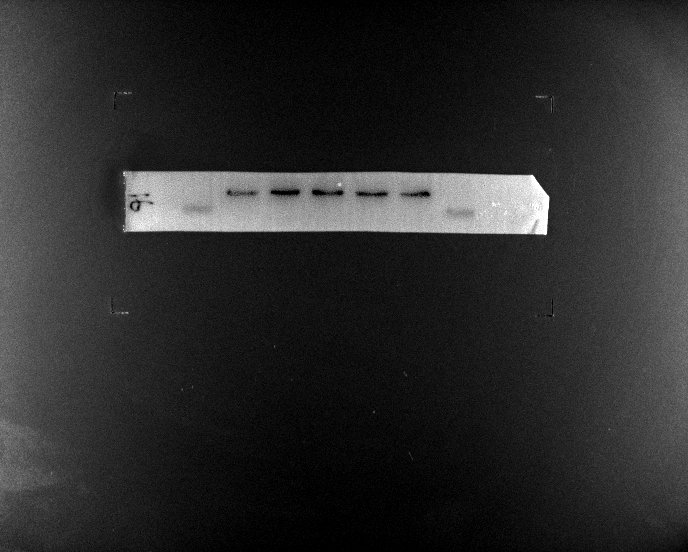

Supplement: Supplementary file 6 [file DataSheet2.zip › Fig.1/WBμ¥íσ╕a/IL-1/1-IL-1 YT.tif]

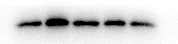

Supplement: Supplementary file 6 [file DataSheet2.zip › Fig.1/WBμ¥íσ╕a/IL-1/3.tif]

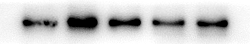

Supplement: Supplementary file 6 [file DataSheet2.zip › Fig.1/WBμ¥íσ╕a/IL-1/2.tif]

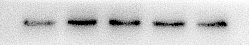

Supplement: Supplementary file 6 [file DataSheet2.zip › Fig.1/WBμ¥íσ╕a/IL-1/1.tif]

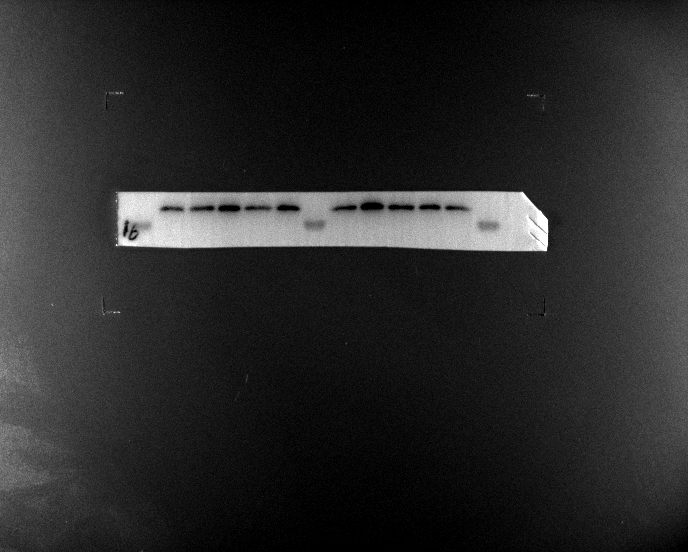

Supplement: Supplementary file 6 [file DataSheet2.zip › Fig.1/WBμ¥íσ╕a/IL-1/3-IL-1 YT.tif]

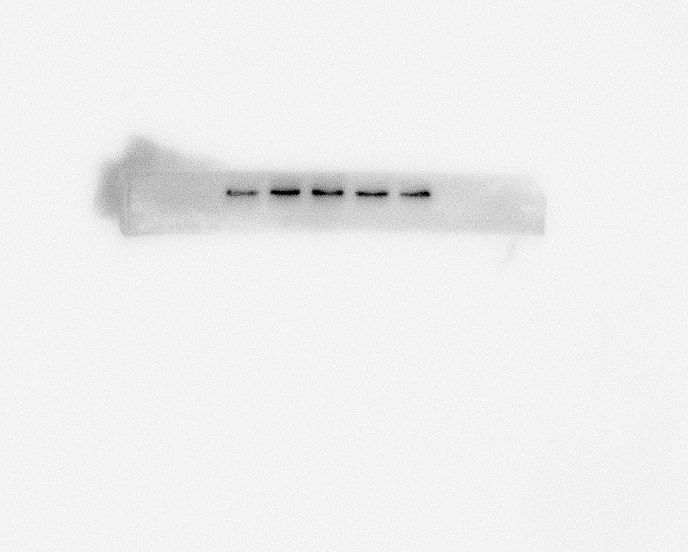

Supplement: Supplementary file 6 [file DataSheet2.zip › Fig.1/WBμ¥íσ╕a/IL-1/1-IL-1.tif]

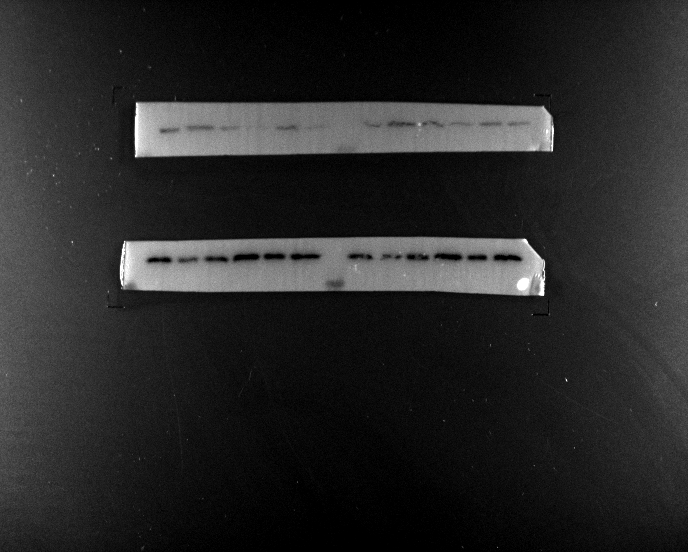

Supplement: Supplementary file 7 [file DataSheet5.zip › Fig.8/2. PTEN/1-PTEN YT.tif]

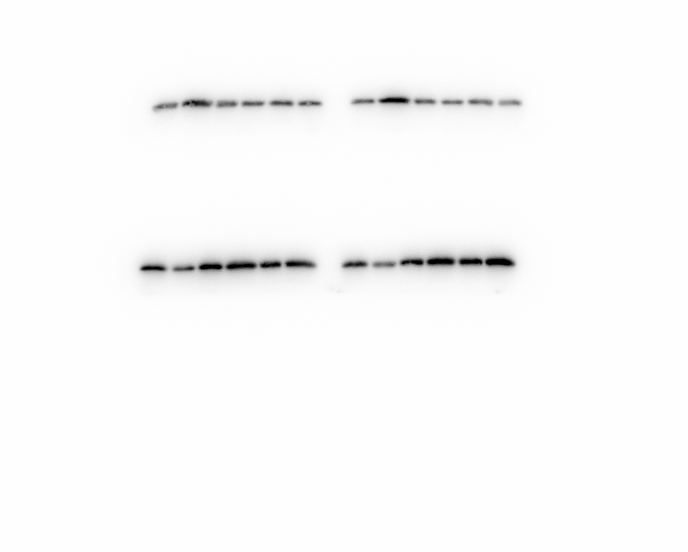

Supplement: Supplementary file 7 [file DataSheet5.zip › Fig.8/2. PTEN/2-3-PTEN.tif]

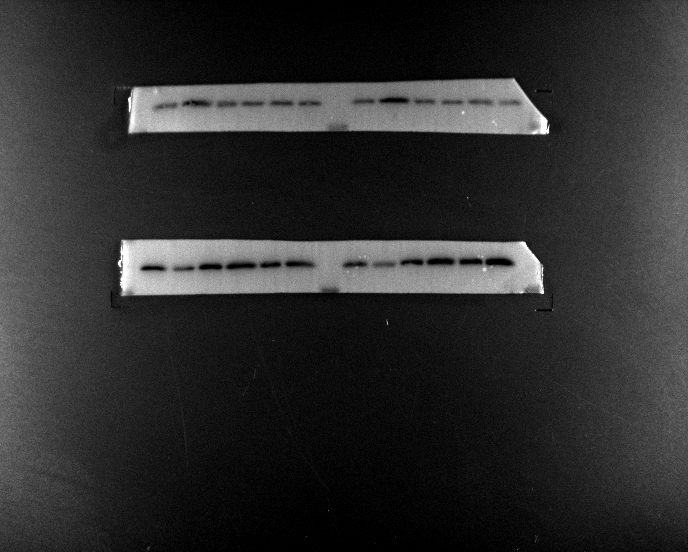

Supplement: Supplementary file 7 [file DataSheet5.zip › Fig.8/2. PTEN/2-3-PTEN YT.tif]

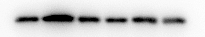

Supplement: Supplementary file 7 [file DataSheet5.zip › Fig.8/2. PTEN/3.tif]

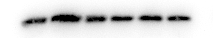

Supplement: Supplementary file 7 [file DataSheet5.zip › Fig.8/2. PTEN/2.tif]

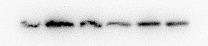

Supplement: Supplementary file 7 [file DataSheet5.zip › Fig.8/2. PTEN/1.tif]

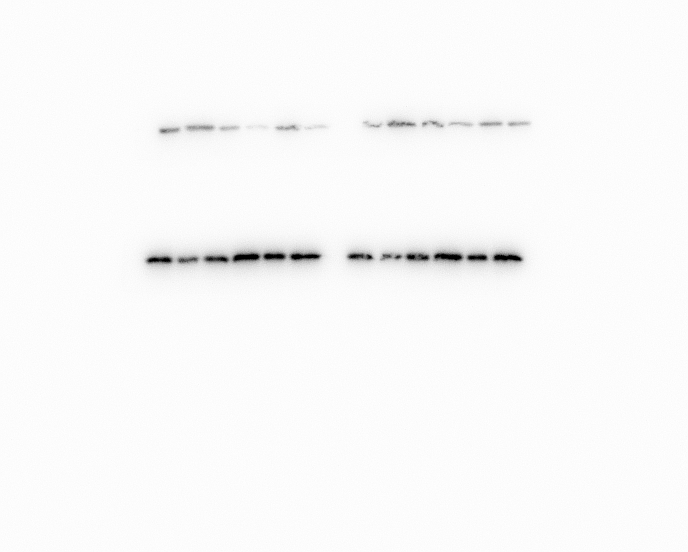

Supplement: Supplementary file 7 [file DataSheet5.zip › Fig.8/2. PTEN/1-PTEN.tif]

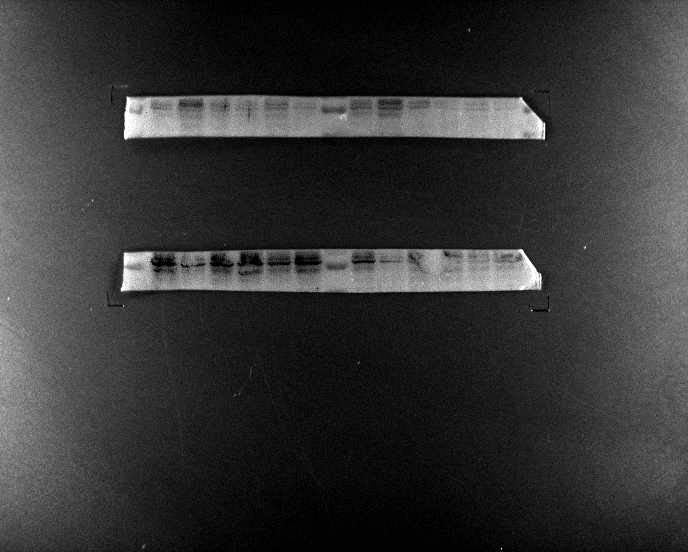

Supplement: Supplementary file 7 [file DataSheet5.zip › Fig.8/1. p-PI3K/2-p-PI3K YT.tif]

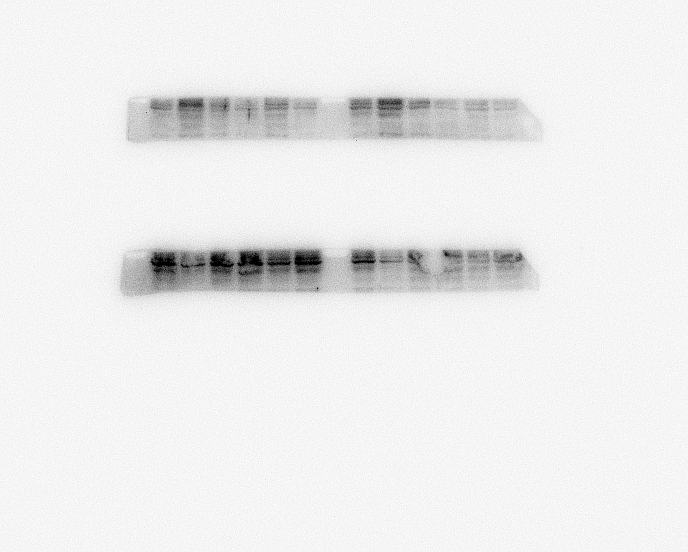

Supplement: Supplementary file 7 [file DataSheet5.zip › Fig.8/1. p-PI3K/2-p-PI3K.tif]

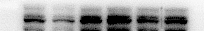

Supplement: Supplementary file 7 [file DataSheet5.zip › Fig.8/1. p-PI3K/3.tif]

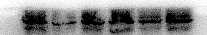

Supplement: Supplementary file 7 [file DataSheet5.zip › Fig.8/1. p-PI3K/2.tif]

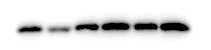

Supplement: Supplementary file 7 [file DataSheet5.zip › Fig.8/1. p-PI3K/1.tif]

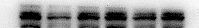

Supplement: Supplementary file 7 [file DataSheet5.zip › Fig.8/1. p-PI3K/4.tif]

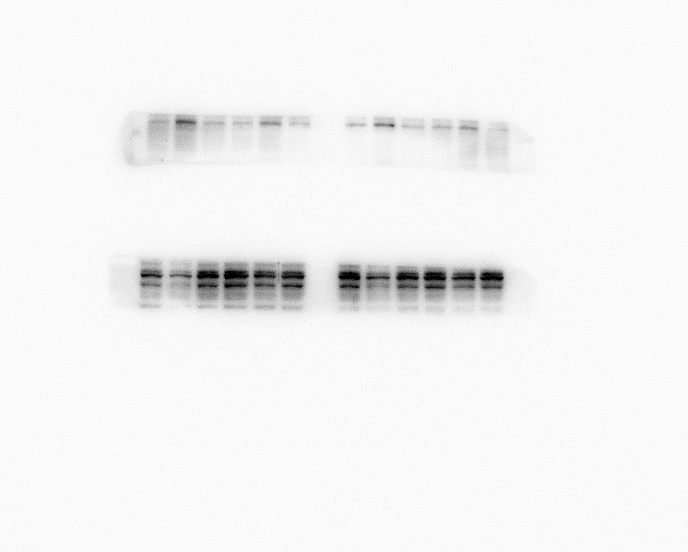

Supplement: Supplementary file 7 [file DataSheet5.zip › Fig.8/1. p-PI3K/3-4-p-PI3K.tif]

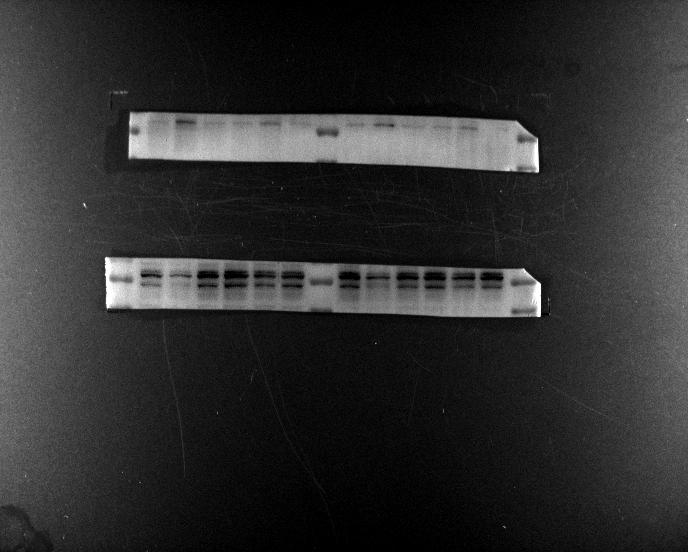

Supplement: Supplementary file 7 [file DataSheet5.zip › Fig.8/1. p-PI3K/3-4-p-PI3K YT.tif]

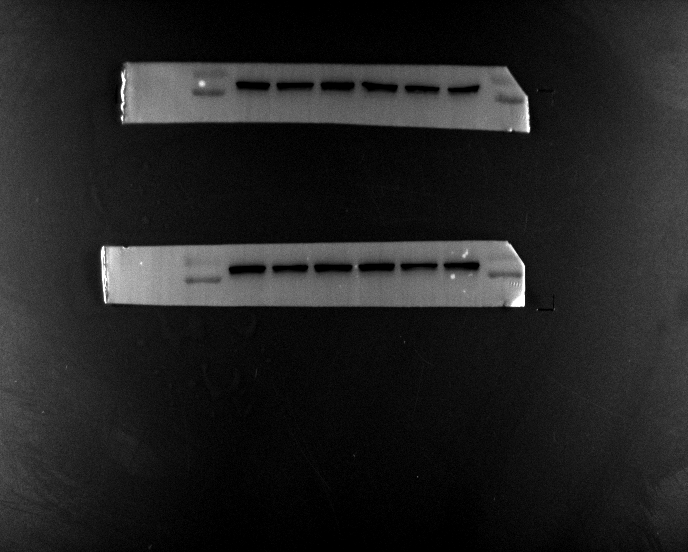

Supplement: Supplementary file 7 [file DataSheet5.zip › Fig.8/3. PI3K/1-2-PI3K YT.tif]

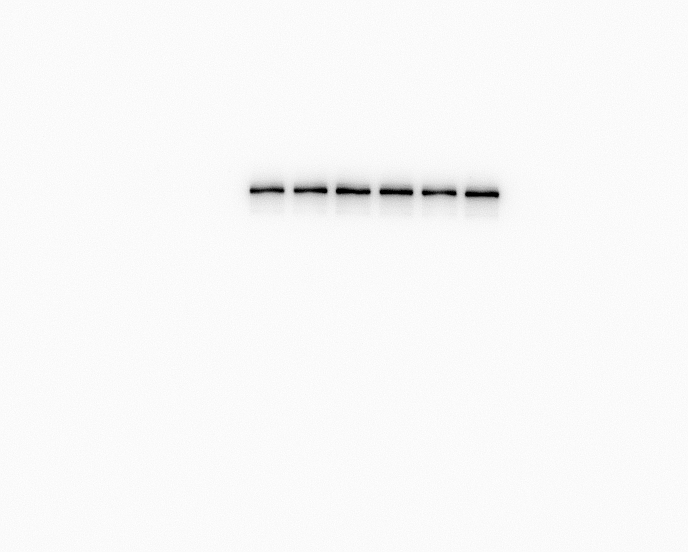

Supplement: Supplementary file 7 [file DataSheet5.zip › Fig.8/3. PI3K/3-PTEN.tif]

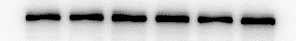

Supplement: Supplementary file 7 [file DataSheet5.zip › Fig.8/3. PI3K/3.tif]

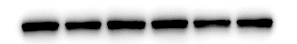

Supplement: Supplementary file 7 [file DataSheet5.zip › Fig.8/3. PI3K/2.tif]

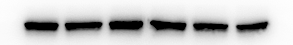

Supplement: Supplementary file 7 [file DataSheet5.zip › Fig.8/3. PI3K/1.tif]

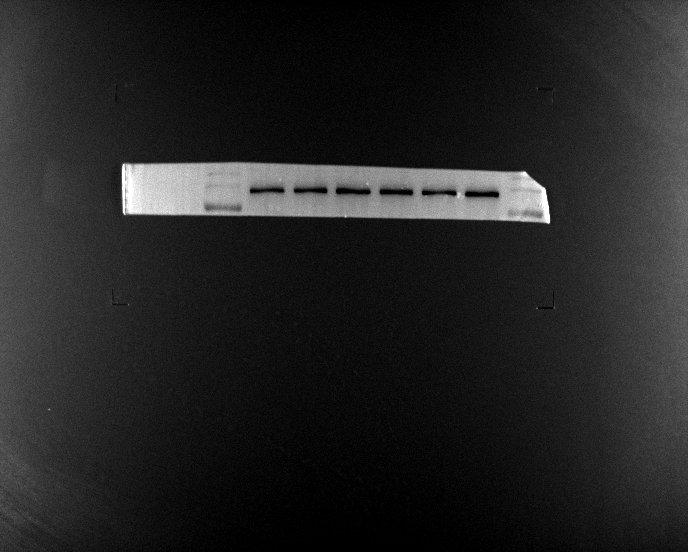

Supplement: Supplementary file 7 [file DataSheet5.zip › Fig.8/3. PI3K/3-PTEN YT.tif]

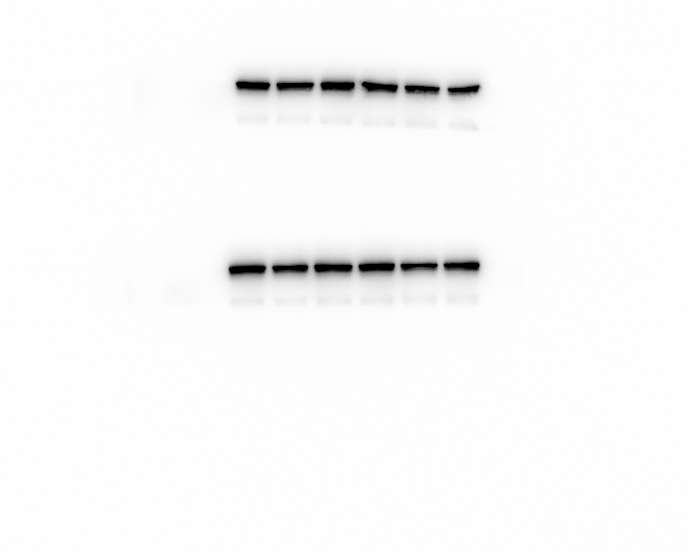

Supplement: Supplementary file 7 [file DataSheet5.zip › Fig.8/3. PI3K/1-2-PI3K.tif]

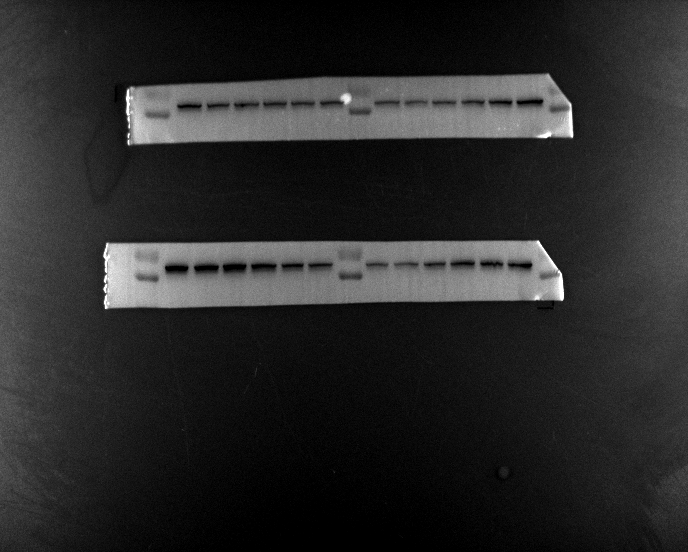

Supplement: Supplementary file 7 [file DataSheet5.zip › Fig.8/5. AKT/1-AKT YT.tif]

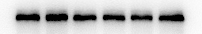

Supplement: Supplementary file 7 [file DataSheet5.zip › Fig.8/5. AKT/3.tif]

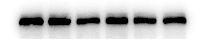

Supplement: Supplementary file 7 [file DataSheet5.zip › Fig.8/5. AKT/2.tif]

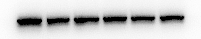

Supplement: Supplementary file 7 [file DataSheet5.zip › Fig.8/5. AKT/1.tif]

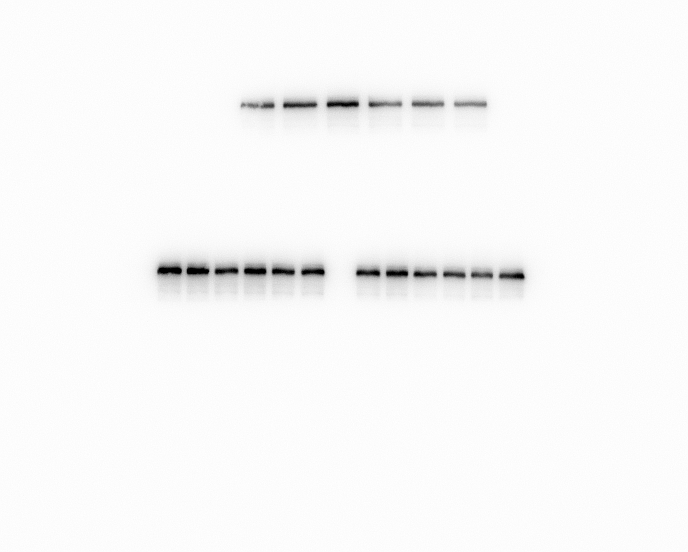

Supplement: Supplementary file 7 [file DataSheet5.zip › Fig.8/5. AKT/2-AKT.tif]

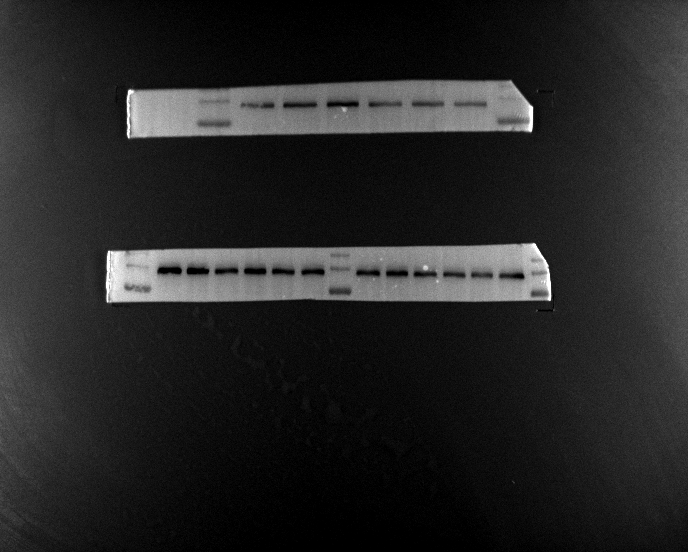

Supplement: Supplementary file 7 [file DataSheet5.zip › Fig.8/5. AKT/2-AKT YT.tif]

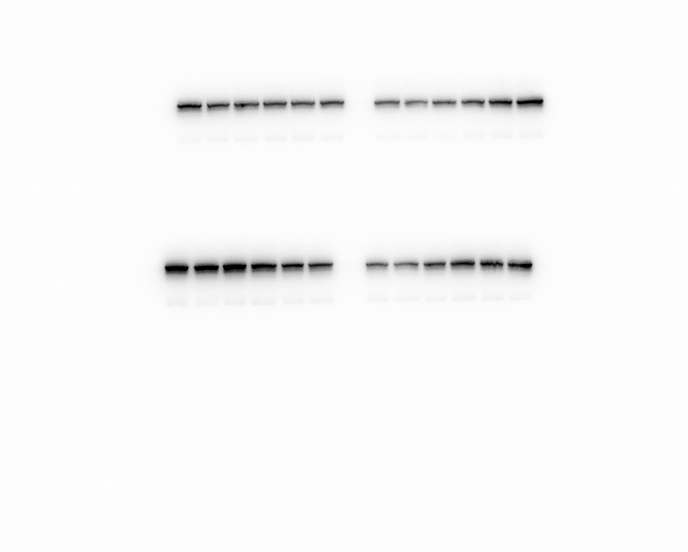

Supplement: Supplementary file 7 [file DataSheet5.zip › Fig.8/5. AKT/1-AKT.tif]

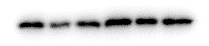

Supplement: Supplementary file 7 [file DataSheet5.zip › Fig.8/4. P-AKT/3.tif]

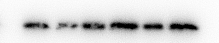

Supplement: Supplementary file 7 [file DataSheet5.zip › Fig.8/4. P-AKT/2.tif]

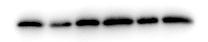

Supplement: Supplementary file 7 [file DataSheet5.zip › Fig.8/4. P-AKT/1.tif]

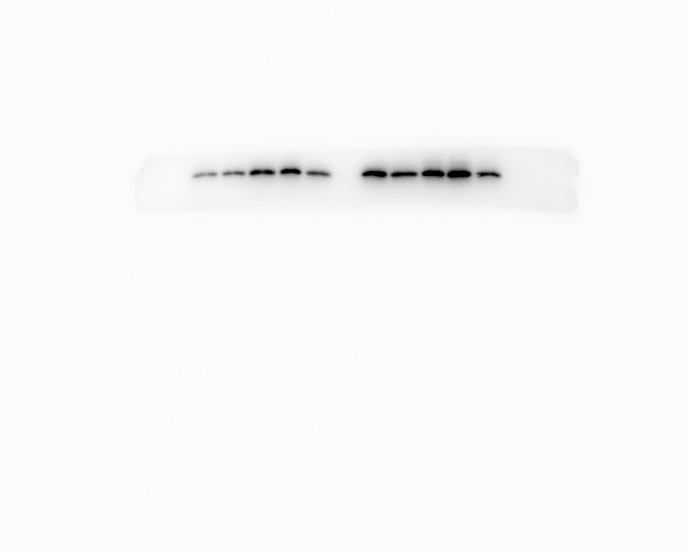

Supplement: Supplementary file 7 [file DataSheet5.zip › Fig.8/OE METTL3/METTL3/1-METTL3.tif]

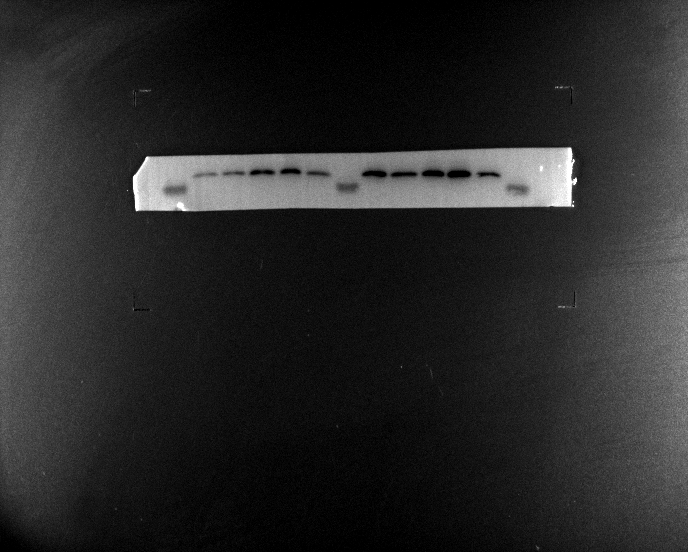

Supplement: Supplementary file 7 [file DataSheet5.zip › Fig.8/OE METTL3/METTL3/1-METTL3 YT.tif]

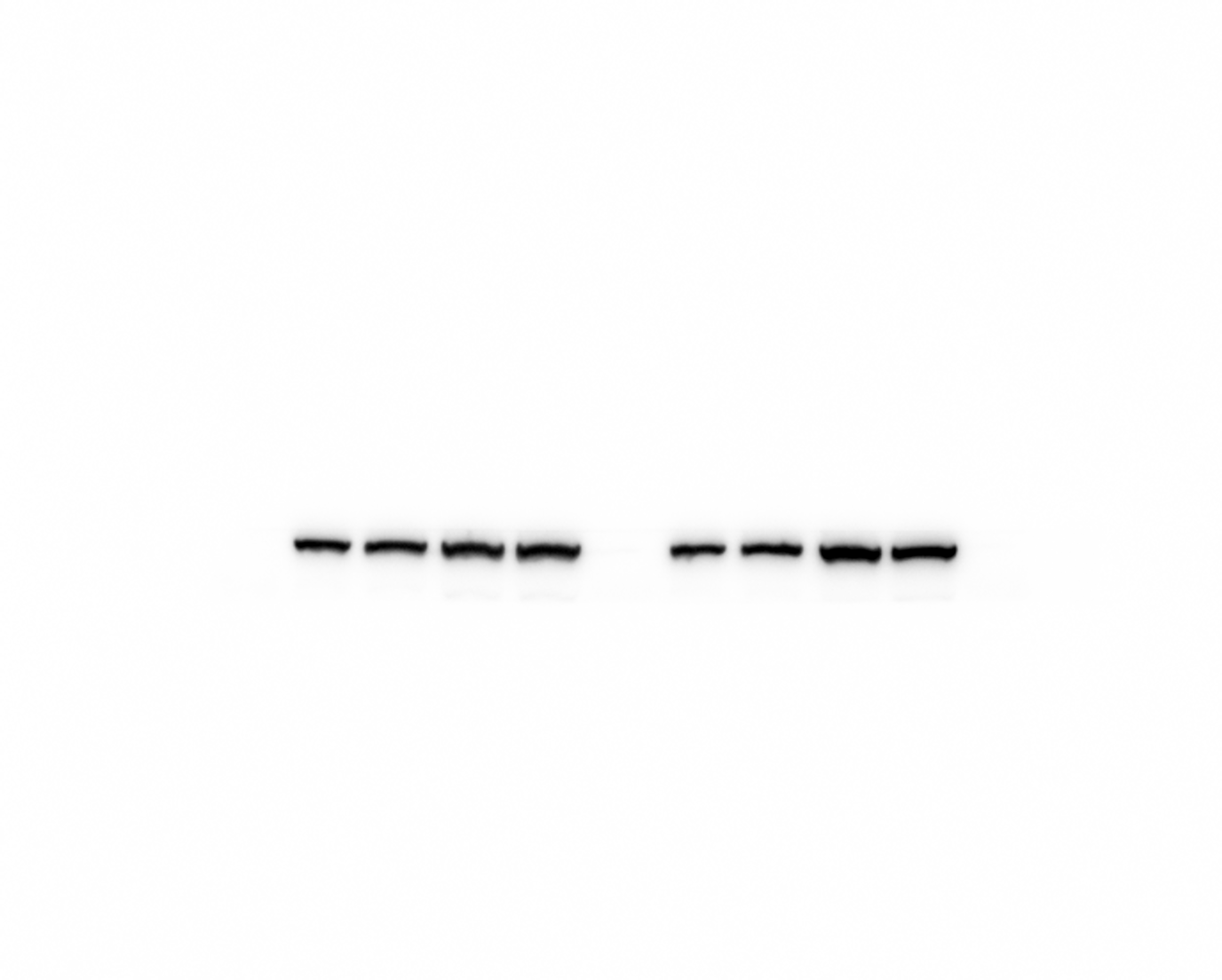

Supplement: Supplementary file 7 [file DataSheet5.zip › Fig.8/OE METTL3/METTL3/4-METTL3.tif]

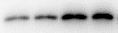

Supplement: Supplementary file 7 [file DataSheet5.zip › Fig.8/OE METTL3/METTL3/1.tif]

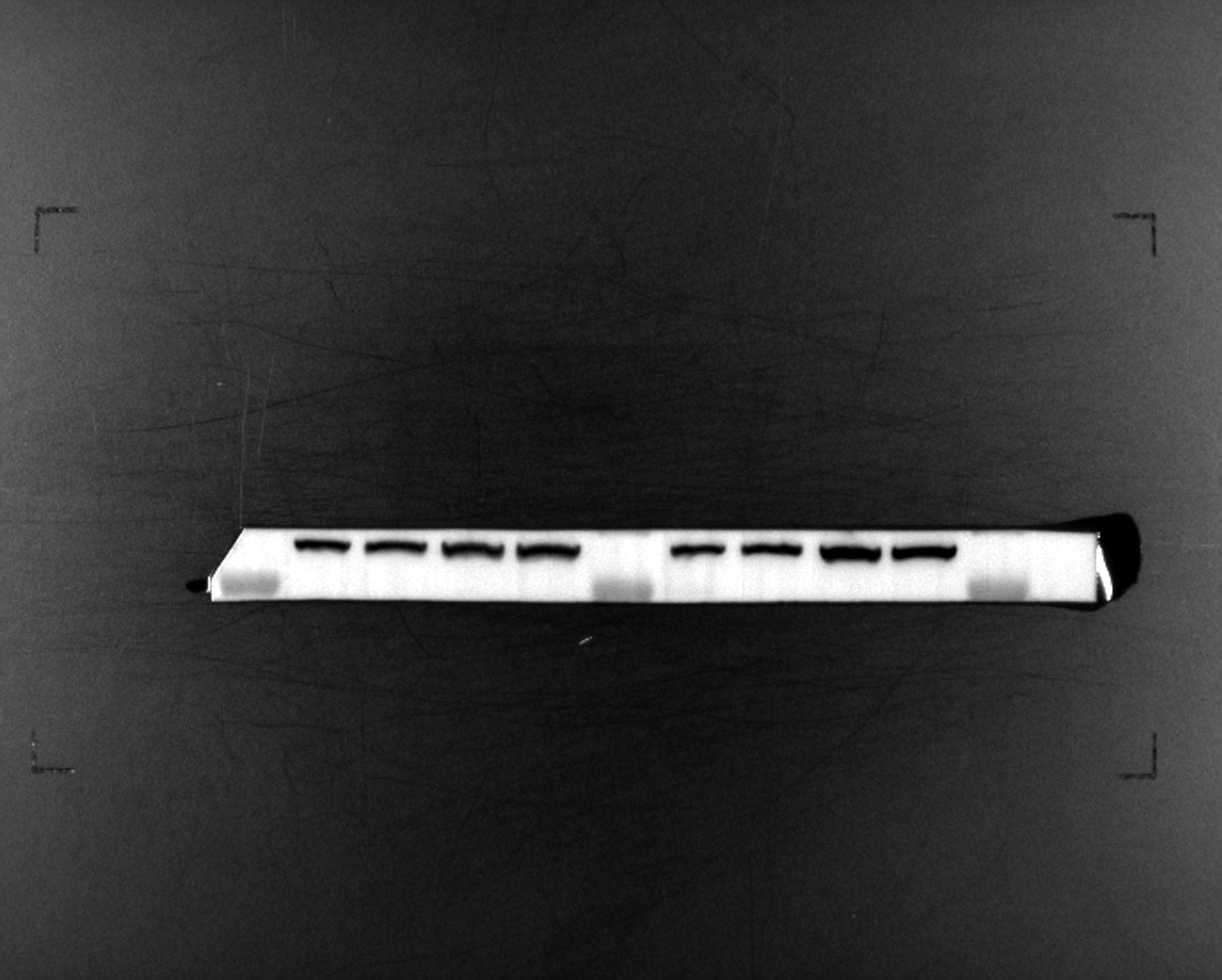

Supplement: Supplementary file 7 [file DataSheet5.zip › Fig.8/OE METTL3/METTL3/4-METTL3 YT.tif]

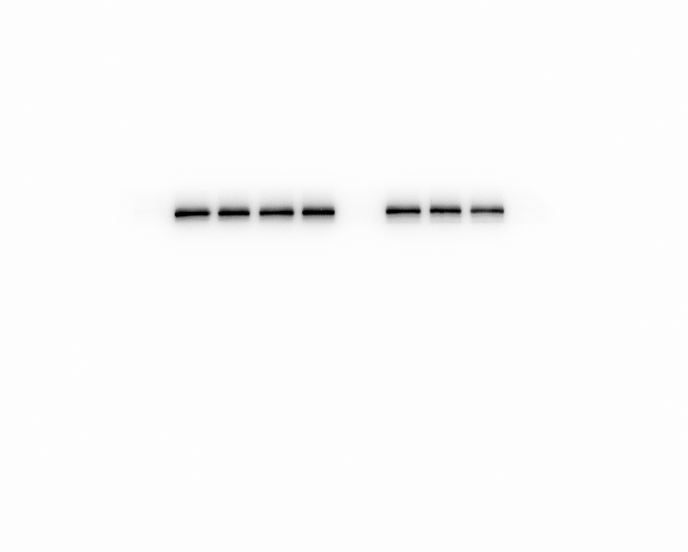

Supplement: Supplementary file 7 [file DataSheet5.zip › Fig.8/OE METTL3/GAPDH/3-GAPDH.tif]

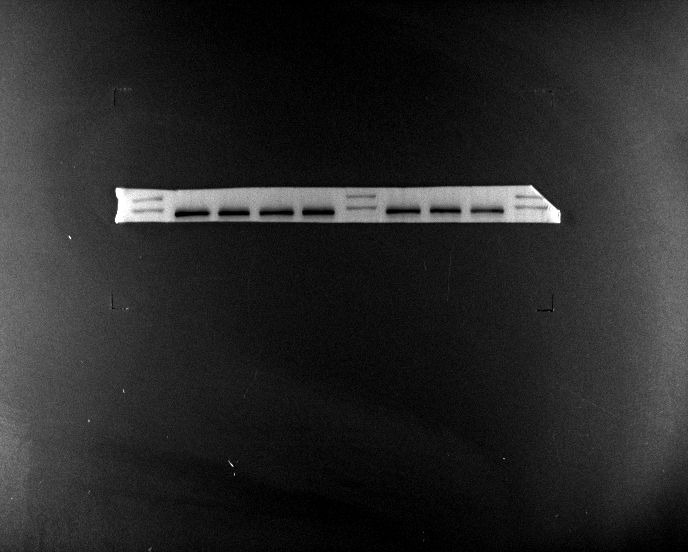

Supplement: Supplementary file 7 [file DataSheet5.zip › Fig.8/OE METTL3/GAPDH/3-GAPDH YT.tif]

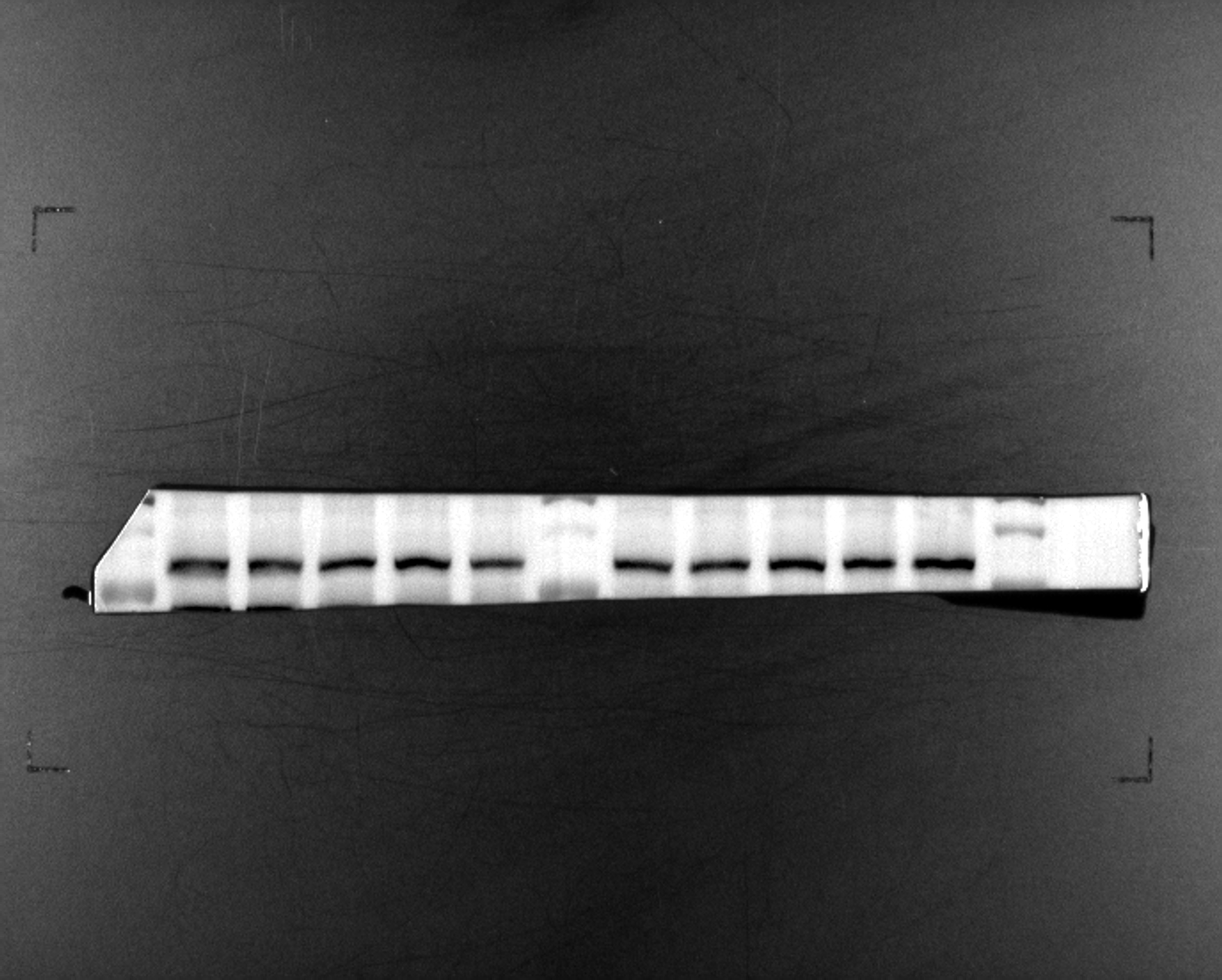

Supplement: Supplementary file 7 [file DataSheet5.zip › Fig.8/OE METTL3/GAPDH/2-GAPDH YT.tif]

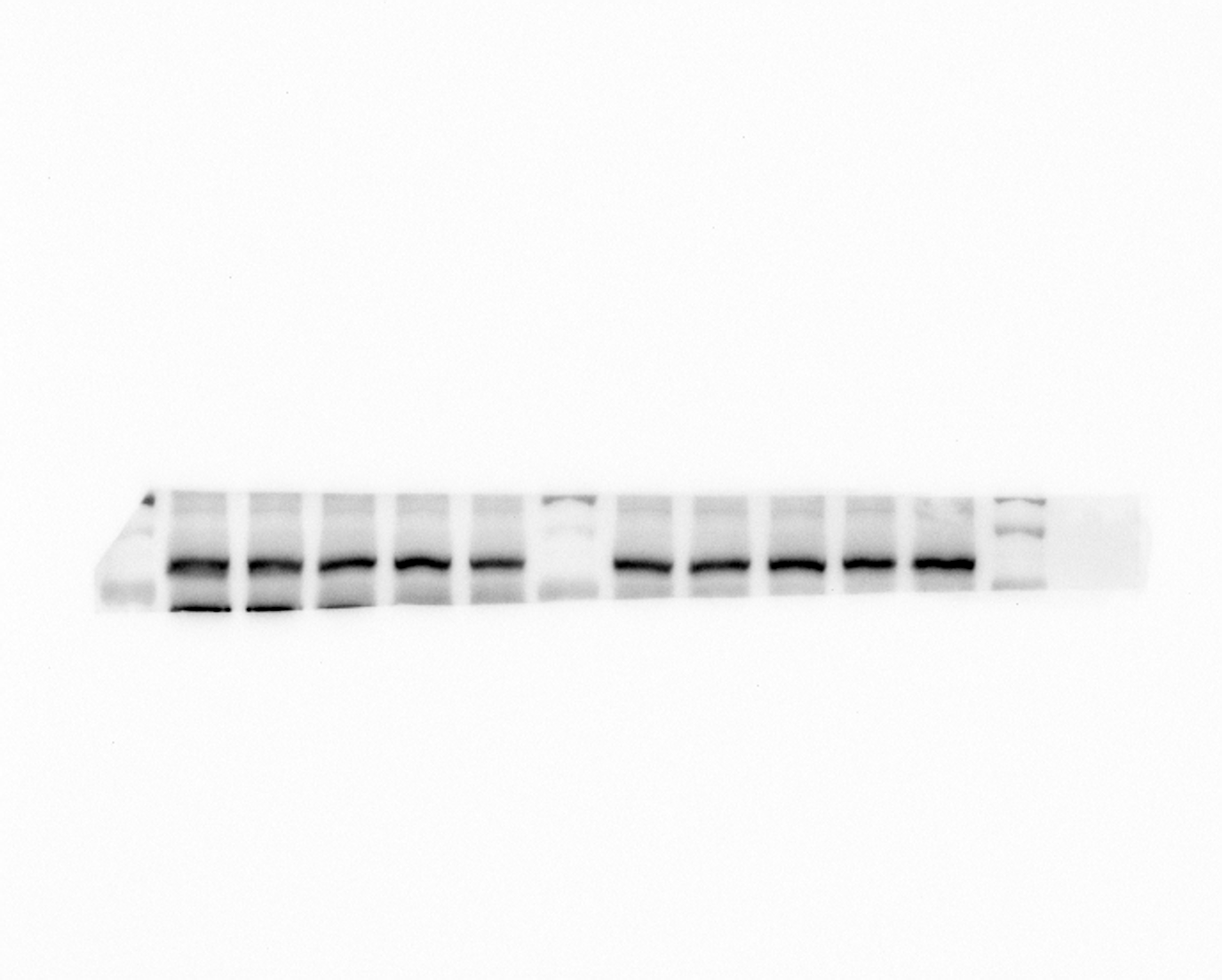

Supplement: Supplementary file 7 [file DataSheet5.zip › Fig.8/OE METTL3/GAPDH/2-GAPDH.tif]

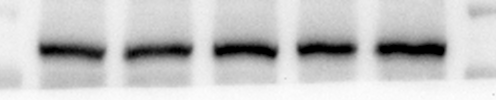

Supplement: Supplementary file 7 [file DataSheet5.zip › Fig.8/OE METTL3/GAPDH/3.tif]

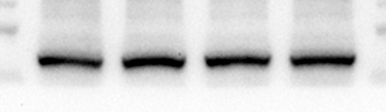

Supplement: Supplementary file 7 [file DataSheet5.zip › Fig.8/OE METTL3/GAPDH/2.tif]

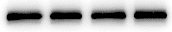

Supplement: Supplementary file 7 [file DataSheet5.zip › Fig.8/OE METTL3/GAPDH/1.tif]

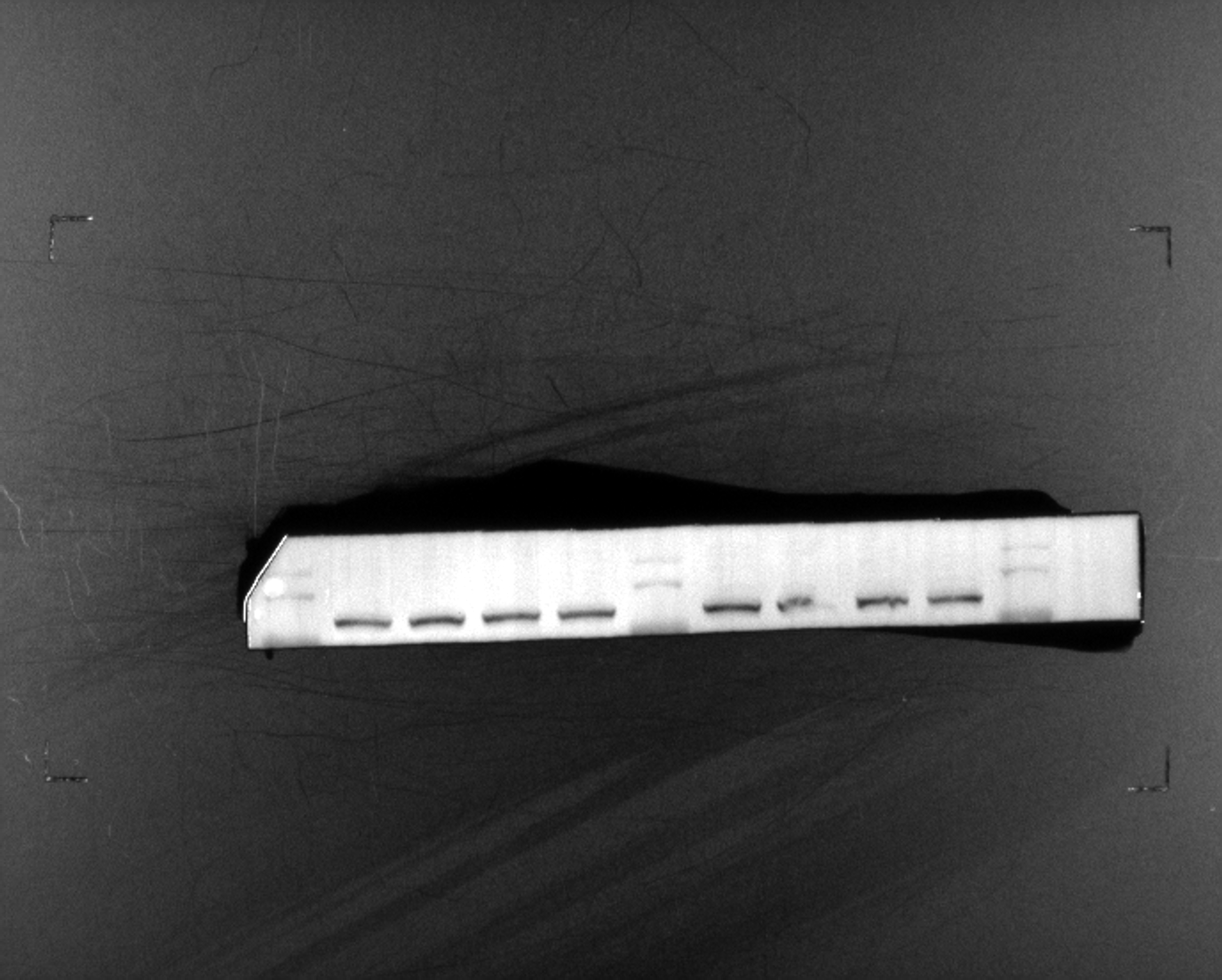

Supplement: Supplementary file 7 [file DataSheet5.zip › Fig.8/OE METTL3/GAPDH/1-GAPDH YT.tif]

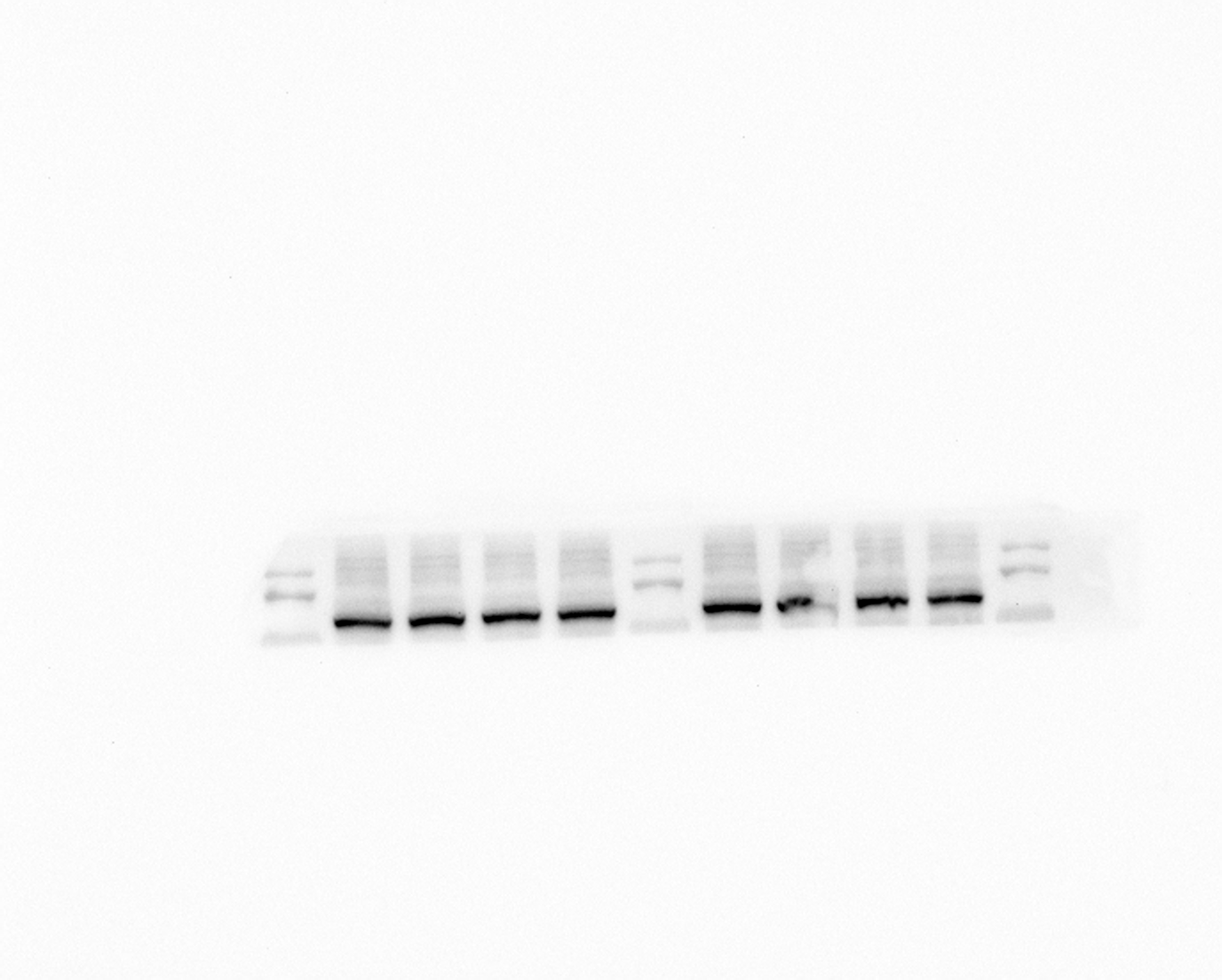

Supplement: Supplementary file 7 [file DataSheet5.zip › Fig.8/OE METTL3/GAPDH/1-GAPDH.tif]

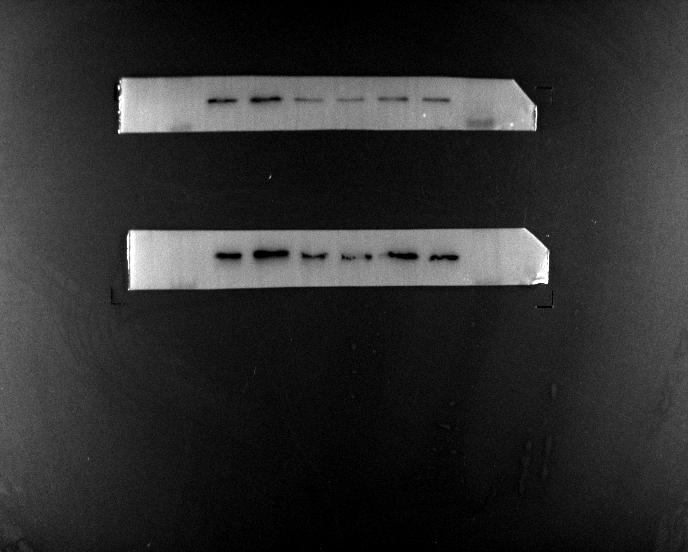

Supplement: Supplementary file 8 [file DataSheet7.zip › Fig.10/1. GSDMD/1-GSDMD-N YT.tif]

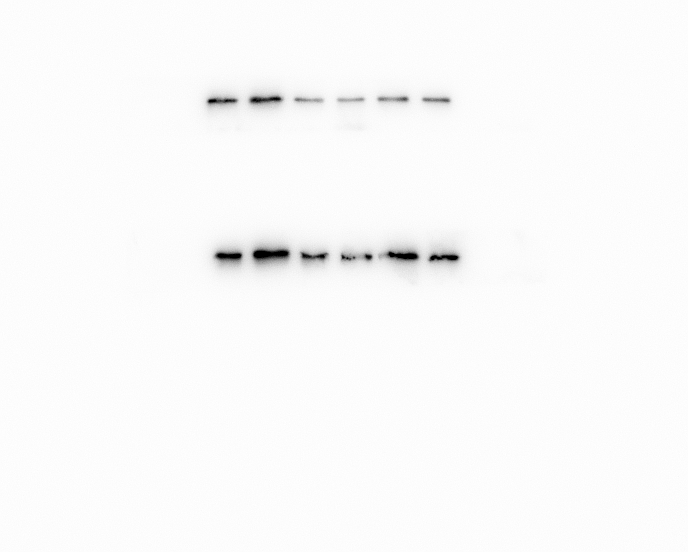

Supplement: Supplementary file 8 [file DataSheet7.zip › Fig.10/1. GSDMD/1-GSDMD-N.tif]

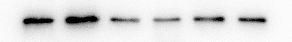

Supplement: Supplementary file 8 [file DataSheet7.zip › Fig.10/1. GSDMD/1.tif]

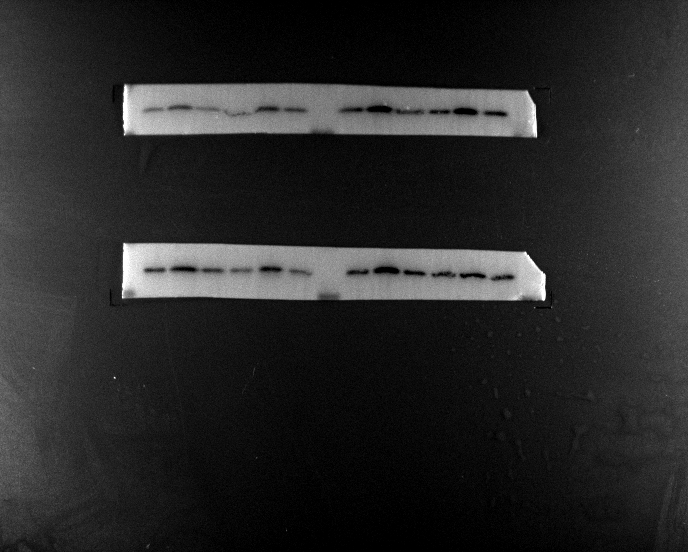

Supplement: Supplementary file 8 [file DataSheet7.zip › Fig.10/1. GSDMD/2-3-GSDMD-N YT.tif]

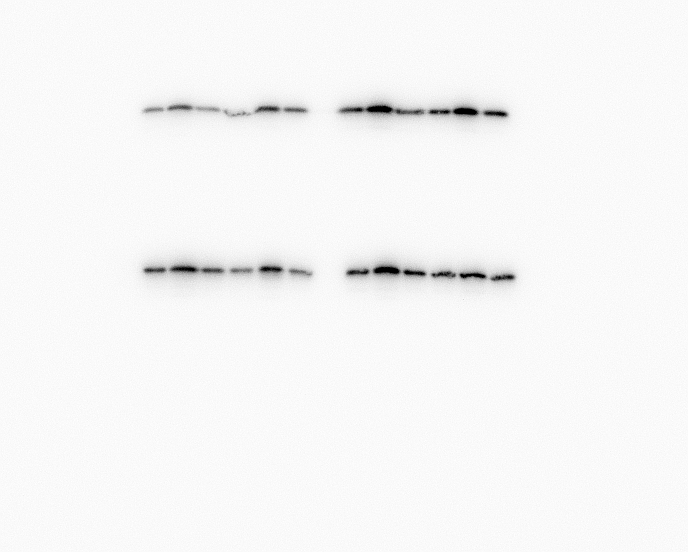

Supplement: Supplementary file 8 [file DataSheet7.zip › Fig.10/1. GSDMD/2-3-GSDMD-N.tif]

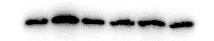

Supplement: Supplementary file 8 [file DataSheet7.zip › Fig.10/1. GSDMD/2.tif]

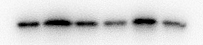

Supplement: Supplementary file 8 [file DataSheet7.zip › Fig.10/1. GSDMD/3.tif]

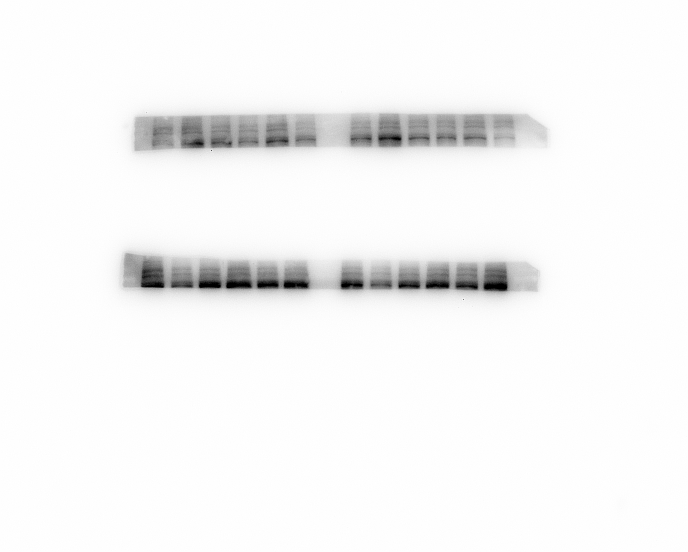

Supplement: Supplementary file 8 [file DataSheet7.zip › Fig.10/2. NLRP3/1-NLRP3.tif]

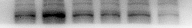

Supplement: Supplementary file 8 [file DataSheet7.zip › Fig.10/2. NLRP3/1.tif]

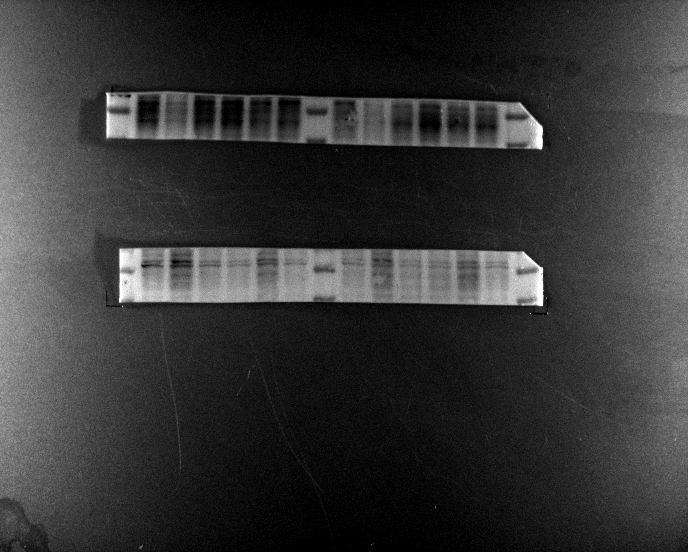

Supplement: Supplementary file 8 [file DataSheet7.zip › Fig.10/2. NLRP3/2-3-NLRP3 YT.tif]

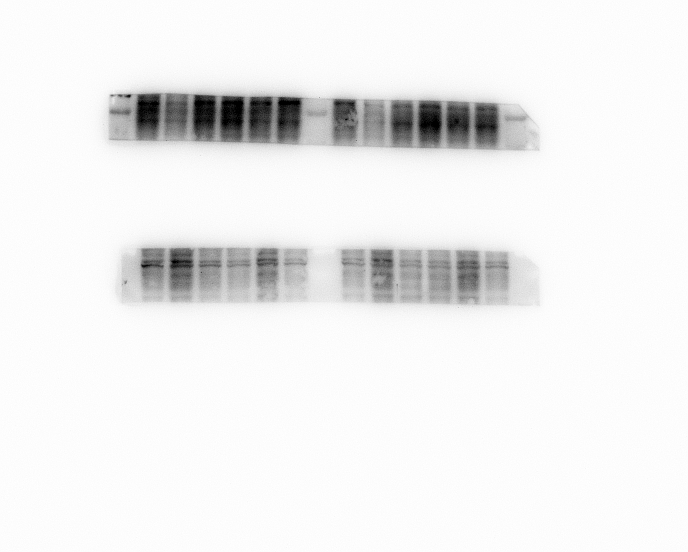

Supplement: Supplementary file 8 [file DataSheet7.zip › Fig.10/2. NLRP3/2-3-NLRP3.tif]

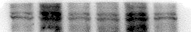

Supplement: Supplementary file 8 [file DataSheet7.zip › Fig.10/2. NLRP3/2.tif]

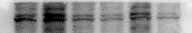

Supplement: Supplementary file 8 [file DataSheet7.zip › Fig.10/2. NLRP3/3.tif]

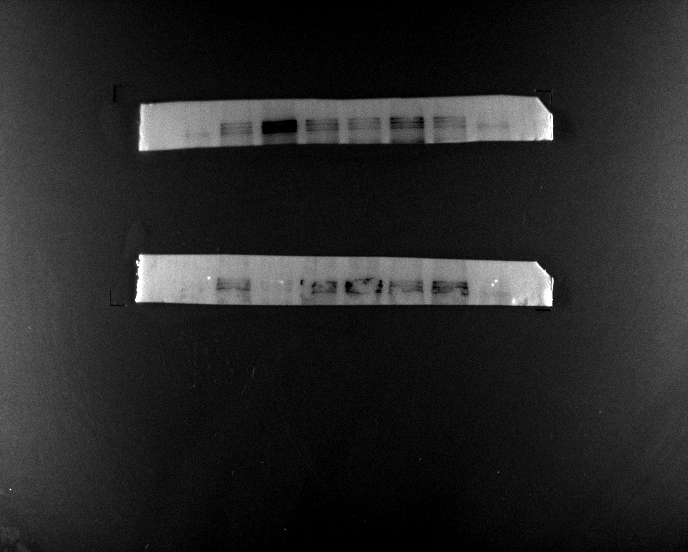

Supplement: Supplementary file 8 [file DataSheet7.zip › Fig.10/2. NLRP3/4-NLRP3 YT.tif]

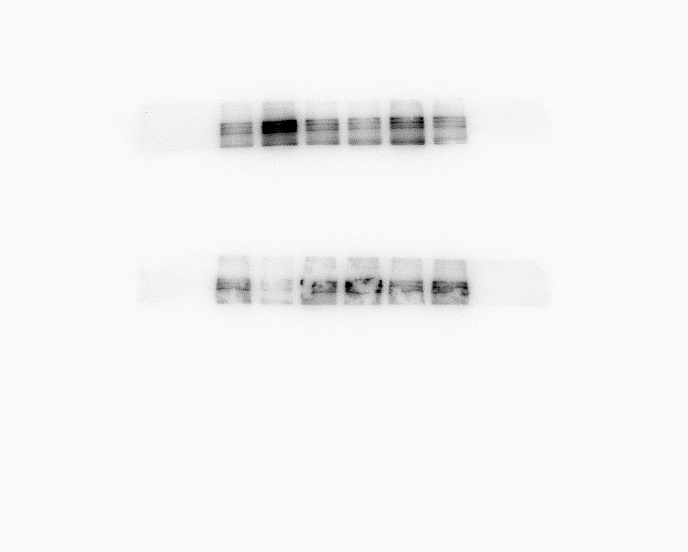

Supplement: Supplementary file 8 [file DataSheet7.zip › Fig.10/2. NLRP3/4-NLRP3.tif]

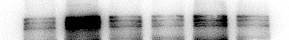

Supplement: Supplementary file 8 [file DataSheet7.zip › Fig.10/2. NLRP3/4.tif]

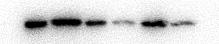

Supplement: Supplementary file 8 [file DataSheet7.zip › Fig.10/3. Caspase-1/1.tif]

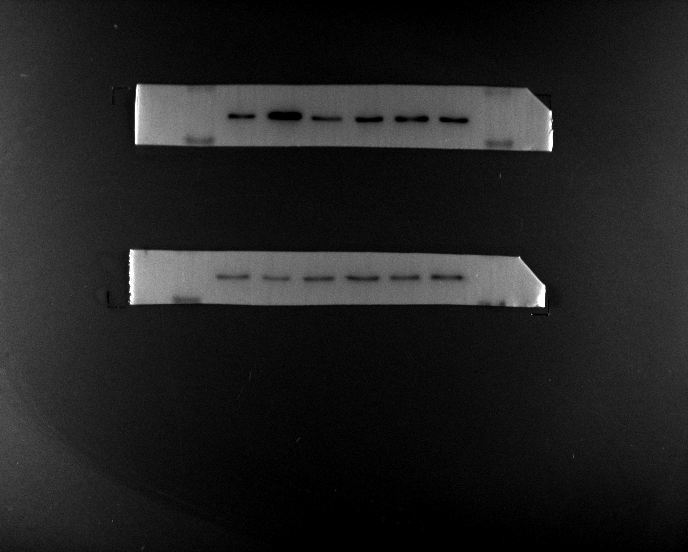

Supplement: Supplementary file 8 [file DataSheet7.zip › Fig.10/3. Caspase-1/2-Caspase YT.tif]

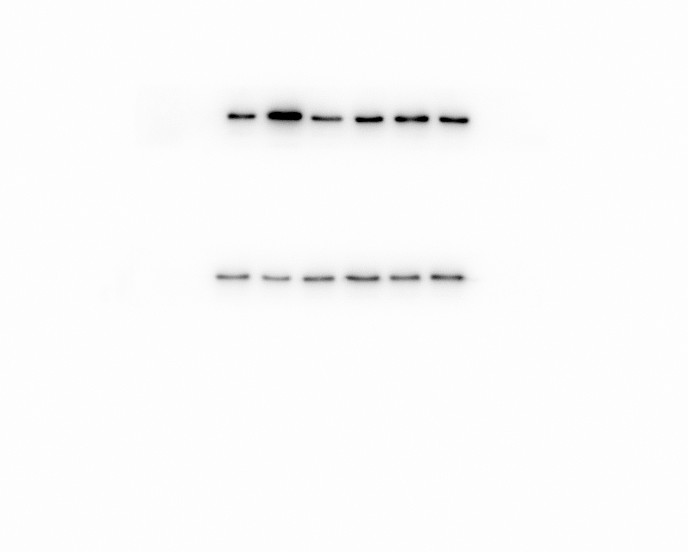

Supplement: Supplementary file 8 [file DataSheet7.zip › Fig.10/3. Caspase-1/2-Caspase.tif]

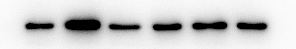

Supplement: Supplementary file 8 [file DataSheet7.zip › Fig.10/3. Caspase-1/2.tif]

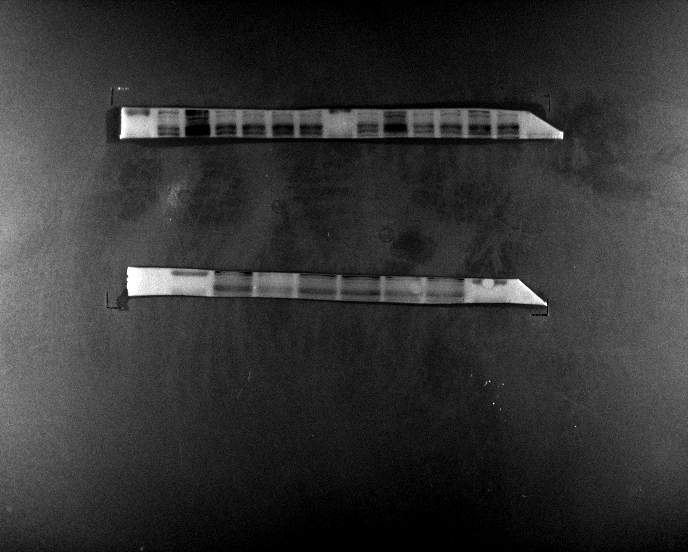

Supplement: Supplementary file 8 [file DataSheet7.zip › Fig.10/3. Caspase-1/3-4-Caspase YT.tif]

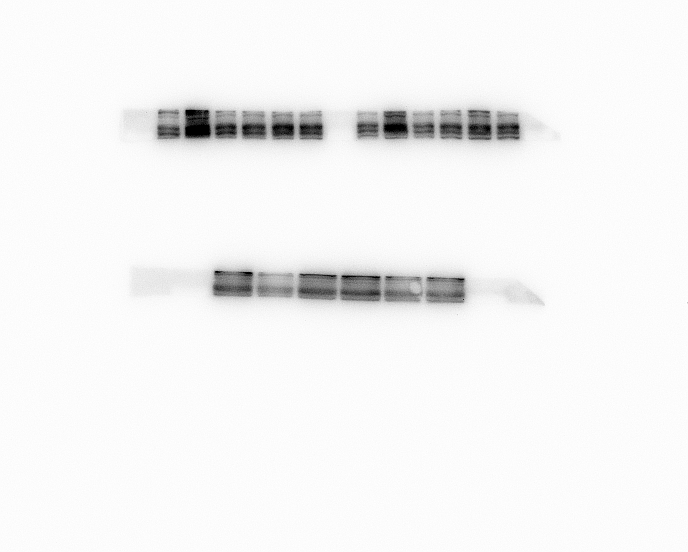

Supplement: Supplementary file 8 [file DataSheet7.zip › Fig.10/3. Caspase-1/3-4-Caspase.tif]

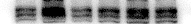

Supplement: Supplementary file 8 [file DataSheet7.zip › Fig.10/3. Caspase-1/3.tif]

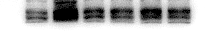

Supplement: Supplementary file 8 [file DataSheet7.zip › Fig.10/3. Caspase-1/4.tif]

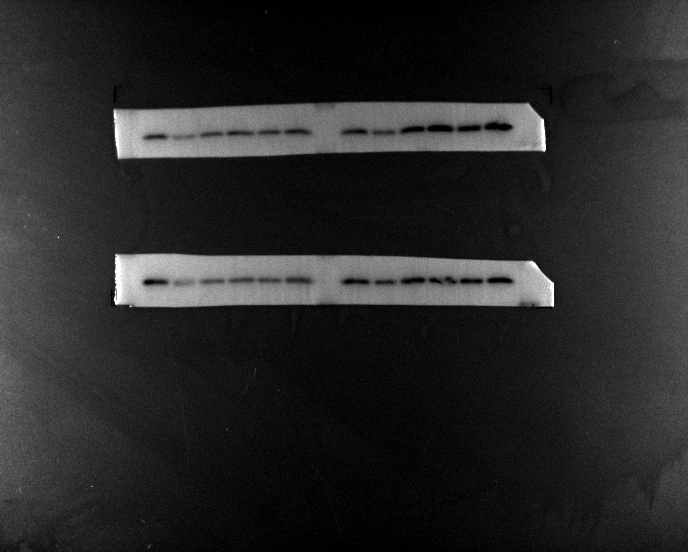

Supplement: Supplementary file 8 [file DataSheet7.zip › Fig.10/4. nephrin/1-Nephrin YT.tif]

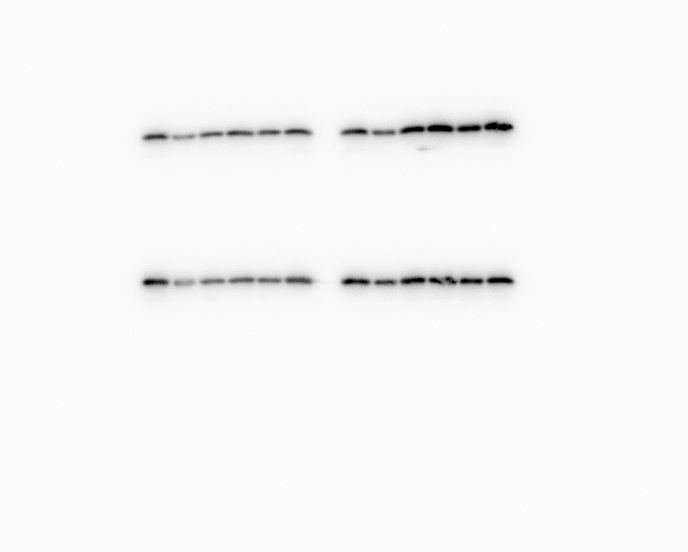

Supplement: Supplementary file 8 [file DataSheet7.zip › Fig.10/4. nephrin/1-Nephrin.tif]

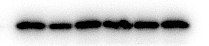

Supplement: Supplementary file 8 [file DataSheet7.zip › Fig.10/4. nephrin/1.tif]

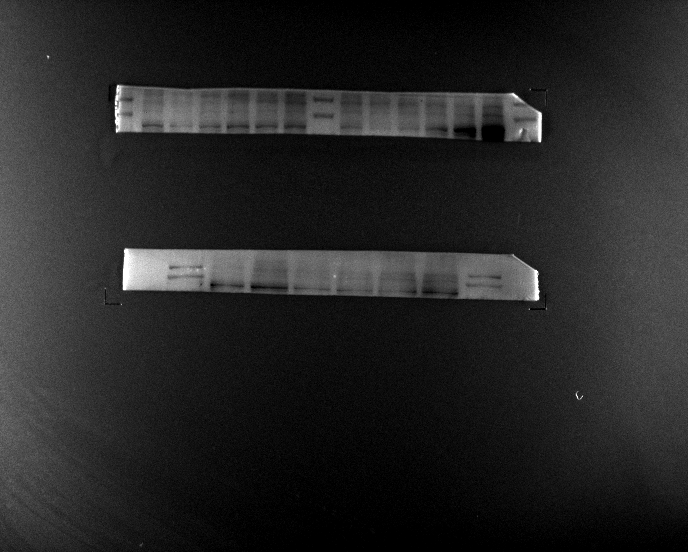

Supplement: Supplementary file 8 [file DataSheet7.zip › Fig.10/4. nephrin/2-Nephrin YT.tif]

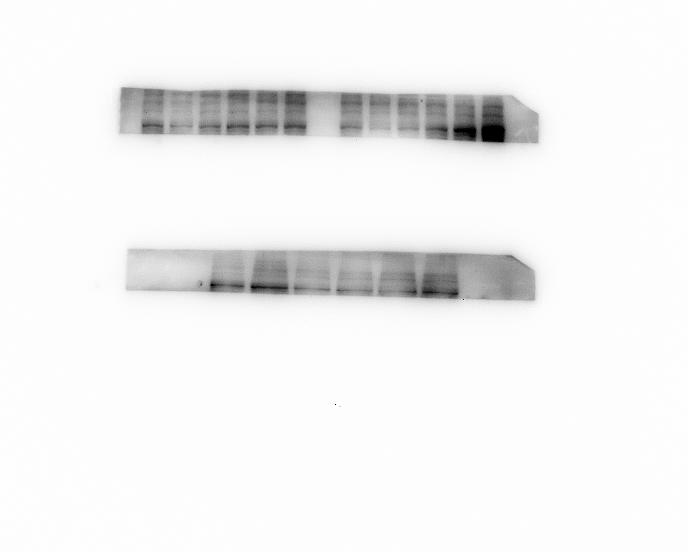

Supplement: Supplementary file 8 [file DataSheet7.zip › Fig.10/4. nephrin/2-Nephrin.tif]

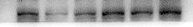

Supplement: Supplementary file 8 [file DataSheet7.zip › Fig.10/4. nephrin/2.tif]

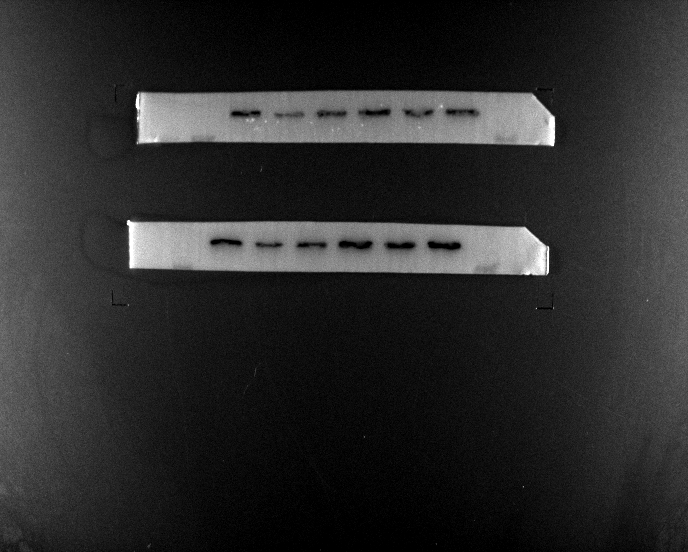

Supplement: Supplementary file 8 [file DataSheet7.zip › Fig.10/4. nephrin/3-Nephrin YT.tif]

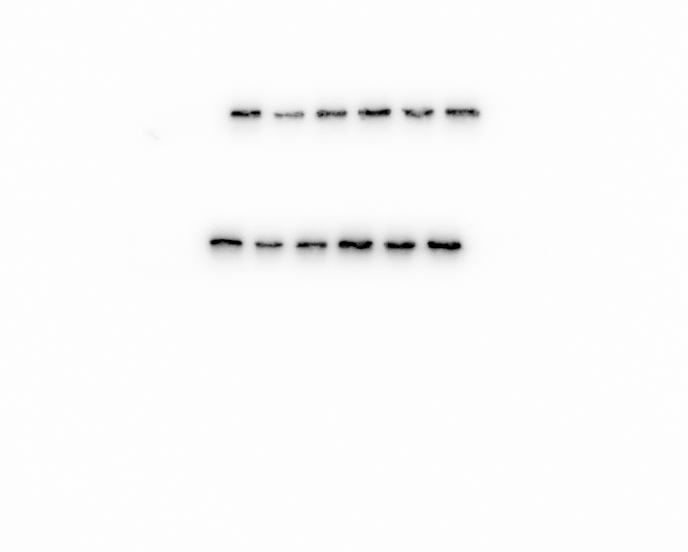

Supplement: Supplementary file 8 [file DataSheet7.zip › Fig.10/4. nephrin/3-Nephrin.tif]

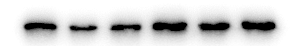

Supplement: Supplementary file 8 [file DataSheet7.zip › Fig.10/4. nephrin/3.tif]

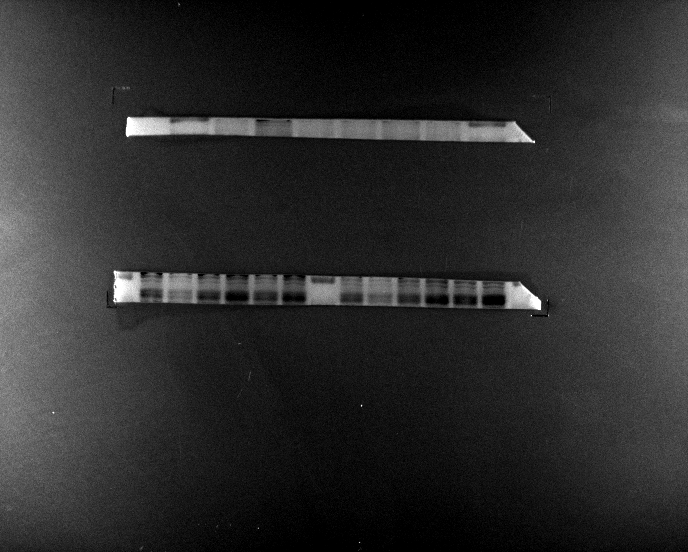

Supplement: Supplementary file 8 [file DataSheet7.zip › Fig.10/5. ZO-1/1-ZO1 YT.tif]

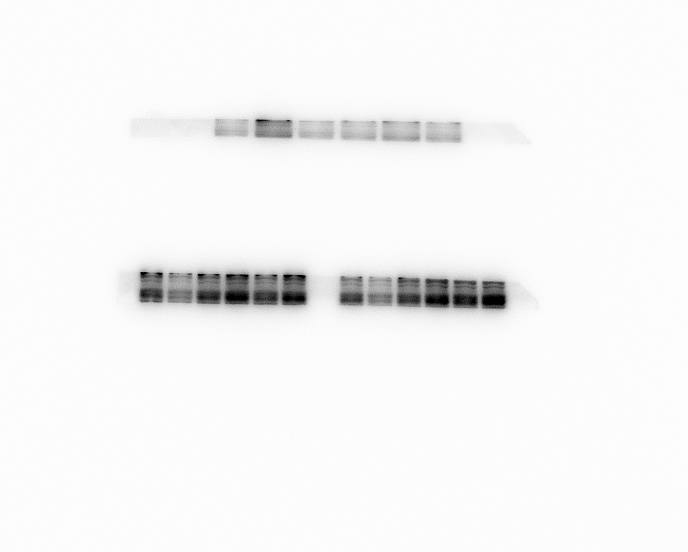

Supplement: Supplementary file 8 [file DataSheet7.zip › Fig.10/5. ZO-1/1-ZO1.tif]

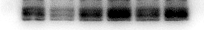

Supplement: Supplementary file 8 [file DataSheet7.zip › Fig.10/5. ZO-1/1.tif]

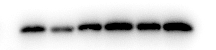

Supplement: Supplementary file 8 [file DataSheet7.zip › Fig.10/5. ZO-1/2.tif]

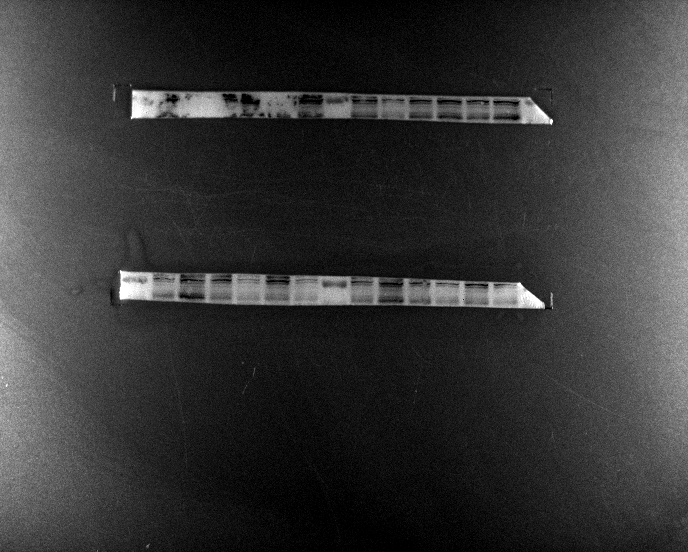

Supplement: Supplementary file 8 [file DataSheet7.zip › Fig.10/5. ZO-1/3-ZO1 YT.tif]

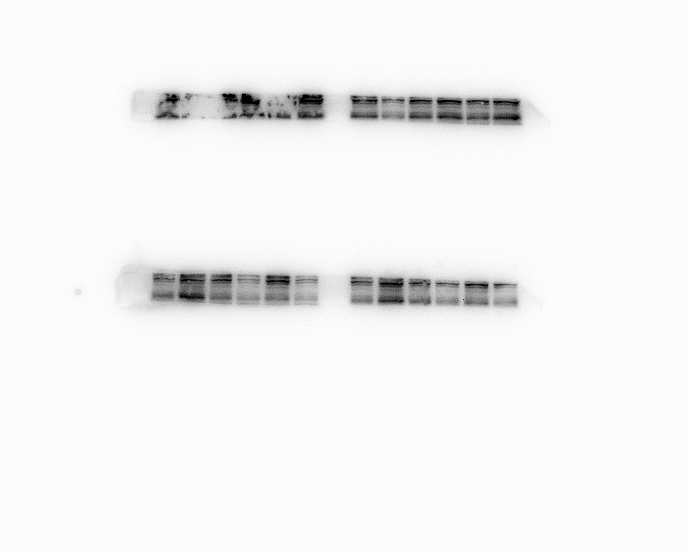

Supplement: Supplementary file 8 [file DataSheet7.zip › Fig.10/5. ZO-1/3-ZO1.tif]
