# Supplementary figures and images for: Total Flavones of Abelmoschus manihot Ameliorates Podocyte Pyroptosis and Injury in High Glucose Conditions by Targeting METTL3-Dependent m6A Modification-Mediated NLRP3-Inflammasome Activation and PTEN/PI3K/Akt Signaling (part 6 of 6)
Source: Front Pharmacol. 2021 Jul 15;12:667644. doi: 10.3389/fphar.2021.667644 (PMC8319635; doi:10.3389/fphar.2021.667644)

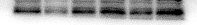

Supplement: Supplementary file 8 [file DataSheet7.zip › Fig.10/5. ZO-1/3.tif]

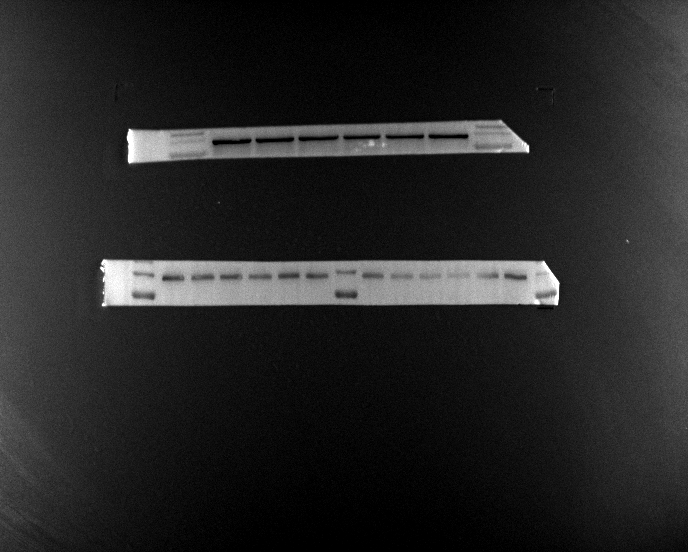

Supplement: Supplementary file 8 [file DataSheet7.zip › Fig.10/6.GAPDH/1-2-GAPDH YT.tif]

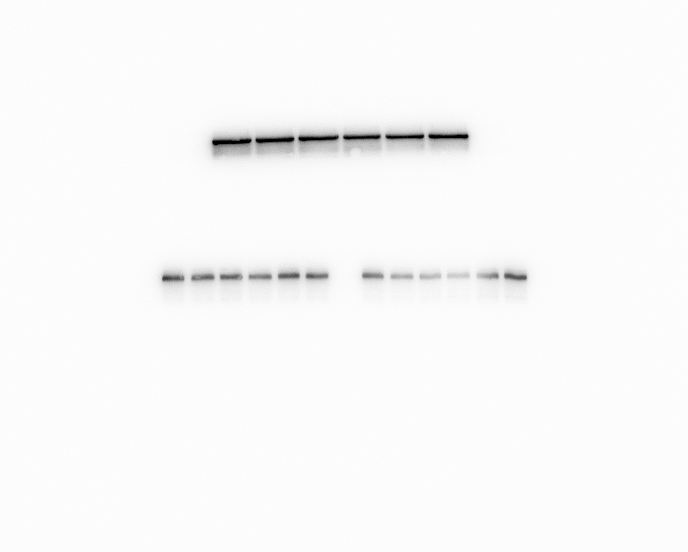

Supplement: Supplementary file 8 [file DataSheet7.zip › Fig.10/6.GAPDH/1-2-GAPDH.tif]

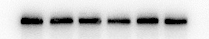

Supplement: Supplementary file 8 [file DataSheet7.zip › Fig.10/6.GAPDH/1.tif]

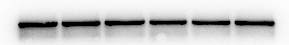

Supplement: Supplementary file 8 [file DataSheet7.zip › Fig.10/6.GAPDH/2.tif]

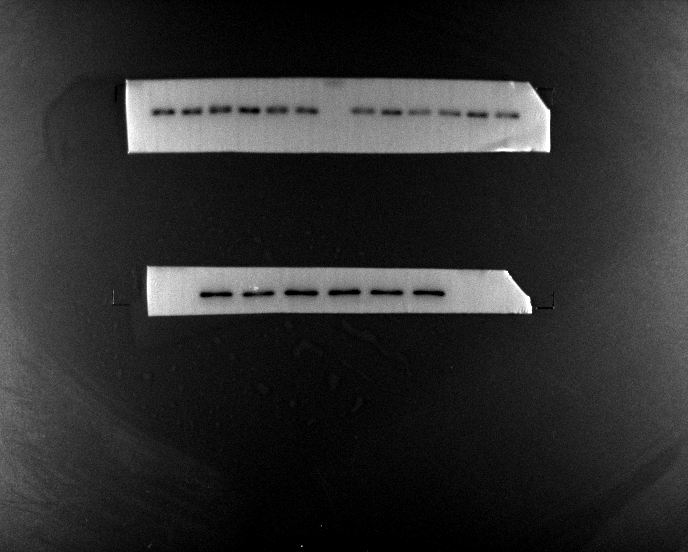

Supplement: Supplementary file 8 [file DataSheet7.zip › Fig.10/6.GAPDH/3-GAPDH YT.tif]

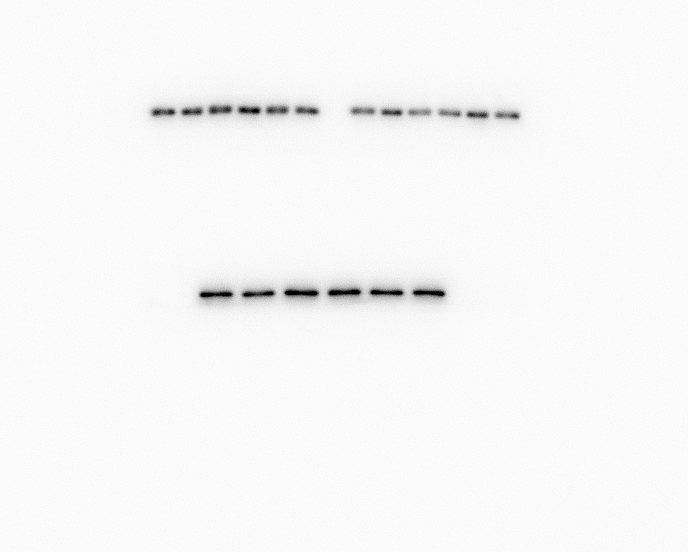

Supplement: Supplementary file 8 [file DataSheet7.zip › Fig.10/6.GAPDH/3-GAPDH.tif]

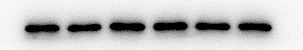

Supplement: Supplementary file 8 [file DataSheet7.zip › Fig.10/6.GAPDH/3.tif]
